# Micellar Mechanisms for Desymmetrization Reactions in Aqueous Media

**DOI:** 10.1021/acsomega.3c04318

**Published:** 2023-09-05

**Authors:** Satomi Niwayama, Yoshikazu Hiraga

**Affiliations:** † Graduate School of Engineering, Muroran Institute of Technology, Muroran, Hokkaido 050-8585, Japan; ‡ Department of Food Sciences and Biotechnology, Hiroshima Institute of Technology, Hiroshima 731-5193, Japan

## Abstract

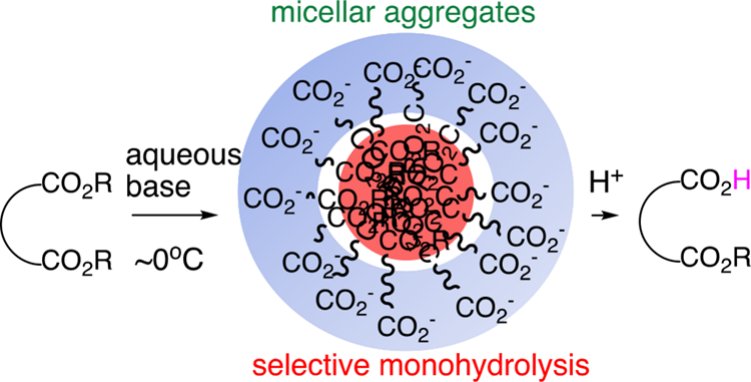

Water-mediated organic reactions significantly contribute to the protection of the environment. Desymmetrization reactions, which convert only one of the identical functional groups within one molecule, are cost-effective because of the low cost of the starting materials. In combination with these two merits, highly efficient and practical selective monohydrolysis reactions of symmetric diesters were previously reported. The products of these reactions are versatile building blocks. The mechanisms of this reaction are hypothesized to proceed through micellar aggregates in which the hydrophilic carboxylate anions formed by monohydrolysis are directed outward and the remaining hydrophobic groups are directed inward in governing the selectivities. Here, dynamic light scattering and electrophoretic light scattering experiments were performed for detection of the key intermediates in the reaction. These experiments revealed the existence of aggregates with negative charges on the surface in the mainly aqueous media, supporting the reaction mechanisms that control the high selectivities.

## Introduction

The development of cost-effective and environmentally benign organic reactions has long been of paramount importance. Water is among the most environmentally friendly and least expensive solvents. Desymmetrization of symmetric compounds is one of the most cost-effective reactions because the starting symmetric compounds are typically easy to obtain on a large scale from inexpensive sources or are commercially available at a low cost. Therefore, the development of efficient processes for the desymmetrization of symmetric compounds in aqueous media would make a significant contribution to “green chemistry”.^1,2^ In fact, many products of enzymatic desymmetrization reactions have been applied to the synthesis of various significant natural products or phrarmaceuticals,^3−9^ although unfortunately, the mechanisms about enzymatic desymmetrization reactions remain largely unsolved, and thus, random screening of enzymes and substrate symmetric compounds is still required for desirable outcomes.

The number of successful organic reactions without enzymes with high yields and high selectivities in aqueous media remains limited because of the hydrophobicities and limited solubilities of organic compounds in water. Furthermore, the reactions in water reported thus far do not necessarily show improvement in reactivity and/or selectivity over those in organic solvents, and many of them require a number of steps before the reactions can be conducted in aqueous media.

**Figure 1:**
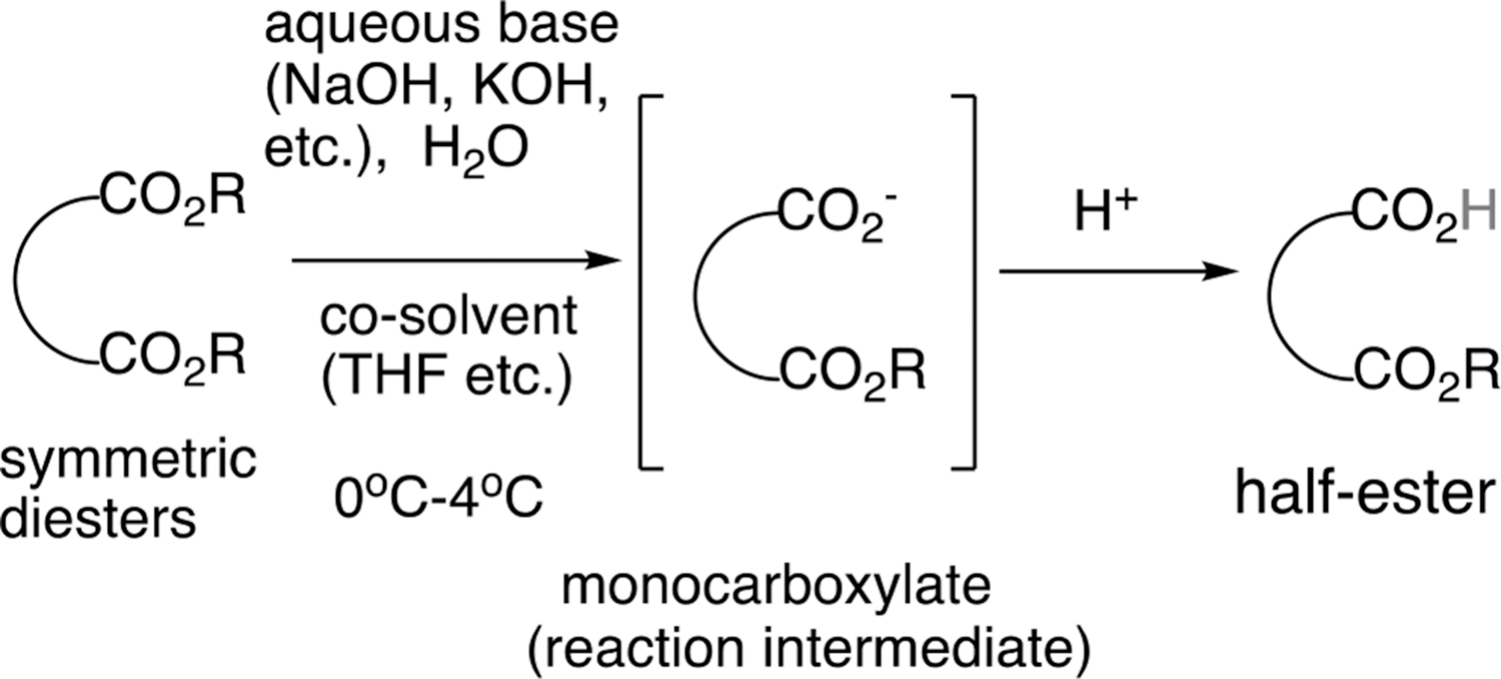
Selective monohydrolysis of symmetric diesters. Only one of the two identical ester groups is hydrolyzed with high selectivity under practical conditions.

Earlier, we reported the water-mediated desymmetrization reactions—monohydrolysis of symmetric diesters—which exhibit some of the highest selectivities^10−20^ (Figure 1 and Table 1). These reactions were among the first examples of water-mediated desymmetrization reactions. Half-esters produced in these reactions are very versatile building blocks applied to the synthesis of pharmaceuticals, natural products, and polymers.^21−31^ These reactions can produce the corresponding half-esters even in near-quantitative yields in many cases.

**Table 1:**
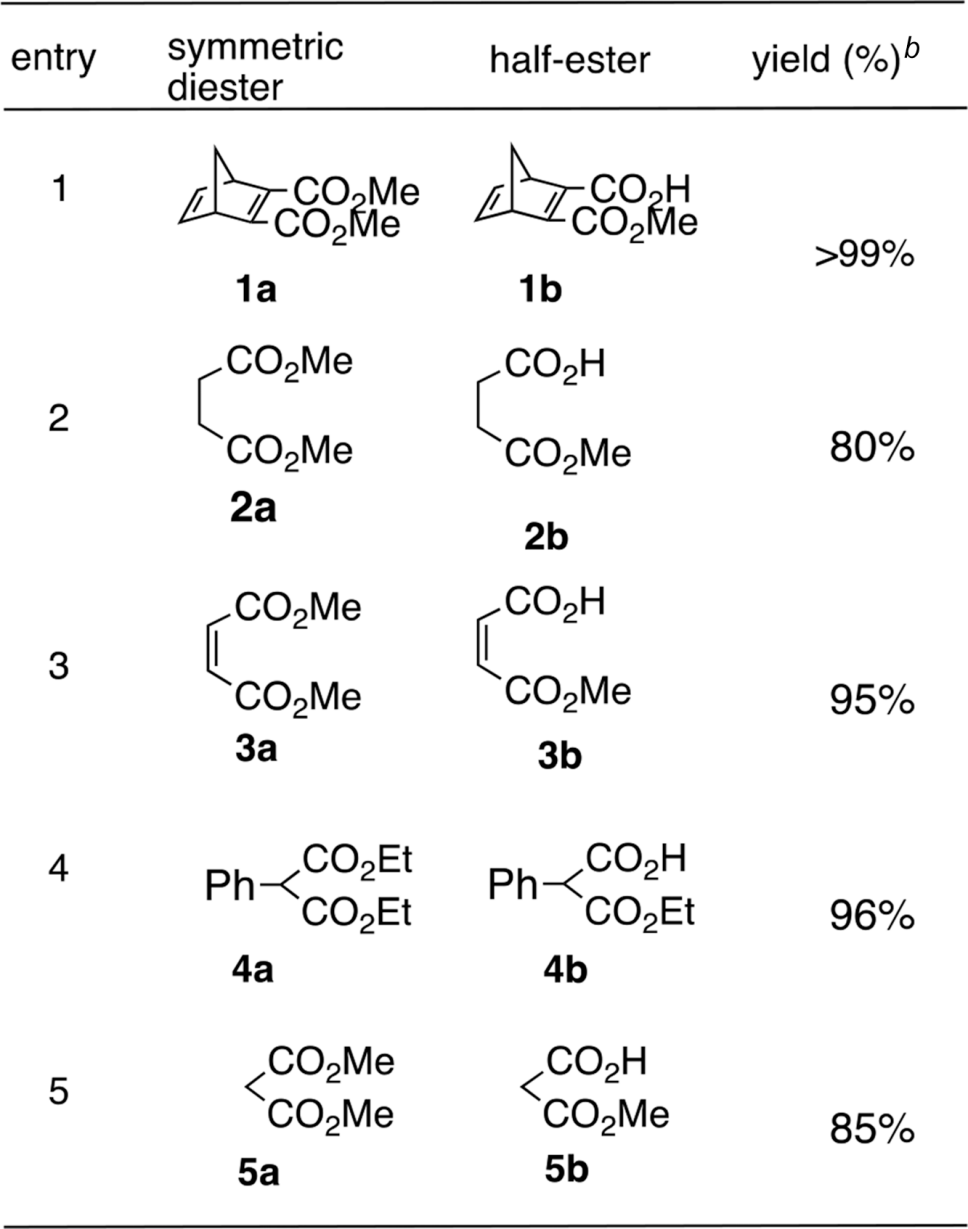
Examples of Selective Monohydrolysis of Symmetric Diesters^a^

a These examples illustrate the structures of the sample analytes in this study. Many more cases exhibiting high yields have been reported.

b Isolated yields.

Under the classical conditions reported previously, among the most typical methods for the synthesis of half-esters are selective monosaponification reactions of symmetric diesters with the use of solid NaOH or KOH and alcoholic solvents such as ethanol or methanol, which typically yield complex mixtures. For example, in our experiments, monosaponification of symmetric diester **1a** with one equivalent of NaOH in methanol yielded only a small amount of the half-ester, **1b**, with a complex mixture as yellowish oil accompanying the starting diester and the corresponding diacid in which both the ester groups were hydrolyzed.^15^

Conversely, the above selective monohydrolysis reaction is carried out by the addition of an aqueous base such as aqueous NaOH or KOH to an aqueous suspension of a symmetric diester that may contain a small amount (<7%) of a polar aprotic co-solvent such as tetrahydrofuran (THF) at 0–4 °C. Under these simple conditions, this reaction produces pure half-esters in high to near-quantitative yields, even with more than one equivalent of the base in many cases. The reaction mixture is also quite clean, showing only the half-ester along with small amounts of the starting diester and diacid, if they existed. Unfortunately, the mechanisms for the selectivities of this reaction under such simple conditions have remained long unsolved.

**Figure 2:**
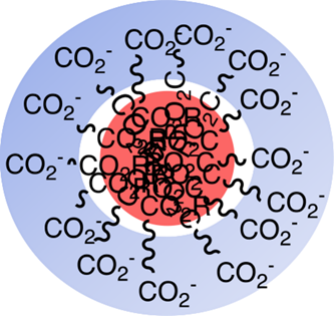
Potential aggregates of the reaction intermediate. In aqueous media, hydrophobic portions are pointed inside, keeping away from exposure to the aqueous base. The aggregates may further congregate.

In order to explain the mechanism of the selective monohydrolysis reactions of symmetric diesters, we hypothesize that once one of the carboalkoxy groups is converted to a carboxylate anion, it may form micelle-like aggregates in which the hydrophilic carboxylates (COO^–^) are pointed outside and the hydrophobic carboalkoxy groups along with other hydrophobic portions are pointed inside (Figure 2). In this way, the remaining carboalkoxy group is protected from further hydrolysis in the aqueous media. However, there was no experimental verification to substantiate the hypothesis.

Here, we attempted to detect such aggregates by dynamic light scattering (DLS) experiments and electrophoretic light scattering (ELS) experiments. DLS is a technique that allows measurement of size profiles of small particles including colloidal particles by scattering of laser. ELS allows measurement of the zeta potential, which is a measure of charges by particles suspended in a liquid such as water. Colloidal particles suspended in water or aqueous liquids generally carry double layers consisting of the Stern layer and the diffuse layer. The zeta potential indicates the electric potential from the slipping plane, which is the furthermost region that the surface change can influence in the double layer. These techniques provide insight into aggregative stabilities of colloidal aggregates and are standard for characterization of various colloidal dispersions such as nanoparticles, often for pharmaceuticals, cosmetics, paints, and ink applications.^32−37^ We therefore expect that detection of these data from the reaction intermediates, which are carboxylate anions of half-esters, proves a certain stability of such charged aggregates in the solution, hence proving the reaction mechanism.

## Results and Discussion

In the actual experimental procedures, a symmetric diester is dissolved in a small amount of a polar aprotic co-solvent (such as THF) and water is added, and at least one equivalent of NaOH or KOH aqueous solution is added at 0–4 °C. After monohydrolysis has been completed, the acidification of the reaction mixture with diluted aqueous HCl and subsequent purification affords the corresponding half-ester in high yields. In the monohydrolysis reaction mixture, the produced half-ester is a sodium salt of monocarboxylate when the NaOH aqueous solution is utilized, and it is a potassium salt of monocarboxylate when the KOH aqueous solution is utilized. Therefore, we decided to observe DLS and ELS using sodium monocarboxylates or potassium monocarboxylates in various concentrations. In fact, when several kinds of sodium salts of carboxylate anions are dissolved in water containing a small amount of THF as a co-solvent, reproducing the reaction media, after quenching with diluted HCl, the half-esters were quantitatively recovered. In one instance (diester **1a**), the carboxylate anion solutions thus prepared and the intermediary carboxylate anion solution from the actual reaction mixture of monohydrolysis of **1a** showed essentially the same results in the DLS and ELS measurement (data not shown). Therefore, we believe that carboxylate anions thus prepared serve as the equivalent of the intermediary carboxylate anion in the above selective monohydrolysis reaction.

The sodium salts of the four half-esters **1b′–4b′** thus prepared and the potassium salt of the half-ester **5b′** were dissolved in H_2_O containing a small amount of THF (THF:H_2_O = 2:28) and were adjusted to be various concentrations ranging from 10 to 250 mM. The final proportion of THF to water was adjusted to be the same as that of the real reaction conditions reported before.^10^ The size distribution profile and the zeta potential of the sample solutions were measured at 4 °C with the use of a PLS-450 (Otsuka Electronics, Japan).

Table 2 and Figures S1–S5 summarize the results from the DLS and ELS experiments.

**Table 2:**
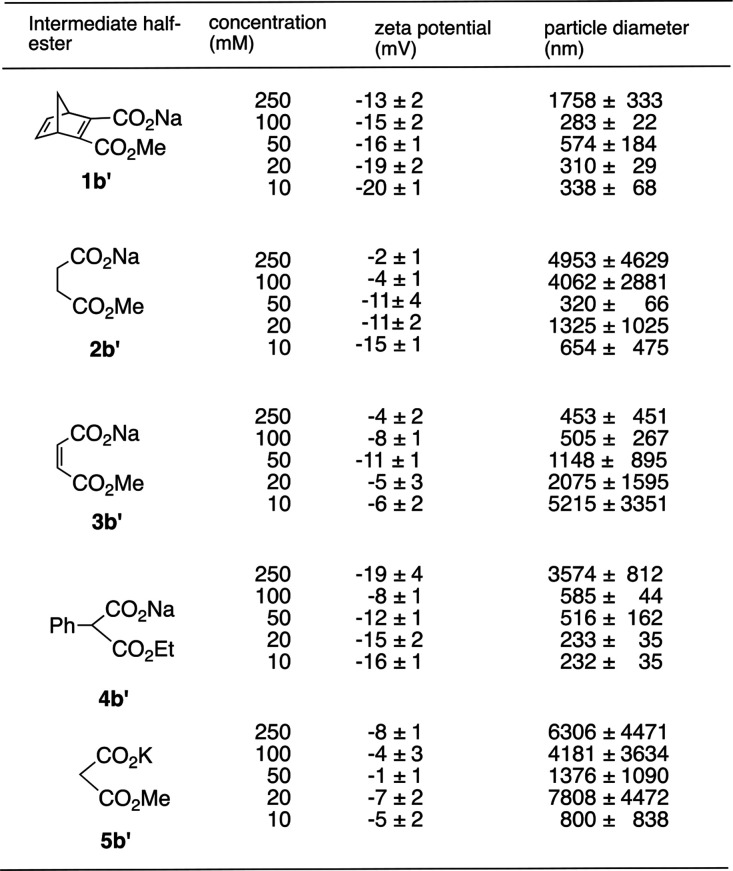
Zeta Potential and Particle Diameter of Various Intermediates in Aqueous Media at 4 °C^a^

a The negative zeta potential values indicate that the aggregates with the observed diameters are negatively charged on the surface.

Measurement of the solutions containing each of the five kinds of monocarboxylates of **1b′**, **2b′**, **3b′**, **4b′**, and **5b′**, respectively, revealed the existence of aggregative particles in a range of around 200–7000 nm in the solution with the same conditions as the actual reaction conditions. They are the intermediates produced from the five kinds of symmetric diesters in the above selective monohydrolysis reaction. These particles also show zeta potential values of around −1 to −20 mV, indicating that these aggregates are negatively charged on the surface, and these particles are moderately stable as colloids.^38,39^ Based on these data, we deduce that the micelle-like aggregates in which the COO^–^ groups are pointed outside are formed as the reaction intermediate, prohibiting further hydrolysis as in our hypothesis.

According to these data, the particles with lower concentrations tend to show a higher zeta potential than the particles with higher concentrations in some compounds. It is known that concentrated suspensions show counterion compression in the slipping plane of particles, shifting the electronic potential inward, leading to a reduced zeta potential. Such phenomena have also been reported previously as exemplified in the concentration dependence by polymer latex.^40^ The zeta potential values also tend to be maximally negative when the concentrations are in the range of approximately 10–50 mM, which roughly corresponds to typical concentrations in this selective monohydrolysis reaction.^10^ These zeta potentials tend be high for the carboxylates that exhibited high selectivities in the reaction. The particle sizes observed by DLS tend to be large when the zeta potential values are small or the concentrations are high perhaps due to the increased van der Waals interaction between the colloid particles,^41^ hence forming bigger aggregates. Therefore, all these data construe typical colloidal characteristics.

In addition, earlier we reported that a non-covalent interaction, n → π* interaction, between the two proximally oriented carbonyl groups in a predominant conformation of symmetric diesters is likely to play a key role discriminating the two identical carbonyl groups before forming the monocarboxylates.^42,43^ The fact that the intermediary aggregates are moderately stable may suggest that the contribution of the n → π* interaction between the two carbonyl groups, hence the structure of the starting diesters, plays a nearly equally important role governing the selectivities.

This mechanism also explains the influence of co-solvent in the selective monohydrolysis reaction we reported before.^13^ The monohydrolysis reaction can proceed only in an aqueous base without a co-solvent, but a small amount (<7%) of a polar aprotic water-miscible co-solvent such as THF, acetonitrile, *N*,*N*-dimethylformamide, or dimethyl sulfoxide can accelerate the reaction rate with high selectivity. While these co-solvents dissolve in water and form one aqueous phase in the reaction media, these co-solvents are likely to help disperse the starting diester in the aqueous reaction media and help increase the contact between the diester and the aqueous phase before the monohydrolysis actually occurs, still maintaining the intermediary aggregates. However, a protic organic co-solvent such as alcohols decrease the reaction rates and the yields of the product half-esters. These results as well as the poor selectivities in the monosaponification reactions in an alcoholic solvent mentioned above can be deduced by the formation of the above micellar aggregate in the aqueous reaction media, as the protic solvents can dissociate the formation of the aggregates because they have both hydrophobic and hydrophilic groups. In fact, we have been successful in improving the yields and selectivities in the selective monohydrolysis reactions of various symmetric diesters by tuning the reaction conditions based on this mechanism.^14,16−20^

Reactions mediated by micelle or micellar substances have been sometimes reported in the aqueous media as environmentally benign reactions. Classical examples include selective monohydroxymerculation of dienes in the presence of surfactants such as sodium lauryl sulfate (SLS),^44^ and more recently, several studies about reactions with the use of surfactants combined with Lewis acid, iodine, and other metals have been reported.^45−52^ They are found to accelerate the reaction rates or promote the selectivities in water solvent due to the amphiphilic nature. However, in this selective monohydrolysis reaction, the reaction intermediate from the starting material itself forms micellar aggregates and governs the selectivity without requiring a special additive under environmentally benign conditions. To our knowledge, this reaction is among the first examples of such reactions, although Chong et al. proposed formation of revered micellar aggregates for selective monobromination of symmetric diols in toluene-aqueous HBr solution.^53^

## Conclusions

In conclusion, by the DLS and ELS experiments of the monocarboxylates of **1b′**, **2b′**, **3b′**, **4b′**, and **5b′**, we found that the intermediary carboxylates in the selective monohydrolysis reaction form aggregates having negative surface charges in the reaction media consisting primarily of water. As the observed zeta potential values are all negative signs, they support our hypothesis that the aggregates mainly have structures in which the carboxylate anions are directed outward and the remaining ester groups and other hydrophobic portions are directed inward. These aggregates are likely to prohibit further hydrolysis of the ester group in aqueous media and therefore lead to high selectivity in the monohydrolysis reaction.

## Methods

### Preparation of Analytes

Carboxylate **5b′** was purchased from Sigma-Aldrich. All other carboxylates **1b′–4b′** that measured the DLS and ELS data were prepared from the sodium salts of the corresponding half-esters, **1b–4b**, synthesized from the corresponding symmetric diesters, **1a–4a**, by the procedure we reported before^10^ as follows: the diester (1.2 mmol) was dissolved in 2 mL of THF, and 20 mL of water was added. The reaction mixture was immersed in an ice-water bath and cooled to 0–4 °C. To this reaction mixture, 8 mL of 0.25 M NaOH was added in small portions with stirring until the consumption of the starting diester was detected by thin layer chromatography (TLC). The reaction mixture was stirred at the same temperature for about 30 min to 1 h, and the reaction mixture was acidified with 1 M HCl at 0 °C, saturated with NaCl, extracted with ethyl acetate three to four times, and dried with sodium sulfate. This extract was evaporated in vacuo and purified by silica gel column chromatography to afford the corresponding half-ester. For the preparation of the sodium salt, **1b′–4b′**, the half-esters thus purified were dissolved in an aqueous solution containing an equal mole of Na_2_CO_3_ and subsequently water was evaporated to dryness under vacuum.

Diester **1a** was prepared from dicyclopentadiene and dimethyl acetylenedicarboxylate according to the literature.^54,55^ Diesters **2a**, **3a**, and **4a** were purchased from Alfa Aesar.

### Measurement of DLS/ELS

Each of sodium salts of the carboxylates, **1b′–4b′**, and potassium salt **5b′** was dissolved in THF first and 10 times the volume of water was added for the preparation of 250, 100, 50, 20, and 10 mM, and the solution was cooled to 0 °C in an ice-water bath. The final proportion of THF:water was adjusted to 2:28 as in the reaction conditions we reported before,^10^ and 1 mL of the thus prepared sample solution was transferred to a DLS cell for measurement of DLS (analysis mode: Contin) and to an ELS standard cell for measurement of ELS (analysis mode: Smoluchowski) using a PLS-450 (Otsuka Electronics Co. Ltd., Japan) with a measurement angle of 90° at 4 °C. The DLS and ELS analyses of the aqueous solution with the same THF proportion without the analytes were also performed for a blank, and no noticeable data were detected. The measurement was repeated 4–5 times for each sample, and the reproducibility was confirmed.

No unexpected or unusually high safety hazards were encountered.

## Supporting Information Available

DLS and ELS data of **1b′–5b′**, zeta potential and particle diameter of **1b′–5b′**, and DLS/ELS data (PDF)
