# Supplementary material for: Micellar Mechanisms for Desymmetrization Reactions in Aqueous Media
Source: ACS Omega. 2023 Sep 5;8(37):33819–24. doi: 10.1021/acsomega.3c04318 (PMC10515588; doi:10.1021/acsomega.3c04318)

Supporting Information

Micellar mechanisms for desymmetrization reactions in aqueous media

Satomi Niwayama[1,*] and Yoshikazu Hiraga[2]

[1] Graduate School of Engineering, Muroran Institute of Technology, Muroran, Hokkaido, 050-8585, Japan

[2] Department of Food Sciences and Biotechnology, Hiroshima Institute of Technology, Hiroshima, 731-5193, Japan

*Corresponding author. Email: niwayama@mmm.muroran-it.ac.jp.

This file includes:

Tables S1 to S5 (Summary of DLS and ELS data of **1b'-5b'**)
Figures S1 to S5 (Zeta potential and particle diameter of **1b'-5b'**)
DLS data
ELS data

Table **S1**. Summary of ELS and DLS for **1b'**

MonoMethylNorbornaDieneNa (**1b'**)

ELS (zeta potential)

	average	SD
250mmol	−12.96	2.30
100mmol	−15.37	1.82
50mmol	−16.28	1.25
20mmol	−19.23	1.87
10mmol	−20.37	1.24

DLS

	particle diameter	
	average	SD
250mmol	1758.1	332.9
100mmol	282.6	22.5
50mmol	573.7	183.8
20mmol	309.6	28.7
10mmol	337.8	67.9

ELS (zeta potential)

250mmol

1	−11.48
2	−16.16
3	−14.65
4	−11.01
5	−11.51
average	−12.96
SD	2.30

DLS

250mmol

		SD	
1	352.2	176.2	
2	1610.2	430.3	
3	1807.7	486.2	
4	2677.2	1368.3	
5	2343.2	667.2	
average	1758.1	332.9	SD

100mmol

1	−14.08
2	−16.33
3	−12.82
4	−16.87
5	−16.74
average	−15.37
SD	1.82

100mmol

		SD	
1	220.5	33.0	
2	264.6	50.1	
3	307.2	53.1	
4	303.9	52.0	
5	316.8	59.1	
average	282.6	22.5	SD

50mmol

1	−18.25
2	−15.88
3	−14.84
4	−15.94
5	−16.50
average	−16.28
SD	1.25

50mmol

		SD	
1	350.9	64.1	
2	798.1	623.8	
3	709.3	533.5	
4	623.3	401.0	
5	386.8	74.5	
average	573.7	183.8	SD

20mmol

1	−17.83
2	−18.86
3	−17.75
4	−22.34
5	−19.36
average	−19.23
SD	1.87

20mmol

		SD	
1	224.5	32.6	
2	252.3	39.9	
3	365.3	100.0	
4	357.0	59.3	
5	348.7	66.7	
average	309.6	28.7	SD

10mmol

1	−19.97
2	−21.03
3	−22.20
4	−19.39
5	−19.27
average	−20.37
SD	1.24

10mmol

		SD	
1	401.5	219.9	
2	309.6	73.1	
3	393.5	235.1	
4	286.0	55.7	
5	298.3	56.4	
average	337.8	67.9	SD

Table S2. Summary of ELS and DLS for **2b'**

Monomethyl succinate Na (**2b'**)

	ELS (zeta potential)		DLS particle diameter	
	average	SD	average	SD
250mmol	−2.38	0.66	4952.7	4628.7
100mmol	−4.19	0.92	4061.8	2881.1
50mmol	−10.83	3.66	320.6	66.2
20mmol	−10.78	1.93	1324.7	1025.2
10mmol	−15.34	1.11	653.9	475.1

ELS (zeta potential)

250mmol

1	−2.27
2	−2.66
3	−2.33
4	−3.24
5	−1.42
average	−2.38
SD	0.66

100mmol

1	−5.23
2	−4.98
3	−4.06
4	−2.98
5	−3.72
average	−4.19
SD	0.92

50mmol

1	−4.63
2	−13.07
3	−11.66
4	−13.90
5	−10.90
average	−10.83
SD	3.66

20mmol

1	−10.68
2	−8.20
3	−11.44
4	−10.08
5	−13.49
average	−10.78
SD	1.93

10mmol

1	−14.41
2	−16.59
3	−15.27
4	−16.32
5	−14.09
average	−15.34
SD	1.11

DLS

250mmol

		SD	
1	7802.1	15057.5	
2	7626.7	13799.7	
3	1726.8	2629.6	
4	2741.3	4610.0	
5	4866.6	9502.4	
average	4952.7	4628.7	SD

100mmol

		SD	
1	4570.9	6061.4	
2	4507.0	7143.4	
3	3498.1	5741.2	
4	3564.1	6273.8	
5	4168.8	6886.7	
average	4061.8	2881.1	SD

50mmol

		SD	
1	319.7	158.2	
2	344.9	183.3	
3	281.1	58.9	
4	338.8	156.7	
5	318.5	151.3	
average	320.6	66.2	SD

20mmol

		SD	
1	353.2	56.9	
2	1101.2	1416.7	
3	1708.7	2742.6	
4	1403.1	1969.3	
5	2057.1	3586.9	
average	1324.7	1025.2	SD

10mmol

		SD	
1	1304.1	2148.0	
2	801.5	918.4	
3	339.2	187.1	
4	397.6	283.4	
5	426.9	266.7	
average	653.9	475.1	SD

Table S3. Summary of ELS and DLS for **3b'**

Monomethyl Maleate Na (**3b'**)

	average	SD
250mmol	-3.96	2.44
100mmol	-7.68	1.47
50mmol	-11.30	1.25
20mmol	-5.26	2.58
10mmol	-6.09	1.60

DLS

	particle diameter	
	average	SD
250mmol	452.8	450.7
100mmol	504.6	266.9
50mmol	1147.5	894.9
20mmol	2075.4	1595.5
10mmol	5214.6	3351.5

ELS (zeta potential)

250mmol

1	-2.68
2	-6.89
3	-6.08
4	-3.06
5	-1.08
average	-3.96
SD	2.44

DLS

250mmol

		SD	
1	227.8	97.4	
2	314.5	212.9	
3	258.0	111.8	
4	1228.8	2235.8	
5	234.9	115.2	
average	452.8	450.7	SD

100mmol

1	-9.19
2	-5.56
3	-8.46
4	-6.81
5	-8.39
average	-7.68
SD	1.47

100mmol

		SD	
1	923.2	1198.7	
2	352.5	216.4	
3	440.5	325.0	
4	433.0	363.7	
5	374.0	244.3	
average	504.6	266.9	SD

50mmol

1	-15.59
2	-9.18
3	-12.17
4	-10.07
5	-9.50
average	-11.30
SD	2.66

50mmol

		SD	
1	985.1	1081.2	
2	2122.5	3430.1	
3	589.6	465.9	
4	1664.4	2613.1	
5	375.9	202.9	
average	1147.5	894.9	SD

20mmol

1	-2.02
2	-8.06
3	-7.26
4	-5.73
5	-3.22
average	-5.26
SD	2.58

20mmol

		SD	
1	1602.3	2410.7	
2	324.9	219.9	
3	2079.6	3336.7	
4	4696.0	6292.2	
5	1674.0	2656.0	
average	2075.4	1595.5	SD

10mmol

1	-5.99
2	-3.38
3	-7.01
4	-7.36
5	-6.71
average	-6.09
SD	1.60

10mmol

		SD	
1	7455.0	3485.6	
2	1532.9	2576.2	
3	4872.6	7342.1	
4	7180.1	11759.1	
5	5032.6	8357.1	
average	5214.6	3351.5	SD

Table S4. Summary of ELS and DLS for **4b'**

Monoethyl Phenylmalonate Na (**4b'**)

	ELS (zeta potential) average	ELS SD	DLS particle diameter average	DLS SD
250mmol	−18.66	4.19	3574.3	812.4
100mmol	−8.34	1.06	584.8	44.0
50mmol	−12.23	1.46	515.5	162.2
20mmol	−15.00	2.29	233.1	34.8
10mmol	−16.05	1.45	231.7	35.1

ELS (zeta potential)

250mmol

1	−18.76
2	−13.12
3	−24.43
4	−16.77
5	−20.24
average	−18.66
SD	4.19

100mmol

1	−7.50
2	−8.85
3	−9.75
4	−7.10
5	−8.52
average	−8.34
SD	1.06

50mmol

1	−10.45
2	−10.89
3	−13.55
4	−13.39
5	−12.88
average	−12.23
SD	1.46

20mmol

1	−13.42
2	−14.08
3	−14.11
4	−19.06
5	−14.35
average	−15.00
SD	2.29

10mmol

1	−17.95
2	−14.56
3	−16.21
4	−14.67
5	−16.85
average	−16.05
SD	1.45

DLS

250mmol

		SD	
1	3024.3	1386.9	
2	3725.6	1885.4	
3	3957.0	2013.7	
4	4255.4	2278.0	
5	2909.1	1332.6	
average	3574.3	812.4	SD

100mmol

		SD	
1	580.9	88.8	
2	569.6	84.0	
3	747.6	140.9	
4	532.7	79.1	
5	493.0	85.7	
average	584.8	44.0	SD

50mmol

		SD	
1	337.6	96.3	
2	531.9	316.9	
3	647.5	531.8	
4	577.2	427.7	
5	483.5	286.2	
average	515.5	162.2	SD

20mmol

		SD	
1	167.0	31.6	
2	212.9	36.5	
3	223.0	35.9	
4	246.0	48.9	
5	316.4	155.6	
average	233.1	34.8	SD

10mmol

		SD	
1	219.5	32.7	
2	283.1	148.9	
3	213.8	47.6	
4	213.7	50.0	
5	228.4	52.4	
average	231.7	35.1	SD

Table S5. Summary of ELS and DLS for **5b'**

Monomethyl malonate K (**5b'**)

	ELS (zeta potential) average	SD		DLS particle diameter average	SD
250mmol	−7.53	1.48	250mmol	6305.72	4471.33
100mmol	−4.29	3.47	100mmol	4181.44	3634.37
50mmol	−1.11	0.68	50mmol	1375.50	1089.59
20mmol	−6.88	2.04	20mmol	7808.04	4471.56
10mmol	−5.21	2.38	10mmol	800.15	837.62

ELS (zeta potential)

250mmol

1	−6.20
2	−8.11
3	−6.81
4	−9.85
5	−6.68
average	−7.53
SD	1.48

100mmol

1	−1.11
2	−7.54
3	−3.81
4	−8.17
5	−0.80
average	−4.29
SD	3.47

50mmol	
1	−1.42
2	−1.67
3	−0.98
4	−1.51
5	0.02
average	−1.11
SD	0.68

20mmol	
1	−6.36
2	−5.17
3	−6.68
4	−10.38
5	−5.82
average	−6.88
SD	2.04

10mmol

1	−6.64
2	−3.20
3	−2.73
4	−8.42
5	−5.05
average	−5.21
SD	2.38

DLS

250mmol		SD	
1	4370.2	7680.8	
2	3413.8	5921.5	
3	6115.6	8106.2	
4	7207.3	10630.6	
5	10421.7	15067.9	
average	6305.7	4471.3	SD

100mmol		SD	
1	3975.8	7848.9	
2	5335.2	9750.3	
3	5831.0	11232.9	
4	3298.6	5614.2	
5	2466.6	3980.6	
average	4181.4	3634.4	SD

50mmol		SD	
1	1305.1	2876.7	
2	390.6	277.0	
3	2598.4	3931.0	
4	1481.3	2366.6	
5	1102.1	523.7	
average	1375.5	1089.6	SD

20mmol		SD	
1	9229.2	12109.0	
2	6670.3	9176.2	
3	6837.1	8079.4	
4	5813.2	7335.5	
5	10490.4	12245.6	
average	7808.0	4471.6	SD

10mmol		SD	
1	239.5	309.4	
2	1281.5	1895.9	
3	1222.4	2587.8	
4	457.2	915.9	
5	ND		
average	800.2	837.6	SD

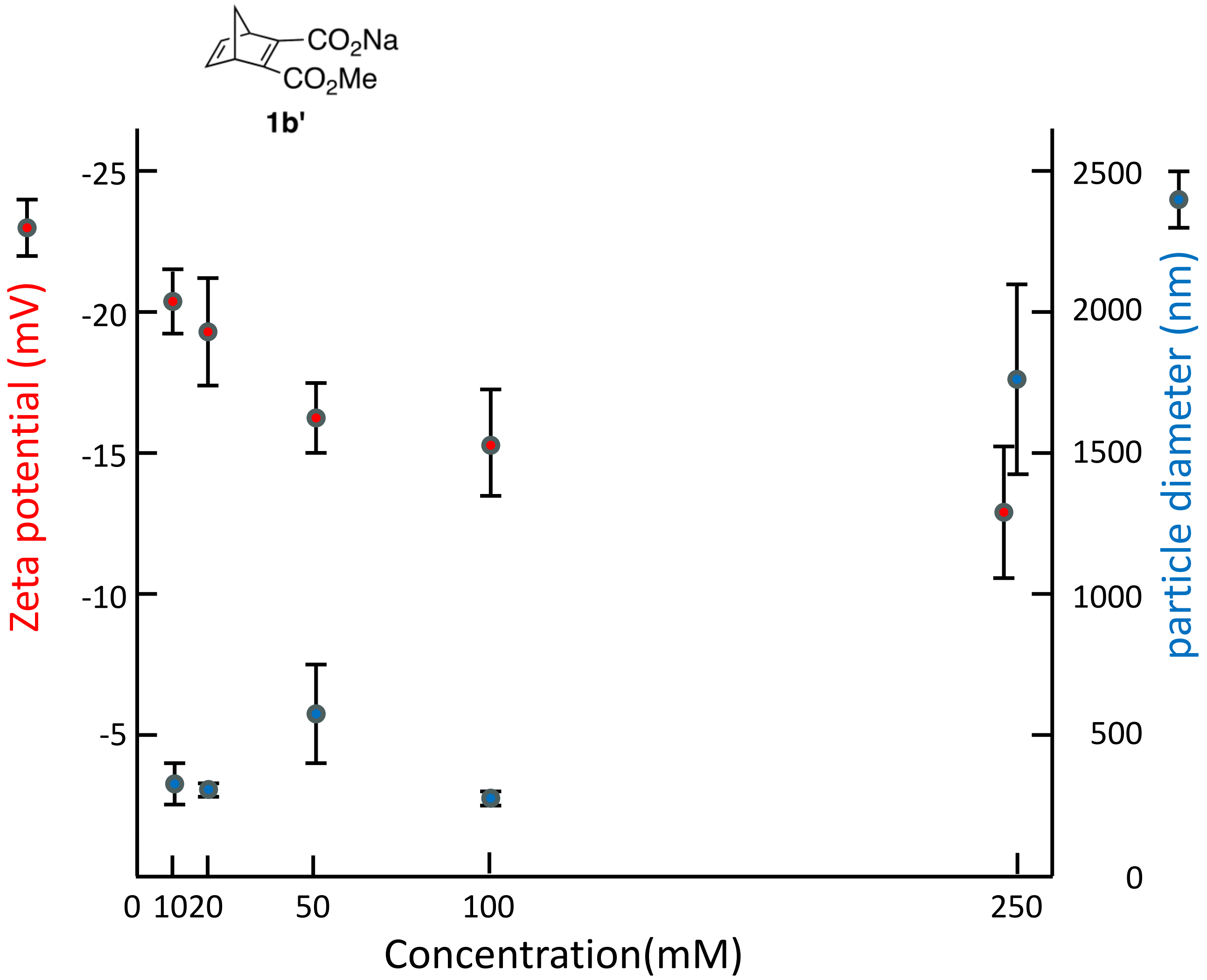

Figure S1. Zata potential and particle diameter of monomethyl norbornadiene Na (**1b'**) in aqueous media at 4 °C

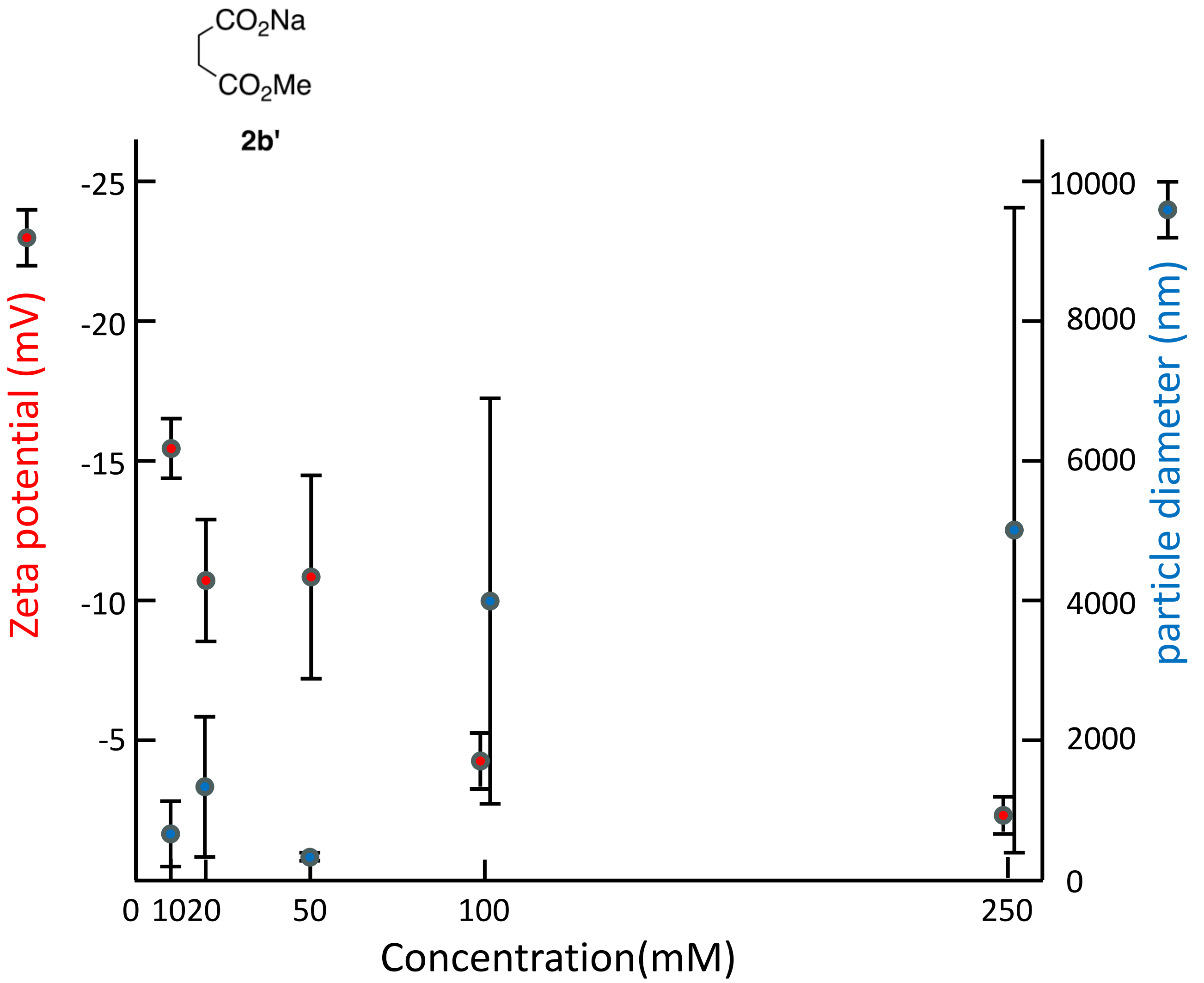

Figure S2. Zata potential and particle diameter of monomethyl succinate Na (**2b'**) in aqueous media at 4 °C

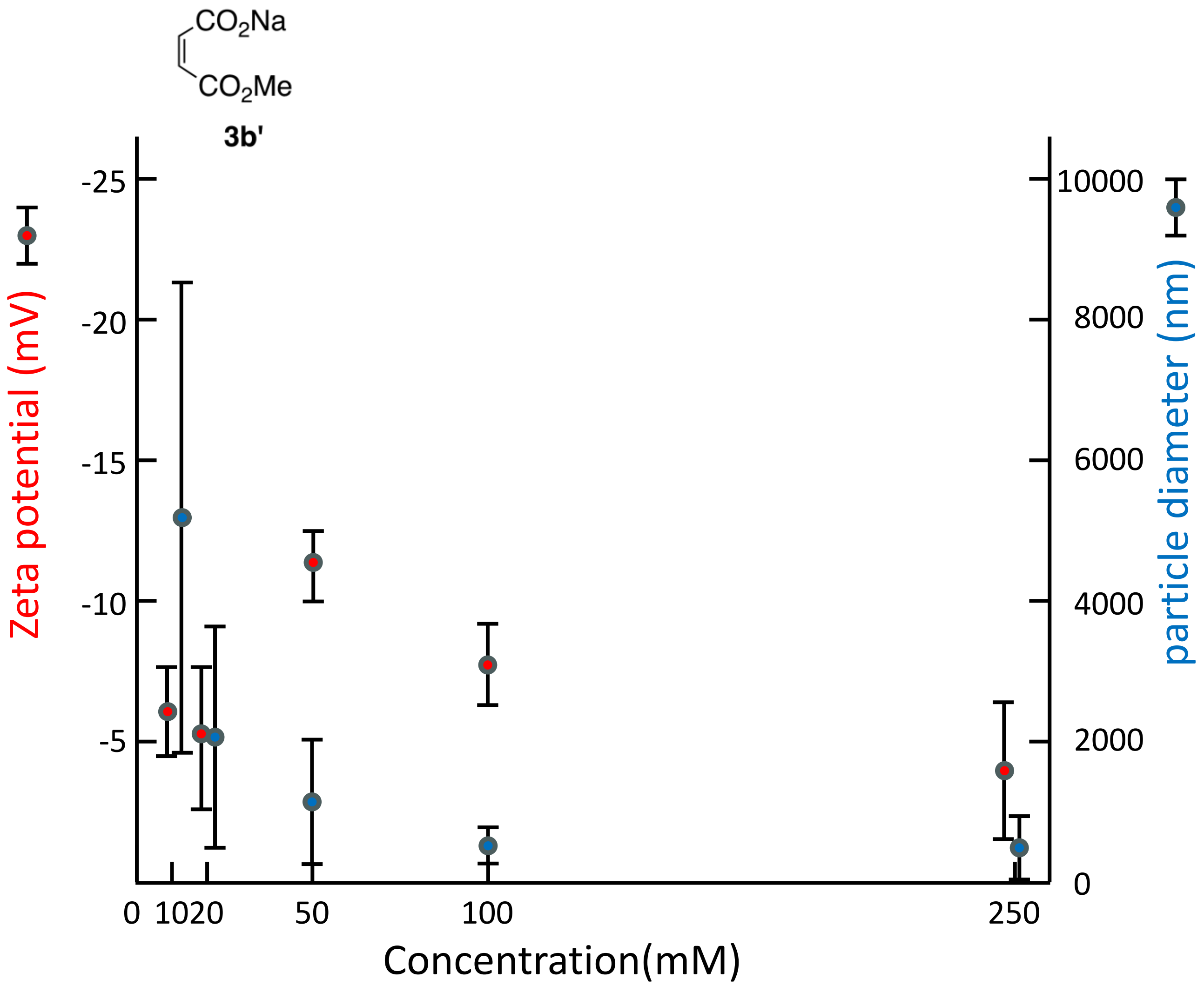

Figure S3. Zata potential and particle diameter of monomethyl maleate Na (**3b'**) in aqueous media at 4 ^{o}C

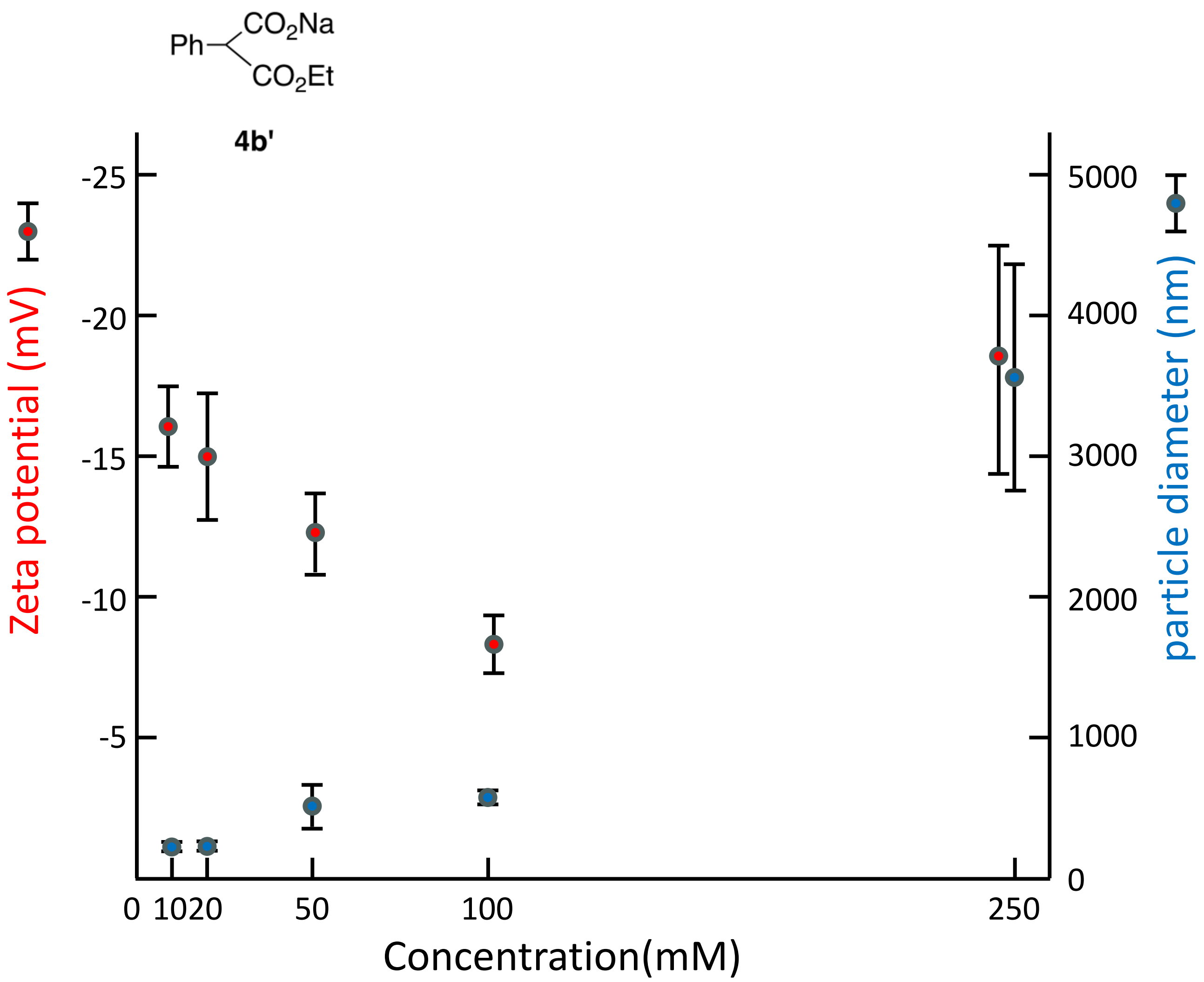

Figure S4. Zata potential and particle diameter of monoethyl-phenyl malonate Na (**4b'**) in aqueous media at 4 °C

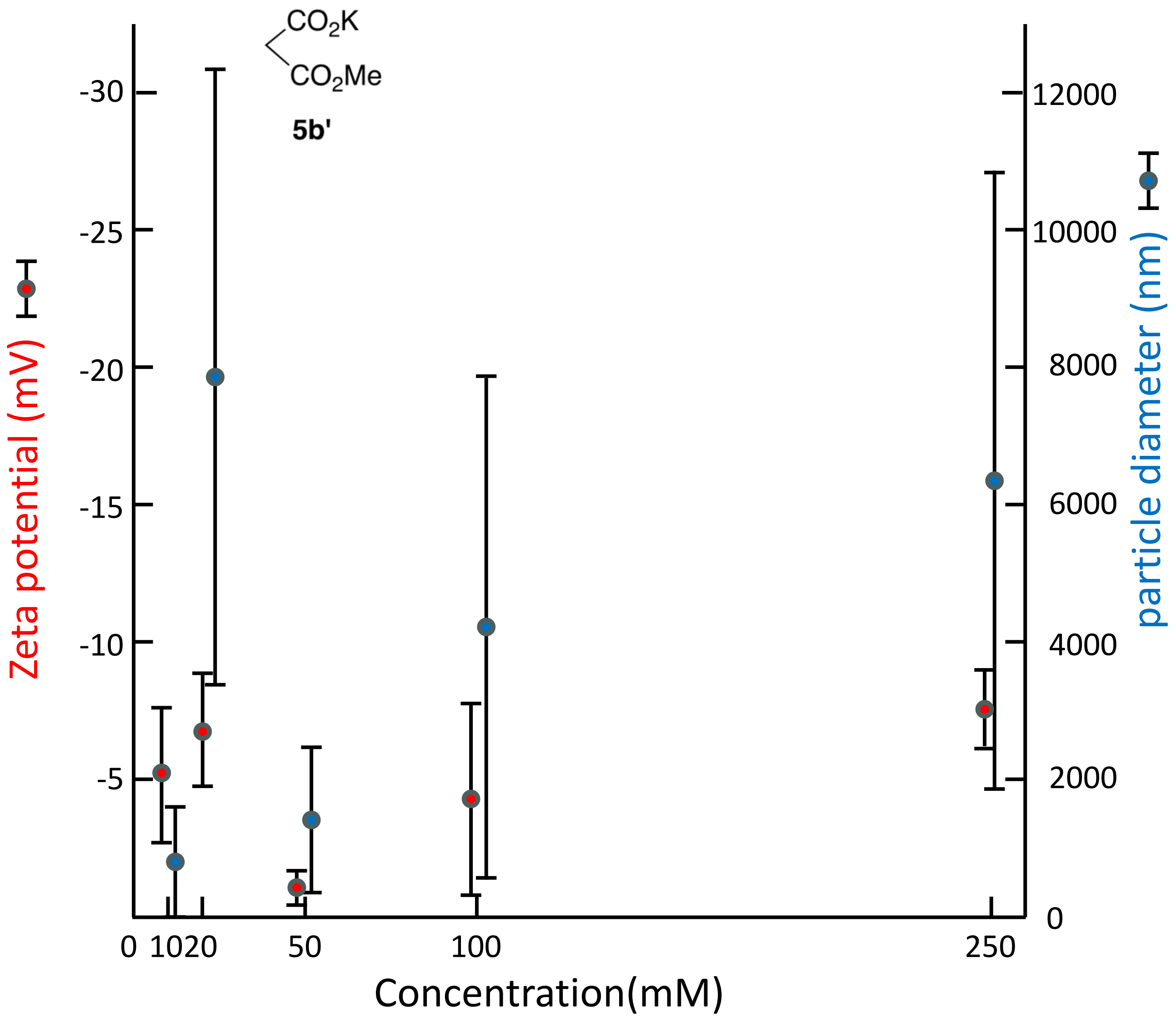

Figure S5. Zata potential and particle diameter of monomethyl malonate K (**5b'**) in aqueous media at 4 ^{o}C

Scattering distribution of monomethyl norbornadiene Na (**1b'**) 250 mM

Data #1

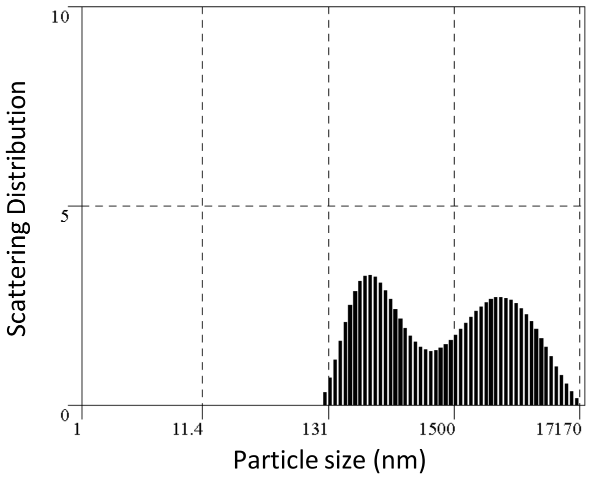

Histogram Analysis Results

Peak	Average (nm)	Standard Deviation
1	352.3	± 176.2
2	4097.4	± 2858.6

Data #2

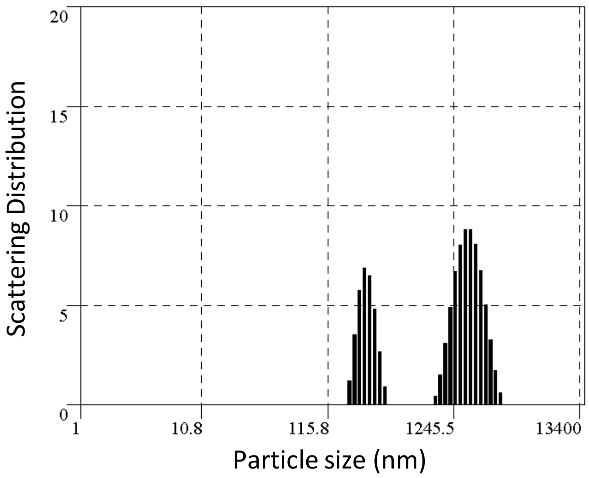

Histogram Analysis Results

Peak	Average (nm)	Standard Deviation
1	226.4	± 36.7
2	1610.2	± 430.3

Data #3

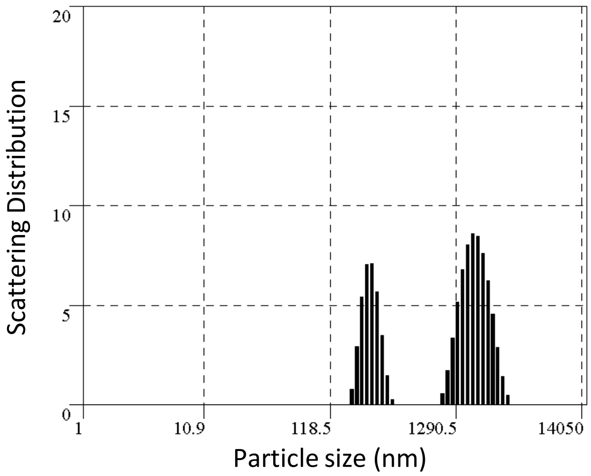

Histogram Analysis Results

Peak	Average (nm)	Standard Deviation
1	239.7	± 40.0
2	1807.7	± 486.2

Data #4

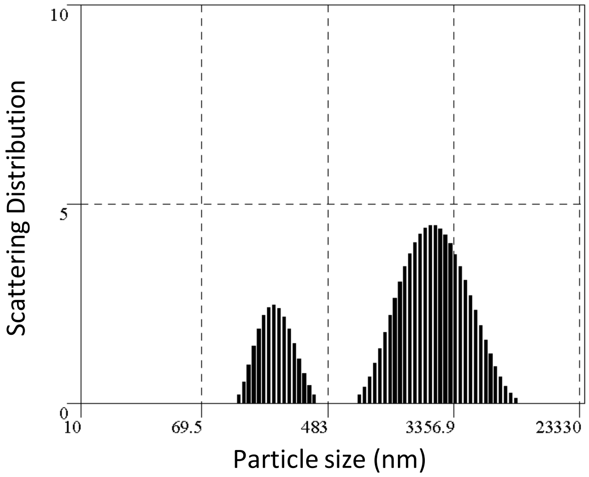

Histogram Analysis Results

Peak	Average (nm)	Standard Deviation
1	207.0	± 54.6
2	2677.2	± 1368.3

Data #5

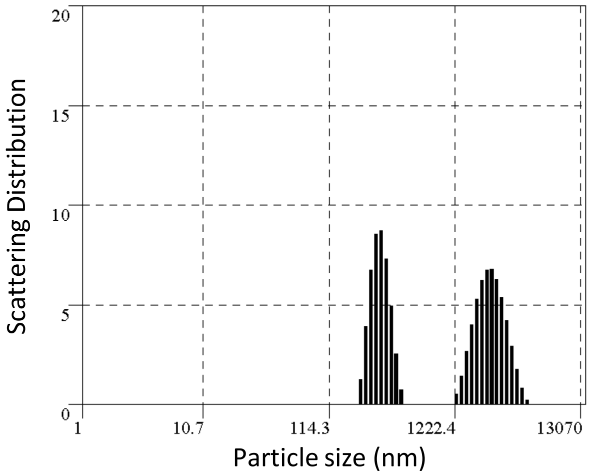

Histogram Analysis Results

Peak	Average (nm)	Standard Deviation
1	281.6	± 49.9
2	2343.2	± 667.2

Scattering distribution of monomethyl norbornadiene Na (**1b'**) 100 mM

Data #1

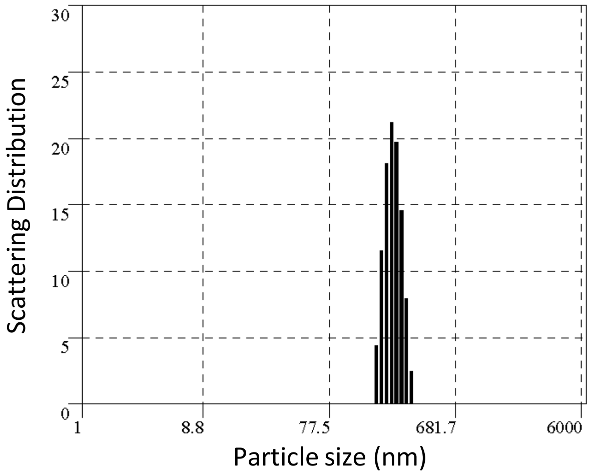

Histogram Analysis Results

Peak	Average (nm)	Standard Deviation
1	220.5	± 33.0

Data #2

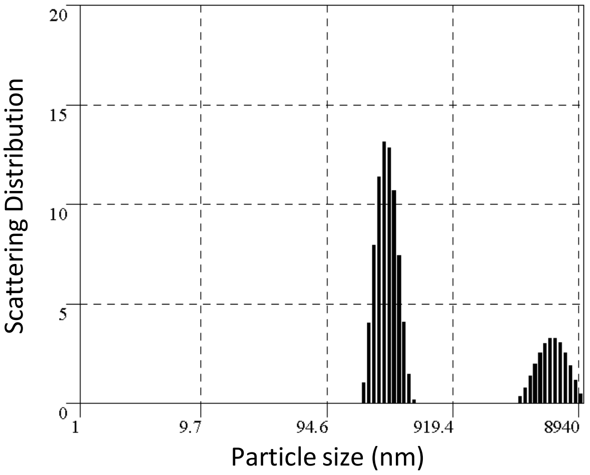

Histogram Analysis Results

Peak	Average (nm)	Standard Deviation
1	264.6	± 50.1
2	5462.1	± 1378.9

Data #3

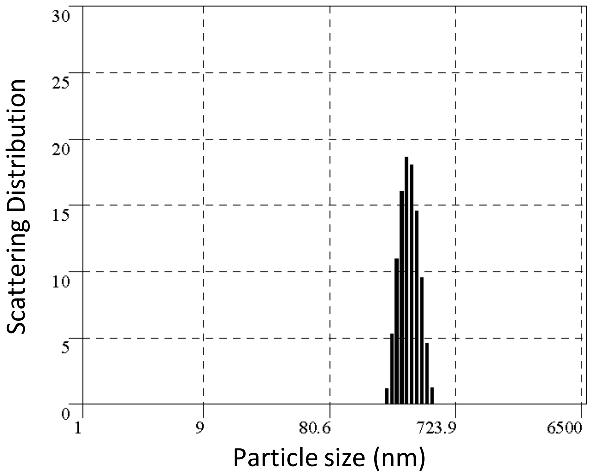

Histogram Analysis Results

Peak	Average (nm)	Standard Deviation
1	307.2	± 53.1

Data #4

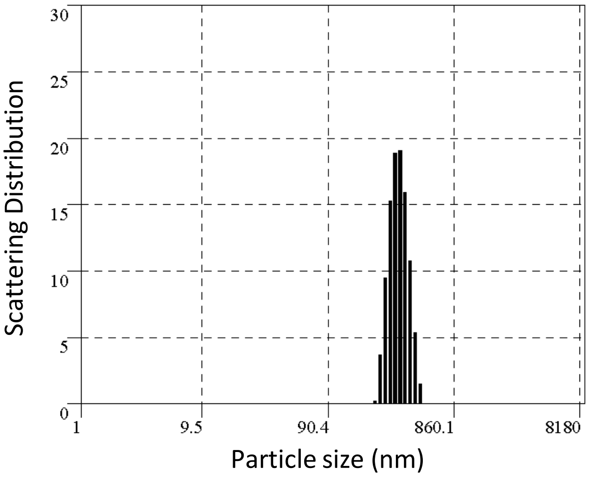

Histogram Analysis Results

Peak	Average (nm)	Standard Deviation
1	303.9	± 52.0

Data #5

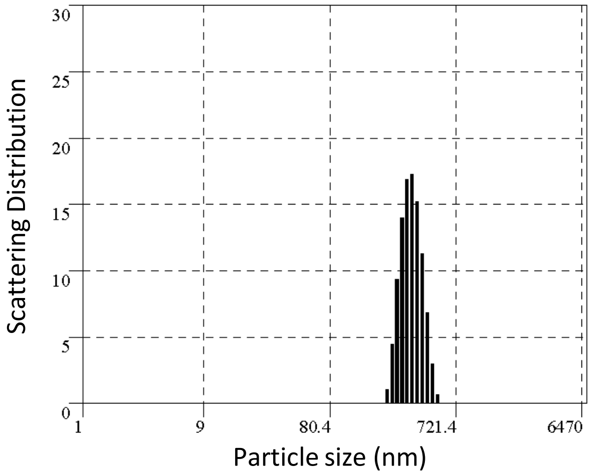

Histogram Analysis Results

Peak	Average (nm)	Standard Deviation
1	316.8	± 59.1

Scattering distribution of monomethyl norbornadiene Na (**1b'**) 50 mM

Data #1

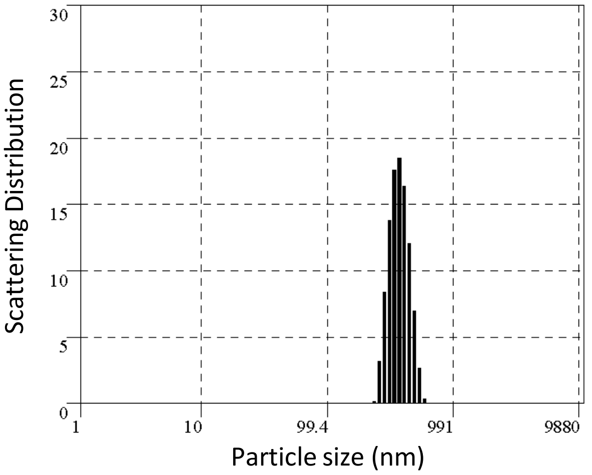

Histogram Analysis Results

Peak	Average (nm)	Standard Deviation
1	350.9	± 64.1

Data #2

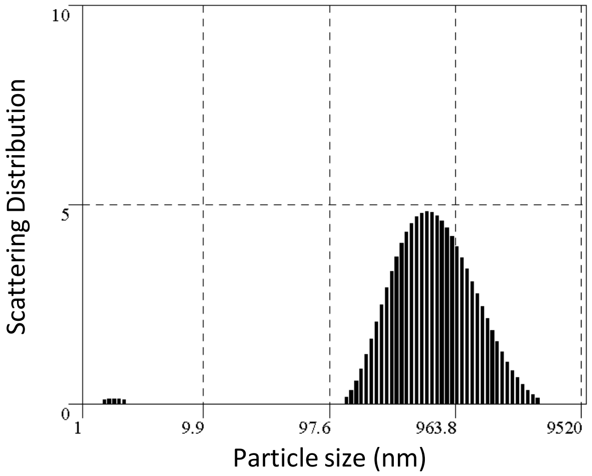

Histogram Analysis Results

Peak	Average (nm)	Standard Deviation
1	1.8	± 0.2
2	798.1	± 623.8

Data #3

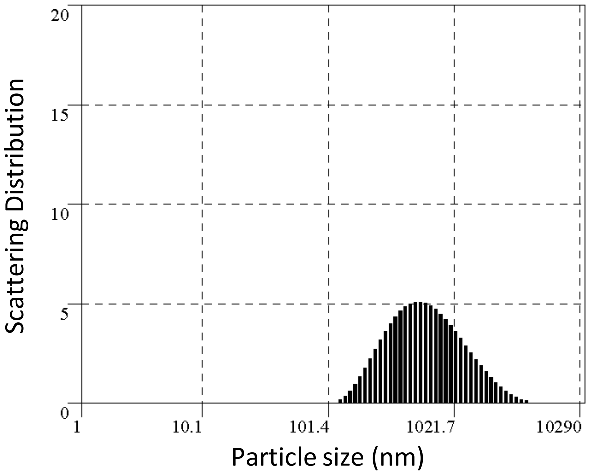

Histogram Analysis Results

Peak	Average (nm)	Standard Deviation
1	709.3	± 533.5

Data #4

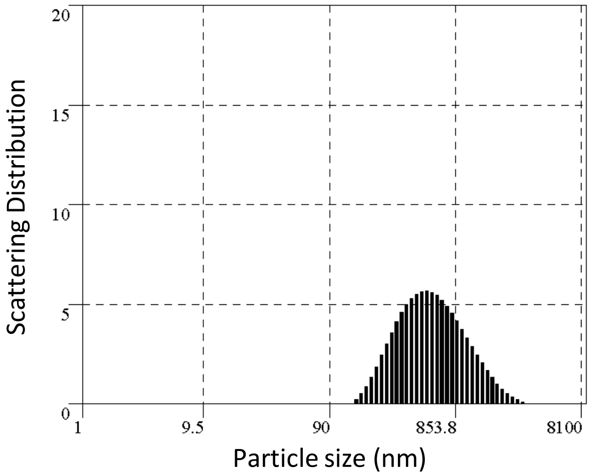

Histogram Analysis Results

Peak	Average (nm)	Standard Deviation
1	623.3	± 401.0

Data #5

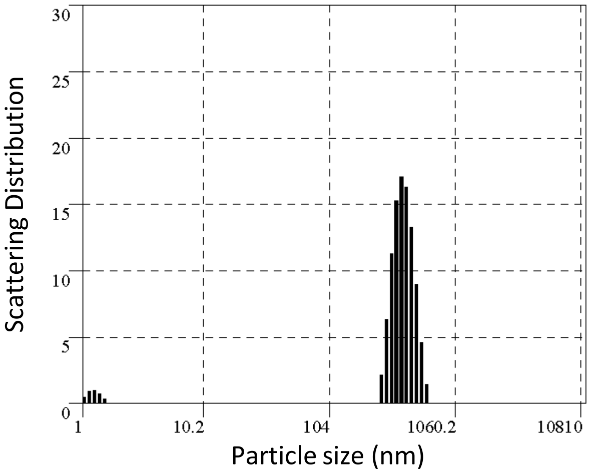

Histogram Analysis Results

Peak	Average (nm)	Standard Deviation
1	1.2	± 0.1
2	386.8	± 74.5

Scattering distribution of monomethyl norbornadiene Na (**1b'**) 20 mM

Data #1

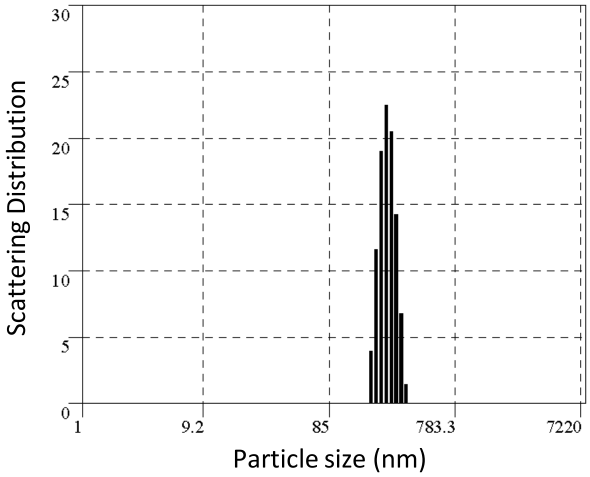

Histogram Analysis Results

Peak	Average (nm)	Standard Deviation
1	224.5	± 32.6

Data #2

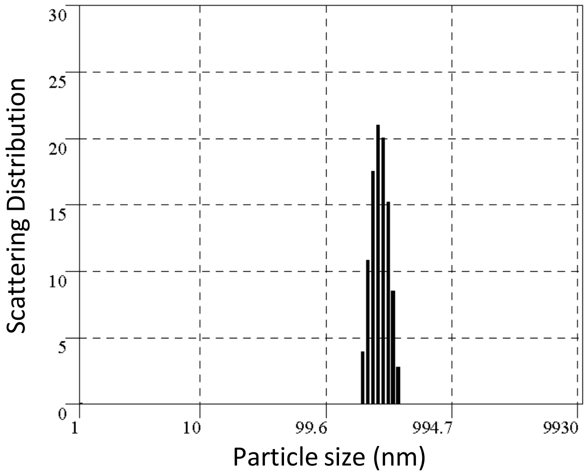

Histogram Analysis Results

Peak	Average (nm)	Standard Deviation
1	252.3	± 39.9

Data #3

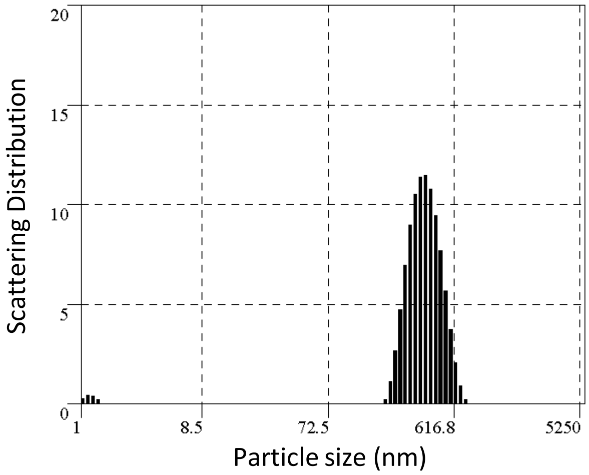

Histogram Analysis Results

Peak	Average (nm)	Standard Deviation
1	1.1	± 0.1
2	365.3	± 100.0

Data #4

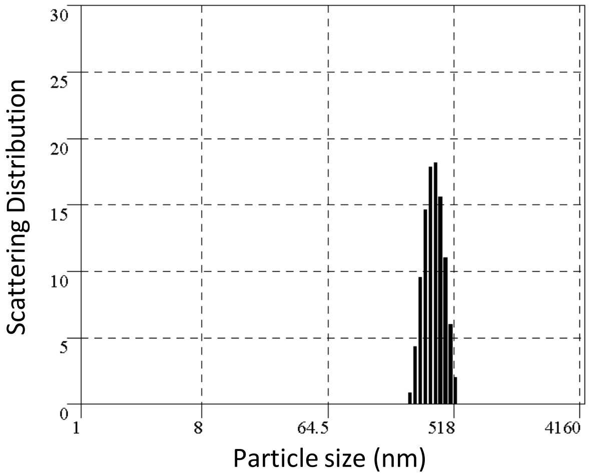

Histogram Analysis Results

Peak	Average (nm)	Standard Deviation
1	357.0	± 59.3

Data #5

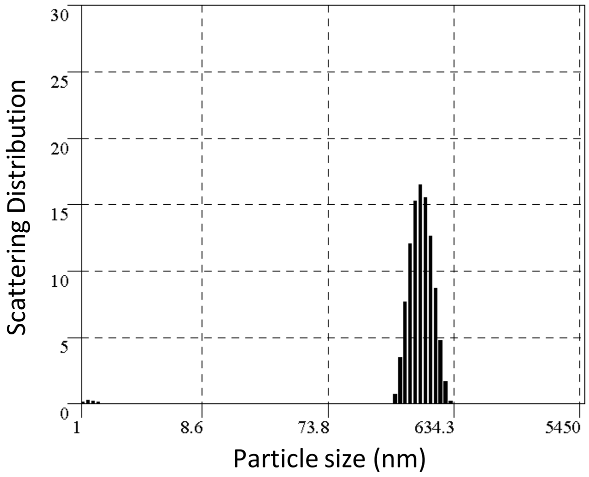

Histogram Analysis Results

Peak	Average (nm)	Standard Deviation
1	1.1	± 0.1
2	348.7	± 66.7

Scattering distribution of monomethyl norbornadiene Na (**1b'**) 10 mM

Data #1

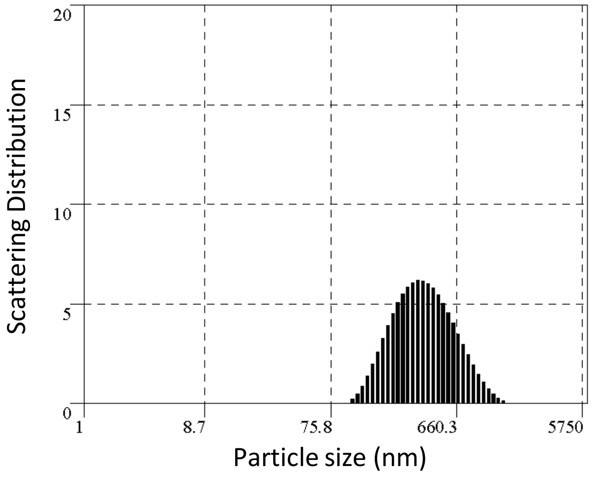

Histogram Analysis Results

Peak	Average (nm)	Standard Deviation
1	401.5	± 219.9

Data #2

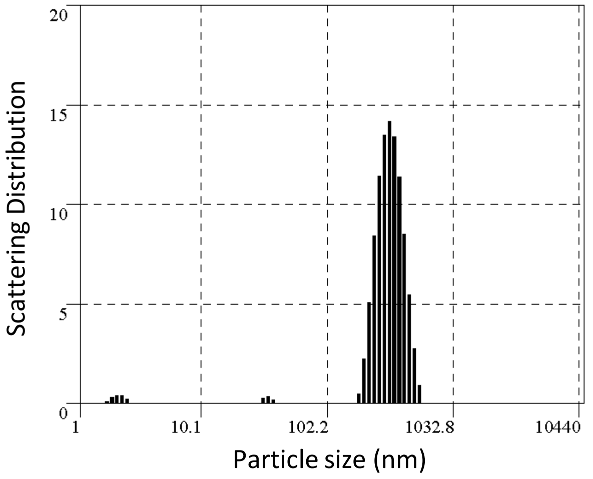

Histogram Analysis Results

Peak	Average (nm)	Standard Deviation
1	2.0	± 0.2
2	31.5	± 2.2
3	309.6	± 73.1

Data #3

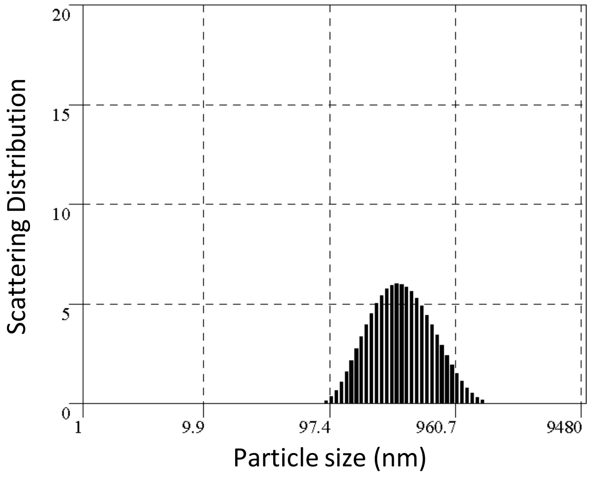

Histogram Analysis Results

Peak	Average (nm)	Standard Deviation
1	393.5	± 235.1

Data #4

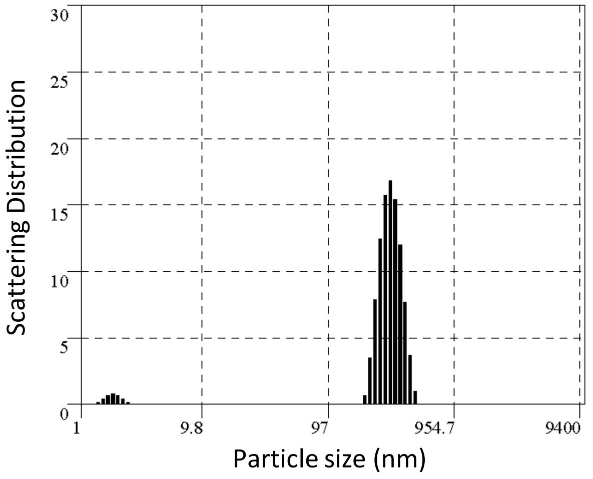

Histogram Analysis Results

Peak	Average (nm)	Standard Deviation
1	1.8	± 0.2
2	286.0	± 55.7

Data #5

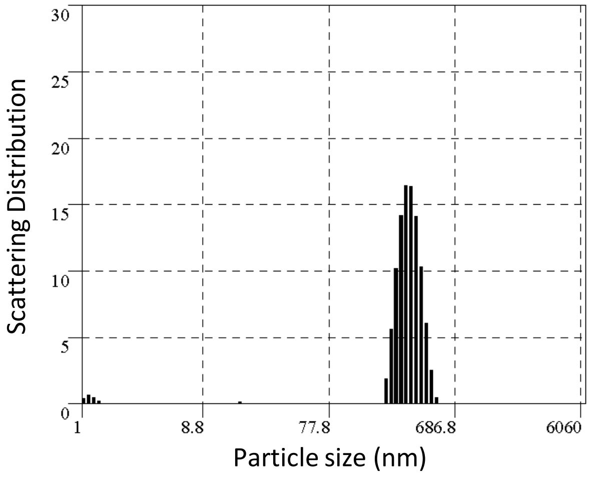

Histogram Analysis Results

Peak	Average (nm)	Standard Deviation
1	1.1	± 0.1
2	15.3	± 0.0
3	298.3	± 56.4

Scattering distribution of monomethyl succinate Na (**2b'**) 250 mM

Data #1

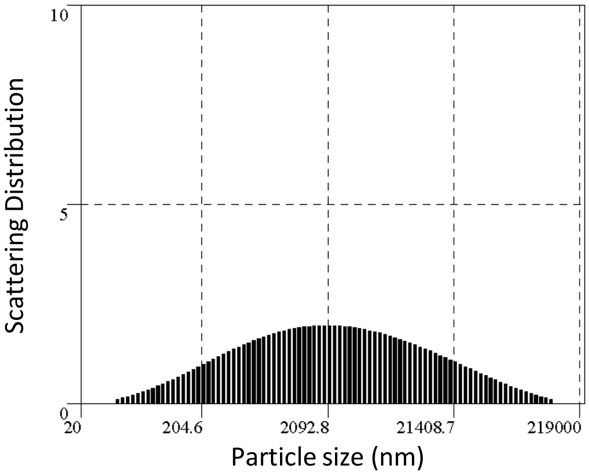

Histogram Analysis Results

Peak	Average (nm)	Standard Deviation
1	7802.1	± 15057.5

Data #2

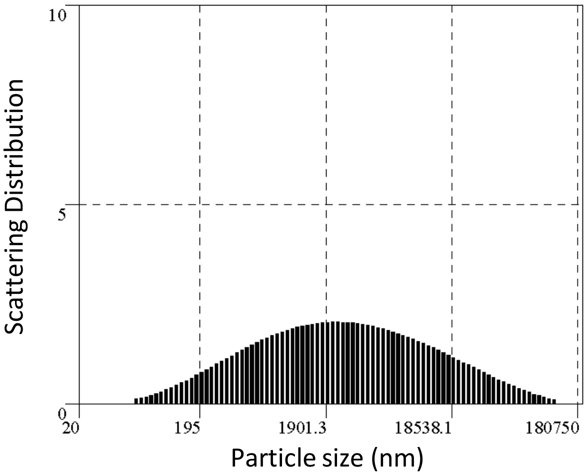

Histogram Analysis Results

Peak	Average (nm)	Standard Deviation
1	7626.7	± 13799.7

Data #3

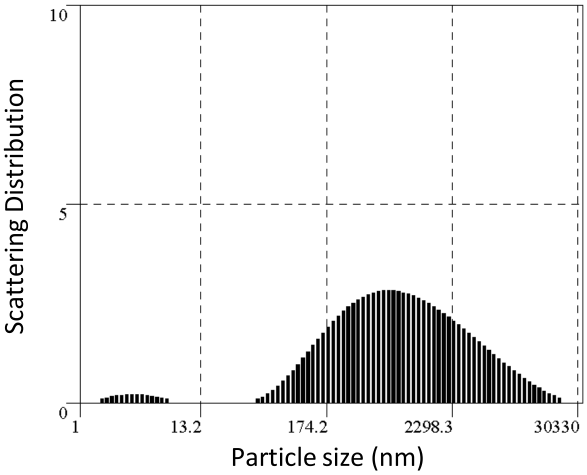

Histogram Analysis Results

Peak	Average (nm)	Standard Deviation
1	3.2	± 1.2
2	1726.8	± 2629.6

Data #4

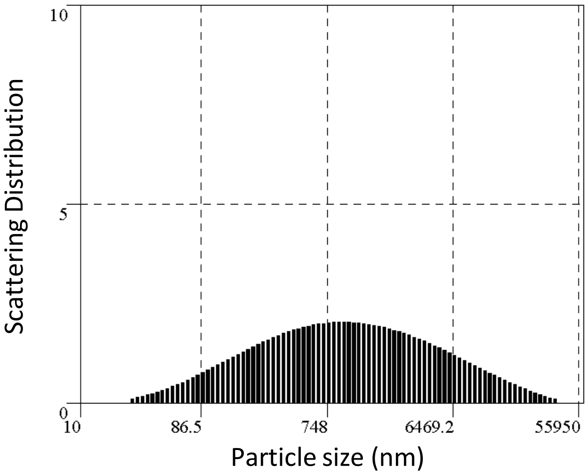

Histogram Analysis Results

Peak	Average (nm)	Standard Deviation
1	2741.3	± 4610.0

Data #5

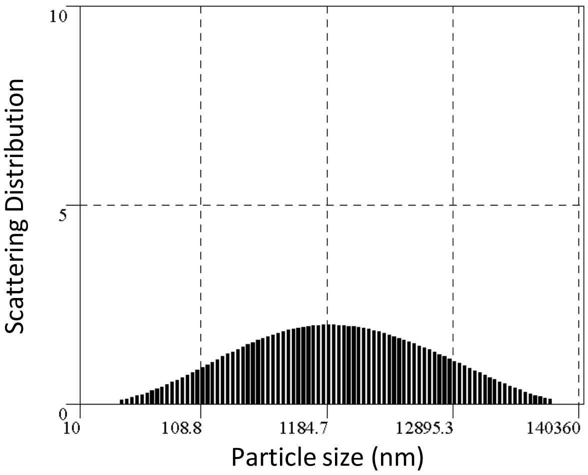

Histogram Analysis Results

Peak	Average (nm)	Standard Deviation
1	4866.6	± 9502.4

Scattering distribution of monomethyl succinate Na (**2b'**) 100 mM

Data #1

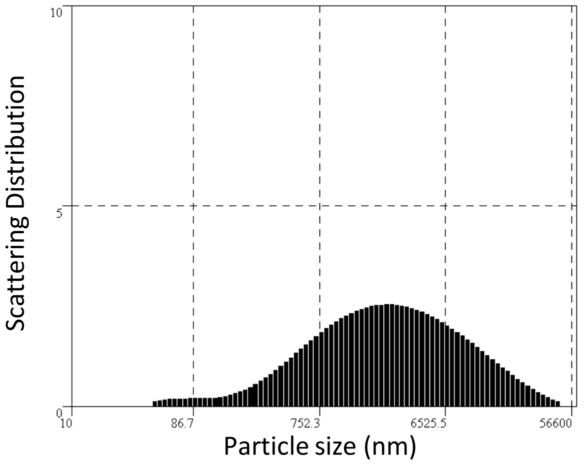

Histogram Analysis Results

Peak	Average (nm)	Standard Deviation
1	4570.9	± 6061.4

Data #2

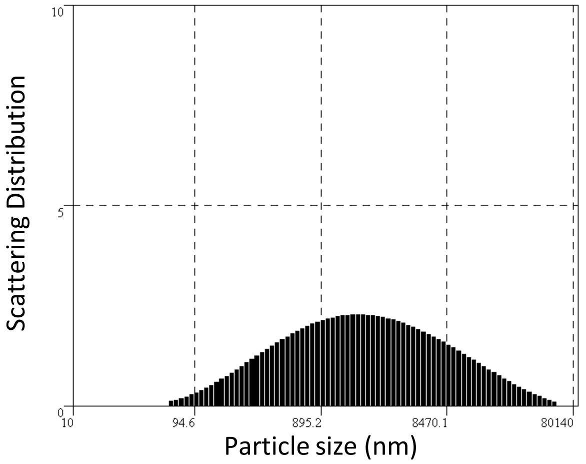

Histogram Analysis Results

Peak	Average (nm)	Standard Deviation
1	4507.0	± 7143.4

Data #3

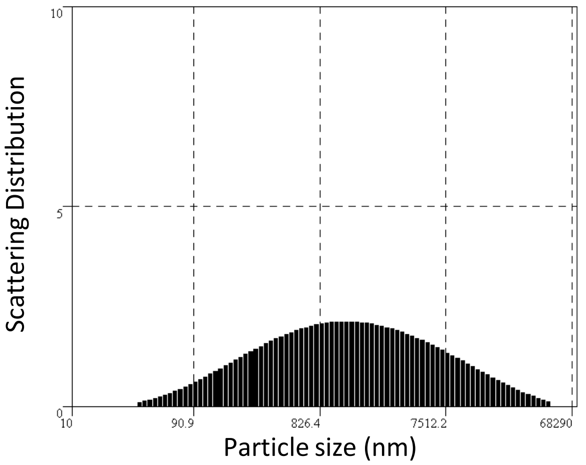

Histogram Analysis Results

Peak	Average (nm)	Standard Deviation
1	3498.1	± 5741.2

Data #4

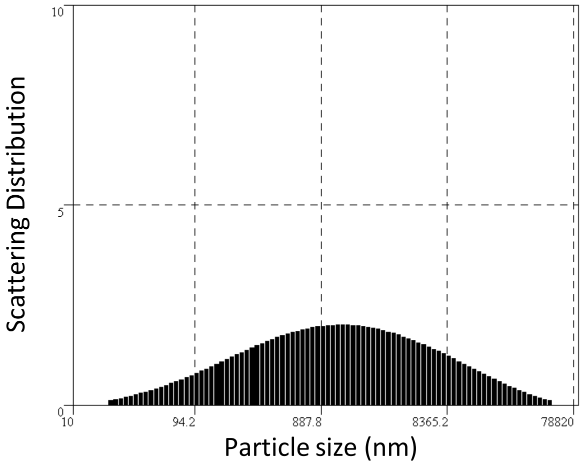

Histogram Analysis Results

Peak	Average (nm)	Standard Deviation
1	3564.1	± 6273.8

Data #5

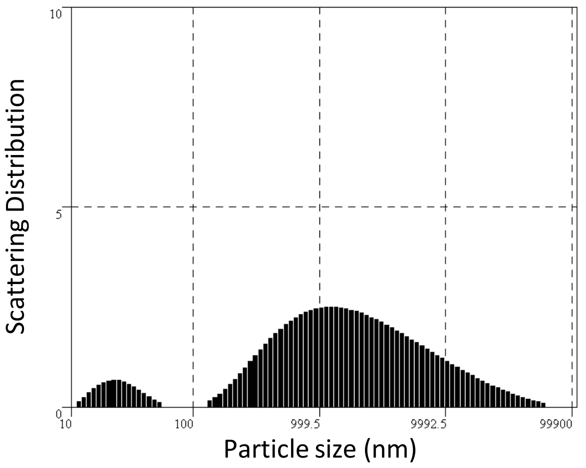

Histogram Analysis Results

Peak	Average (nm)	Standard Deviation
1	23.8	± 8.8
2	4168.8	± 6886.7

Scattering distribution of monomethyl succinate Na (**2b'**) 50 mM

Data #1

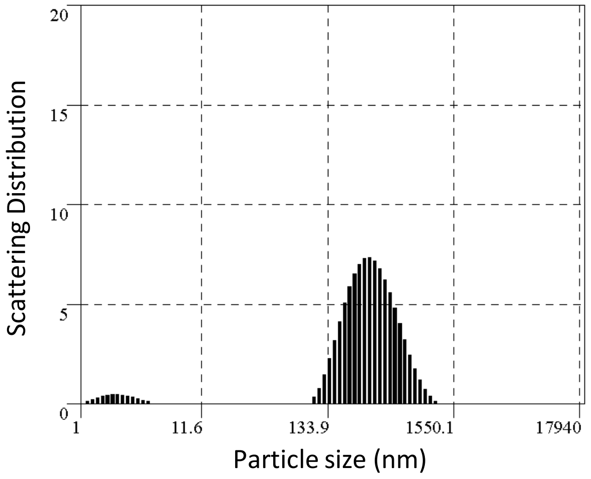

Histogram Analysis Results

Peak	Average (nm)	Standard Deviation
1	2.1	± 0.6
2	319.7	± 158.2

Data #2

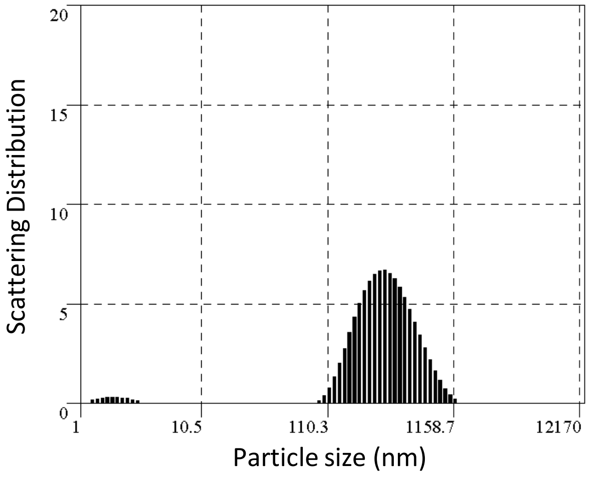

Histogram Analysis Results

Peak	Average (nm)	Standard Deviation
1	1.9	± 0.5
2	344.9	± 183.3

Data #3

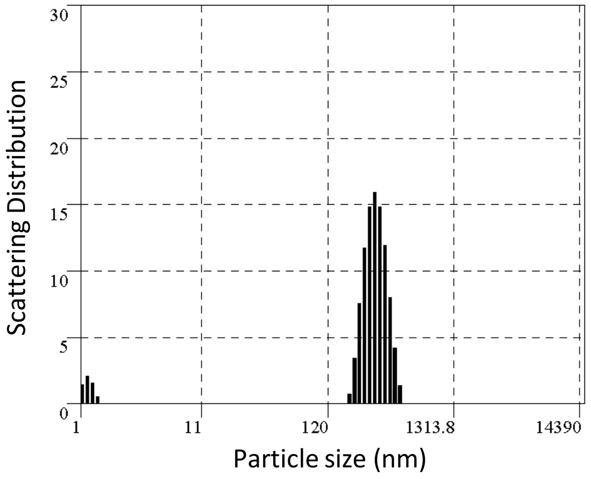

Histogram Analysis Results

Peak	Average (nm)	Standard Deviation
1	1.1	± 0.1
2	281.1	± 58.9

Data #4

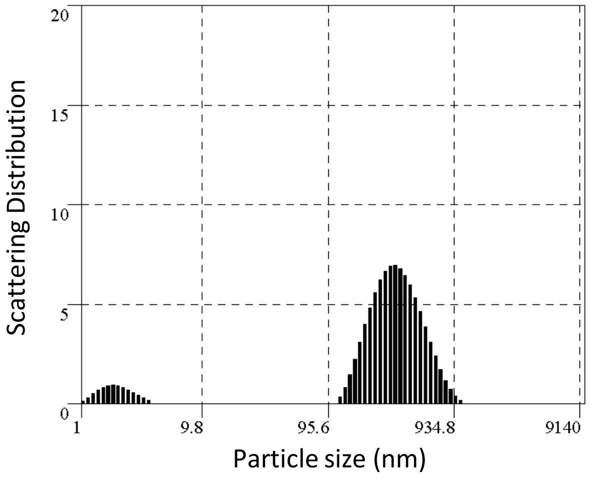

Histogram Analysis Results

Peak	Average (nm)	Standard Deviation
1	1.9	± 0.5
2	338.8	± 156.7

Data #5

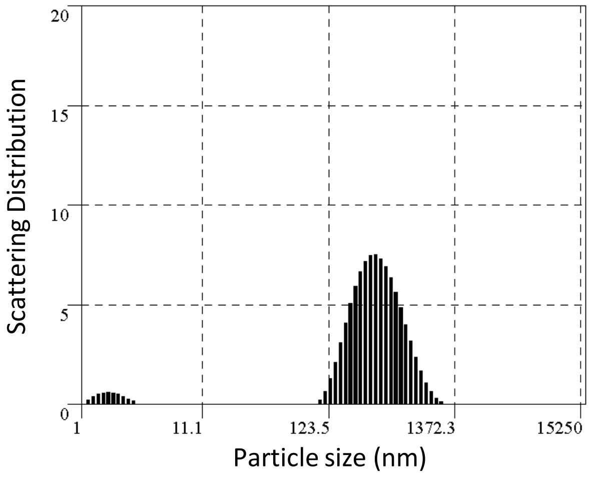

Histogram Analysis Results

Peak	Average (nm)	Standard Deviation
1	1.7	± 0.4
2	318.5	± 151.3

Scattering distribution of monomethyl succinate Na (**2b'**) 20 mM

Data #1

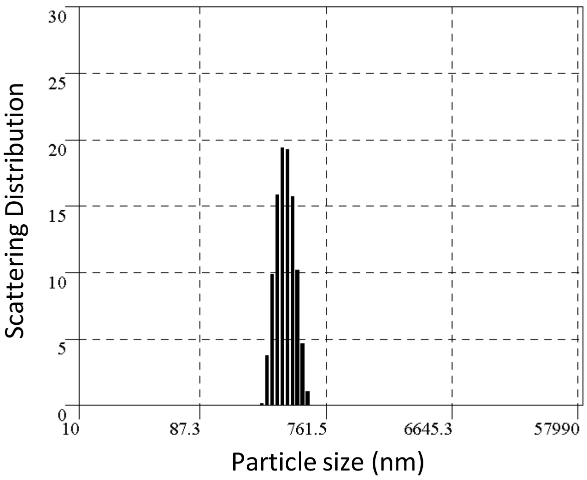

Histogram Analysis Results

Peak	Average (nm)	Standard Deviation
1	353.2	± 56.9

Data #2

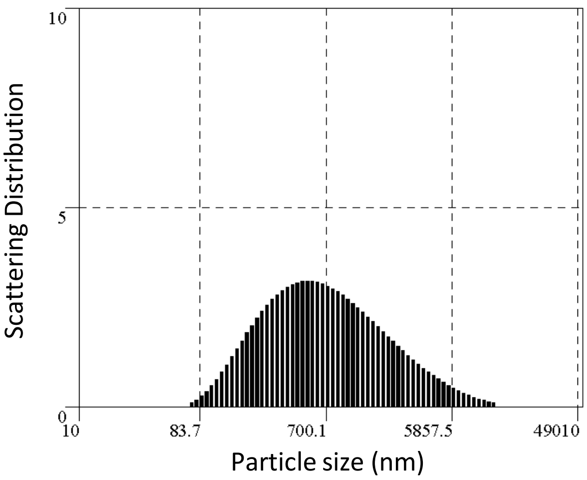

Histogram Analysis Results

Peak	Average (nm)	Standard Deviation
1	1101.2	± 1416.7

Data #3

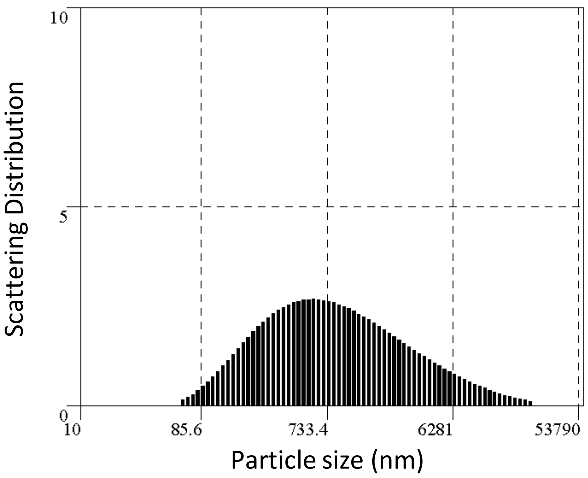

Histogram Analysis Results

Peak	Average (nm)	Standard Deviation
1	1708.7	± 2742.6

Data #4

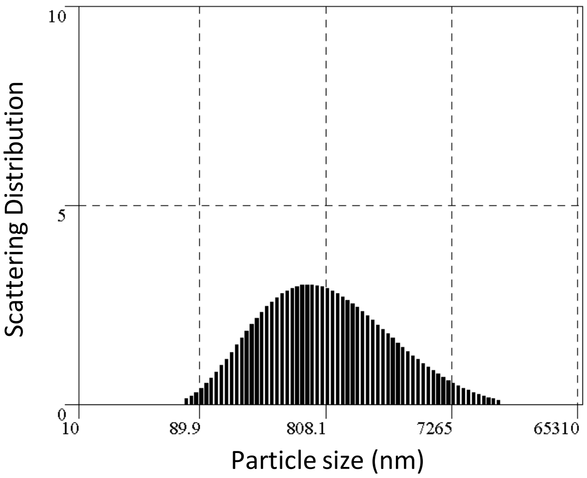

Histogram Analysis Results

Peak	Average (nm)	Standard Deviation
1	1403.1	± 1969.3

Data #5

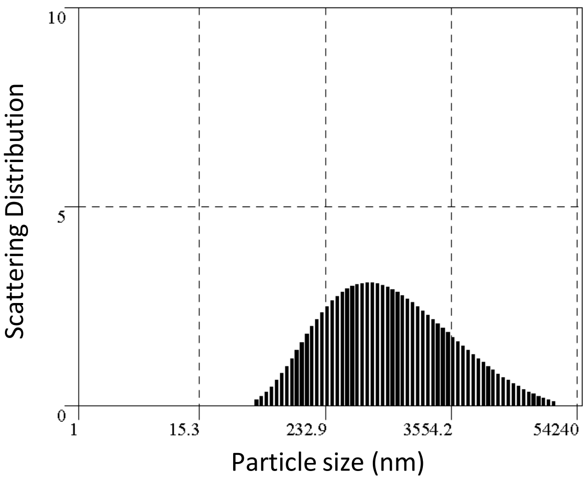

Histogram Analysis Results

Peak	Average (nm)	Standard Deviation
1	2057.1	± 3586.9

Scattering distribution of monomethyl succinate Na (**2b'**) 10 mM

Data #1

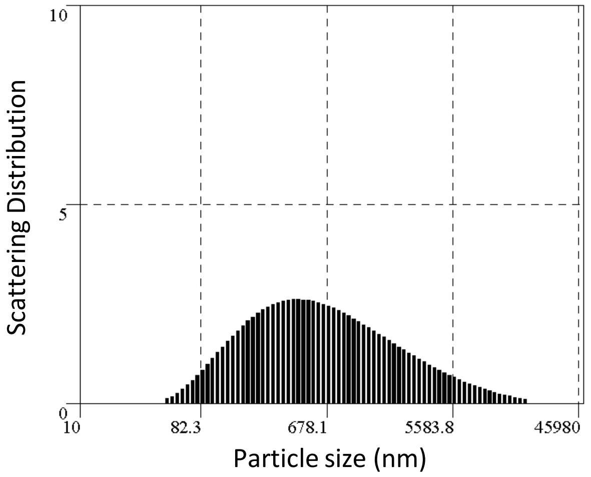

Histogram Analysis Results

Peak	Average (nm)	Standard Deviation
1	1304.1	± 2148.0

Data #2

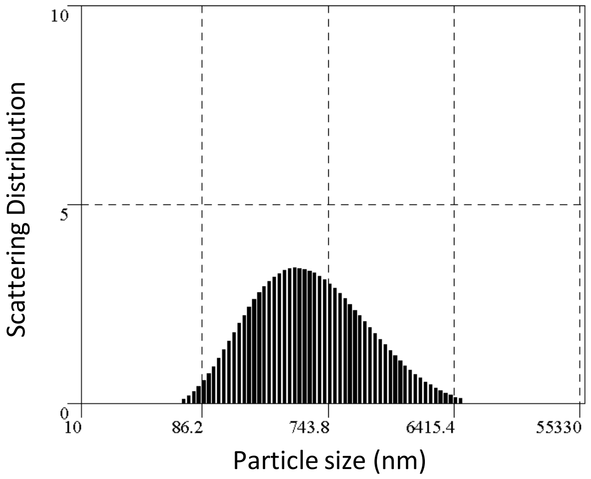

Histogram Analysis Results

Peak	Average (nm)	Standard Deviation
1	801.5	± 918.4

Data #3

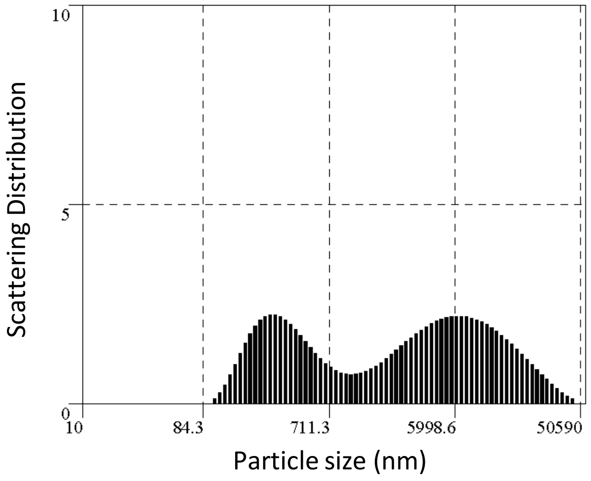

Histogram Analysis Results

Peak	Average (nm)	Standard Deviation
1	339.2	± 187.1
2	8209.4	± 7283.2

Data #4

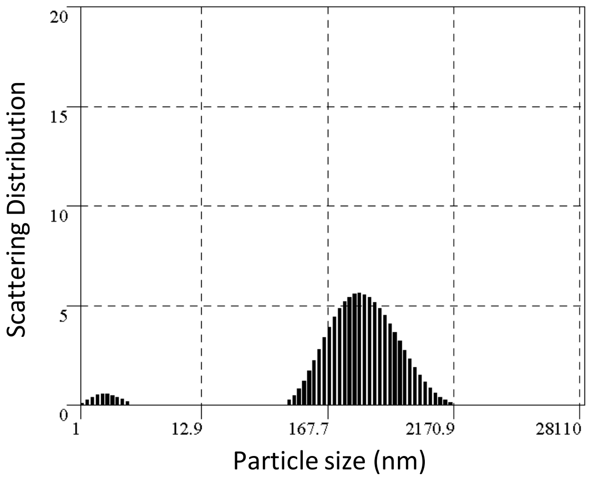

Histogram Analysis Results

Peak	Average (nm)	Standard Deviation
1	1.7	± 0.4
2	397.6	± 283.4

Data #5

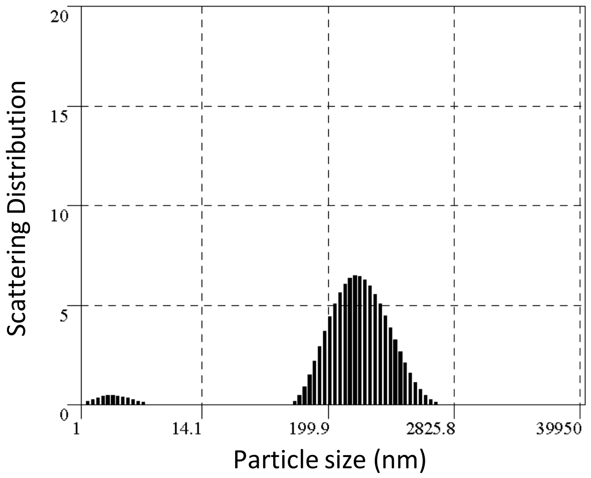

Histogram Analysis Results

Peak	Average (nm)	Standard Deviation
1	2.0	± 0.6
2	426.9	± 266.7

Scattering distribution of monomethyl maleate Na (**3b'**) 250 mM

Data #1

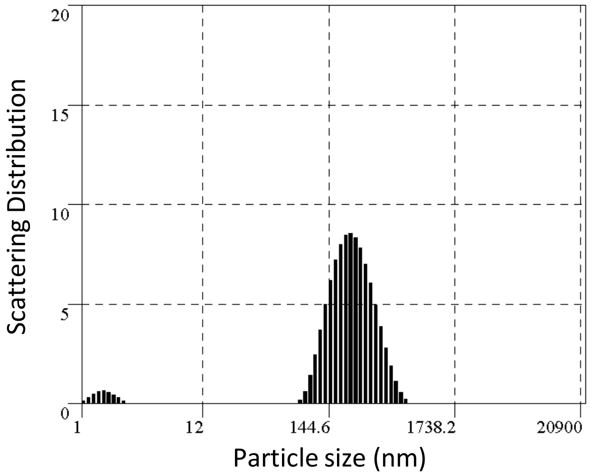

Histogram Analysis Results

Peak	Average (nm)	Standard Deviation
1	1.5	± 0.3
2	227.8	± 97.4

Data #2

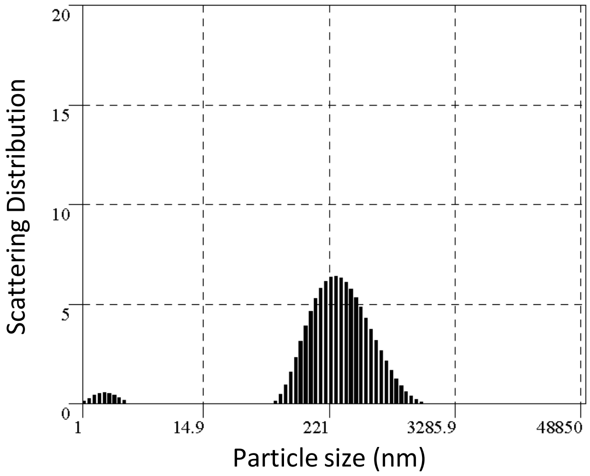

Histogram Analysis Results

Peak	Average (nm)	Standard Deviation
1	1.6	± 0.4
2	314.5	± 212.9

Data #3

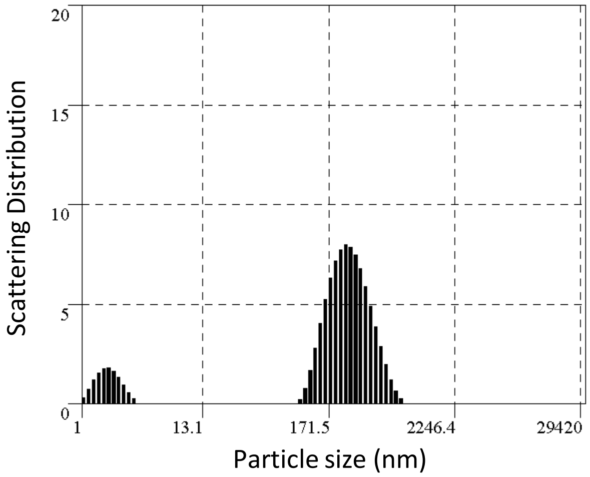

Histogram Analysis Results

Peak	Average (nm)	Standard Deviation
1	1.7	± 0.4
2	258.0	± 111.8

Data #4

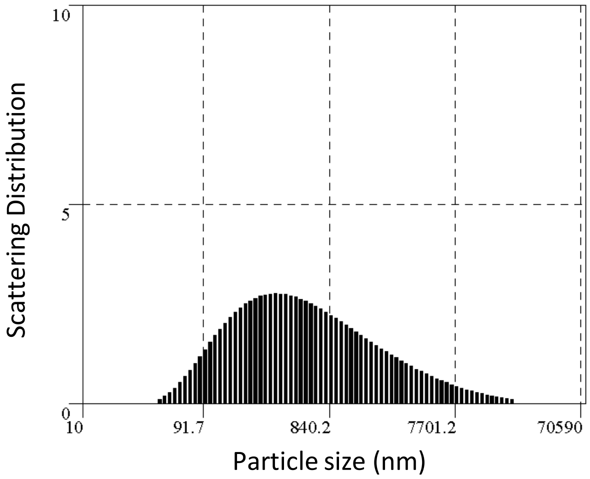

Histogram Analysis Results

Peak	Average (nm)	Standard Deviation
1	1228.8	± 2235.8

Data #5

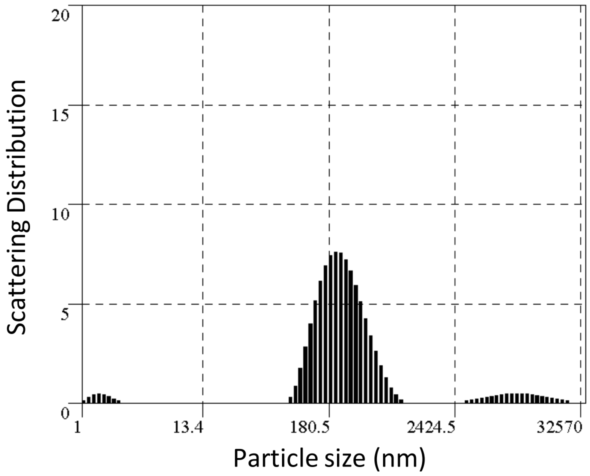

Histogram Analysis Results

Peak	Average (nm)	Standard Deviation
1	1.5	± 0.3
2	234.9	± 115.2
3	9544.9	± 5030.4

Scattering distribution of monomethyl maleate Na (**3b'**) 100 mM

Data #1

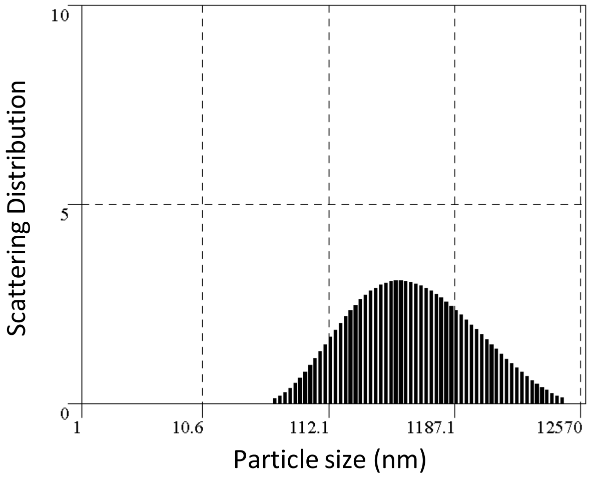

Histogram Analysis Results

Peak	Average (nm)	Standard Deviation
1	923.2	± 1198.7

Data #2

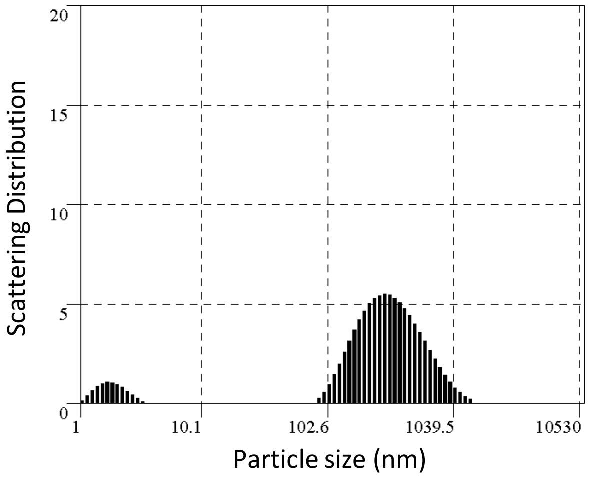

Histogram Analysis Results

Peak	Average (nm)	Standard Deviation
1	1.8	± 0.5
2	352.5	± 216.4

Data #3

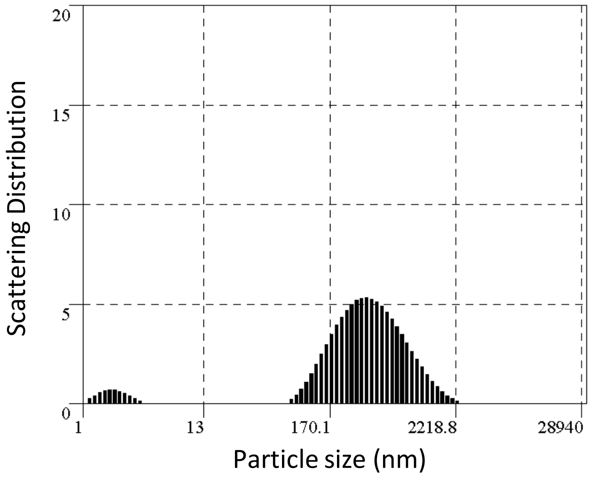

Histogram Analysis Results

Peak	Average (nm)	Standard Deviation
1	1.8	± 0.5
2	440.5	± 325.0

Data #4

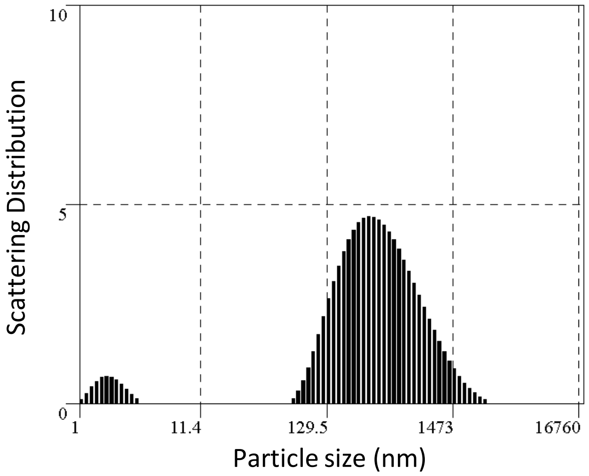

Histogram Analysis Results

Peak	Average (nm)	Standard Deviation
1	1.8	± 0.5
2	433.0	± 363.7

Data #5

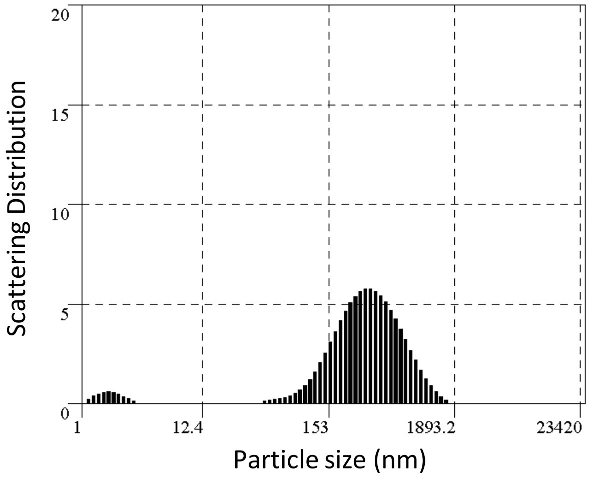

Histogram Analysis Results

Peak	Average (nm)	Standard Deviation
1	1.7	± 0.4
2	374.0	± 244.3

Scattering distribution of monomethyl maleate Na (**3b'**) 50 mM

Data #1

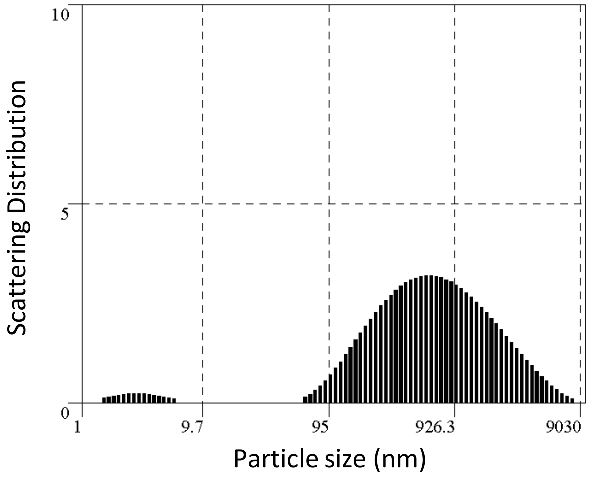

Histogram Analysis Results

Peak	Average (nm)	Standard Deviation
1	2.9	± 1.0
2	985.1	± 1081.2

Data #2

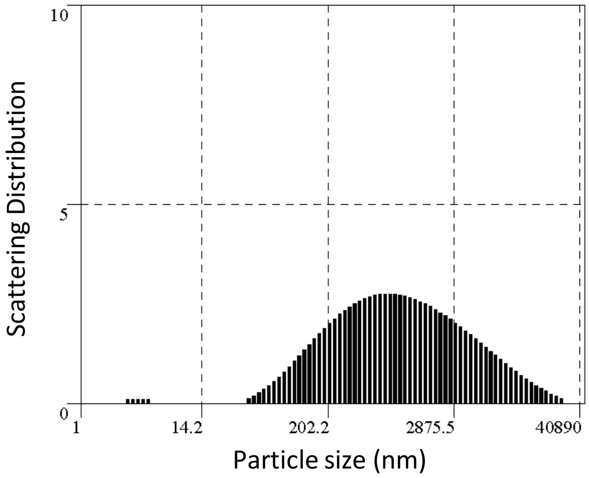

Histogram Analysis Results

Peak	Average (nm)	Standard Deviation
1	3.3	± 0.5
2	2122.5	± 3430.1

Data #3

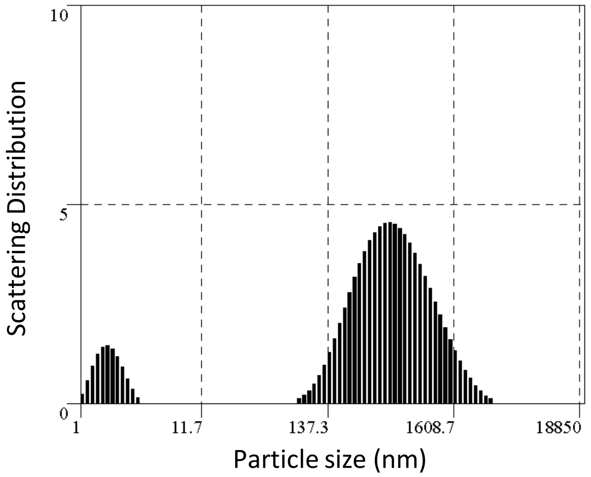

Histogram Analysis Results

Peak	Average (nm)	Standard Deviation
1	1.7	± 0.4
2	589.6	± 465.9

Data #4

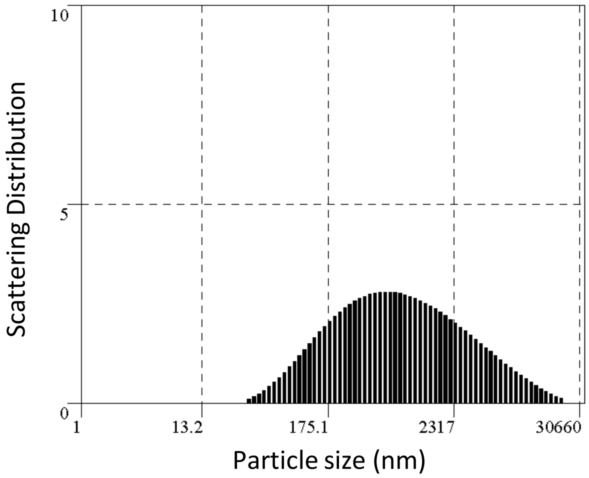

Histogram Analysis Results

Peak	Average (nm)	Standard Deviation
1	1664.4	± 2613.1

Data #5

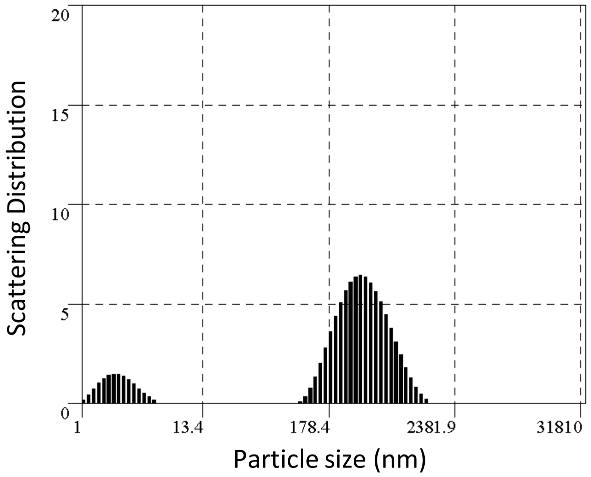

Histogram Analysis Results

Peak	Average (nm)	Standard Deviation
1	2.1	± 0.7
2	375.9	± 202.9

Scattering distribution of monomethyl maleate Na (**3b'**) 20 mM

Data #1

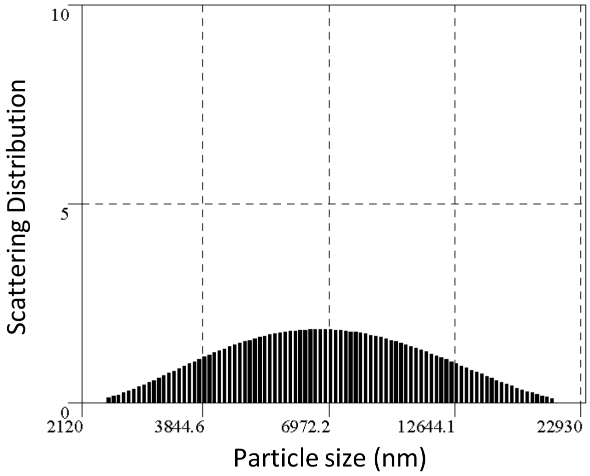

Histogram Analysis Results

Peak	Average (nm)	Standard Deviation
1	7455.0	± 3485.6
2	1602.3	± 2410.7

Data #2

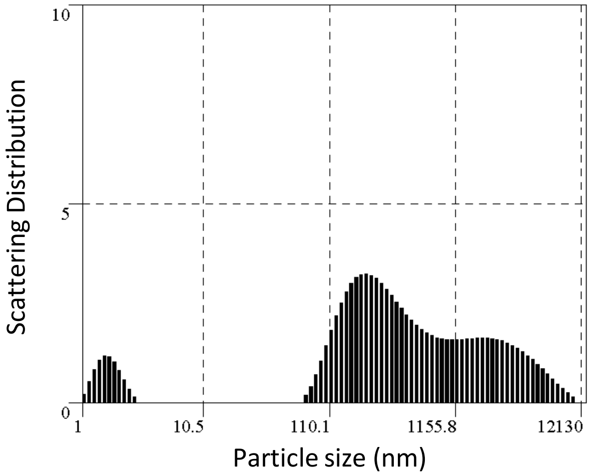

Histogram Analysis Results

Peak	Average (nm)	Standard Deviation
1	1.6	± 0.4
2	324.9	± 219.9
3	2958.8	± 1869.8

Data #3

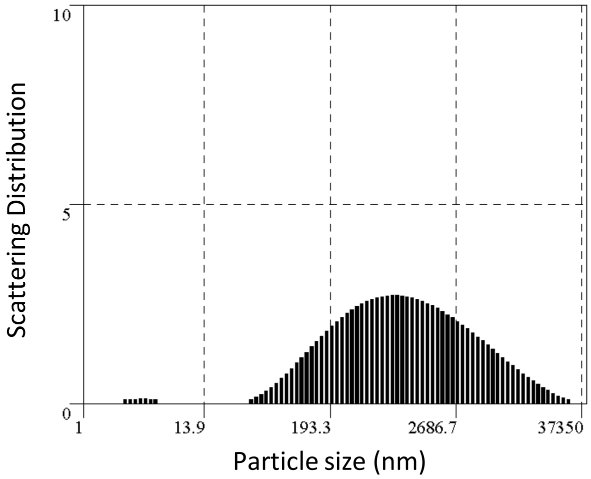

Histogram Analysis Results

Peak	Average (nm)	Standard Deviation
1	3.3	± 0.7
2	2079.6	± 3336.7

Data #4

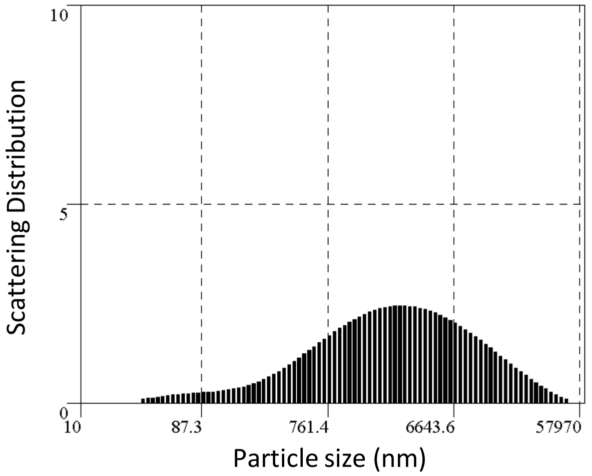

Histogram Analysis Results

Peak	Average (nm)	Standard Deviation
1	4696.0	± 6292.2

Data #5

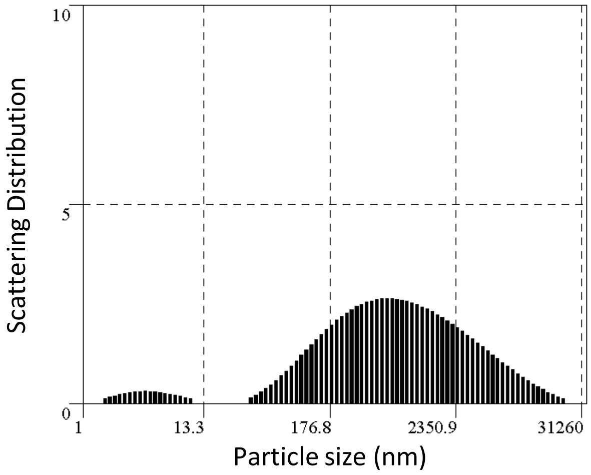

Histogram Analysis Results

Peak	Average (nm)	Standard Deviation
1	4.1	± 1.9
2	1674.0	± 2656.0

Scattering distribution of monomethyl maleate Na (**3b'**) 10 mM

Data #1

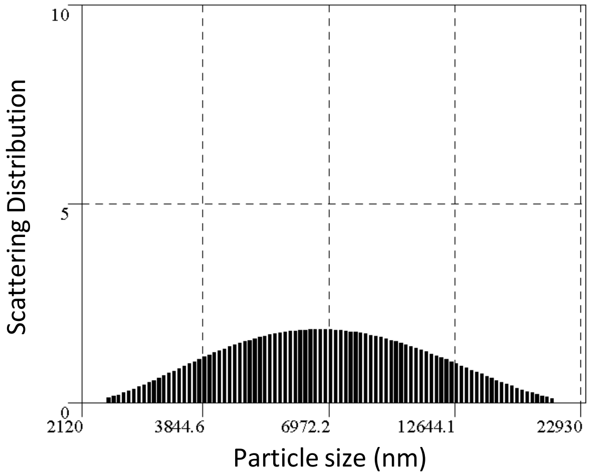

Histogram Analysis Results

Peak	Average (nm)	Standard Deviation
1	7455.0	± 3485.6

Data #2

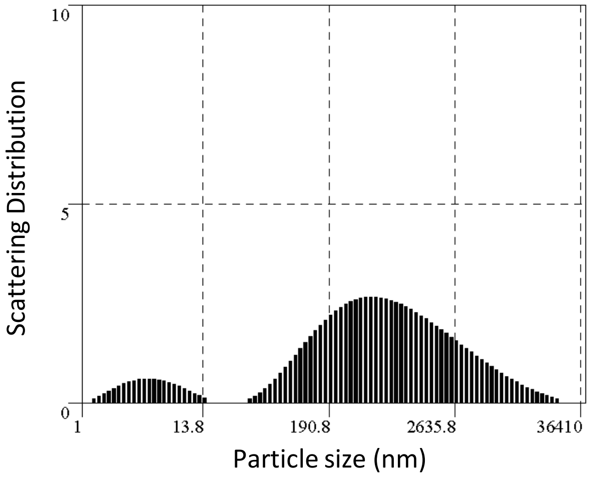

Histogram Analysis Results

Peak	Average (nm)	Standard Deviation
1	4.7	± 2.7
2	1532.9	± 2576.2

Data #3

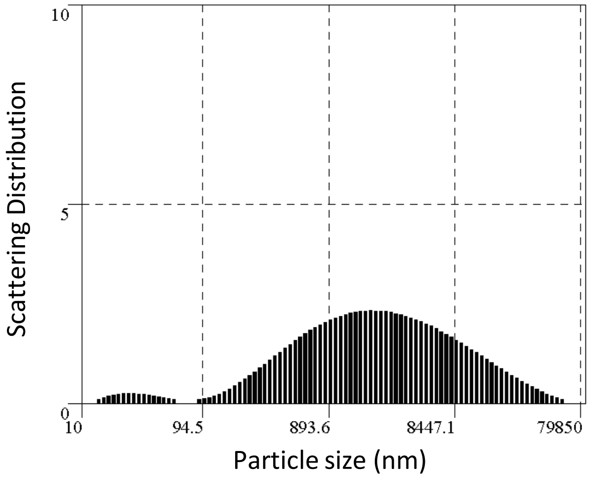

Histogram Analysis Results

Peak	Average (nm)	Standard Deviation
1	27.0	± 10.1
2	4872.6	± 7342.1

Data #4

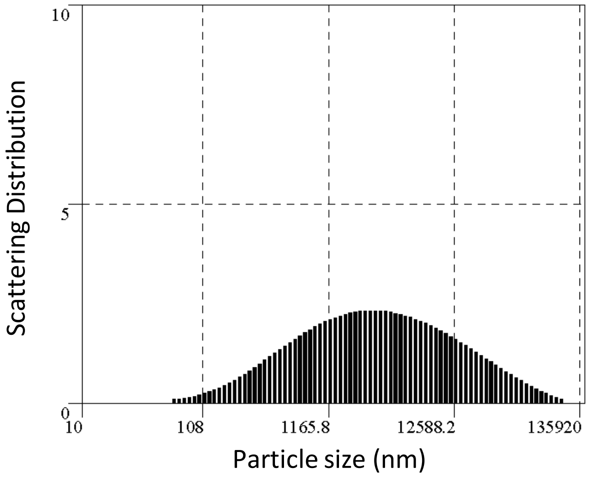

Histogram Analysis Results

Peak	Average (nm)	Standard Deviation
1	7180.1	± 11759.6

Data #5

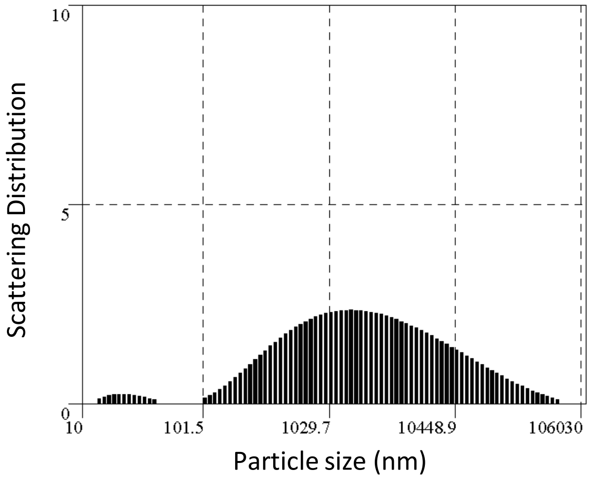

Histogram Analysis Results

Peak	Average (nm)	Standard Deviation
1	22.5	± 6.6
2	5032.6	± 8357.6

Scattering distribution of monomethylphenylmalonate Na (**4b'**) 250 mM

Data #1

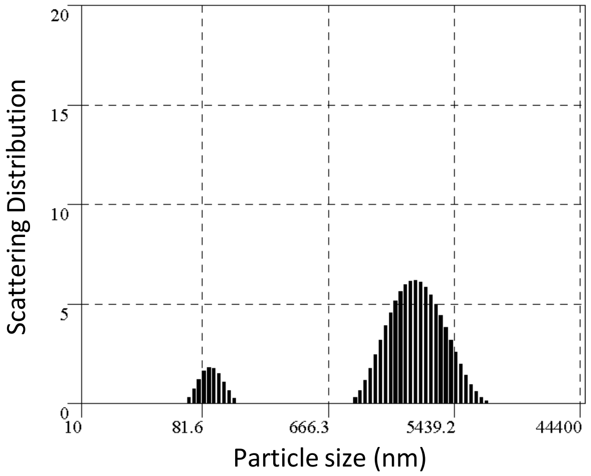

Histogram Analysis Results

Peak	Average (nm)	Standard Deviation
1	87.7	± 16.1
2	3024.3	± 1386.9

Data #2

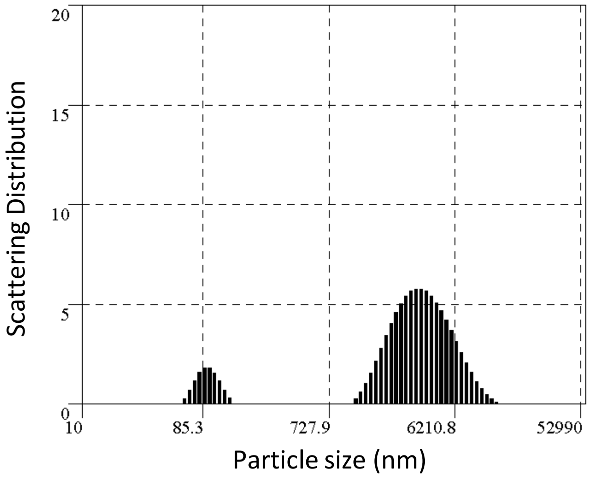

Histogram Analysis Results

Peak	Average (nm)	Standard Deviation
1	85.1	± 15.9
2	3725.6	± 1885.4

Data #3

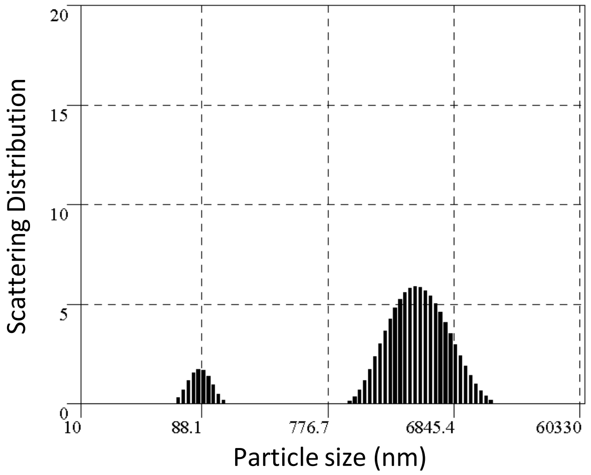

Histogram Analysis Results

Peak	Average (nm)	Standard Deviation
1	78.8	± 14.5
2	3957.0	± 2013.7

Data #4

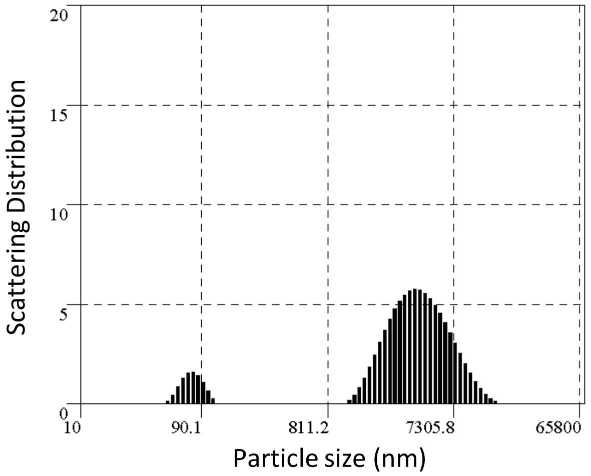

Histogram Analysis Results

Peak	Average (nm)	Standard Deviation
1	70.0	± 13.0
2	4255.4	± 2278.0

Data #5

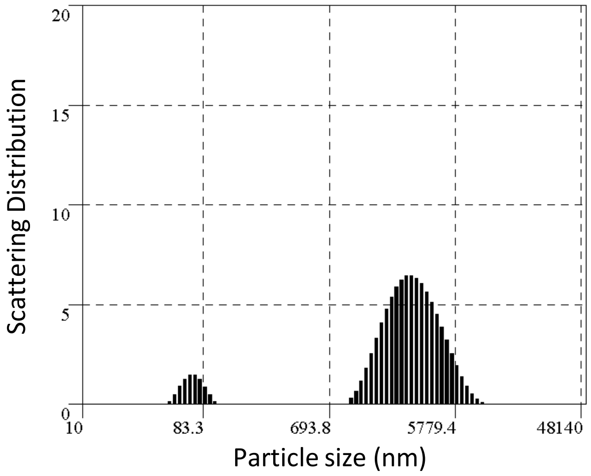

Histogram Analysis Results

Peak	Average (nm)	Standard Deviation
1	64.0	± 11.2
2	2909.1	± 1332.6

Scattering distribution of monomethylphenylmalonate Na (**4b'**) 100 mM

Data #1

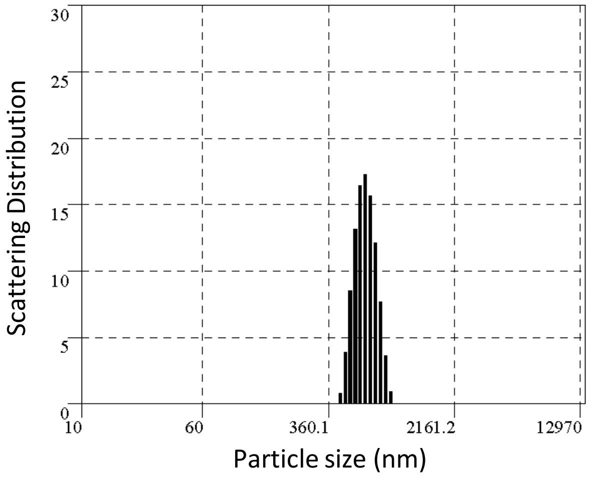

Histogram Analysis Results

Peak	Average (nm)	Standard Deviation
1	580.9	± 88.8

Data #2

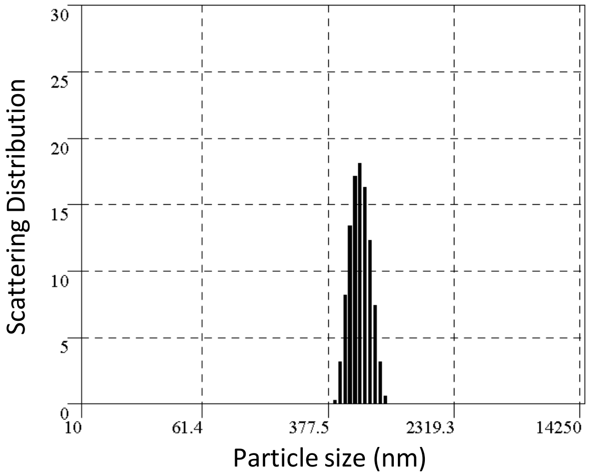

Histogram Analysis Results

Peak	Average (nm)	Standard Deviation
1	569.6	± 84.0

Data #3

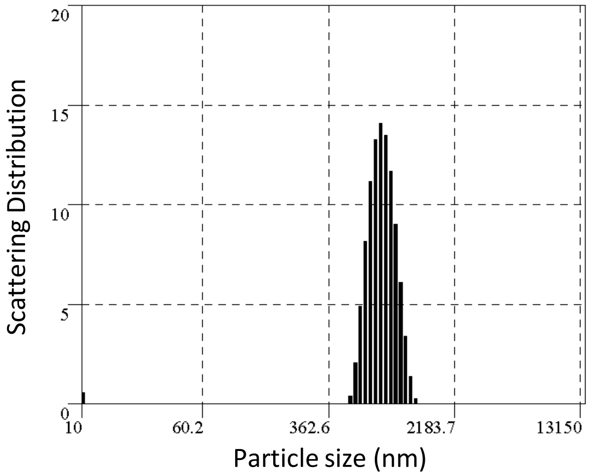

Histogram Analysis Results

Peak	Average (nm)	Standard Deviation
1	747.6	± 140.9

Data #4

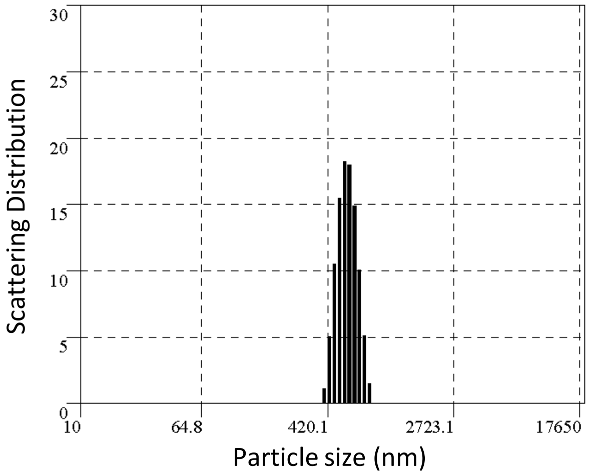

Histogram Analysis Results

Peak	Average (nm)	Standard Deviation
1	532.7	± 79.1

Data #5

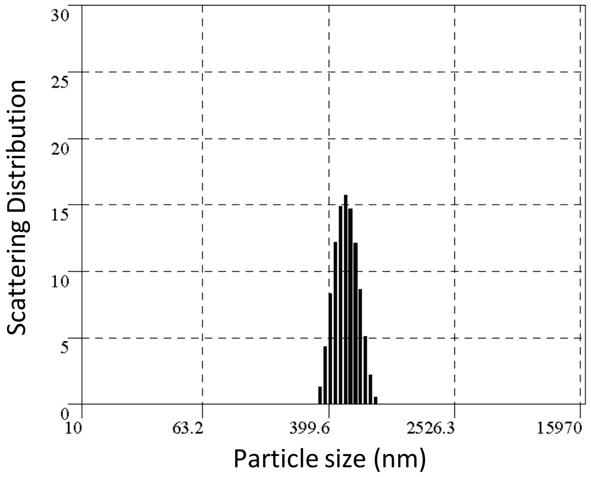

Histogram Analysis Results

Peak	Average (nm)	Standard Deviation
1	493.0	± 85.7

Scattering distribution of monomethylphenylmalonate Na (**4b'**) 50 mM

Data #1

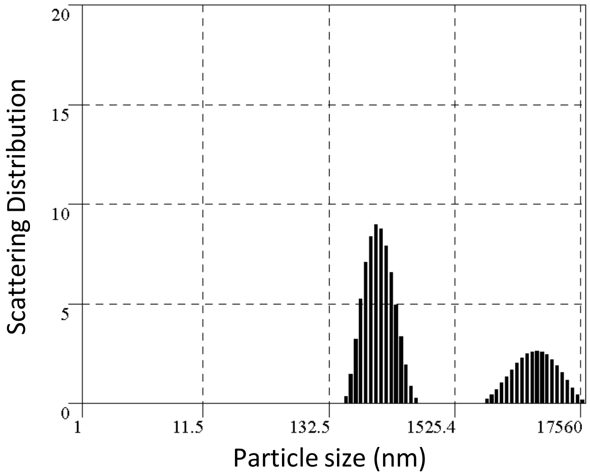

Histogram Analysis Results

Peak	Average (nm)	Standard Deviation
1	337.6	± 96.3
2	7576.8	± 3090.9

Data #2

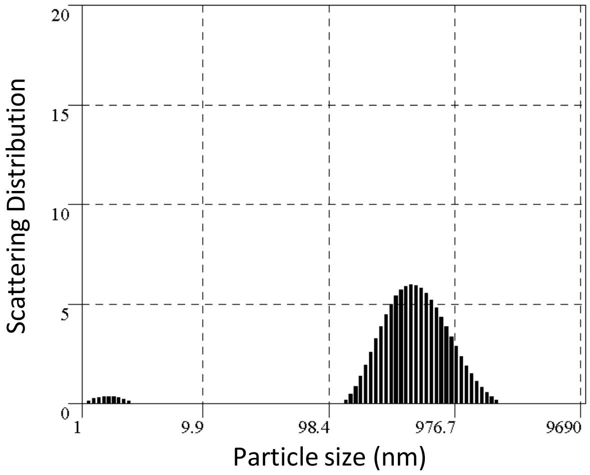

Histogram Analysis Results

Peak	Average (nm)	Standard Deviation
1	1.6	± 0.3
2	531.9	± 316.9

Data #3

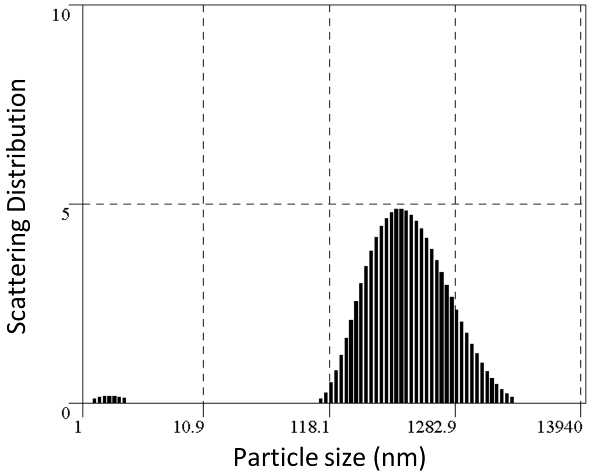

Histogram Analysis Results

Peak	Average (nm)	Standard Deviation
1	1.7	± 0.3
2	647.5	± 531.8

Data #4

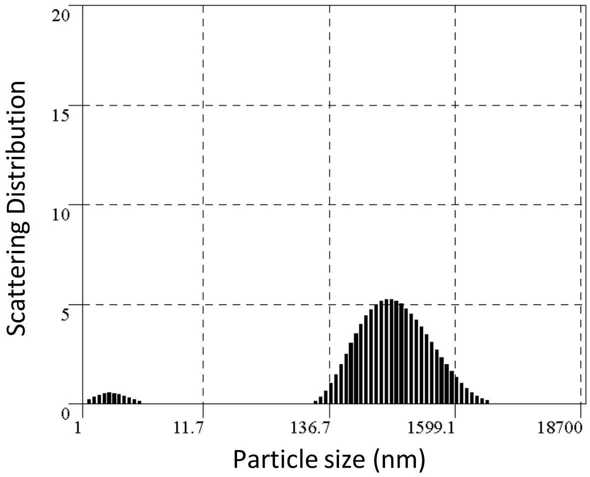

Histogram Analysis Results

Peak	Average (nm)	Standard Deviation
1	1.8	± 0.5
2	577.2	± 427.7

Data #5

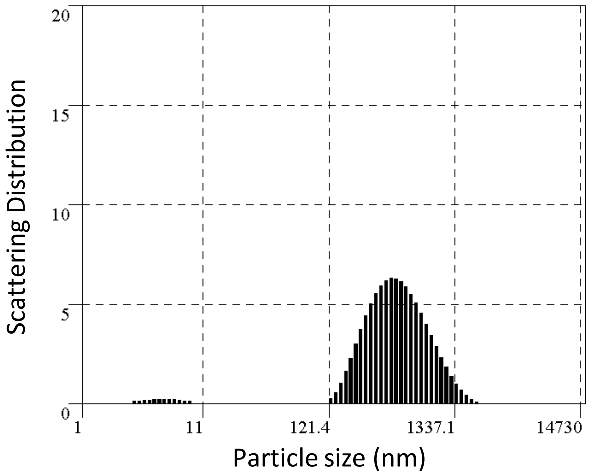

Histogram Analysis Results

Peak	Average (nm)	Standard Deviation
1	4.7	± 1.4
2	483.5	± 286.2

Scattering distribution of monomethylphenylmalonate Na (**4b'**) 20 mM

Data #1

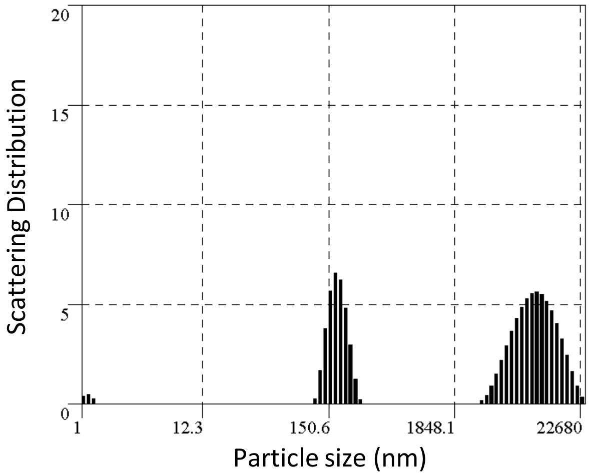

Histogram Analysis Results

Peak	Average (nm)	Standard Deviation
1	1.1	± 0.1
2	167.0	± 31.6
3	9549.8	± 4012.8

Data #2

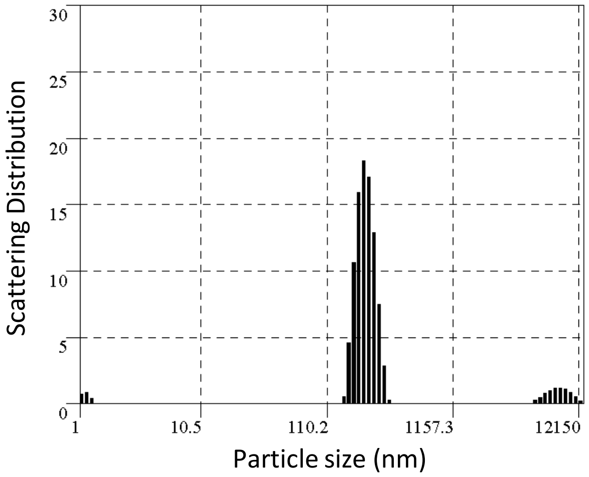

Histogram Analysis Results

Peak	Average (nm)	Standard Deviation
1	1.1	± 0.1
2	212.9	± 36.5
3	8172.5	± 1723.8

Data #3

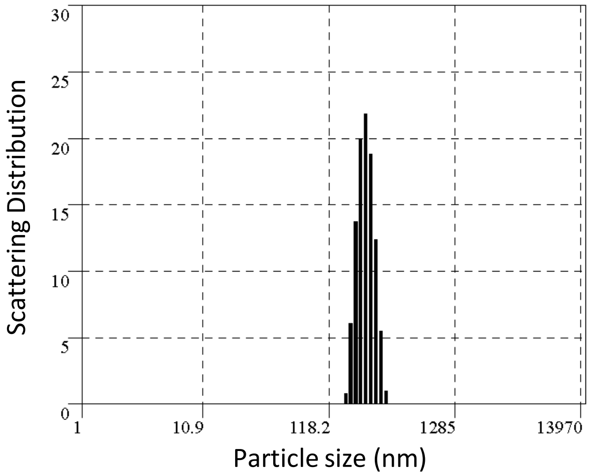

Histogram Analysis Results

Peak	Average (nm)	Standard Deviation
1	223.0	± 35.9

Data #4

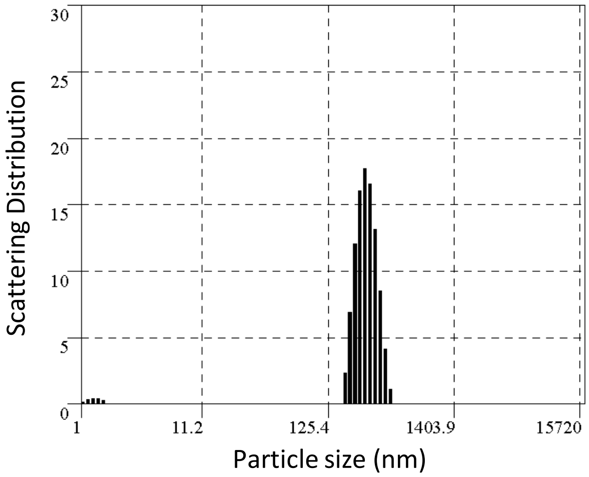

Histogram Analysis Results

Peak	Average (nm)	Standard Deviation
1	1.2	± 0.1
2	246.0	± 48.9

Data #5

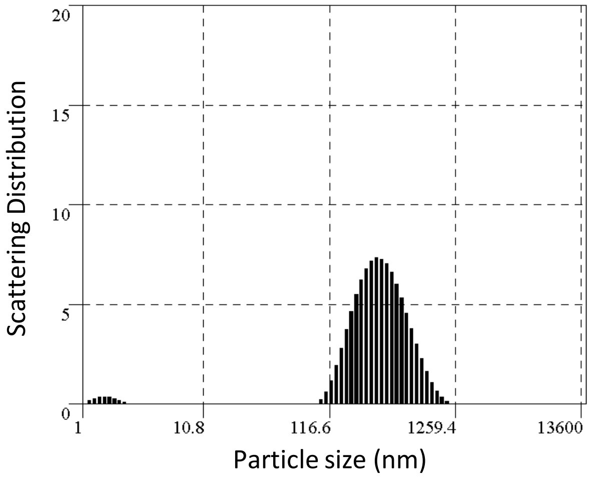

Histogram Analysis Results

Peak	Average (nm)	Standard Deviation
1	1.5	± 0.3
2	316.4	± 155.6

Scattering distribution of monomethylphenylmalonate Na (**4b'**) 10 mM

Data #1

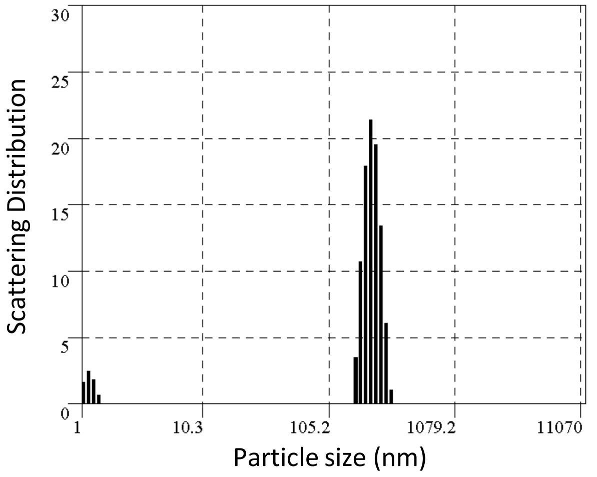

Histogram Analysis Results

Peak	Average (nm)	Standard Deviation
1	1.1	± 0.1
2	219.5	± 32.7

Data #2

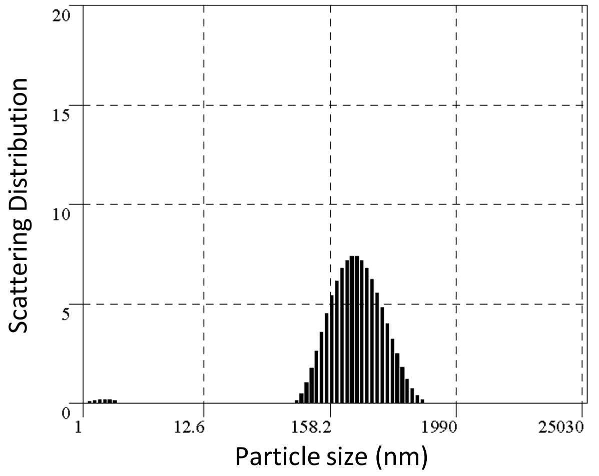

Histogram Analysis Results

Peak	Average (nm)	Standard Deviation
1	1.5	± 0.2
2	283.1	± 148.9

Data #3

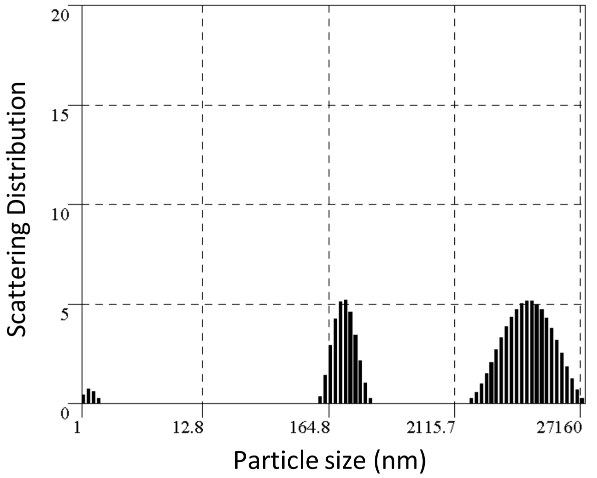

Histogram Analysis Results

Peak	Average (nm)	Standard Deviation
1	1.2	± 0.1
2	213.8	± 47.6
3	10053.6	± 4821.2

Data #4

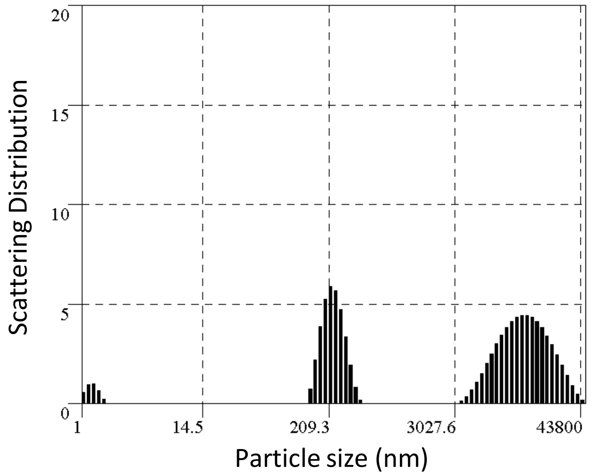

Histogram Analysis Results

Peak	Average (nm)	Standard Deviation
1	1.2	± 0.2
2	213.7	± 50.0
3	14326.9	± 7738.2

Data #5

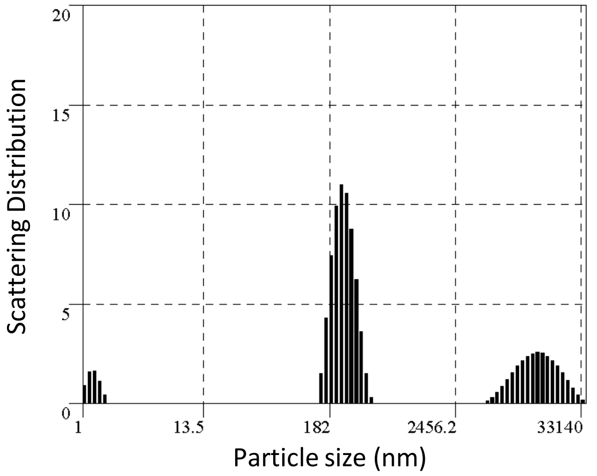

Histogram Analysis Results

Peak	Average (nm)	Standard Deviation
1	1.2	± 0.1
2	228.4	± 52.4
3	13834.3	± 5893.0

Scattering distribution of monomethyl malonate K (**5b'**) 250 mM

Data #1

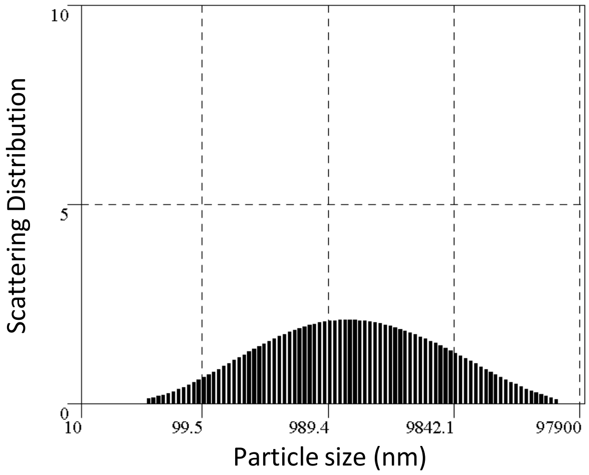

Histogram Analysis Results

Peak	Average (nm)	Standard Deviation
1	4370.2	± 7680.8

Data #2

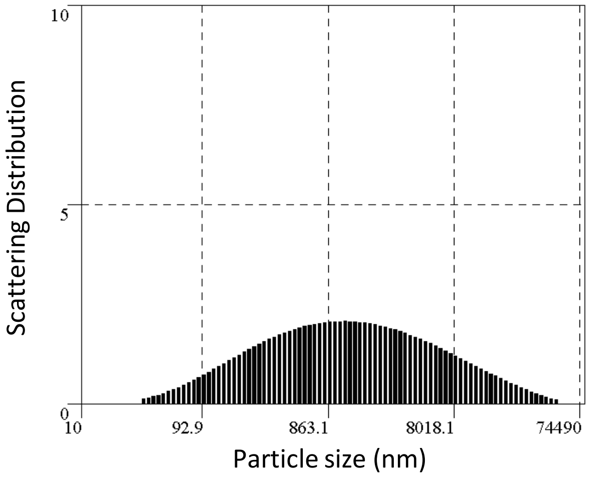

Histogram Analysis Results

Peak	Average (nm)	Standard Deviation
1	3413.8	± 5921.5

Data #3

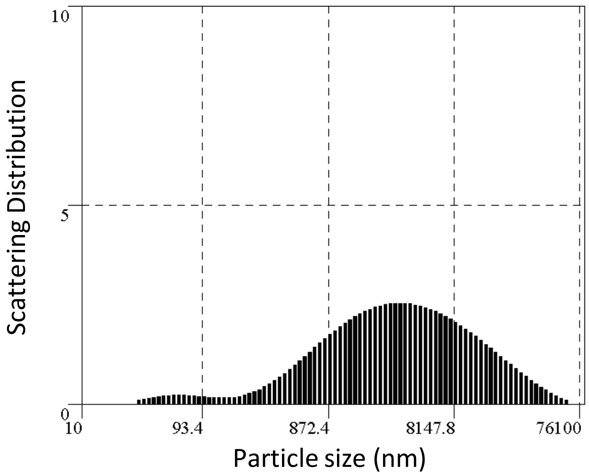

Histogram Analysis Results

Peak	Average (nm)	Standard Deviation
1	61.8	± 24.8
2	6115.6	± 8106.2

Data #4

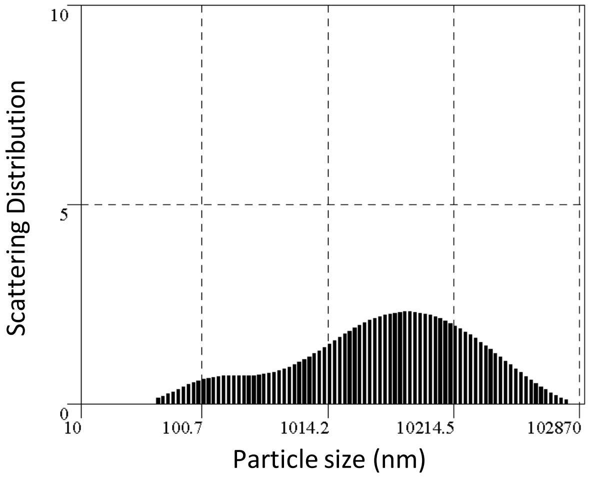

Histogram Analysis Results

Peak	Average (nm)	Standard Deviation
1	7207.3	± 10630.6

Data #5

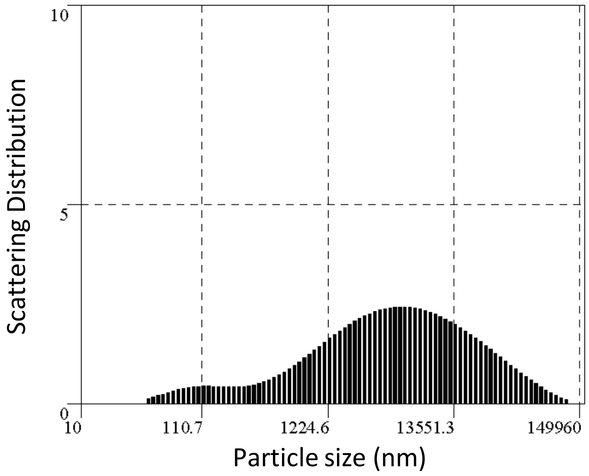

Histogram Analysis Results

Peak	Average (nm)	Standard Deviation
1	95.0	± 38.3
2	10421.7	± 15067.9

Scattering distribution of monomethyl malonate K (**5b'**) 100 mM

Data #1

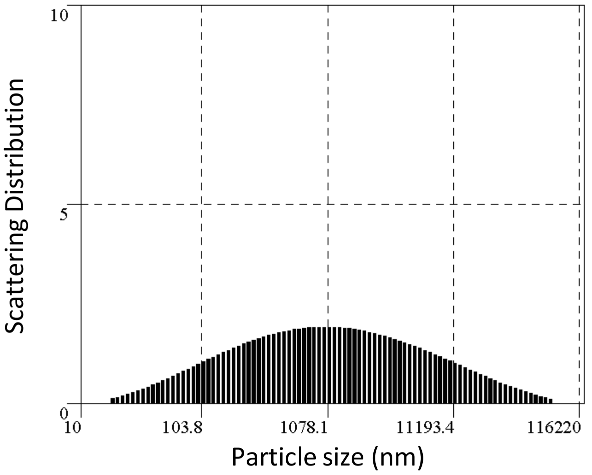

Histogram Analysis Results

Peak	Average (nm)	Standard Deviation
1	3975.8	± 7848.9

Data #2

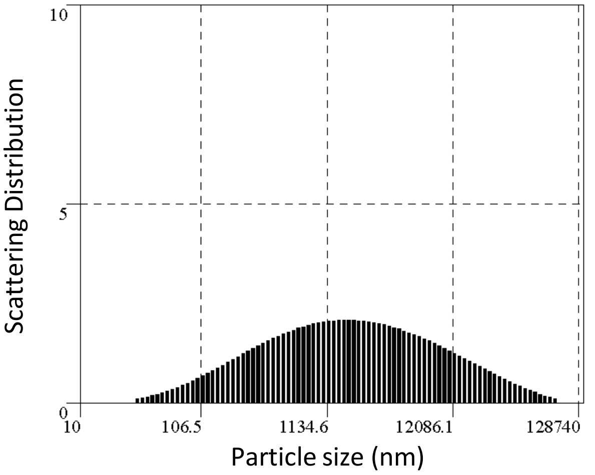

Histogram Analysis Results

Peak	Average (nm)	Standard Deviation
1	5335.2	± 9750.3

Data #3

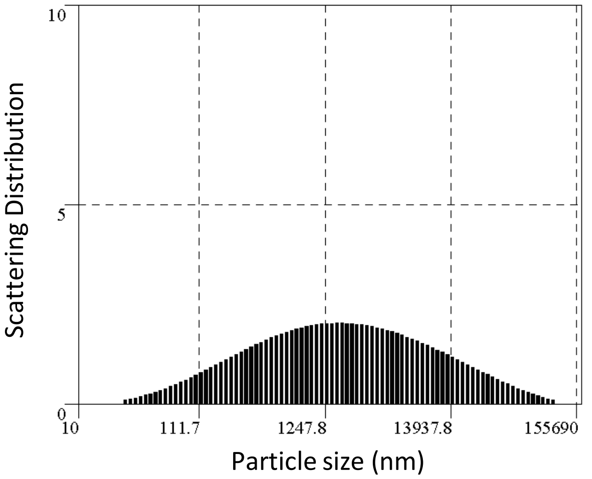

Histogram Analysis Results

Peak	Average (nm)	Standard Deviation
1	5831.0	± 11232.9

Data #4

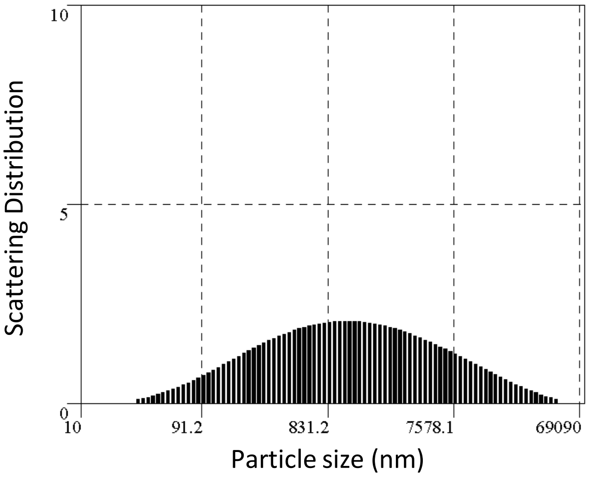

Histogram Analysis Results

Peak	Average (nm)	Standard Deviation
1	3298.6	± 5614.2

Data #5

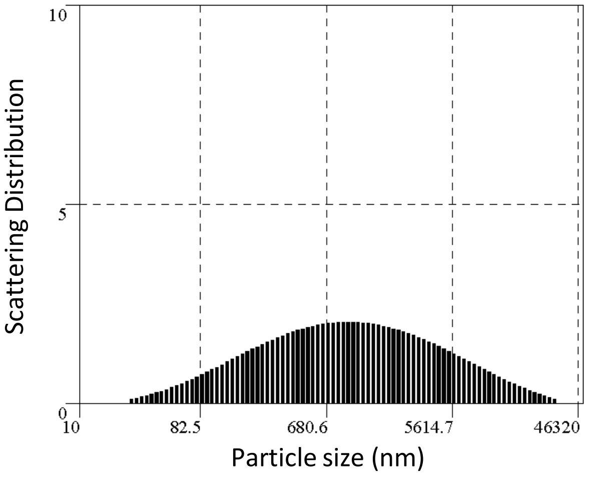

Histogram Analysis Results

Peak	Average (nm)	Standard Deviation
1	2466.6	± 3980.6

Scattering distribution of monomethyl malonate K (**5b'**) 50 mM

Data #1

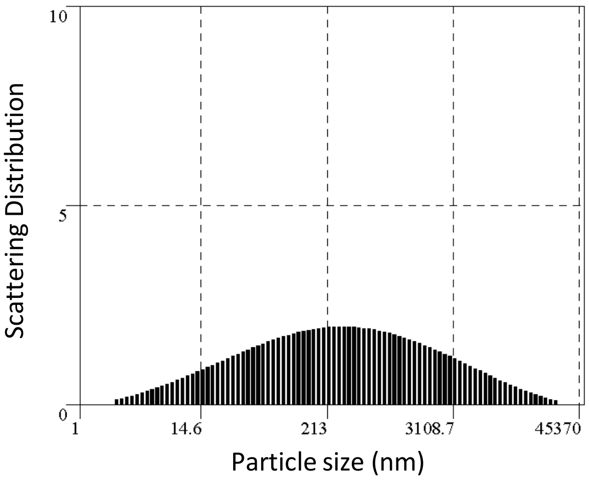

Histogram Analysis Results

Peak	Average (nm)	Standard Deviation
1	1305.1	± 2876.7

Data #2

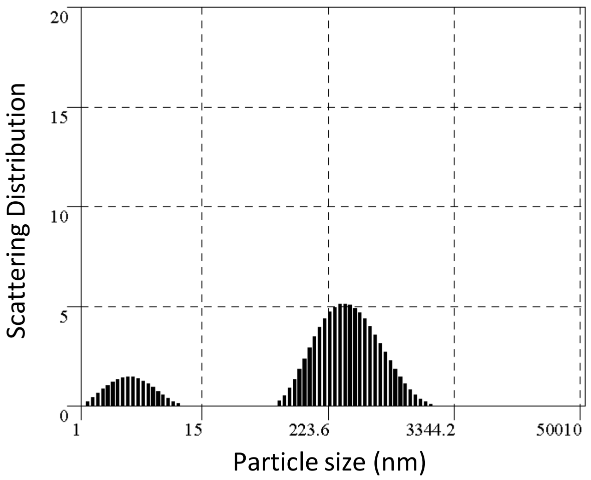

Histogram Analysis Results

Peak	Average (nm)	Standard Deviation
1	3.1	± 1.4
2	390.6	± 277.0

Data #3

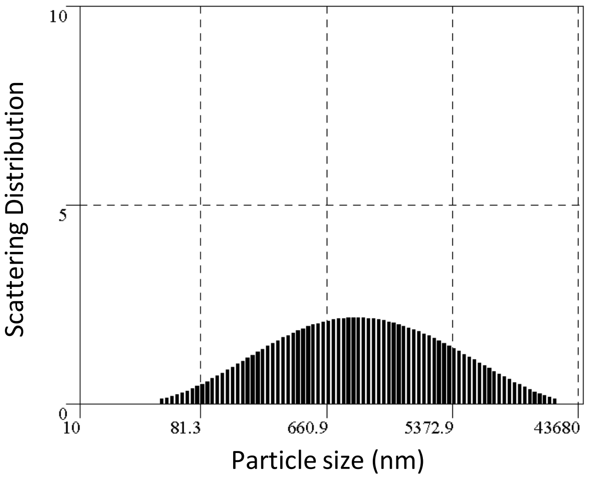

Histogram Analysis Results

Peak	Average (nm)	Standard Deviation
1	2598.4	± 3931.0

Data #4

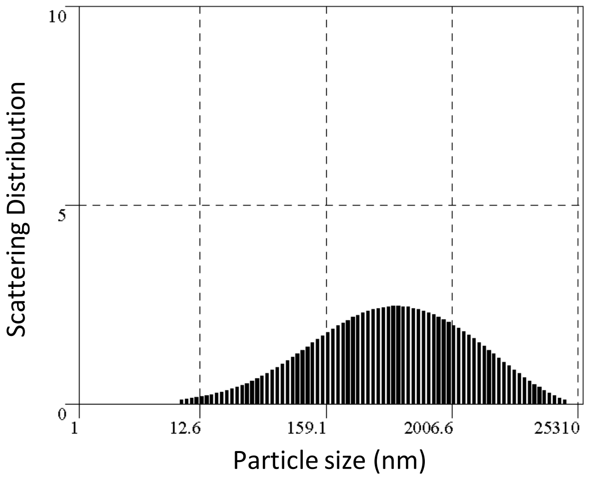

Histogram Analysis Results

Peak	Average (nm)	Standard Deviation
1	1481.3	± 2366.6

Data #5

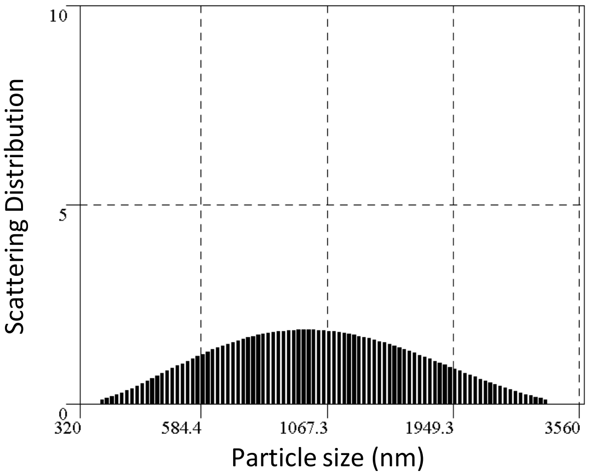

Histogram Analysis Results

Peak	Average (nm)	Standard Deviation
1	1102.1	± 523.7

Scattering distribution of monomethyl malonate K (**5b'**) 20 mM

Data #1

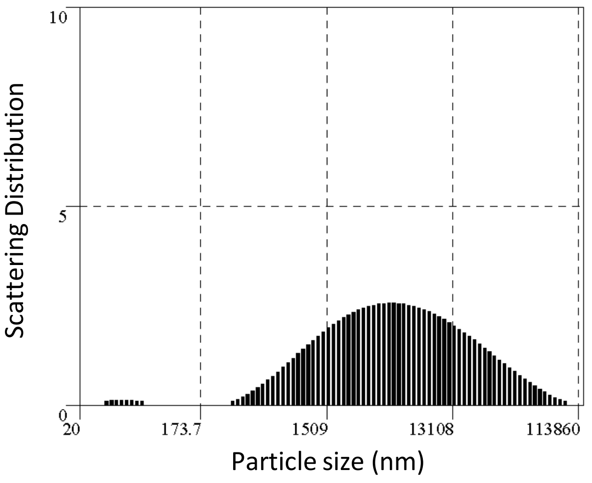

Histogram Analysis Results

Peak	Average (nm)	Standard Deviation
1	42.7	± 8.2
2	9229.2	± 12109.0

Data #2

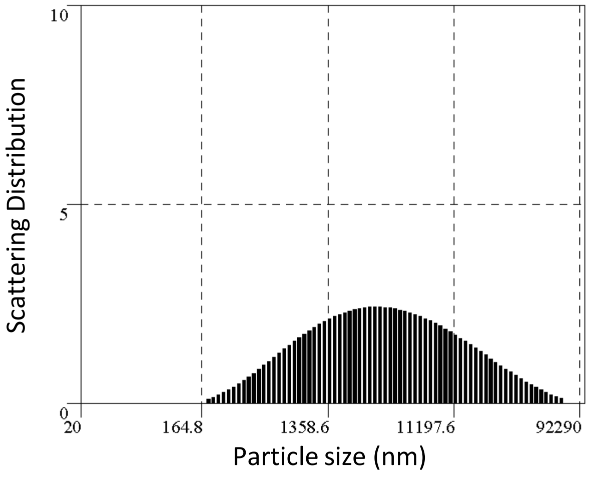

Histogram Analysis Results

Peak	Average (nm)	Standard Deviation
1	6670.3	± 9176.2

Data #3

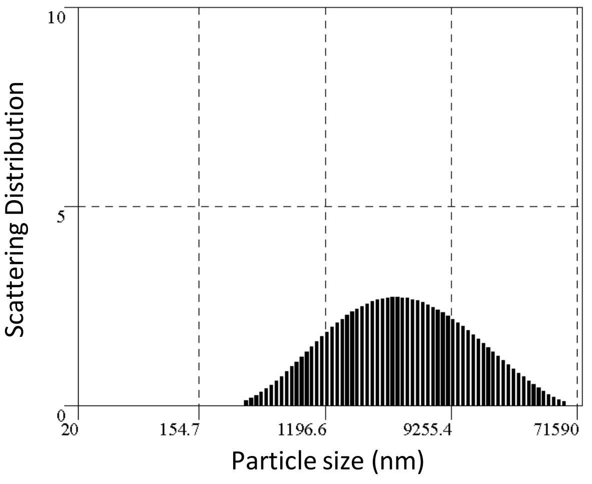

Histogram Analysis Results

Peak	Average (nm)	Standard Deviation
1	6837.1	± 8079.4

Data #4

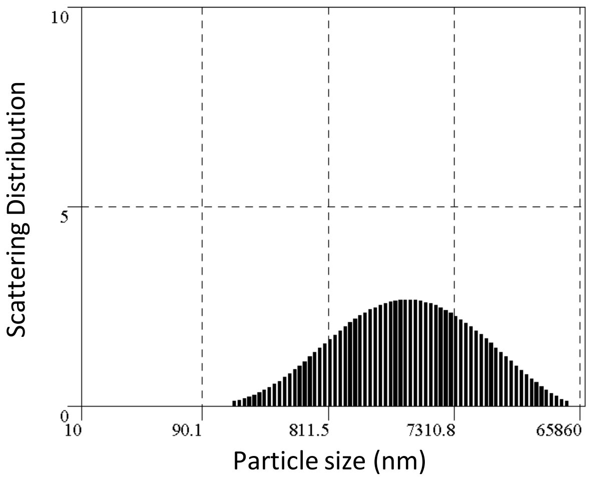

Histogram Analysis Results

Peak	Average (nm)	Standard Deviation
1	5813.2	± 7335.5

Data #5

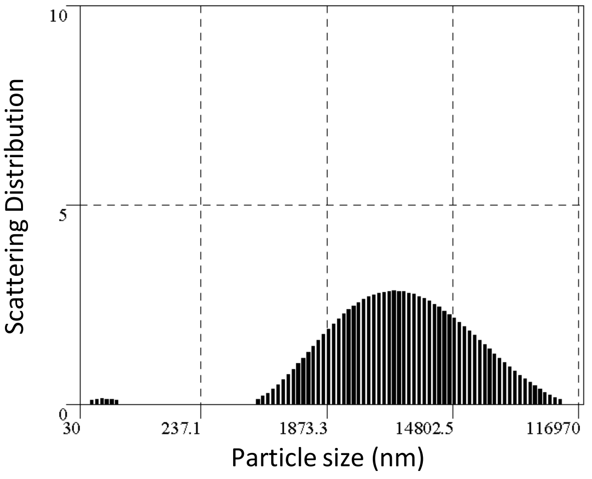

Histogram Analysis Results

Peak	Average (nm)	Standard Deviation
1	44.0	± 5.9
2	10490.4	± 12245.6

Scattering distribution of monomethyl malonate K (**5b'**) 10 mM

Data #1

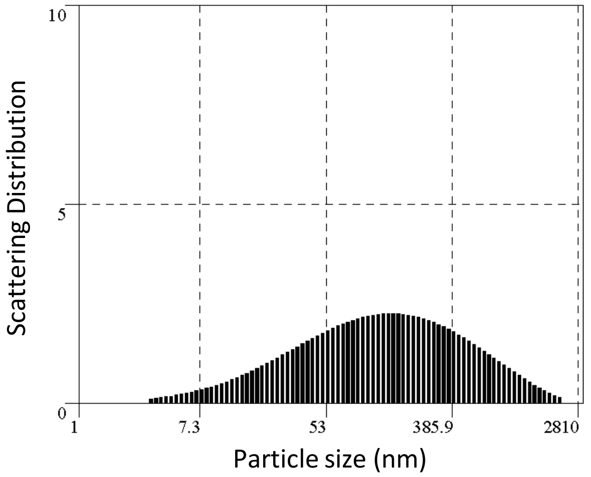

Histogram Analysis Results

Peak	Average (nm)	Standard Deviation
1	239.5	± 309.4

Data #2

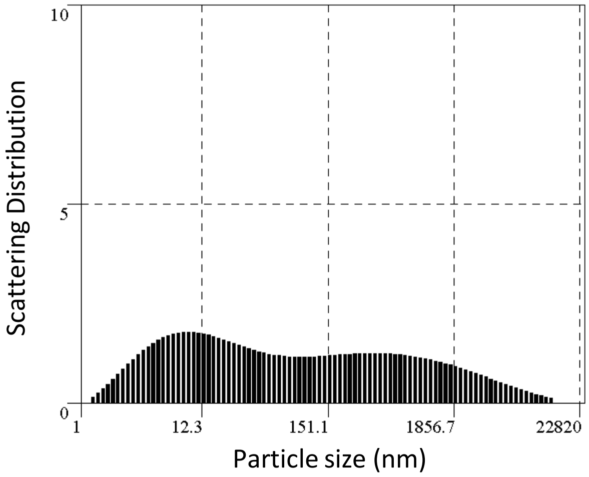

Histogram Analysis Results

Peak	Average (nm)	Standard Deviation
1	18.1	± 17.7
2	1281.5	± 1895.9

Data #3

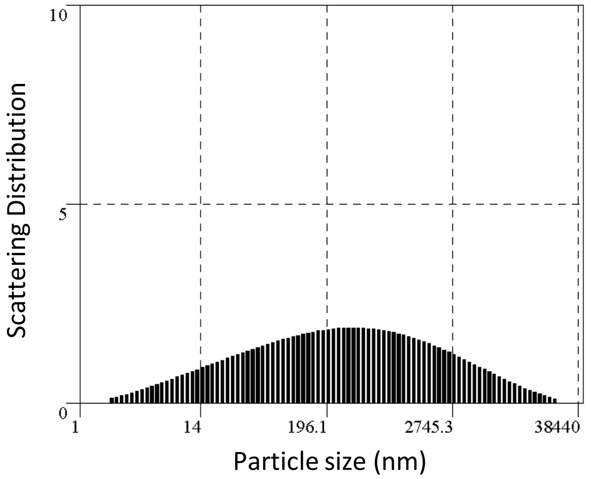

Histogram Analysis Results

Peak	Average (nm)	Standard Deviation
1	1222.4	± 2587.8

Data #4

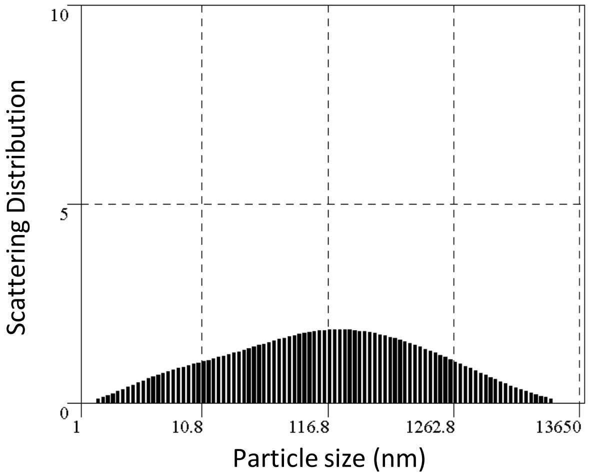

Histogram Analysis Results

Peak	Average (nm)	Standard Deviation
1	457.2	± 915.9

Data #5

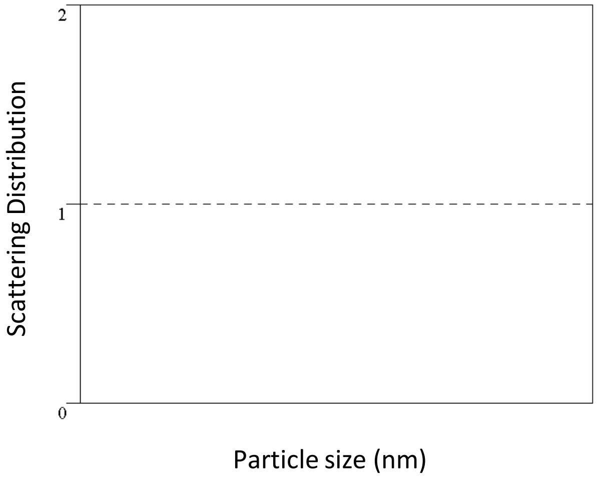

Histogram Analysis Results

Peak	Average (nm)	Standard Deviation
1	0.7	± 0.1
2	1.3	± 0.3
3	85.5	± 11.0

Zeta Potential of monomethyl norbornadiene Na (**1b'**) 250 mM

Electro-osmosis plot: Mobility distribution inside cell

Data #1

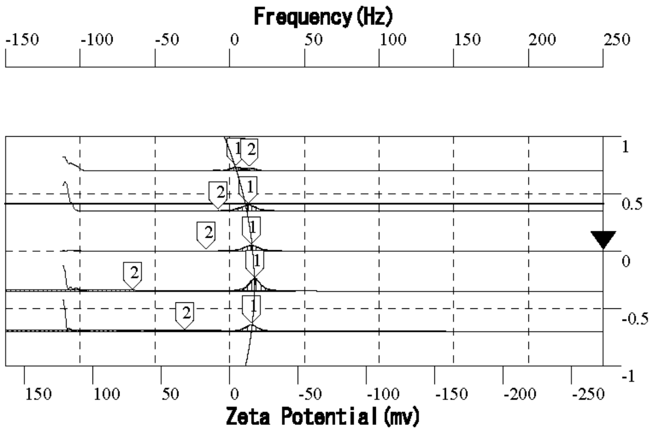

Zeta potential : −11.48 (mV)

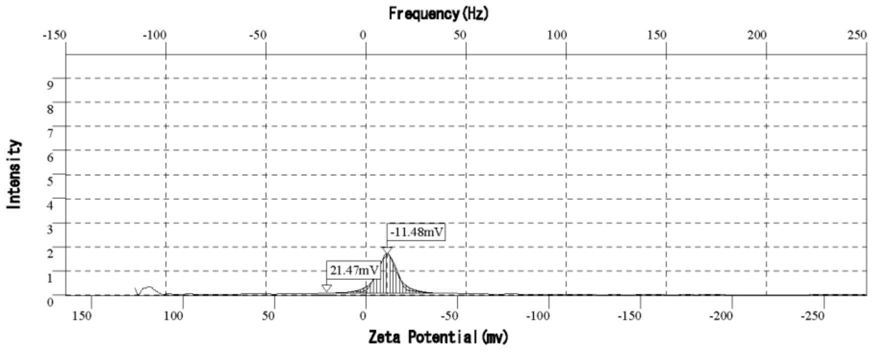

Data #2

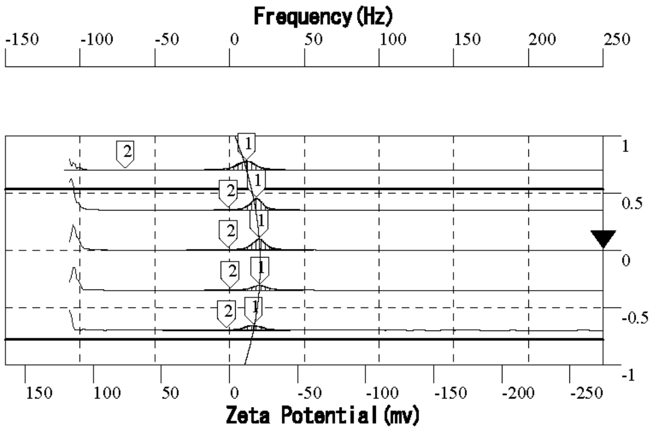

Zeta potential : −16.16 (mV)

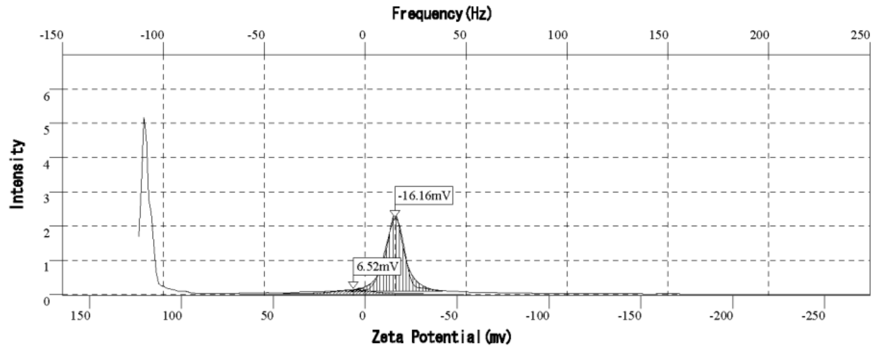

Data #3

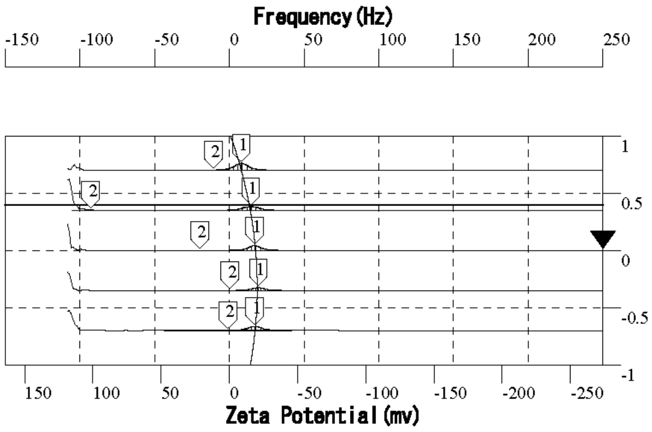

Zeta potential : −14.65 (mV)

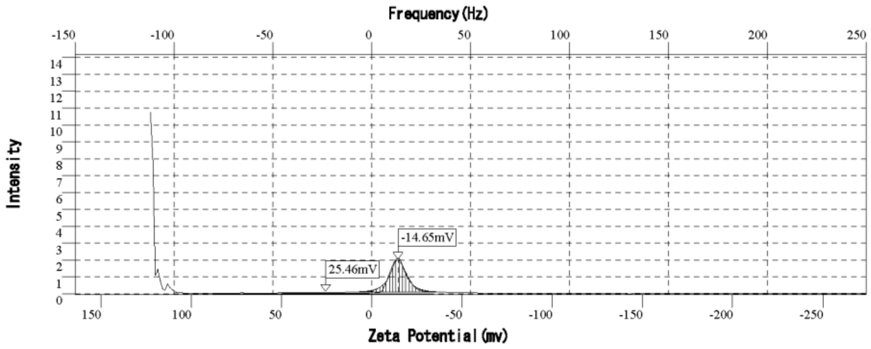

Data #4

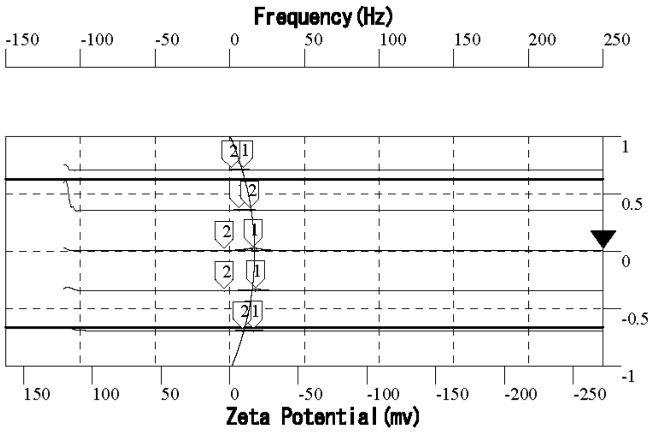

Zeta potential : −11.01 (mV)

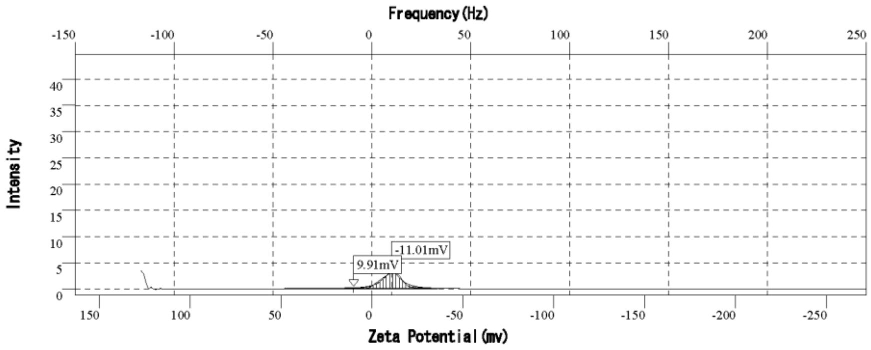

Data #5

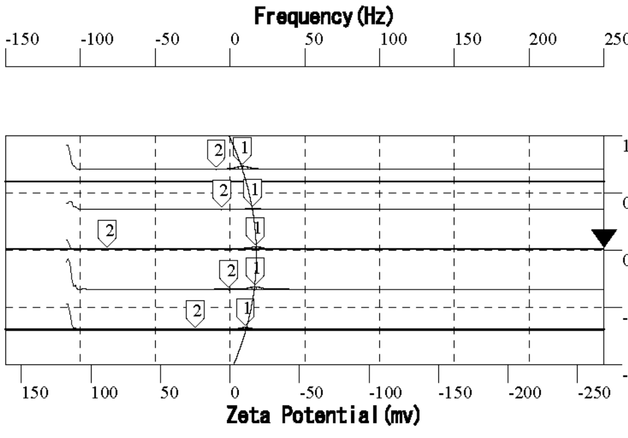

Zeta potential : −11.51 (mV)

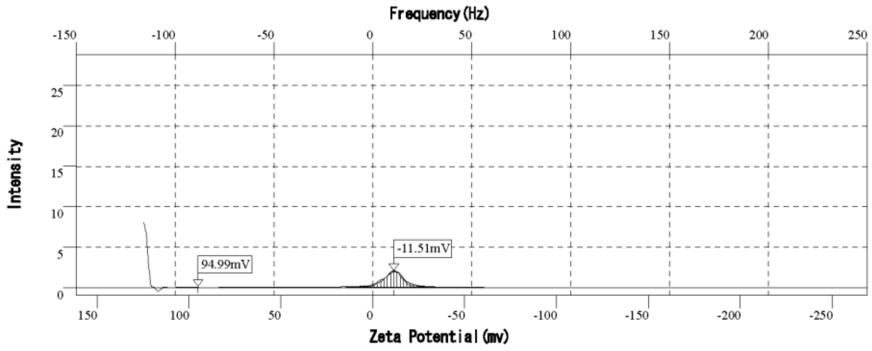

Zeta Potential of monomethyl norbornadiene Na (**1b'**) 100 mM

Electro-osmosis plot: Mobility distribution inside cell

Data #1

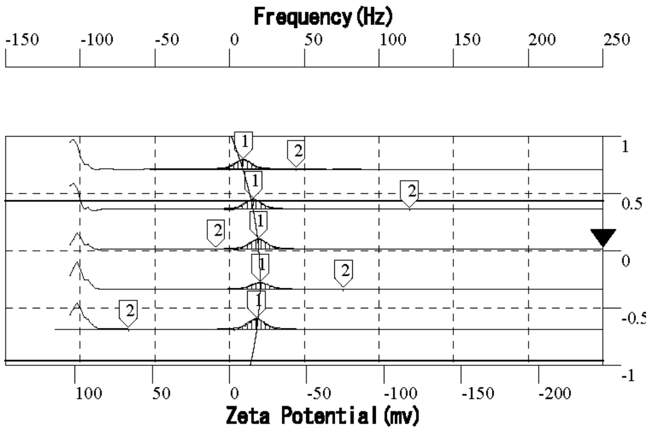

Zeta potential : −14.08 (mV)

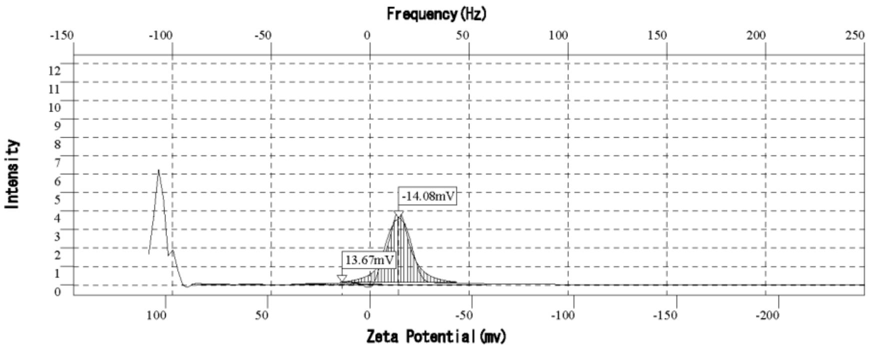

Data #2

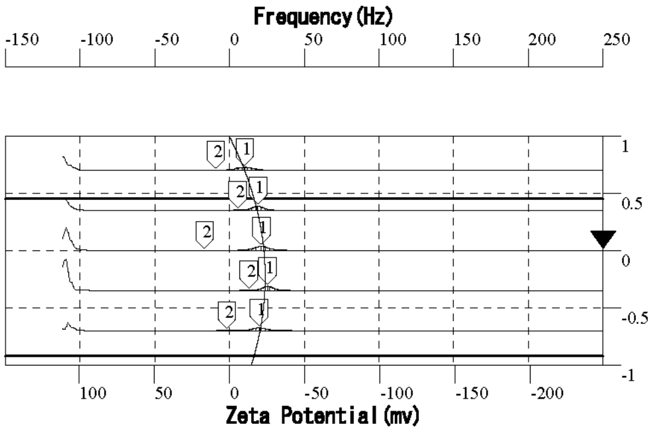

Zeta potential : −16.33 (mV)

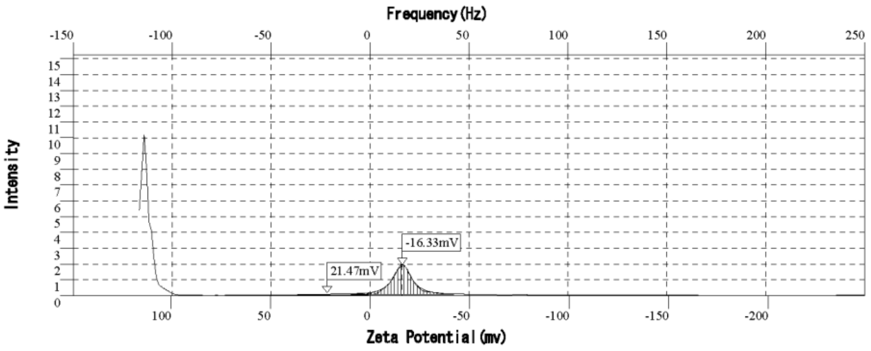

Data #3

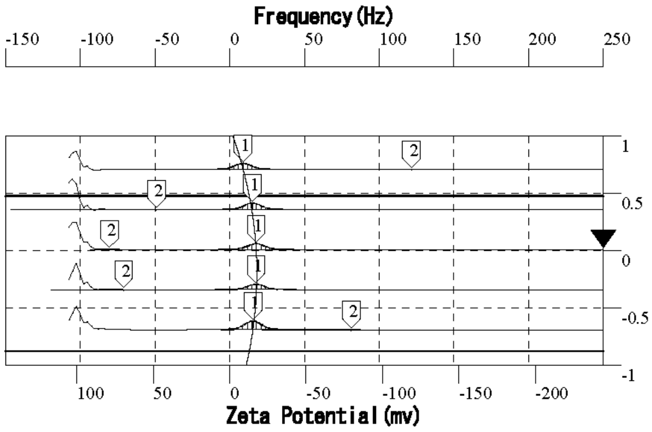

Zeta potential : −12.82 (mV)

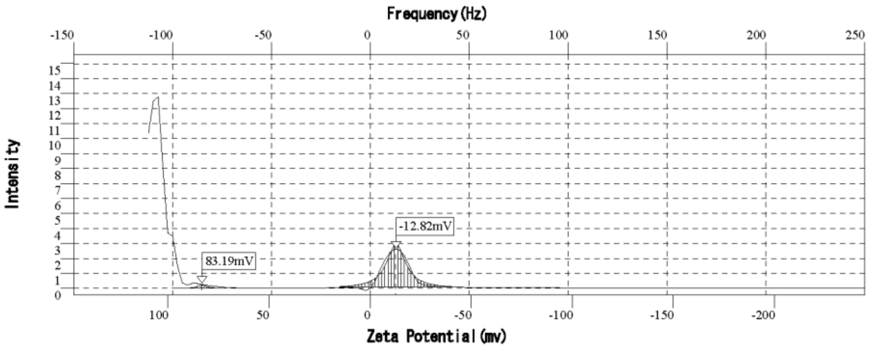

Data #4

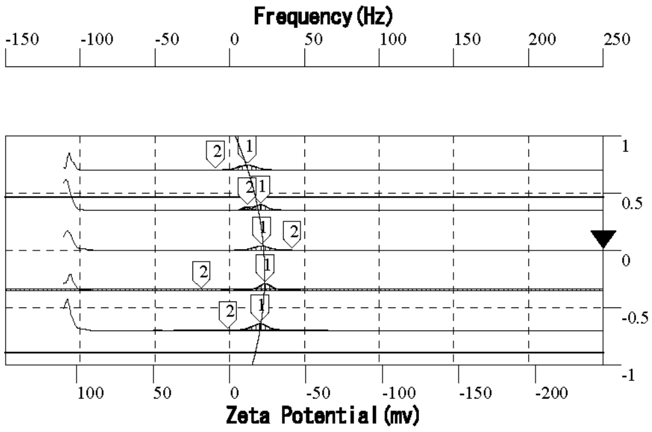

Zeta potential : −16.87 (mV)

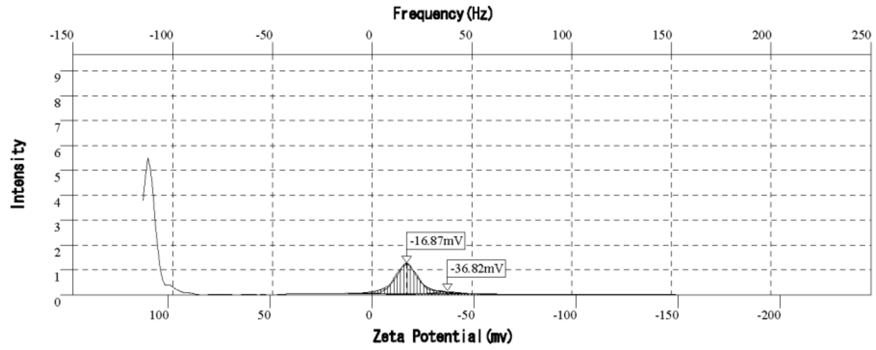

Data #5

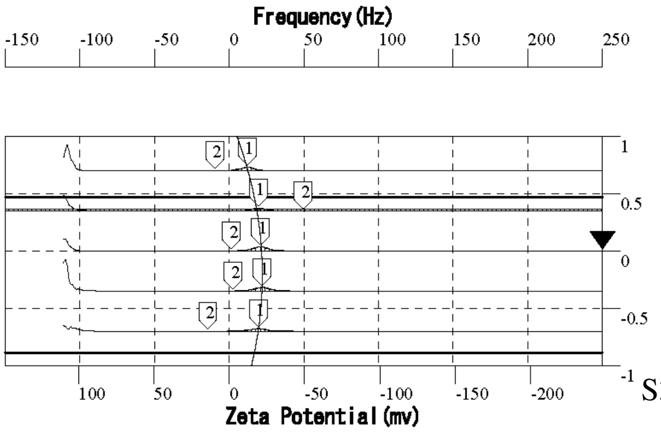

Zeta potential : −16.74 (mV)

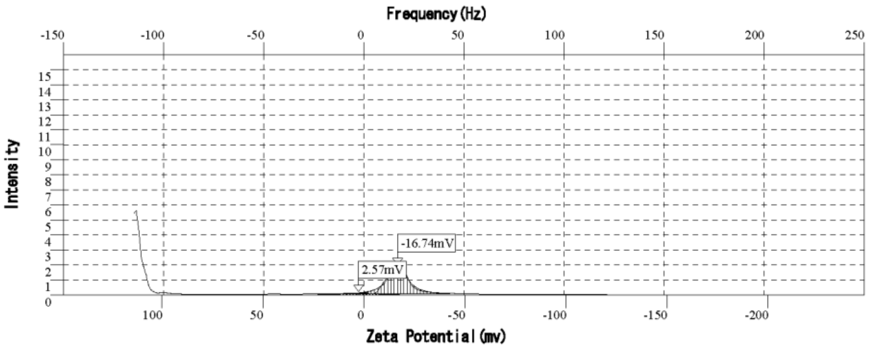

Zeta Potential of monomethyl norbornadiene Na (**1b'**) 50 mM

Electro-osmosis plot: Mobility distribution inside cell

Data #1

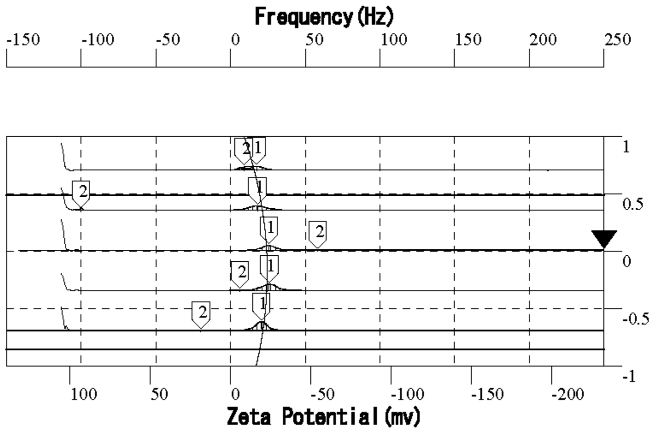

Zeta potential : −18.25 (mV)

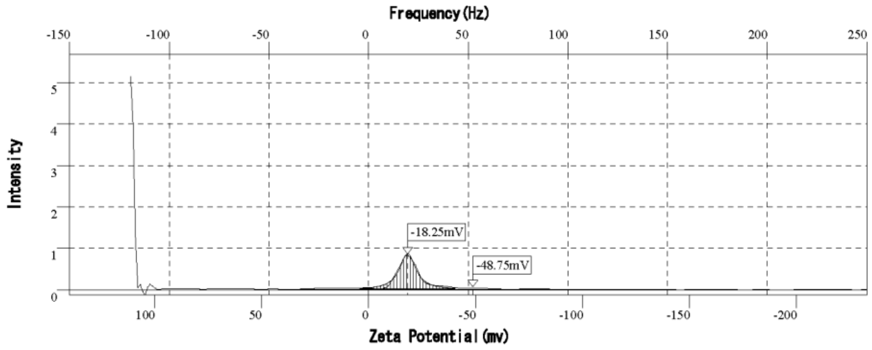

Data #2

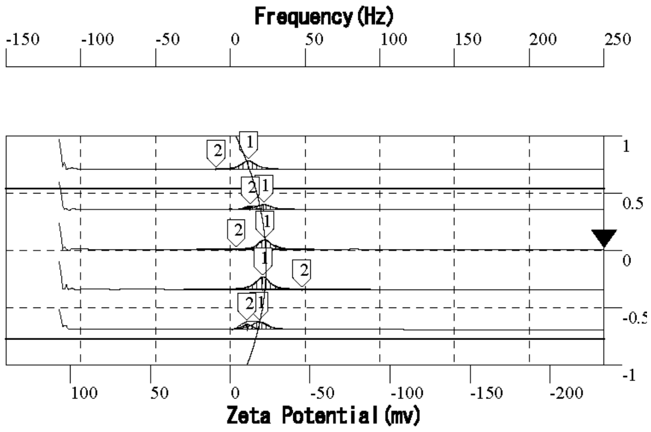

Zeta potential : −15.88 (mV)

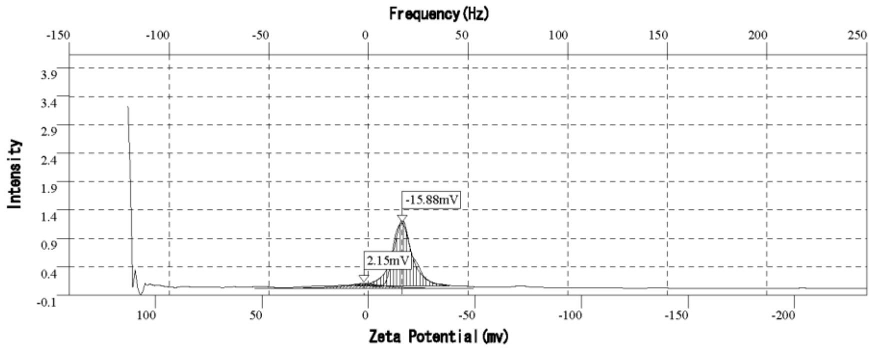

Data #3

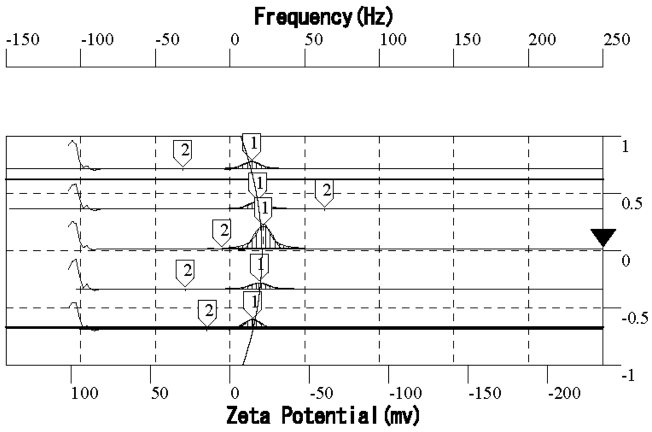

Zeta potential : −14.84 (mV)

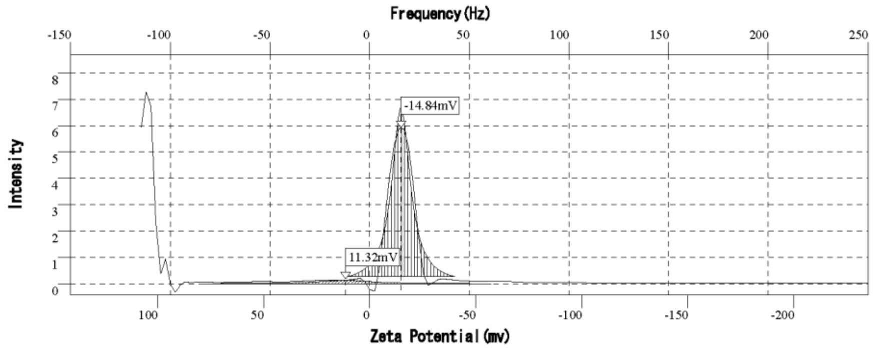

Data #4

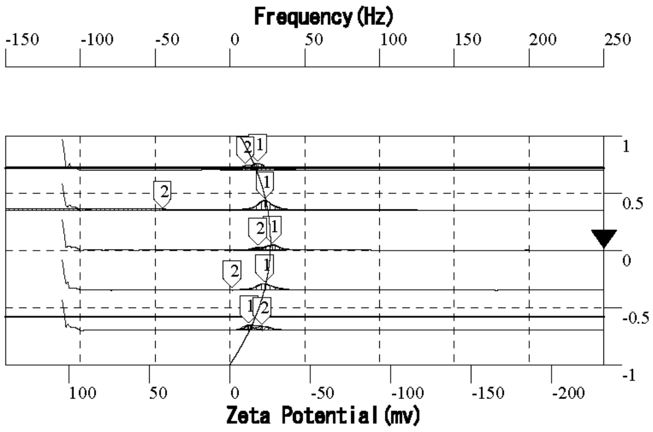

Zeta potential : −15.94 (mV)

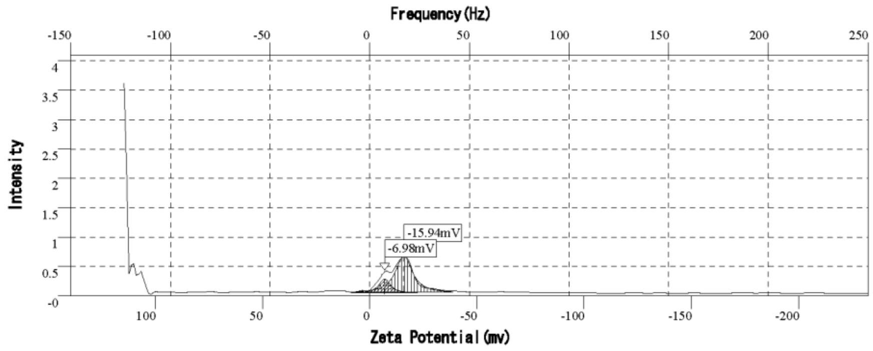

Data #5

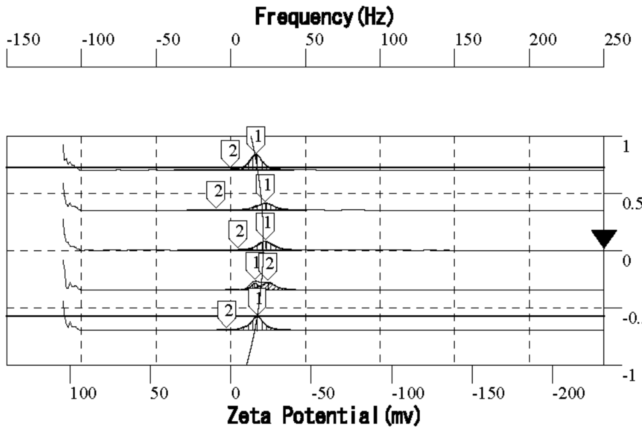

Zeta potential : −16.50 (mV)

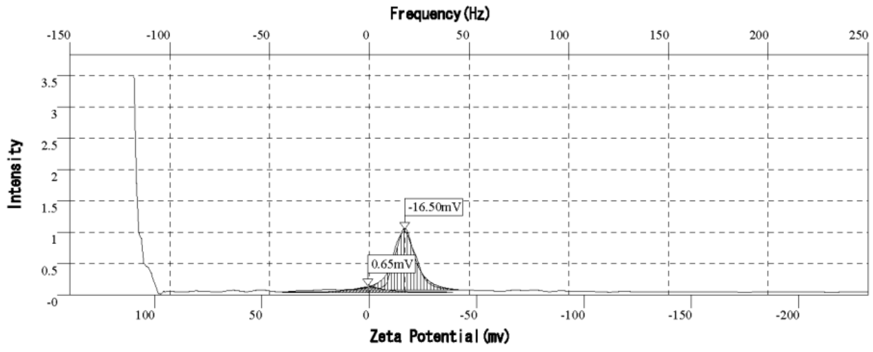

Zeta Potential of monomethyl norbornadiene Na (**1b'**) 20 mM

Electro-osmosis plot: Mobility distribution inside cell

Data #1

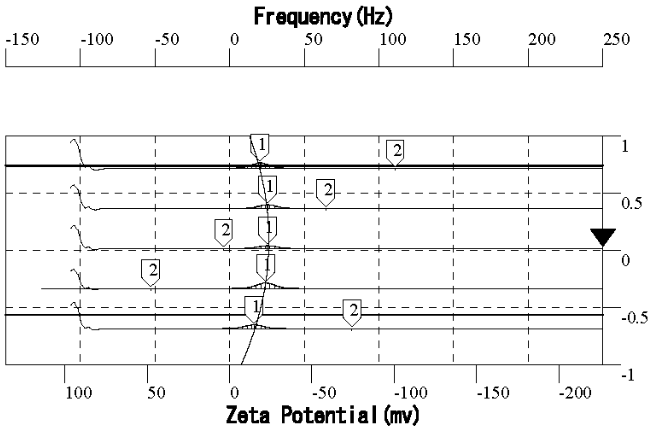

Zeta potential : −17.83 (mV)

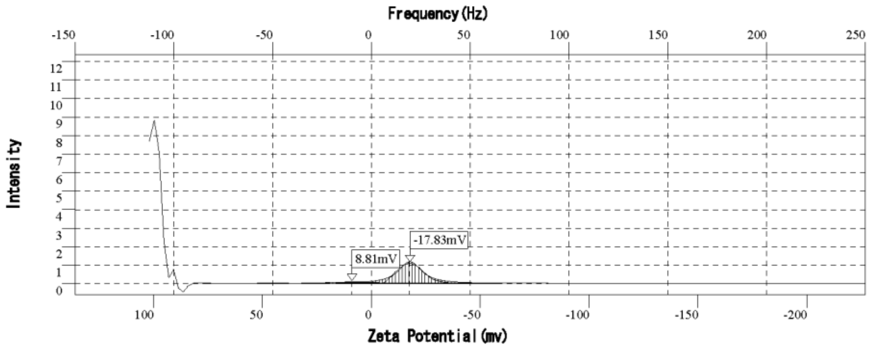

Data #2

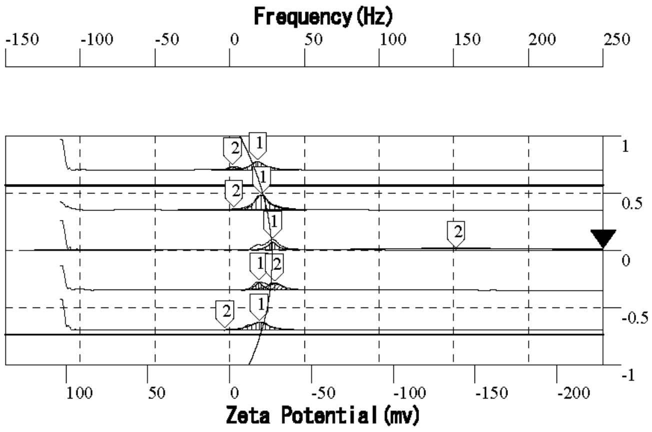

Zeta potential : −18.86 (mV)

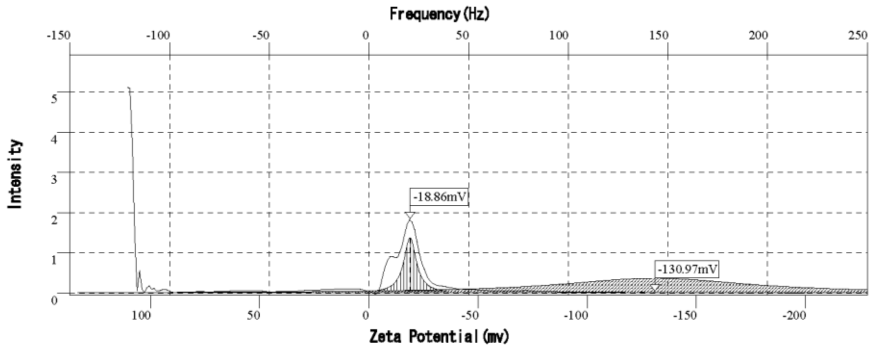

Data #3

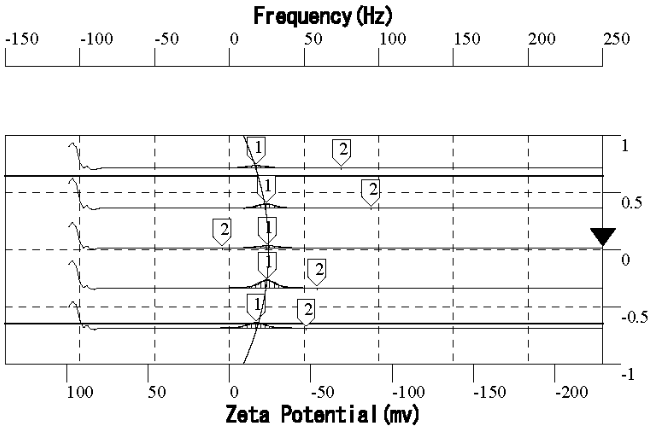

Zeta potential : −17.75 (mV)

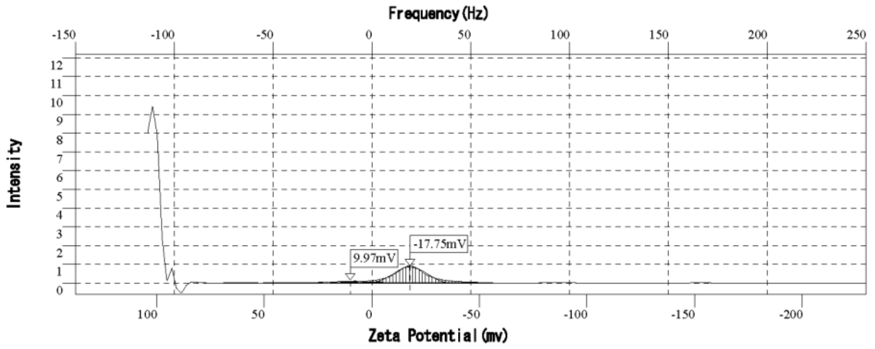

Data #4

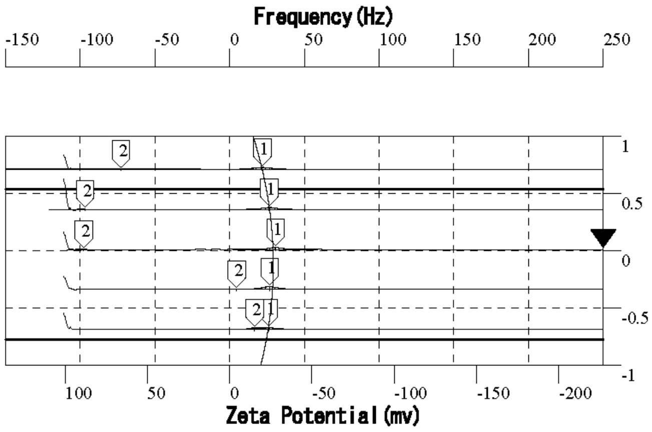

Zeta potential : −22.34 (mV)

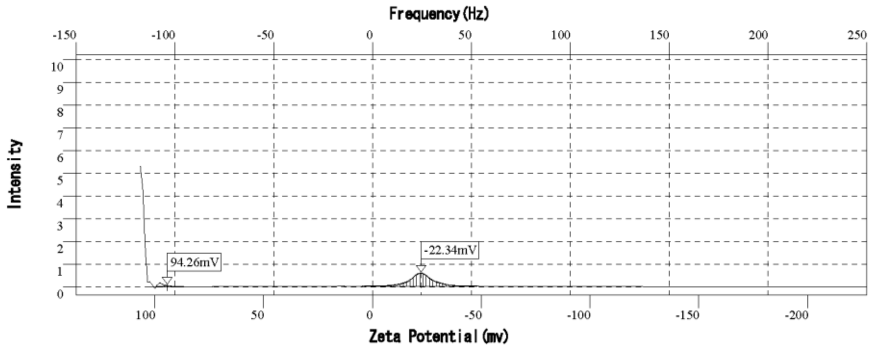

Data #5

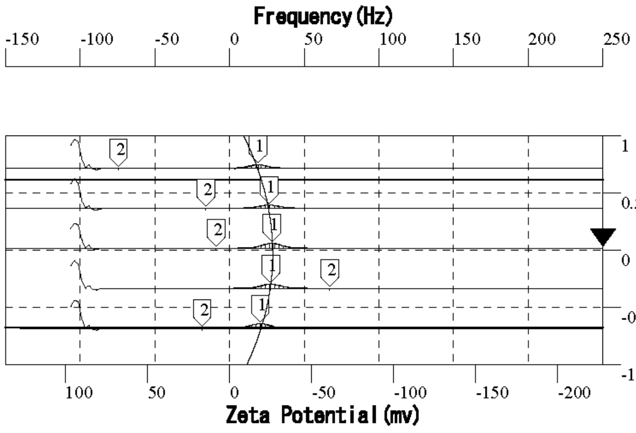

Zeta potential : −19.36 (mV)

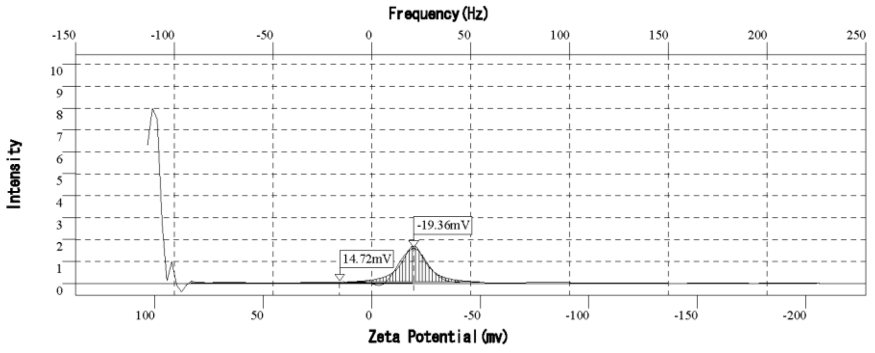

Zeta Potential of monomethyl norbornadiene Na (**1b'**) 10 mM

Electro-osmosis plot: Mobility distribution inside cell

Data #1

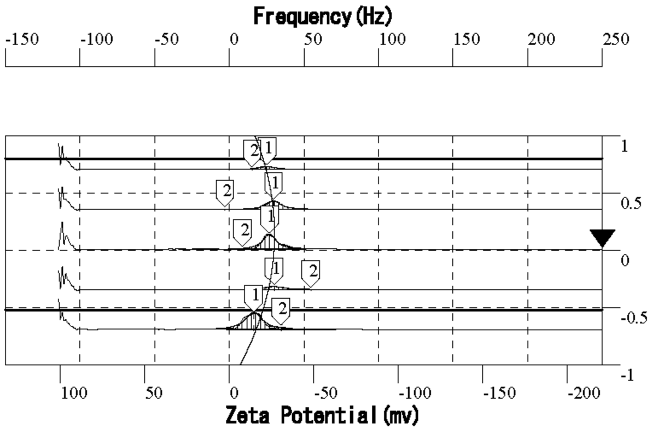

Zeta potential : −19.97 (mV)

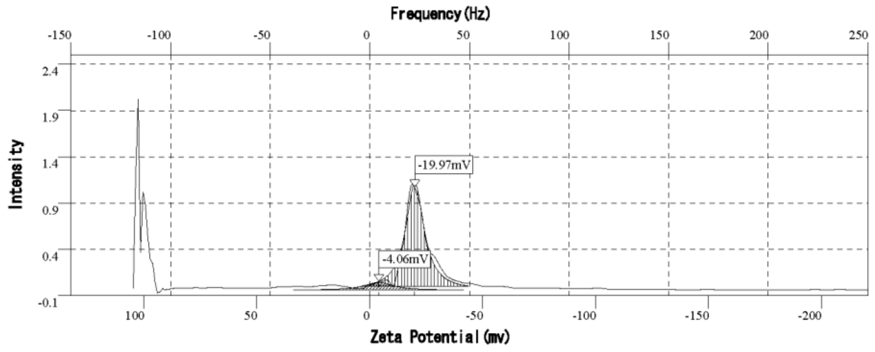

Data #2

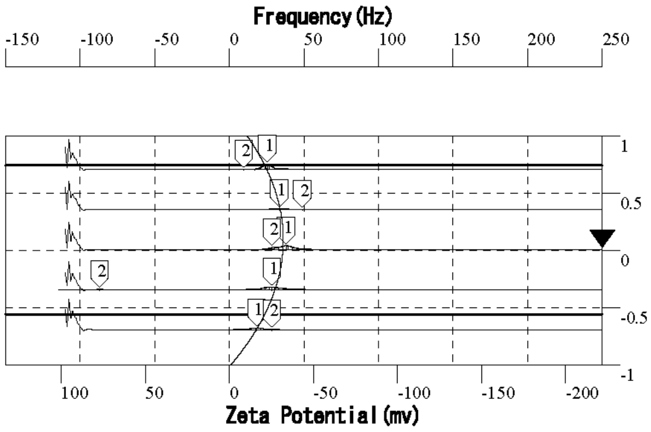

Zeta potential : −21.03 (mV)

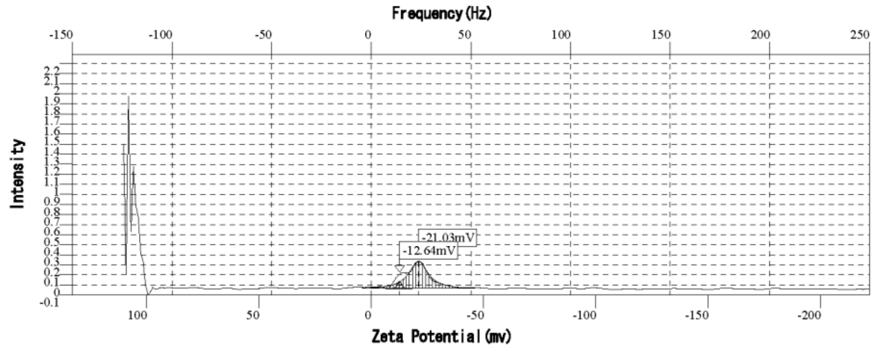

Data #3

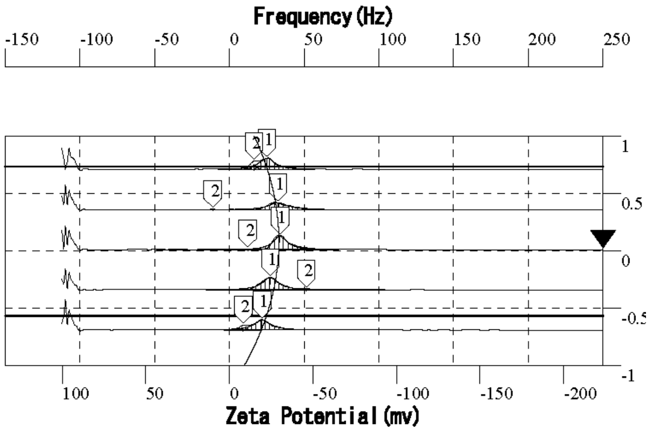

Zeta potential : −22.20 (mV)

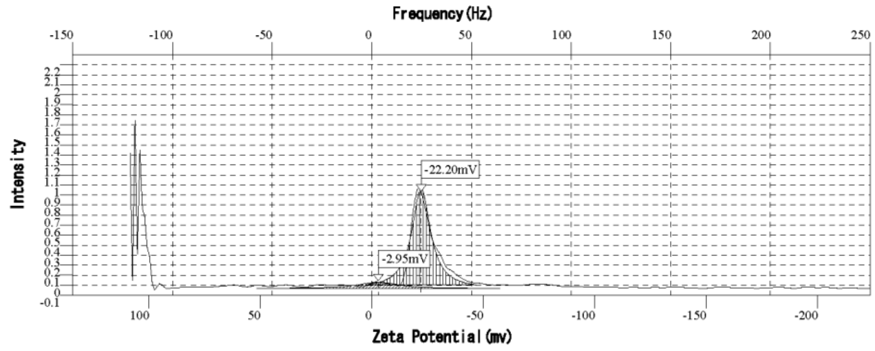

Data #4

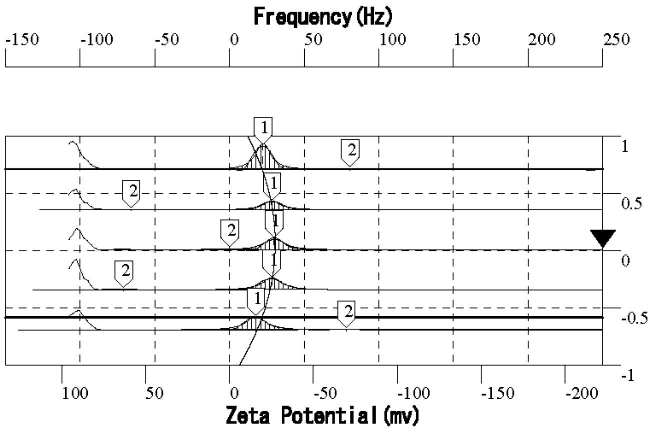

Zeta potential : −19.39 (mV)

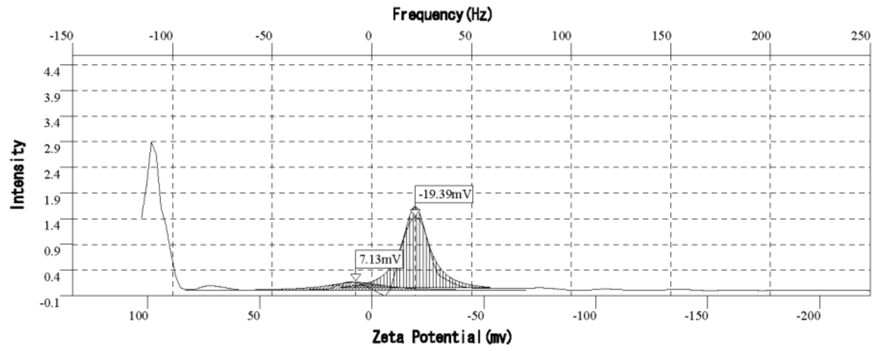

Data #5

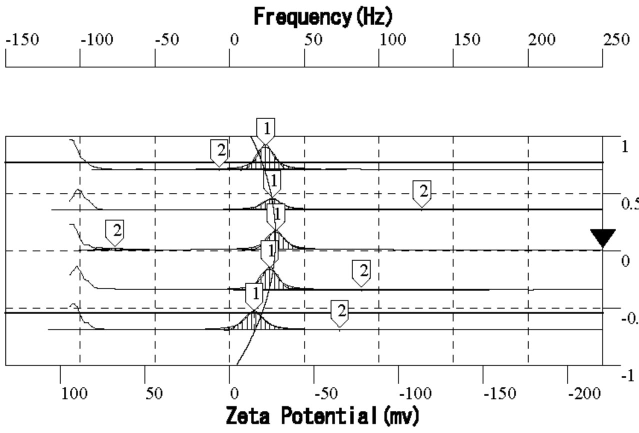

Zeta potential : −19.27 (mV)

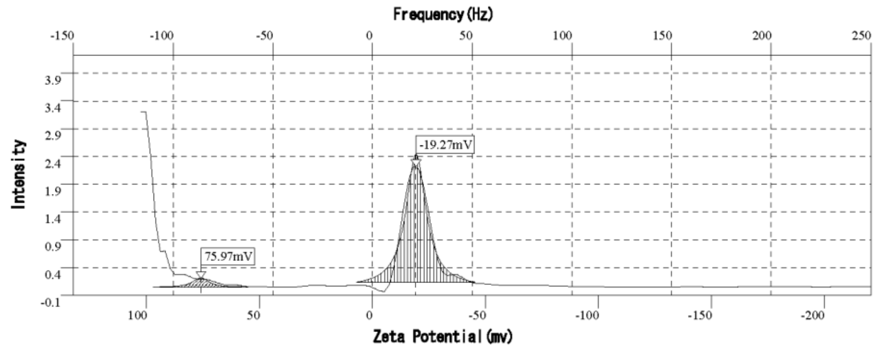

Zeta Potential of monomethyl succinate Na (**2b'**) 250 mM

Electro-osmosis plot: Mobility distribution inside cell

Data #1

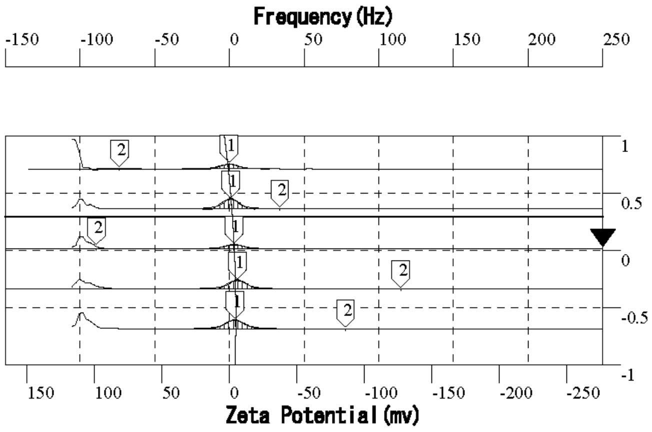

Zeta potential : −2.27 (mV)

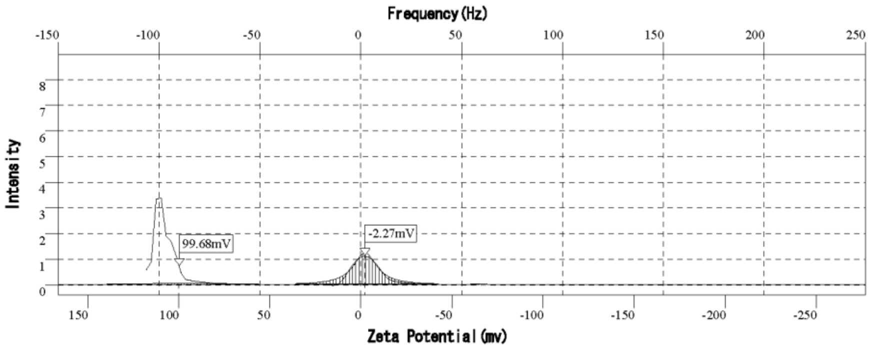

Data #2

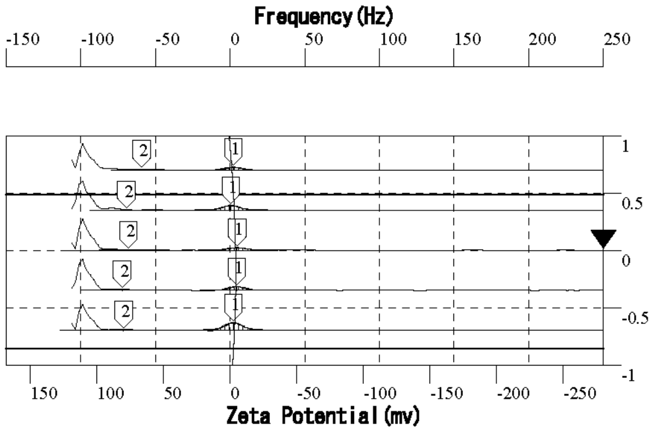

Zeta potential : −2.66 (mV)

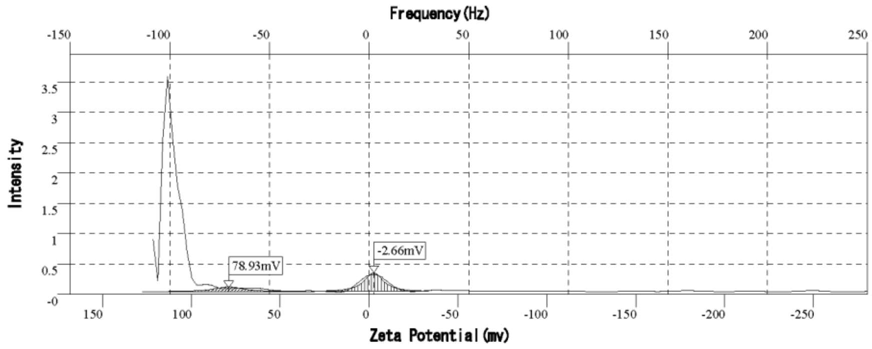

Data #3

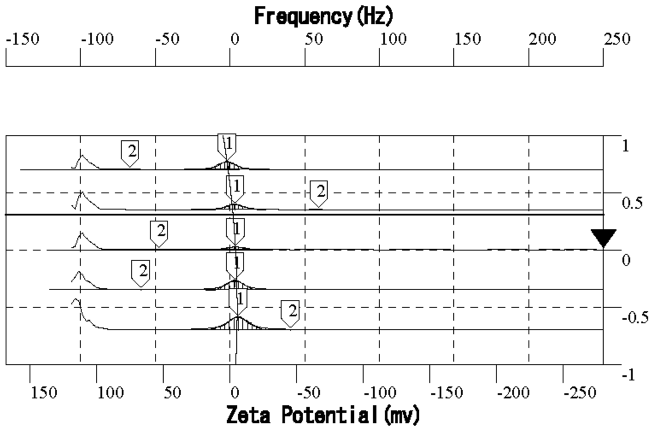

Zeta potential : −2.33 (mV)

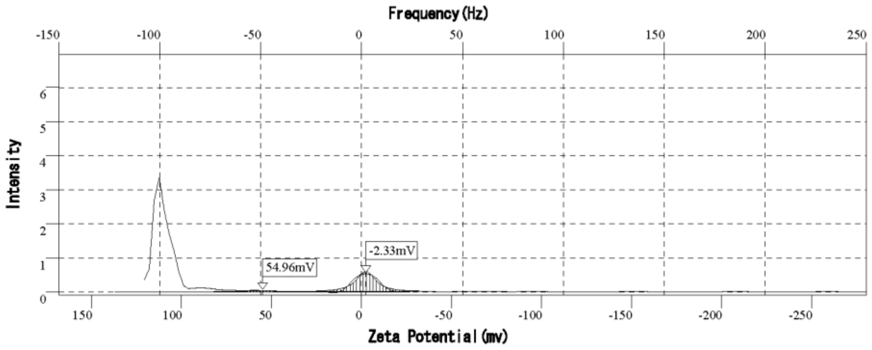

Data #4

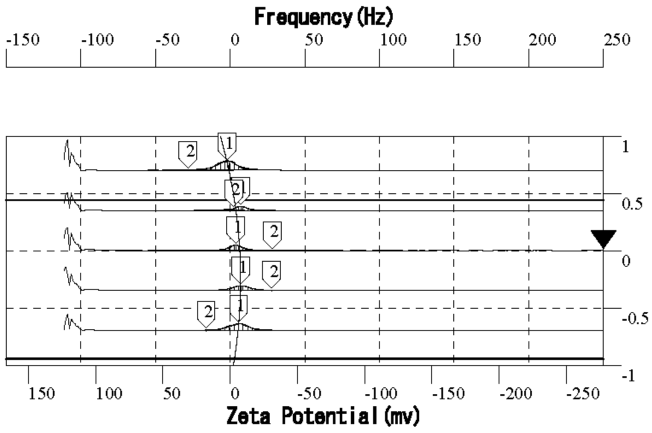

Zeta potential : −3.24 (mV)

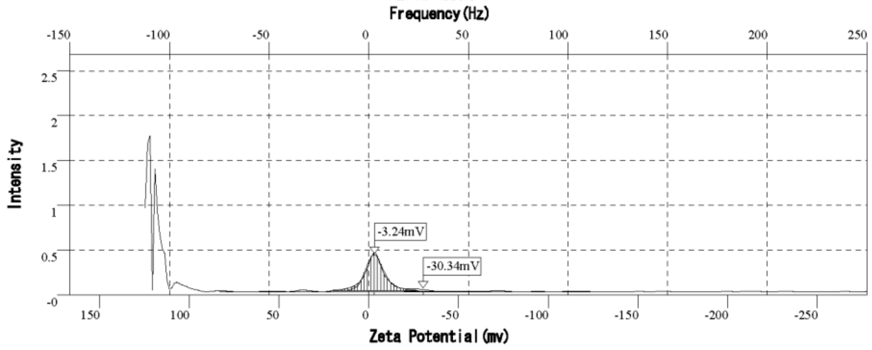

Data #5

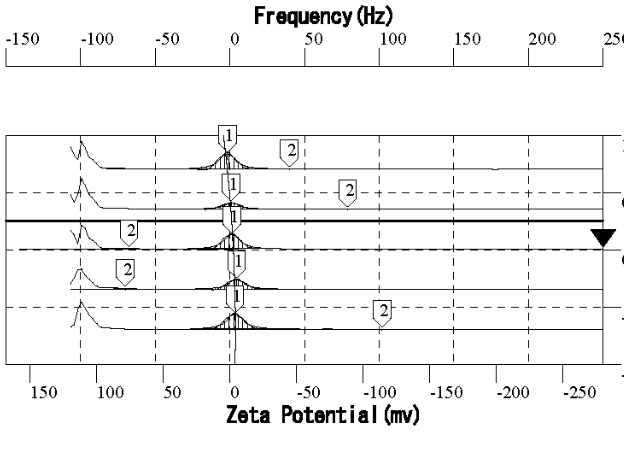

Zeta potential : −1.42 (mV)

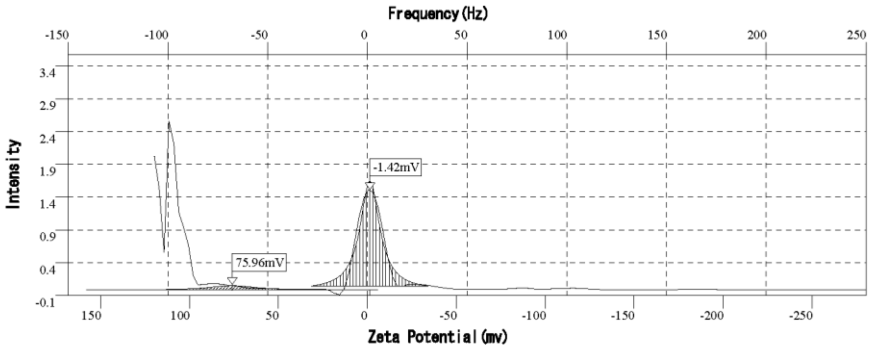

Zeta Potential of monomethyl succinate Na (**2b'**) 100 mM

Electro-osmosis plot: Mobility distribution inside cell

Data #1

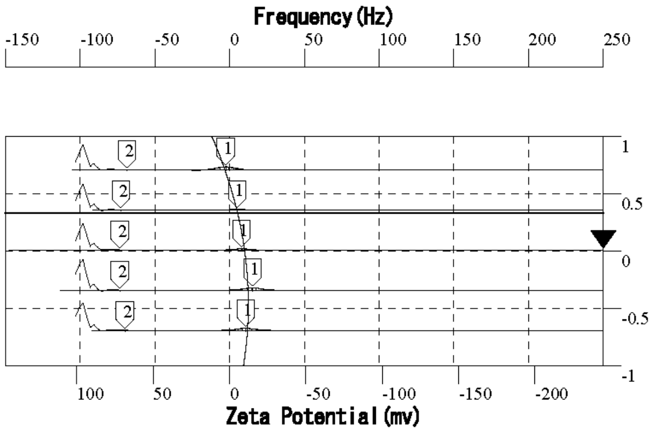

Zeta potential : −5.23 (mV)

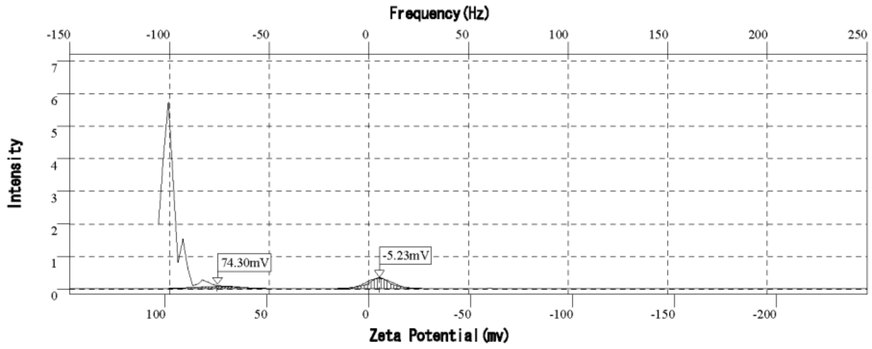

Data #2

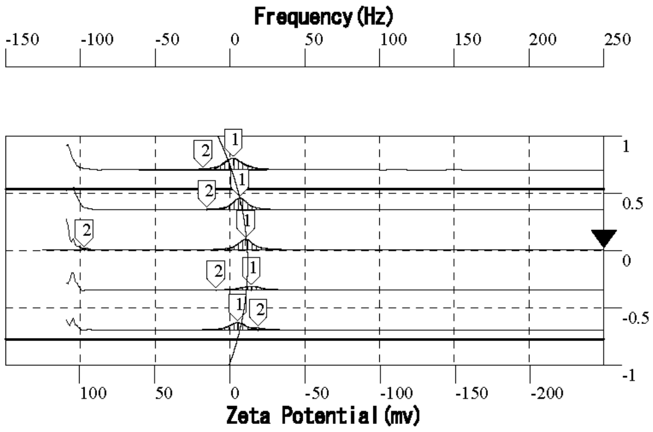

Zeta potential : −4.98 (mV)

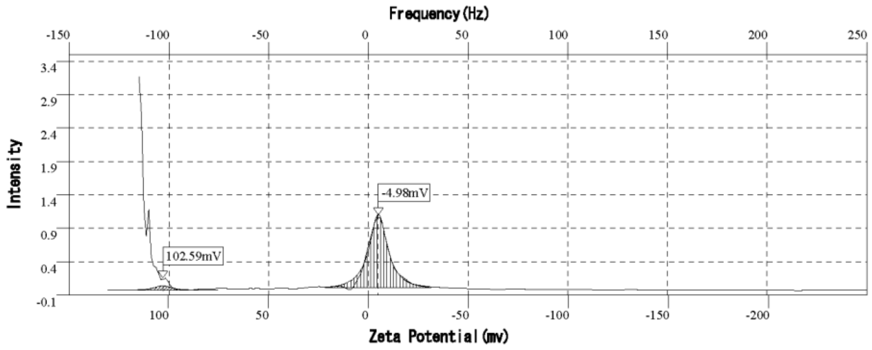

Data #3

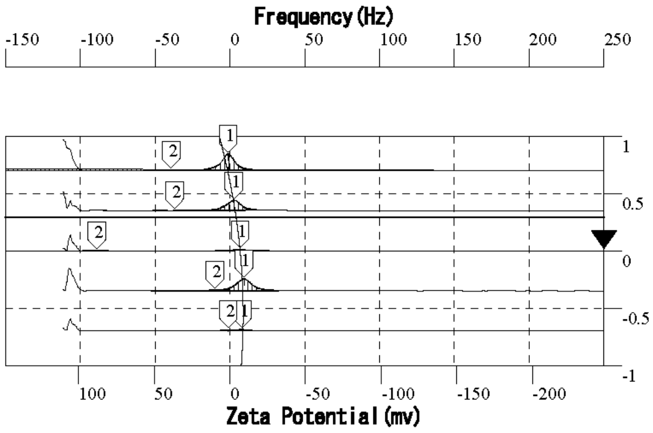

Zeta potential : −4.06 (mV)

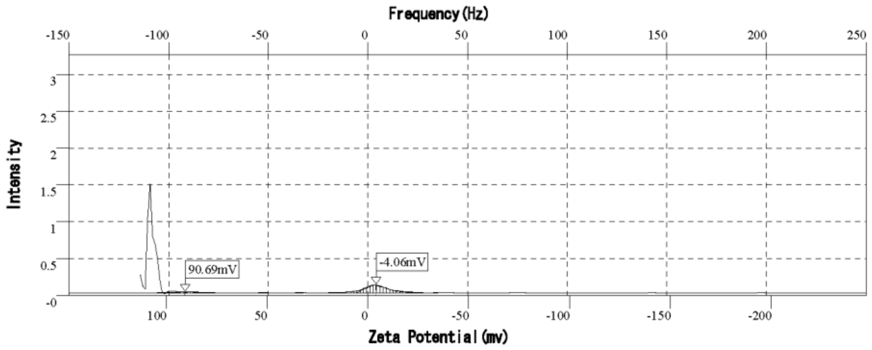

Data #4

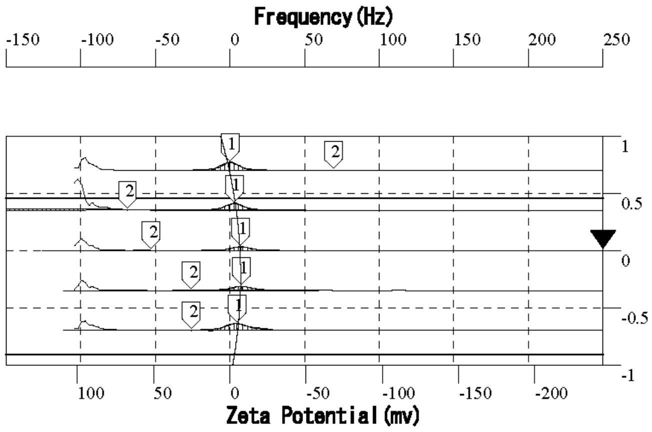

Zeta potential : −2.98 (mV)

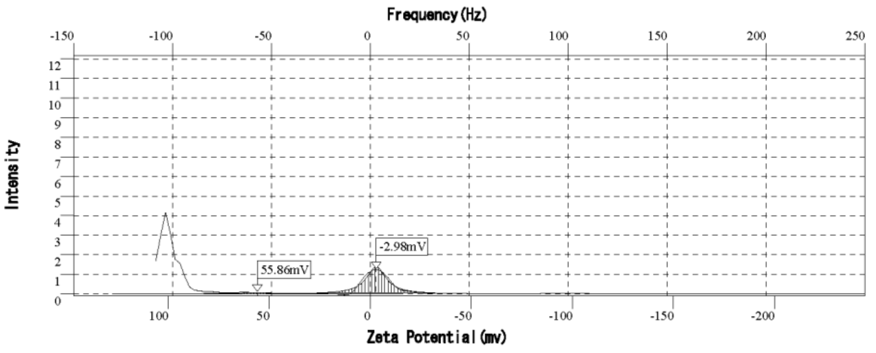

Data #5

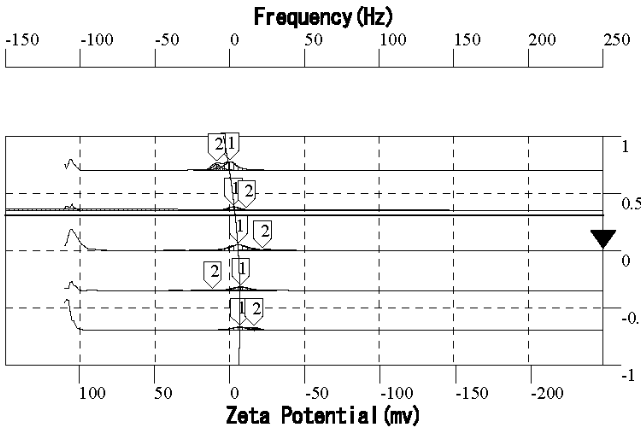

Zeta potential : −3.72 (mV)

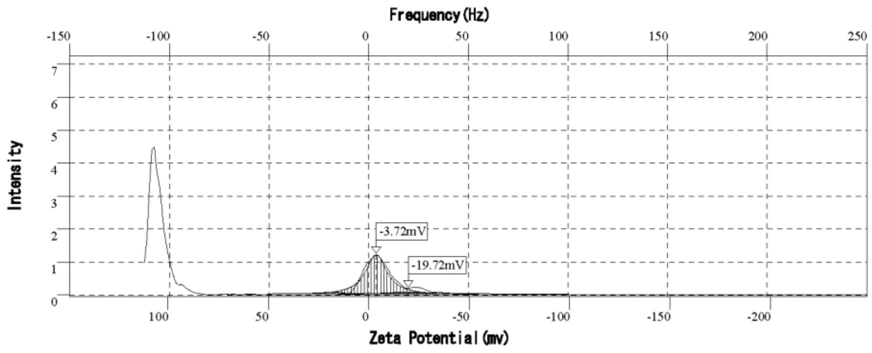

Zeta Potential of monomethyl succinate Na (**2b'**) 50 mM

Electro-osmosis plot: Mobility distribution inside cell

Data #1

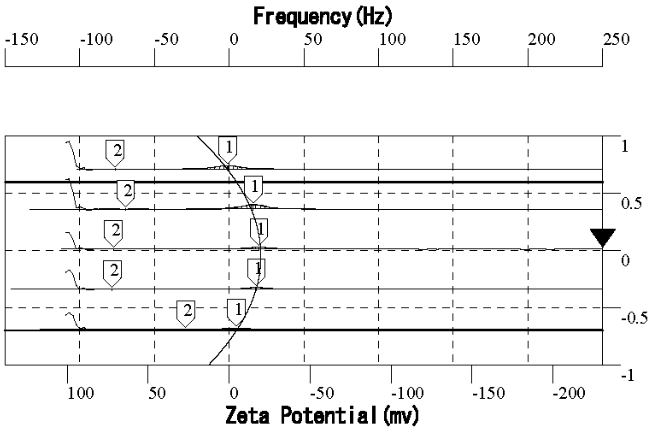

Zeta potential : −4.63 (mV)

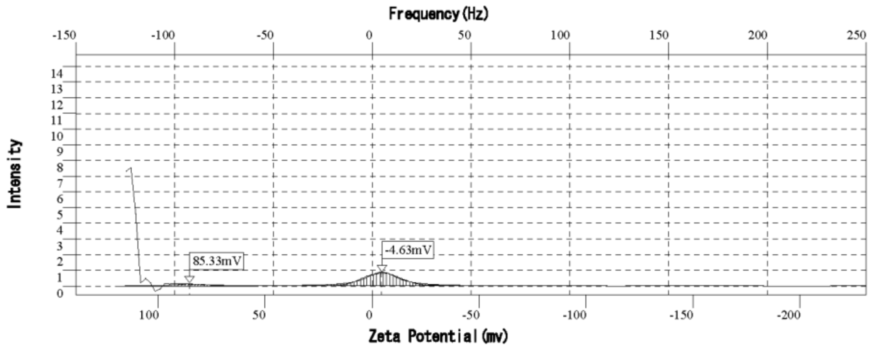

Data #2

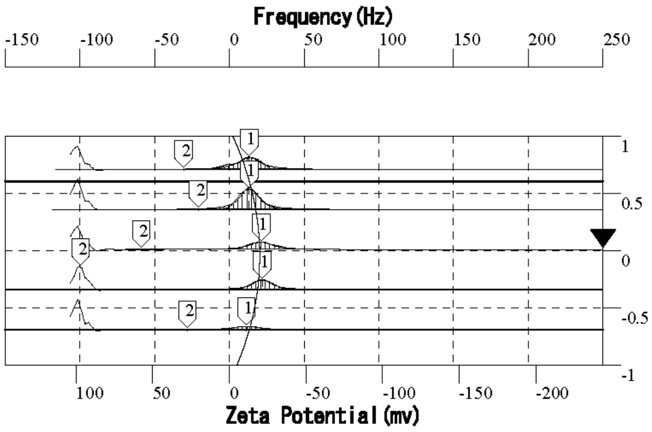

Zeta potential : −13.07 (mV)

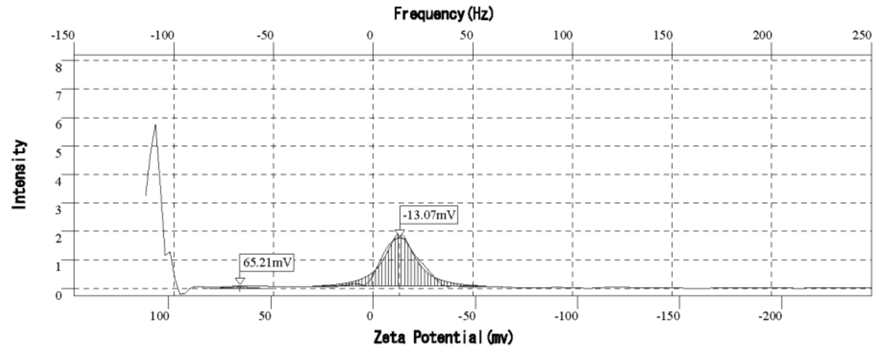

Data #3

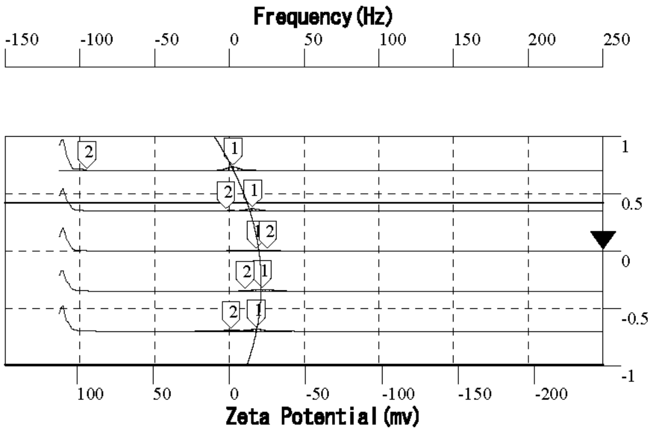

Zeta potential : −11.66 (mV)

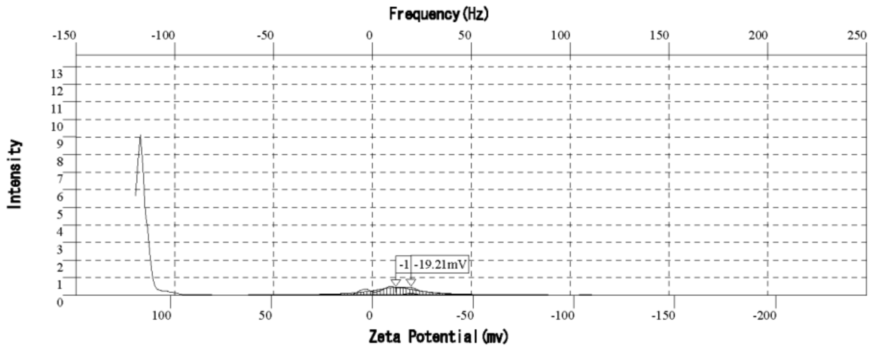

Data #4

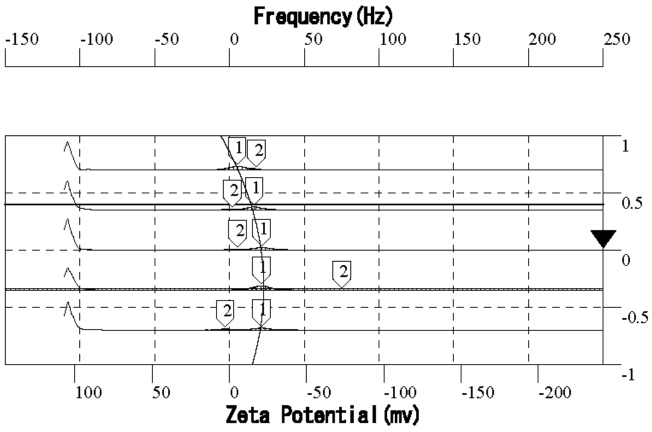

Zeta potential : −13.90 (mV)

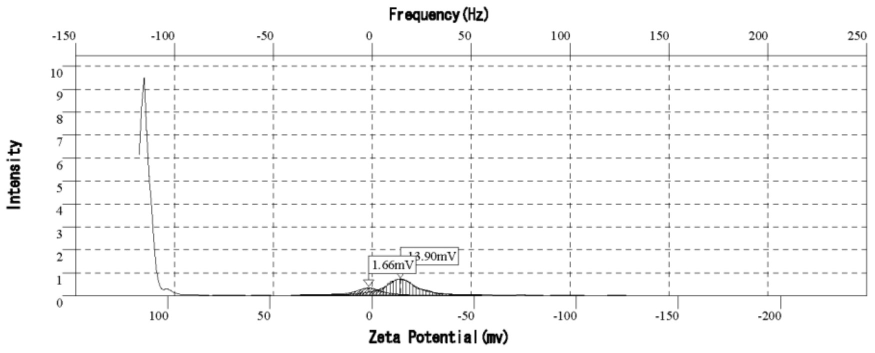

Data #5

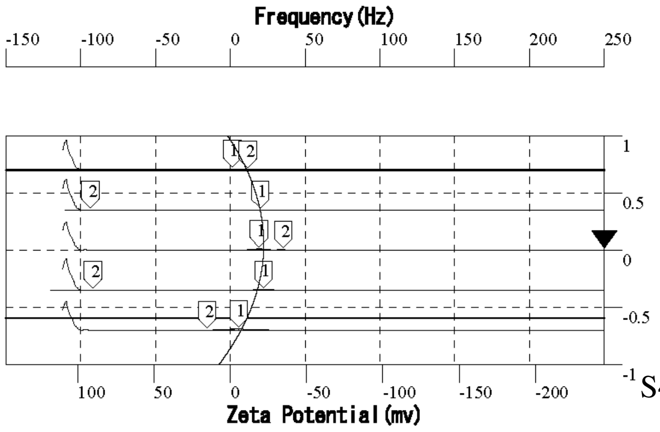

Zeta potential : −10.90 (mV)

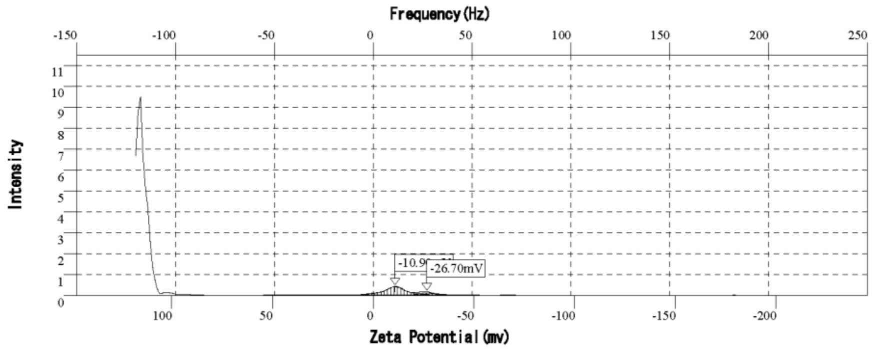

Zeta Potential of monomethyl succinate Na (**2b'**) 20 mM

Electro-osmosis plot: Mobility distribution inside cell

Data #1

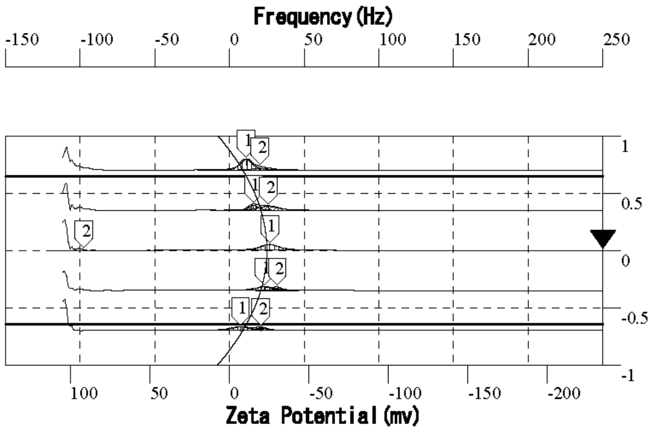

Zeta potential : −10.68 (mV)

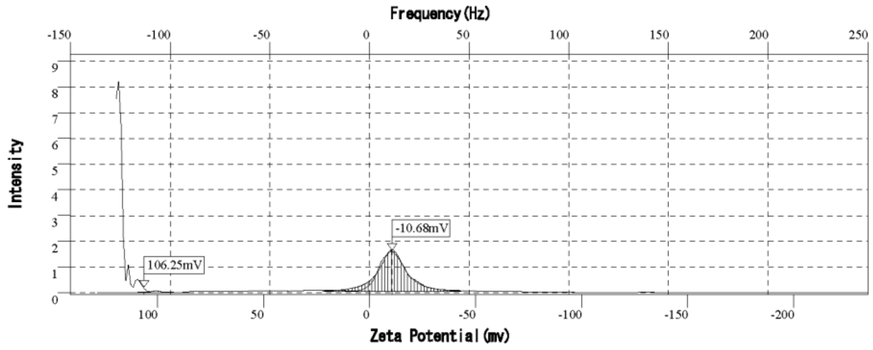

Data #2

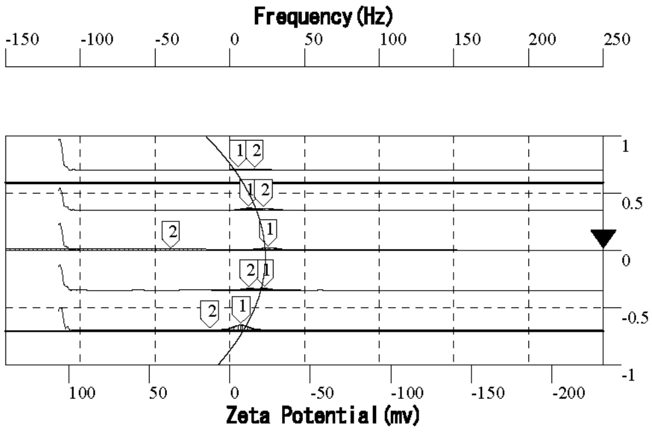

Zeta potential : −8.20 (mV)

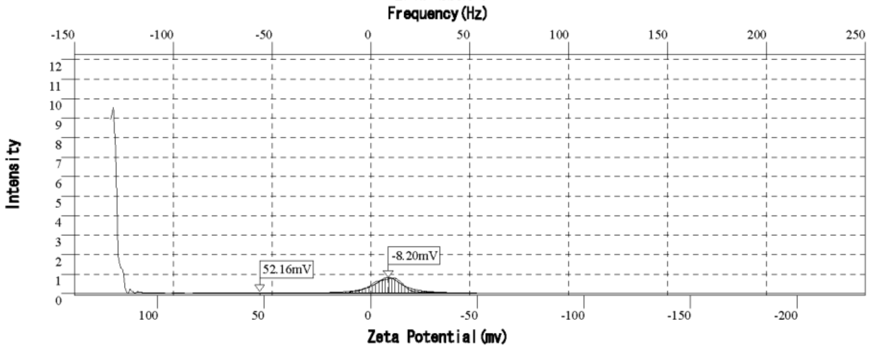

Data #3

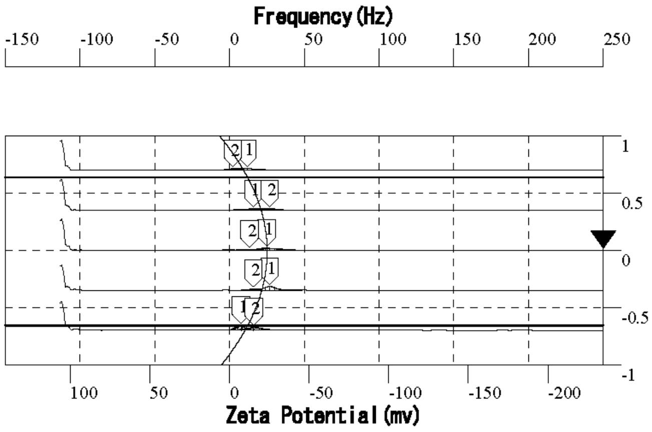

Zeta potential : −11.44 (mV)

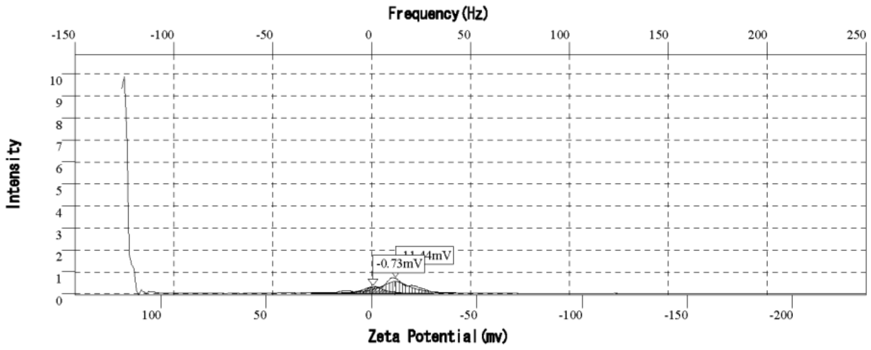

Data #4

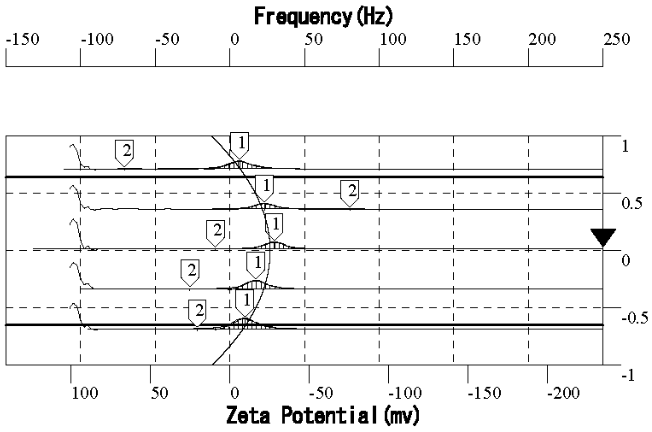

Zeta potential : −10.08 (mV)

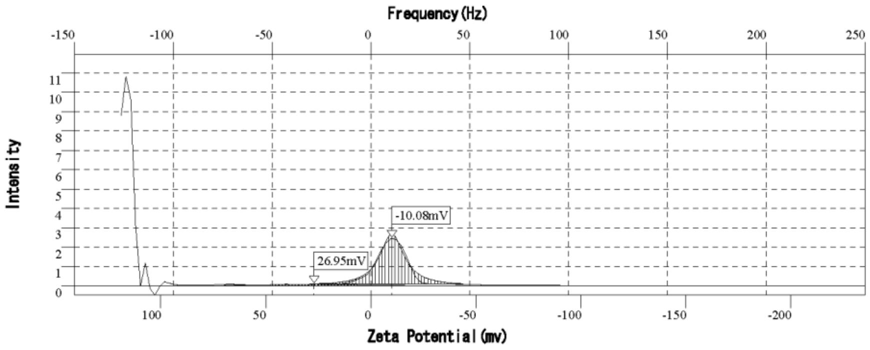

Data #5

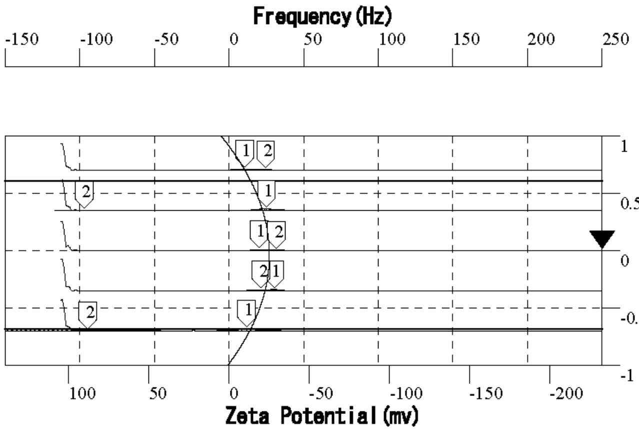

Zeta potential : −13.49 (mV)

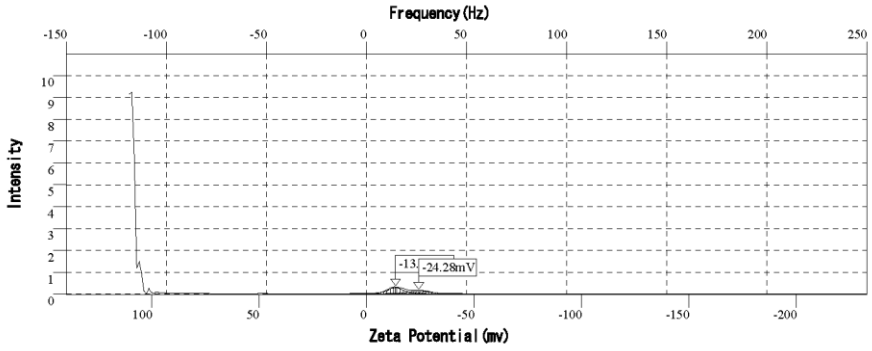

Zeta Potential of monomethyl succinate Na (**2b'**) 10 mM

Electro-osmosis plot: Mobility distribution inside cell

Data #1

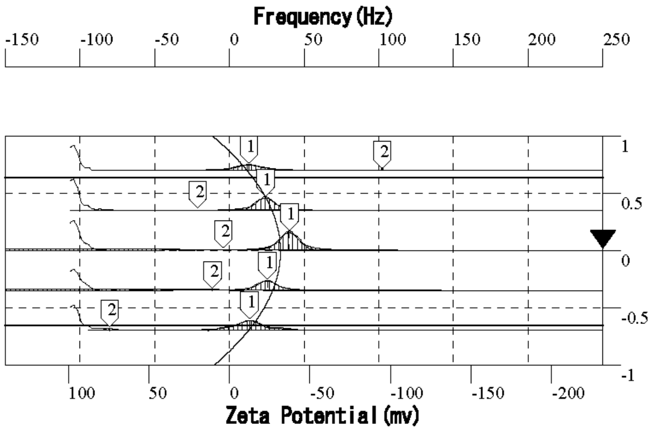

Zeta potential : −14.41 (mV)

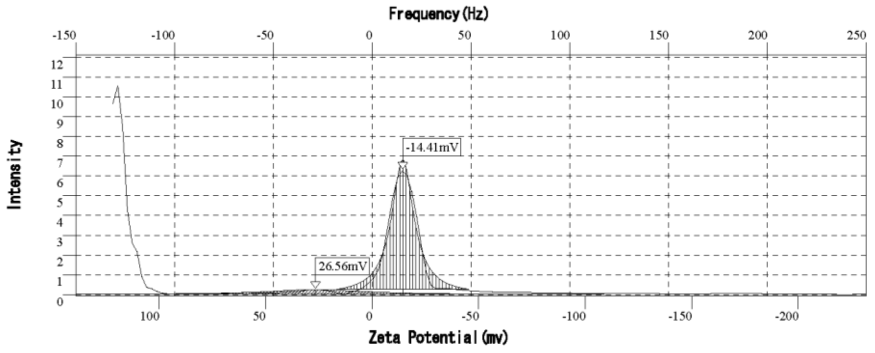

Data #2

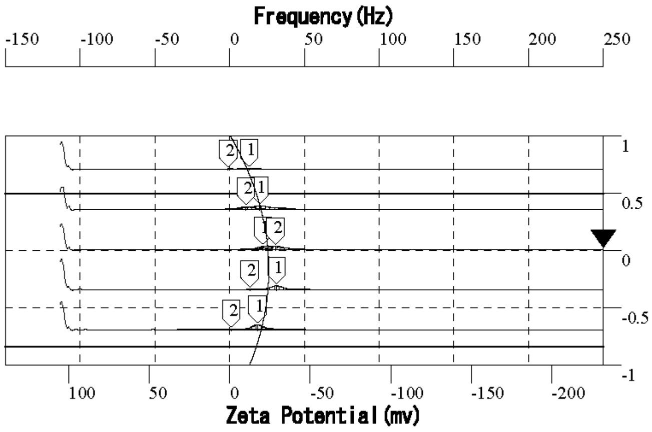

Zeta potential : −16.59 (mV)

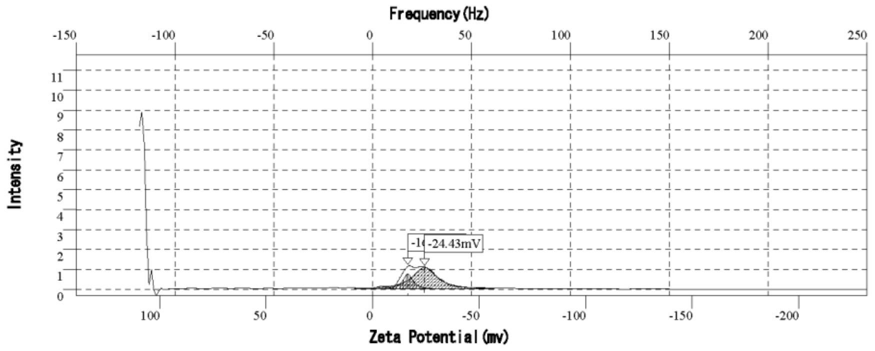

Data #3

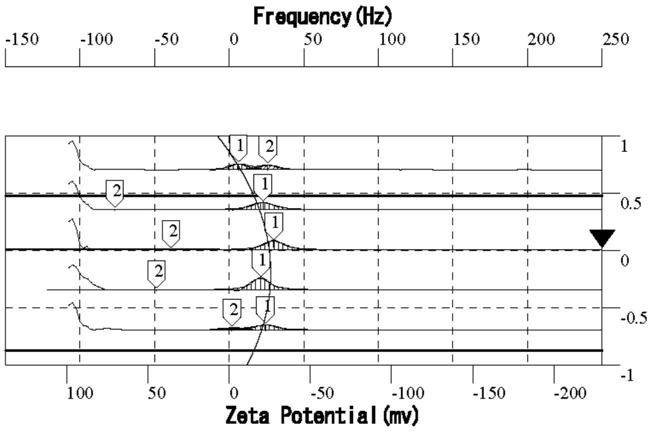

Zeta potential : −15.27 (mV)

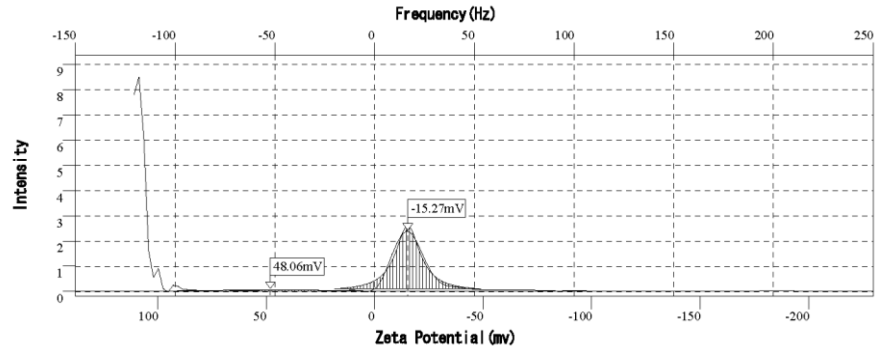

Data #4

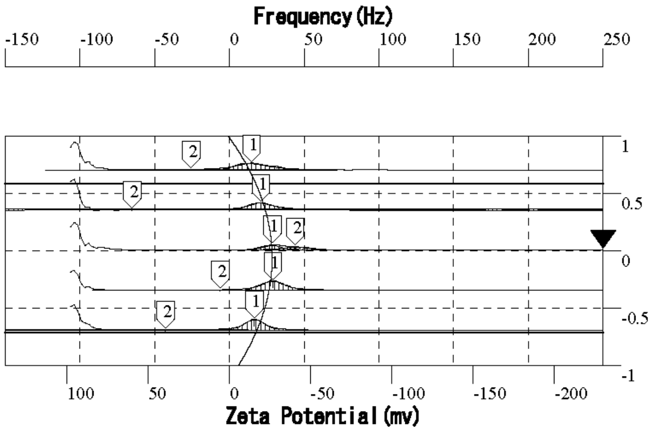

Zeta potential : −16.32 (mV)

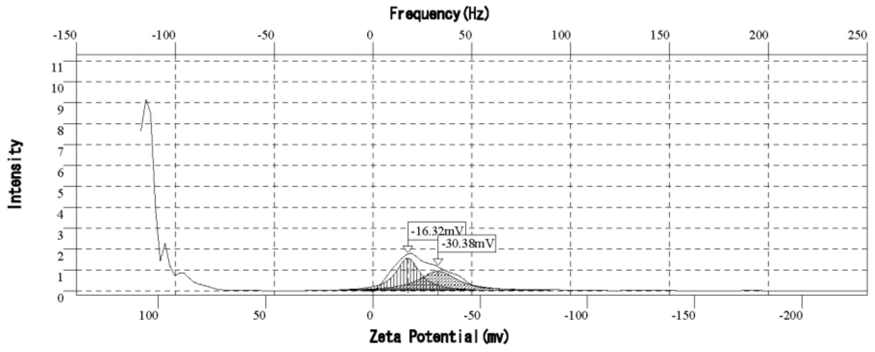

Data #5

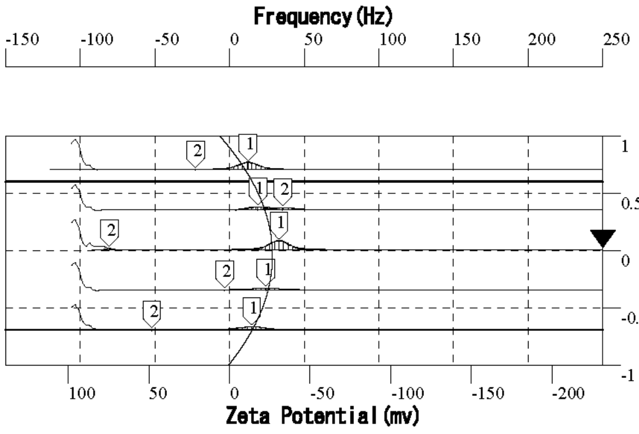

Zeta potential : −14.09 (mV)

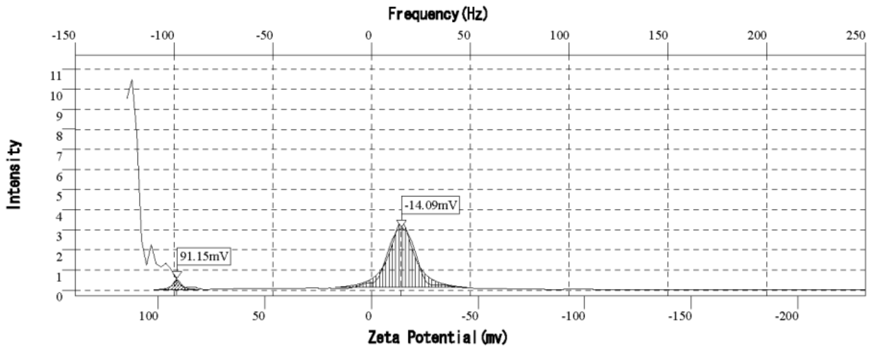

Zeta Potential of monomethyl maleate Na (**3b'**) 250 mM

Electro-osmosis plot: Mobility distribution inside cell

Data #1

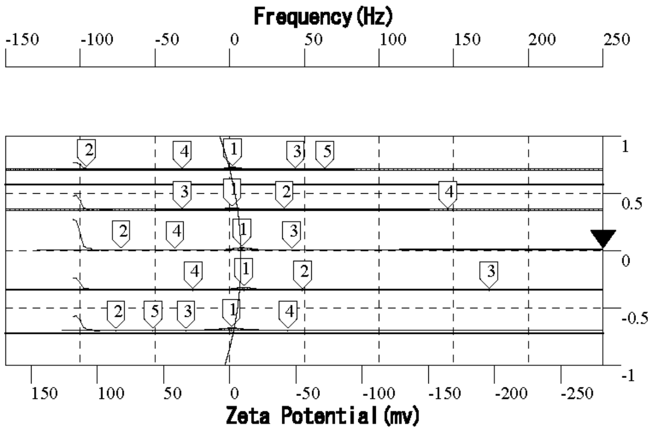

Zeta potential : −2.68 (mV)

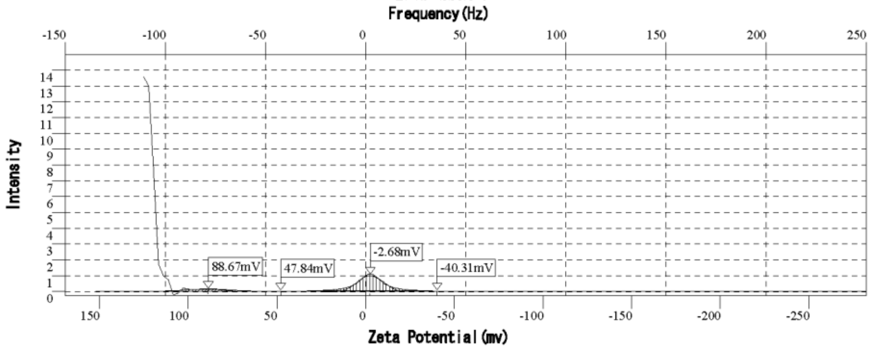

Data #2

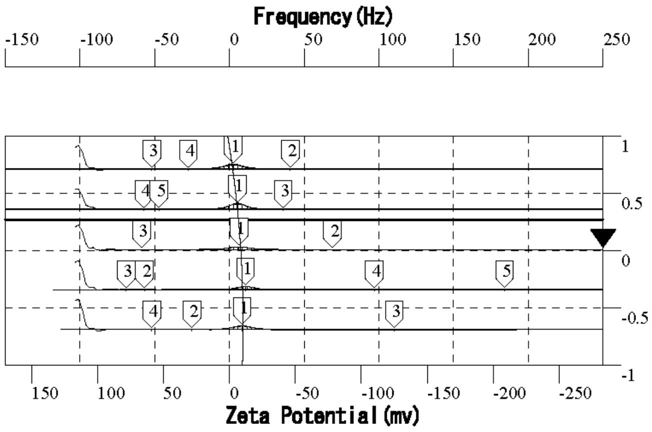

Zeta potential : −6.89 (mV)

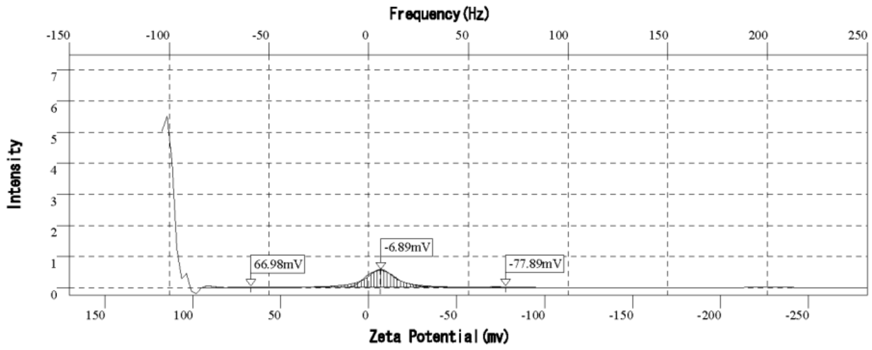

Data #3

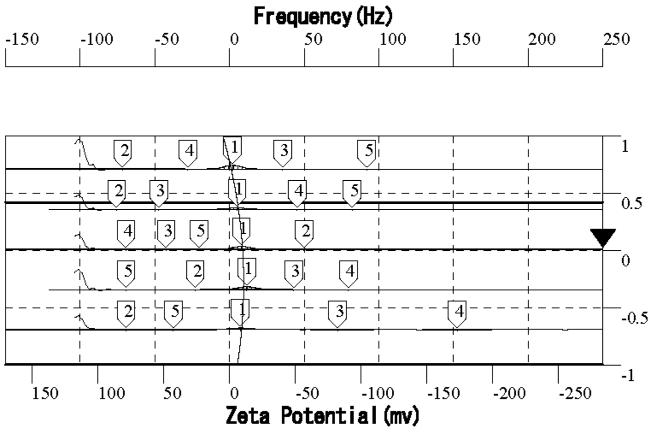

Zeta potential : −6.08 (mV)

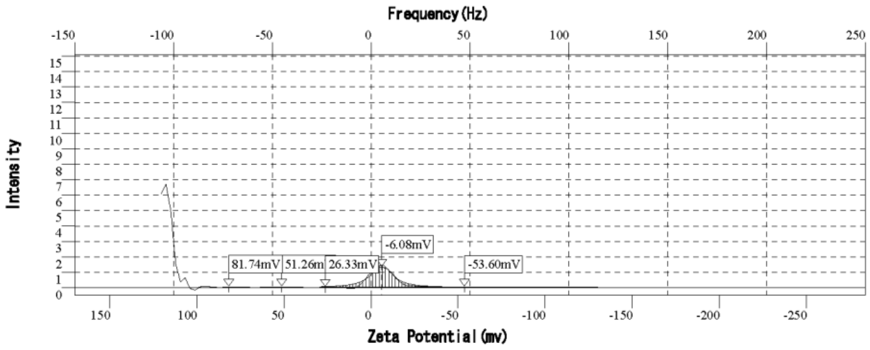

Data #4

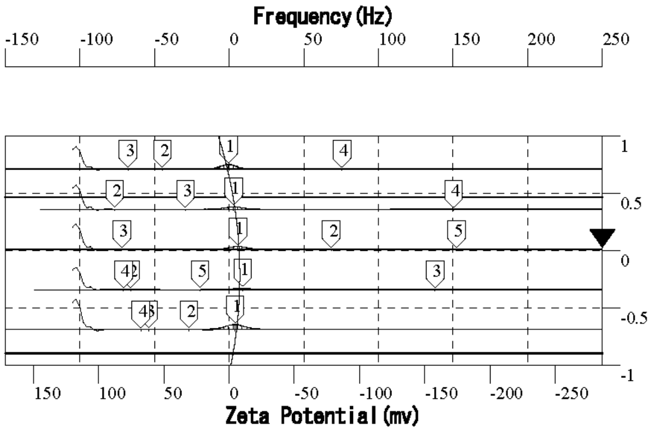

Zeta potential : −3.06 (mV)

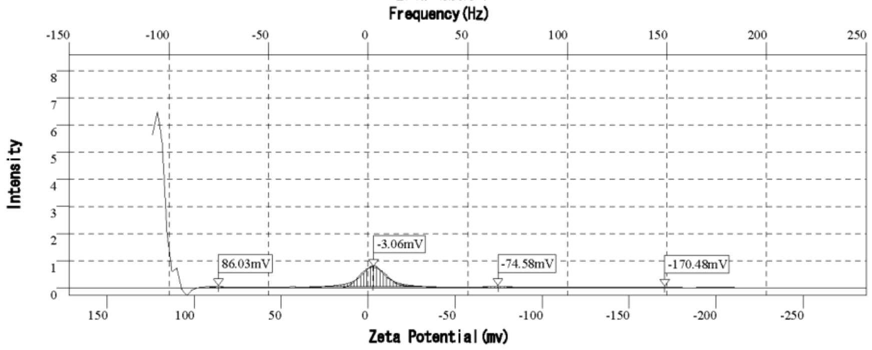

Data #5

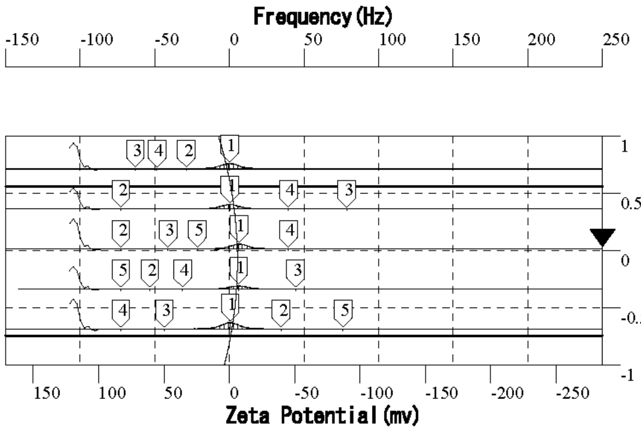

Zeta potential : −1.08 (mV)

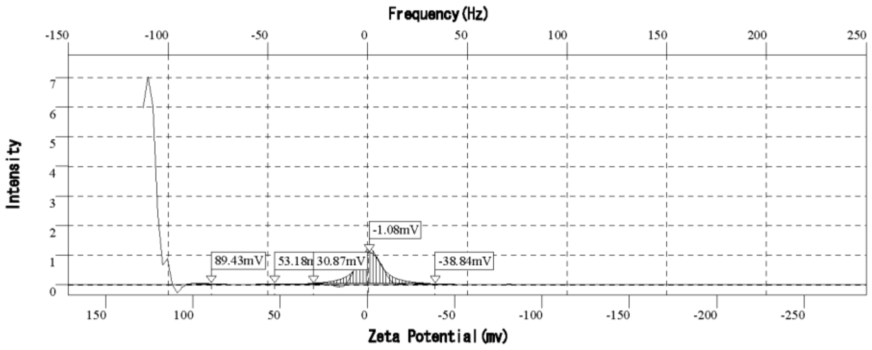

Zeta Potential of monomethyl maleate Na (**3b'**) 100 mM

Electro-osmosis plot: Mobility distribution inside cell

Data #1

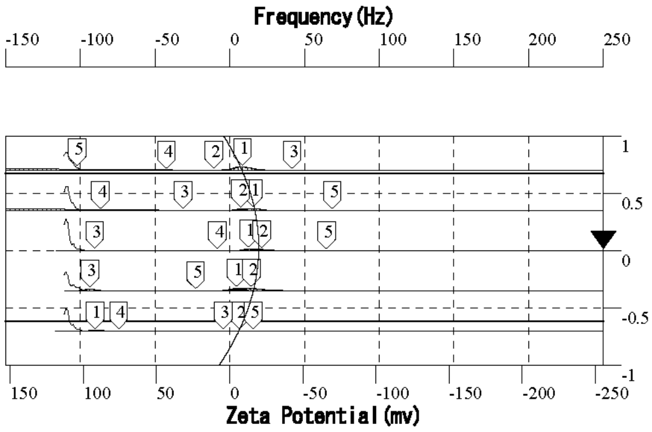

Zeta potential : −9.19 (mV)

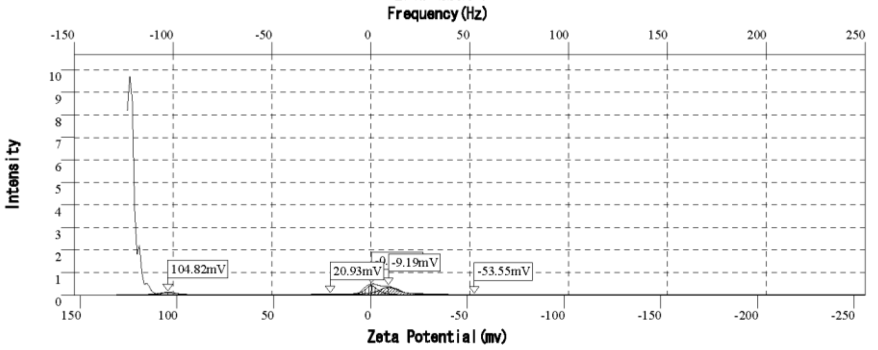

Data #2

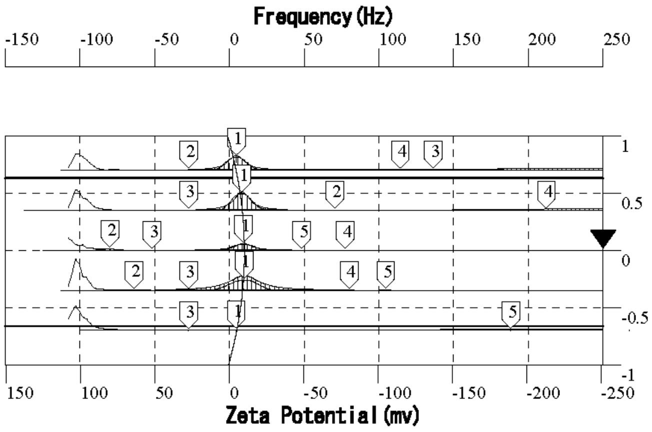

Zeta potential : −5.56 (mV)

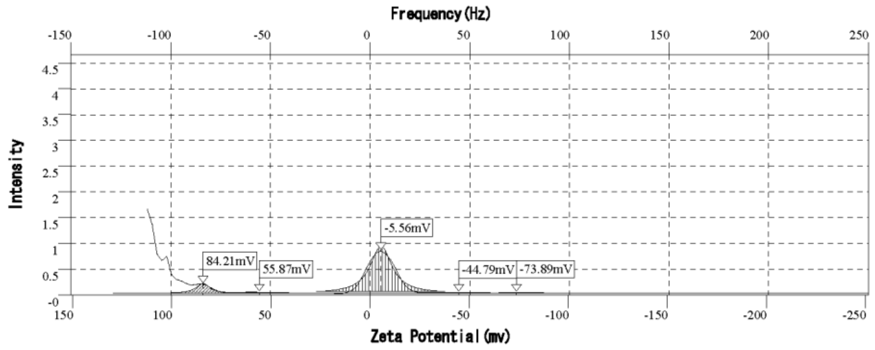

Data #3

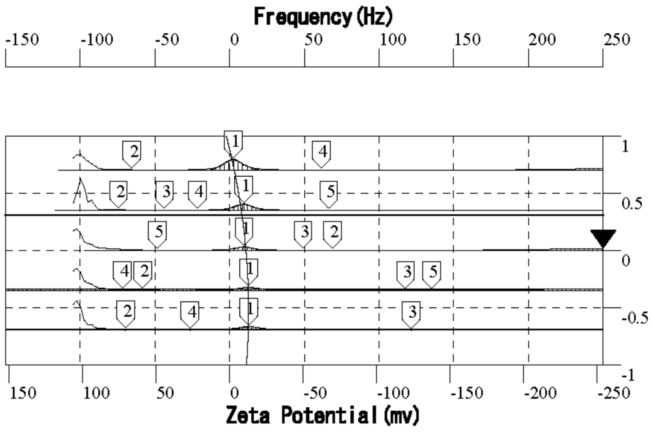

Zeta potential : −8.46 (mV)

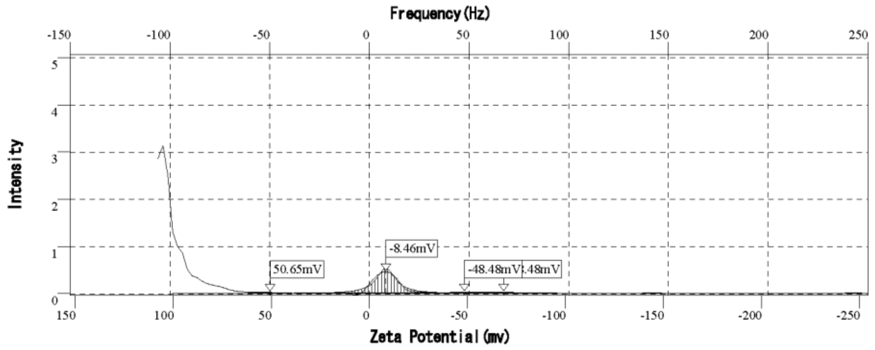

Data #4

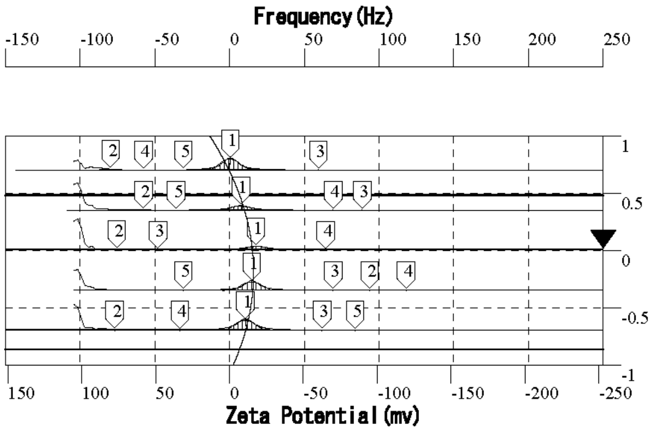

Zeta potential : −6.81 (mV)

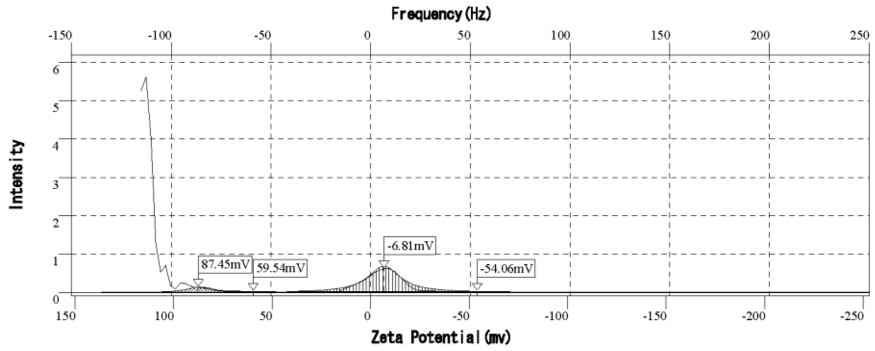

Data #5

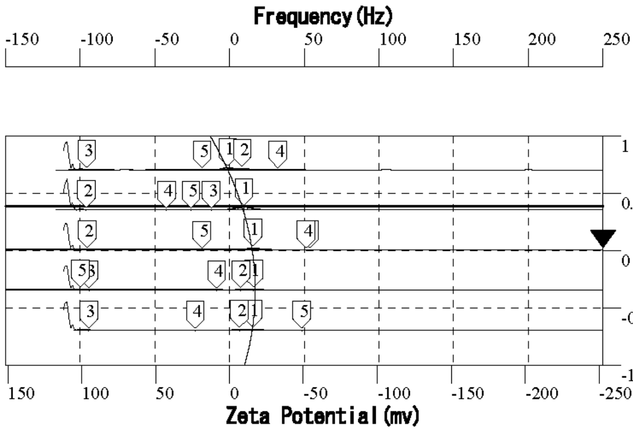

Zeta potential : −8.39 (mV)

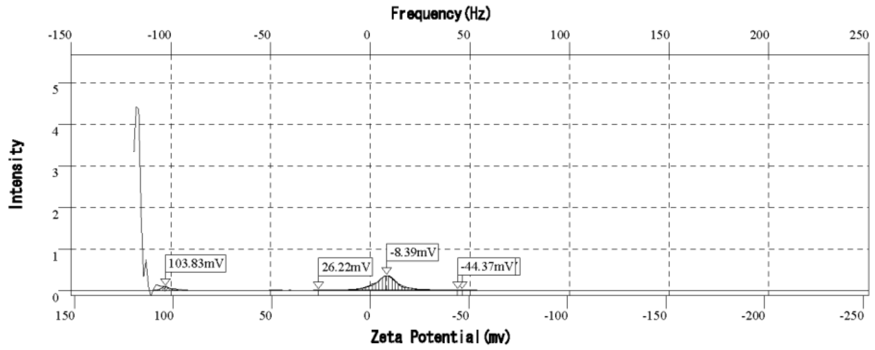

Zeta Potential of monomethyl maleate Na (**3b'**) 50 mM

Electro-osmosis plot: Mobility distribution inside cell

Data #1

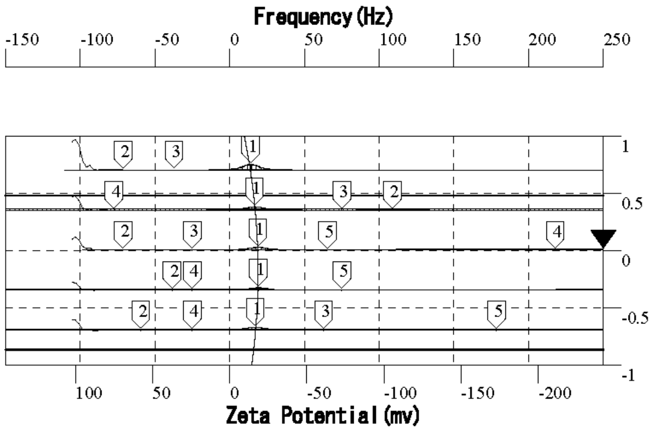

Zeta potential : −15.59 (mV)

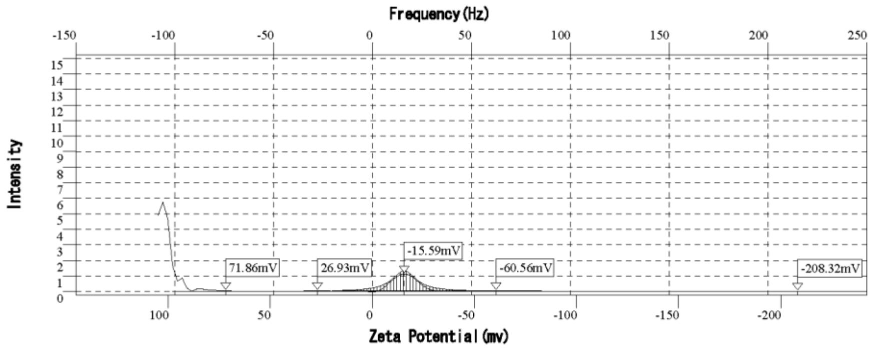

Data #2

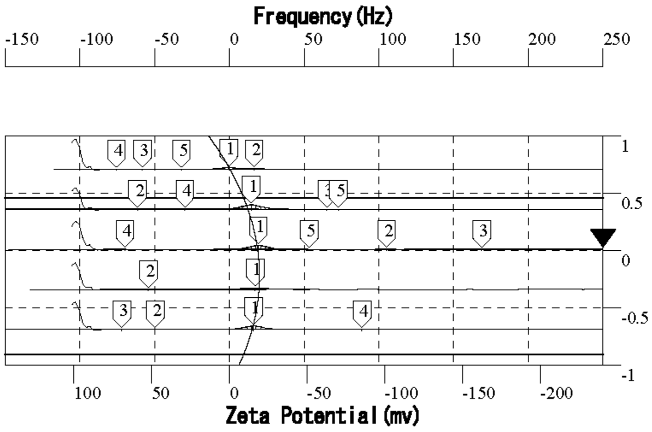

Zeta potential : −9.18 (mV)

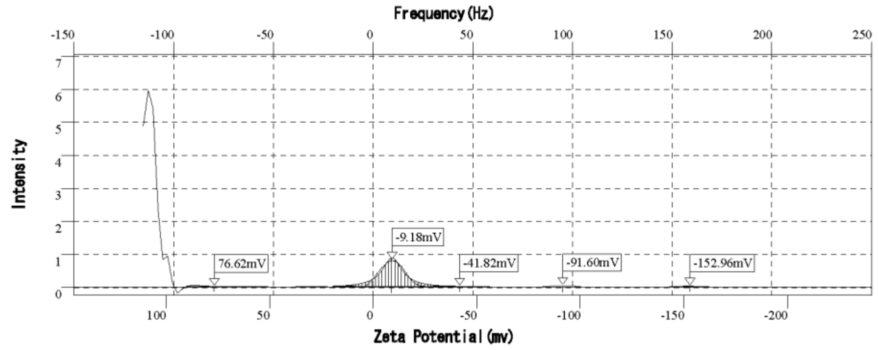

Data #3

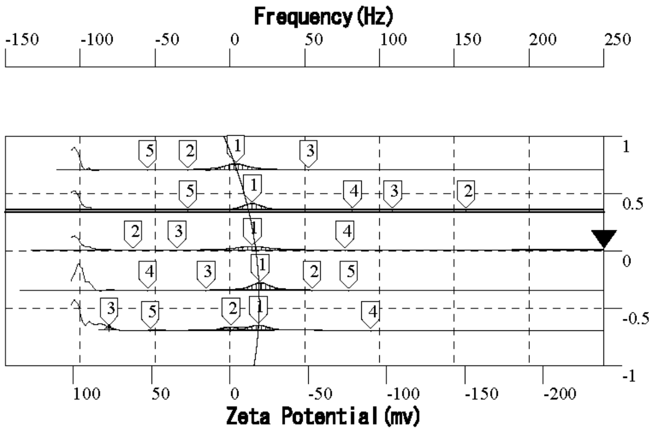

Zeta potential : −12.17 (mV)

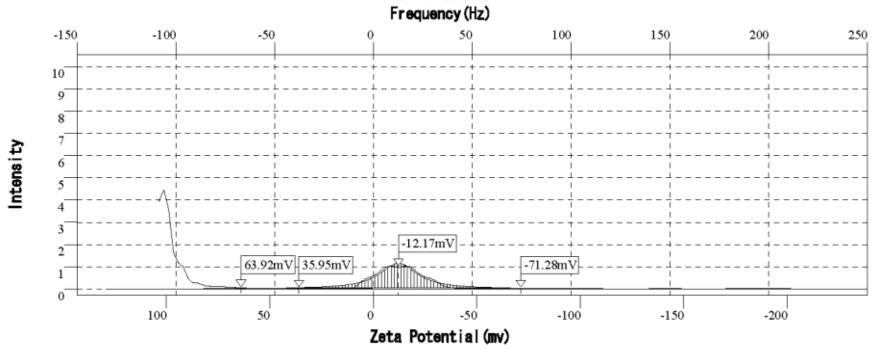

Data #4

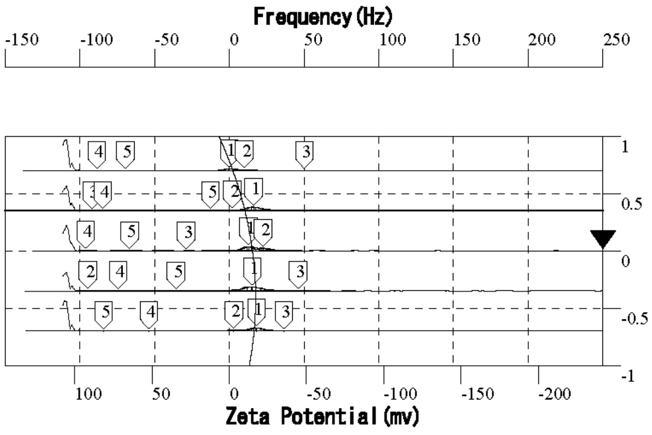

Zeta potential : −10.07 (mV)

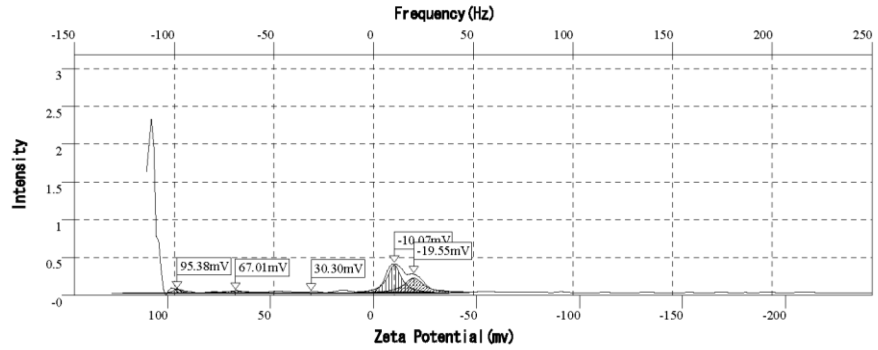

Data #5

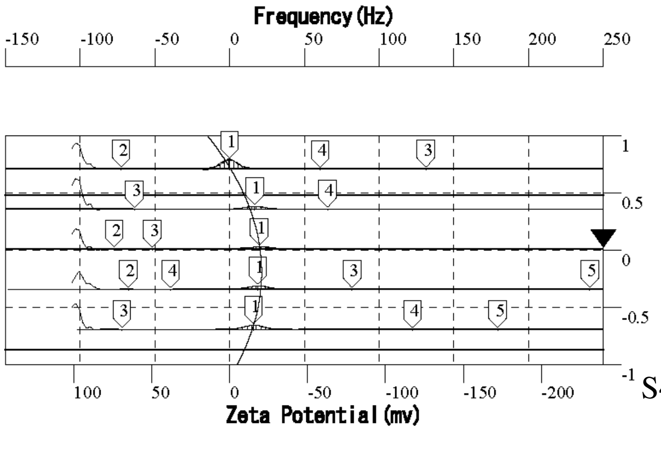

Zeta potential : −9.50 (mV)

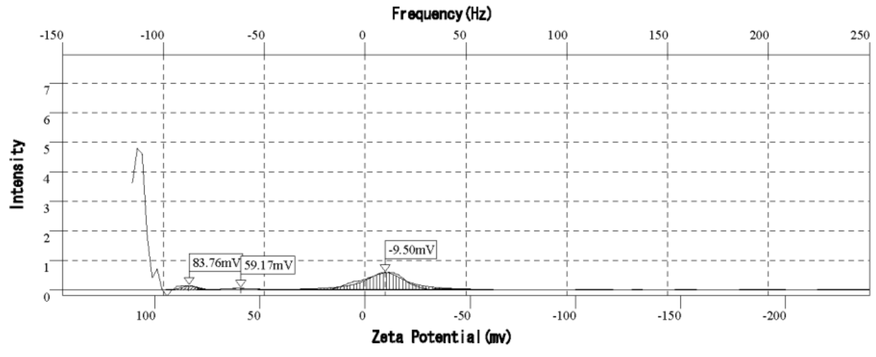

Zeta Potential of monomethyl maleate Na (**3b'**) 20 mM

Electro-osmosis plot: Mobility distribution inside cell

Data #1

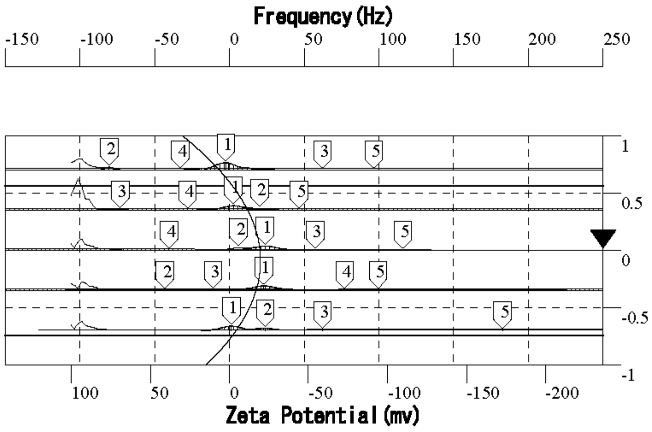

Zeta potential : −2.02 (mV)

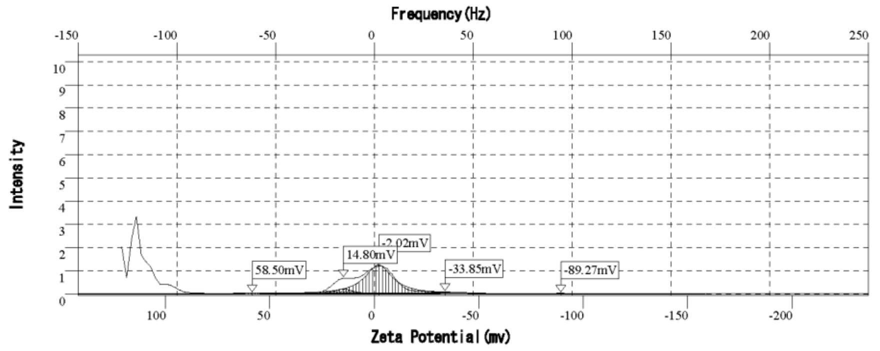

Data #2

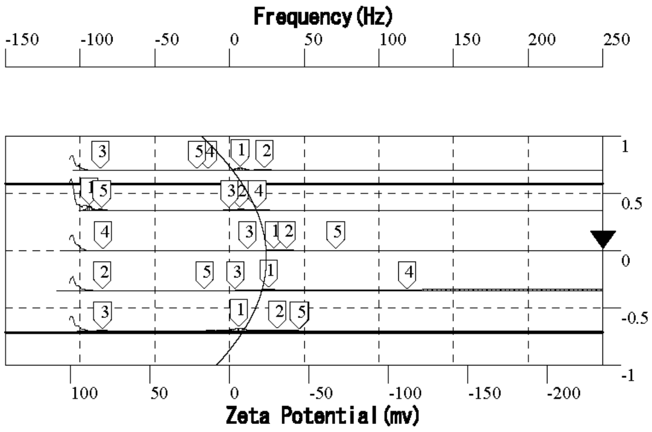

Zeta potential : −8.06 (mV)

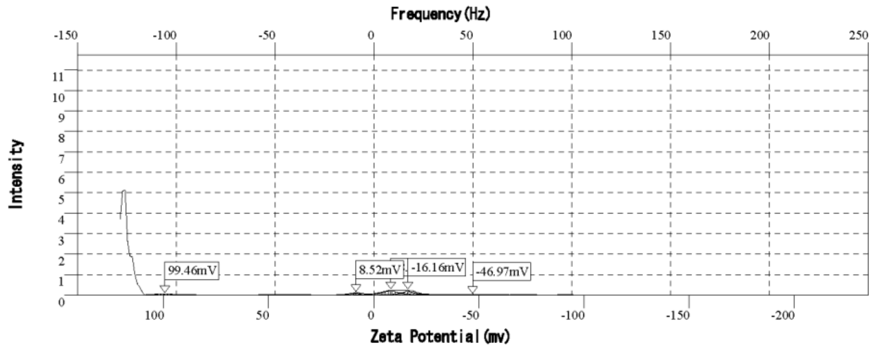

Data #3

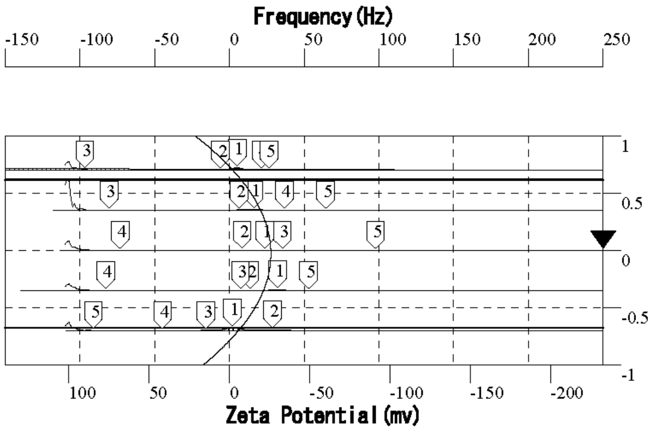

Zeta potential : −7.26 (mV)

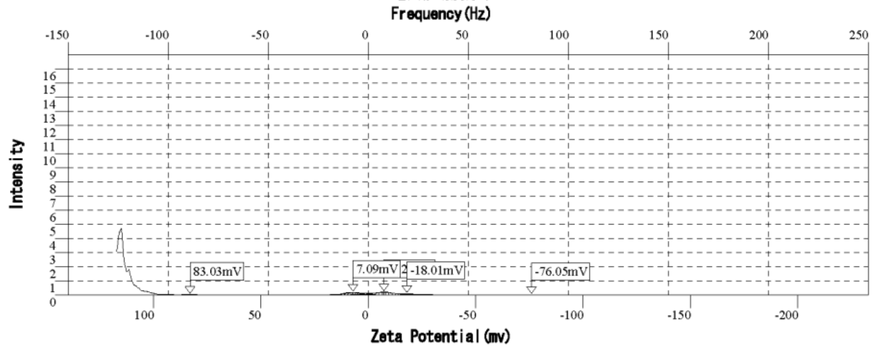

Data #4

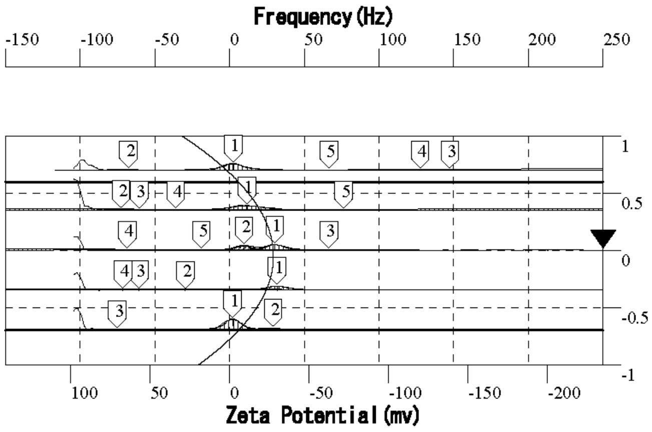

Zeta potential : −5.73 (mV)

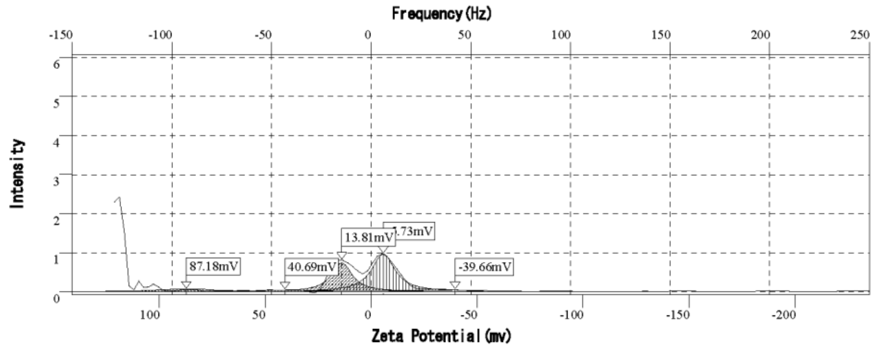

Data #5

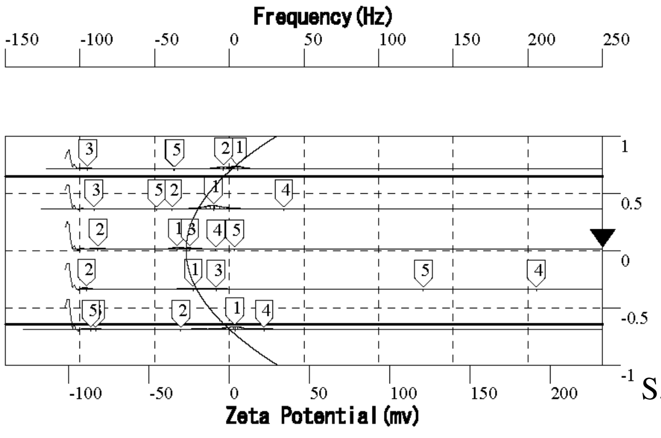

Zeta potential : −3.22 (mV)

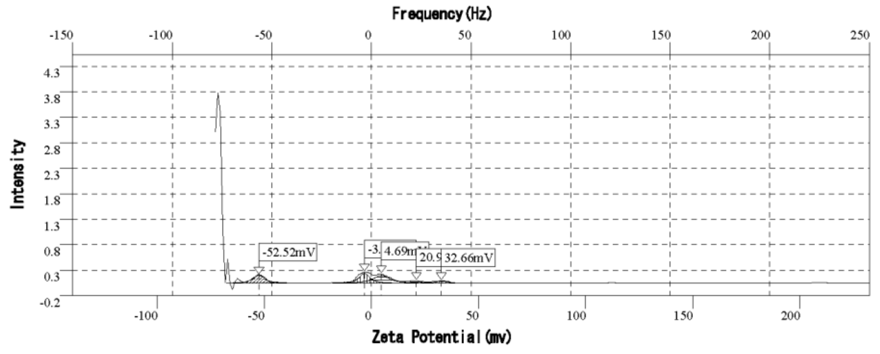

Zeta Potential of monomethyl maleate Na (**3b'**) 10 mM

Electro-osmosis plot: Mobility distribution inside cell

Data #1

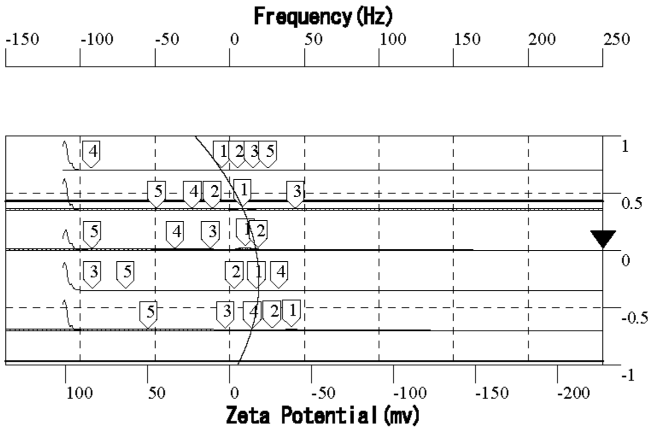

Zeta potential : −5.99 (mV)

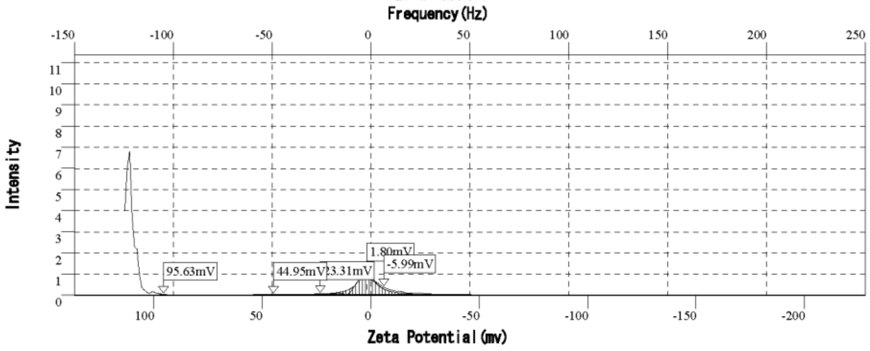

Data #2

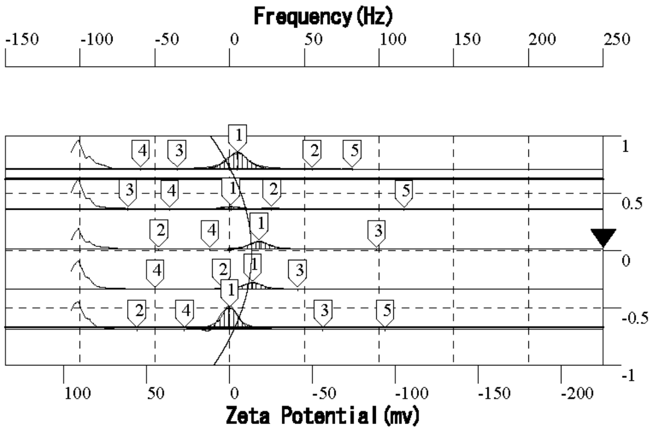

Zeta potential : −3.38 (mV)

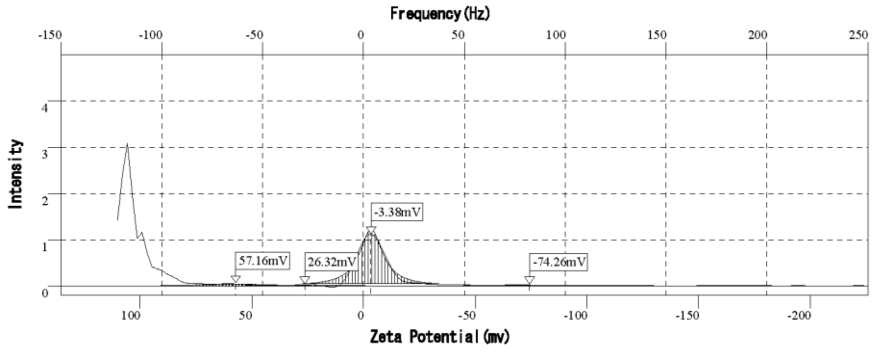

Data #3

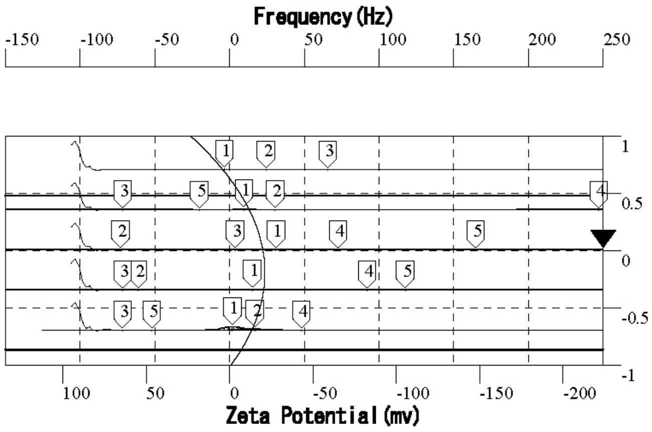

Zeta potential : −7.01 (mV)

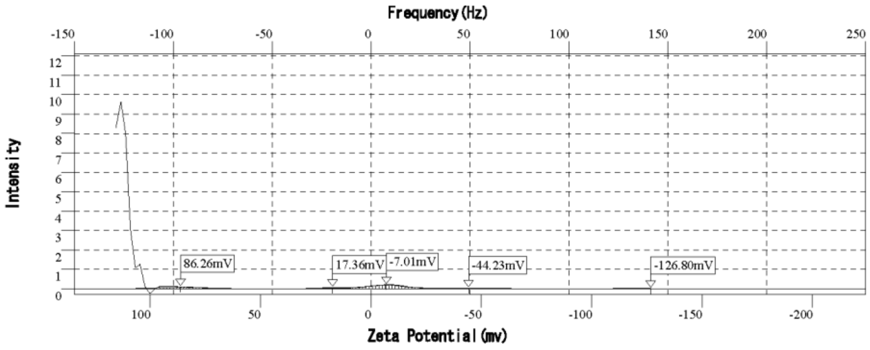

Data #4

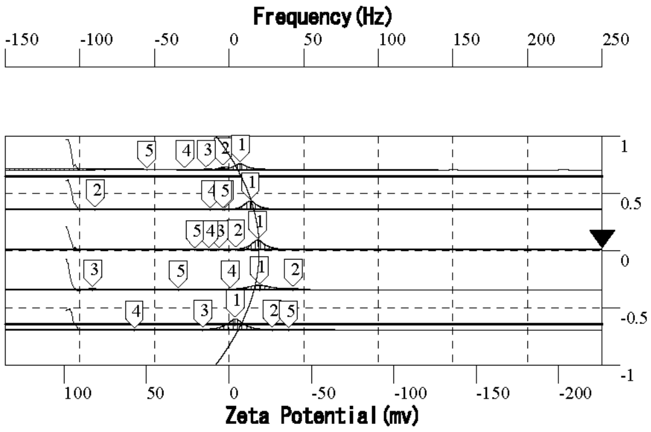

Zeta potential : −7.36 (mV)

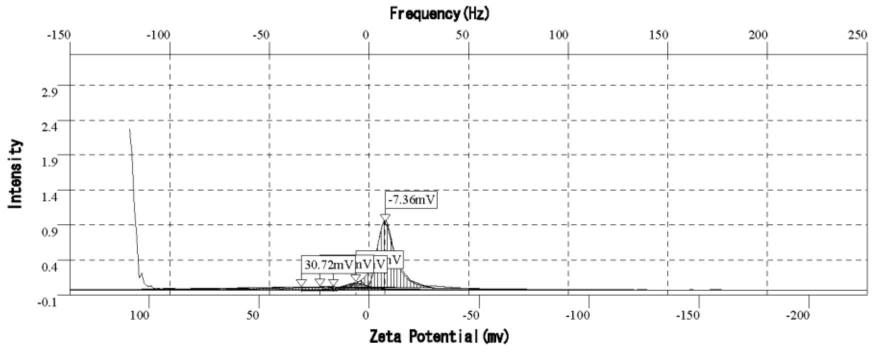

Data #5

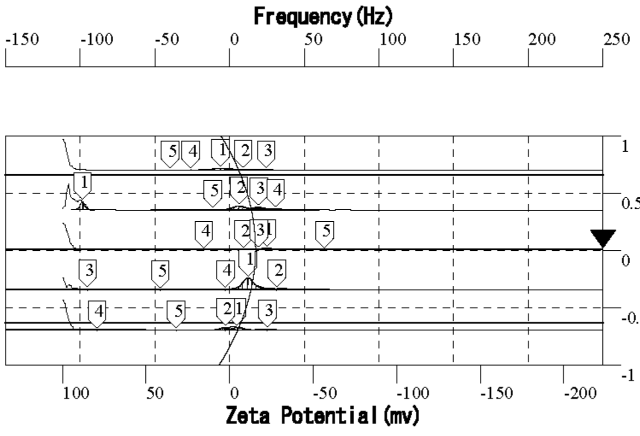

Zeta potential : −6.71 (mV)

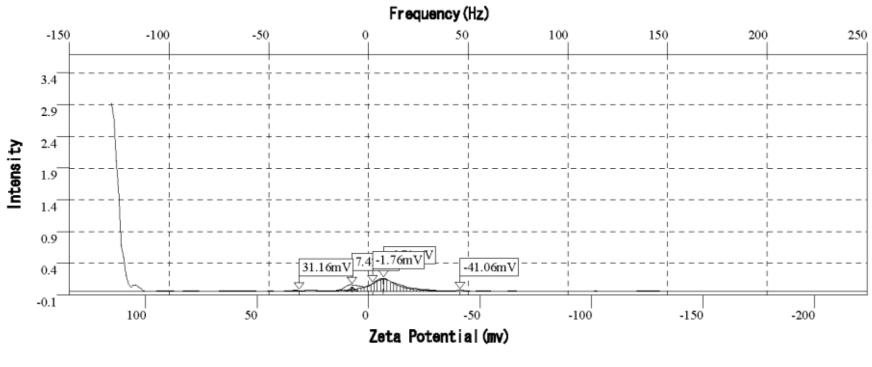

Zeta Potential of monoethylphenyl malonate Na (**4b'**) 250 mM

Electro-osmosis plot: Mobility distribution inside cell

Data #1

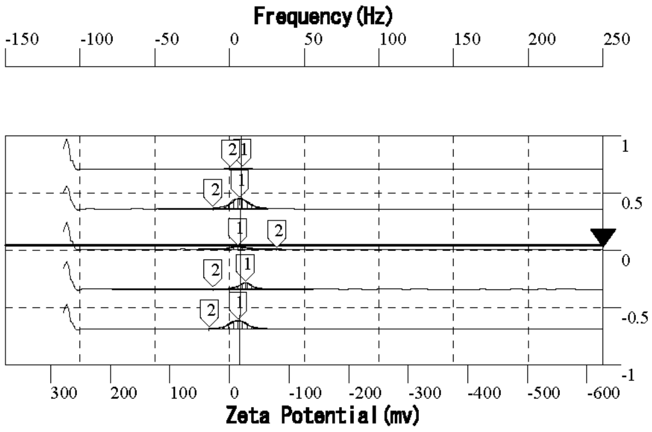

Zeta potential : −18.76 (mV)

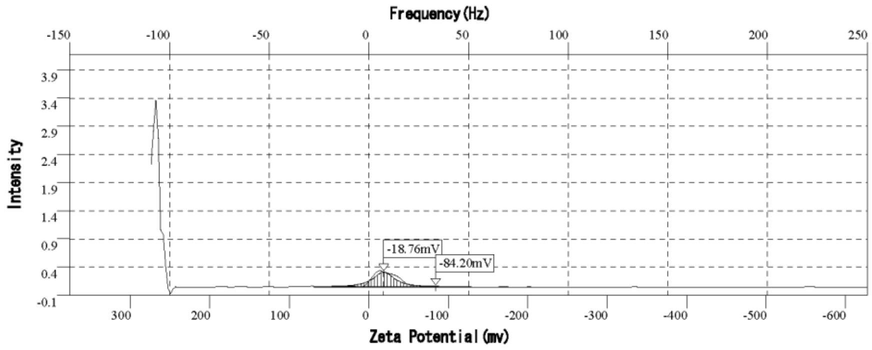

Data #2

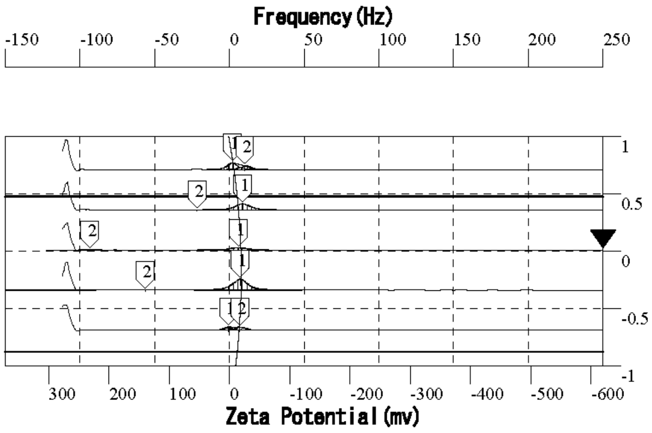

Zeta potential : −13.12 (mV)

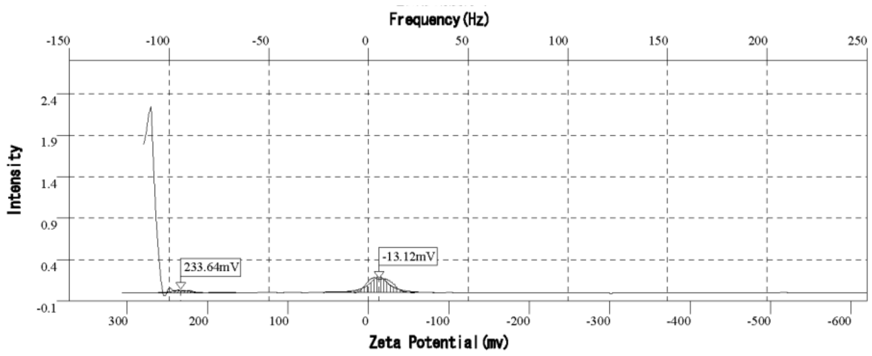

Data #3

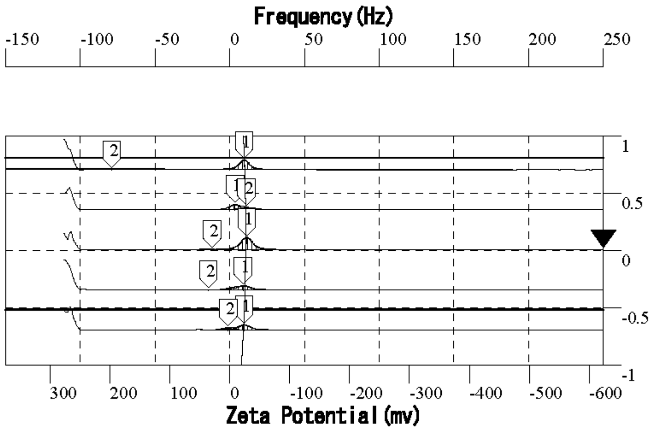

Zeta potential : −24.43 (mV)

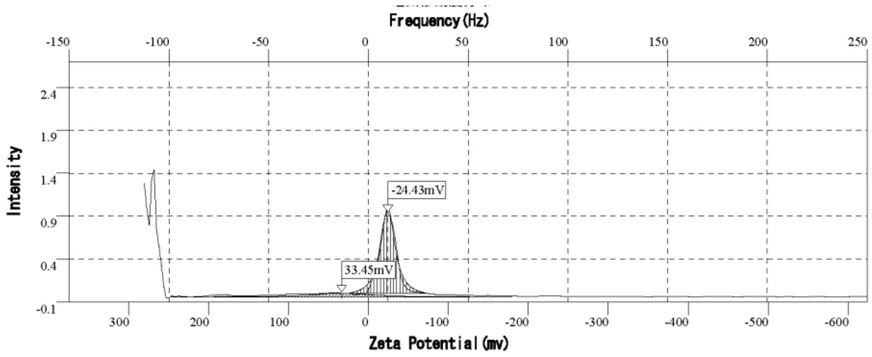

Data #4

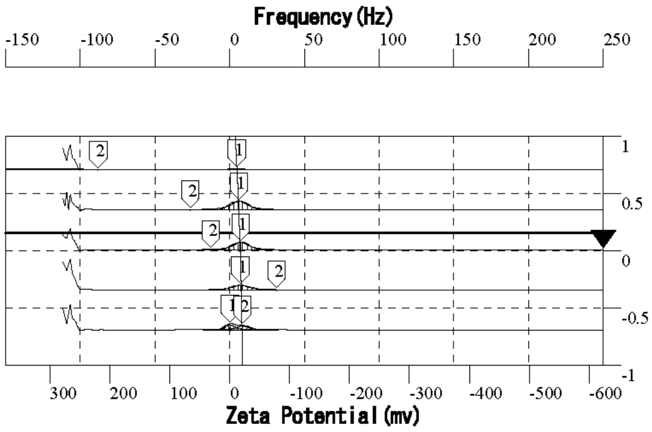

Zeta potential : −16.77 (mV)

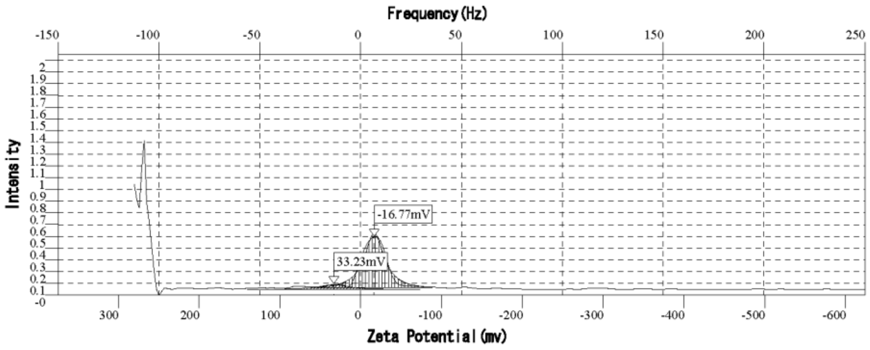

Data #5

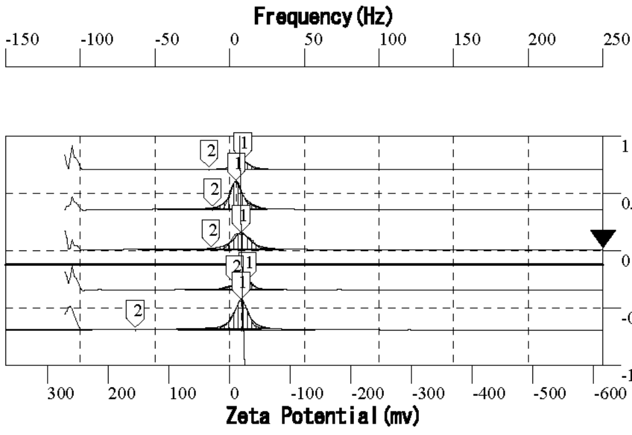

Zeta potential : −20.24 (mV)

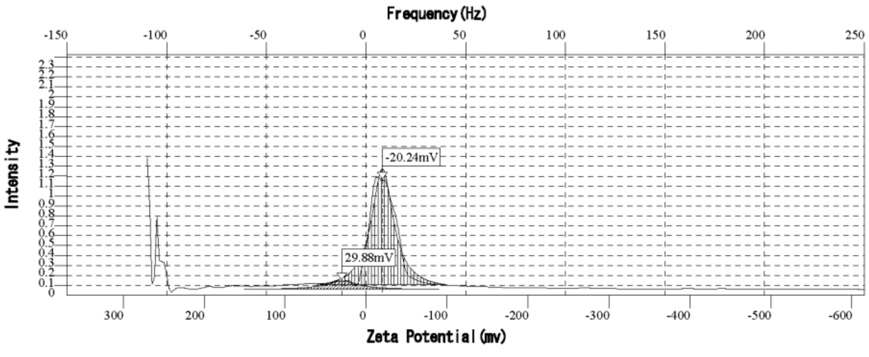

Zeta Potential of monoethylphenyl malonate Na (**4b'**) 100 mM

Electro-osmosis plot: Mobility distribution inside cell

Data #1

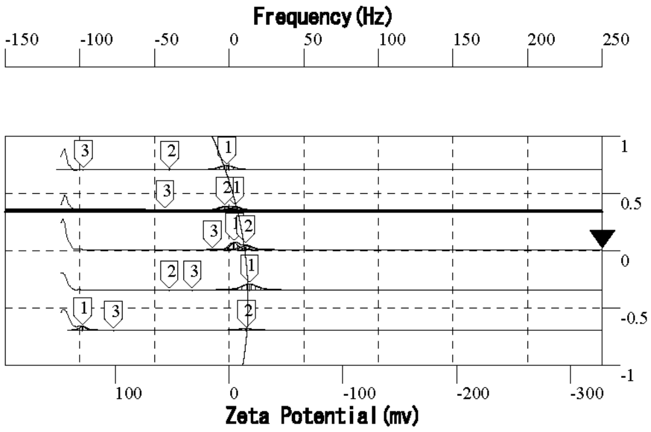

Zeta potential : −7.50 (mV)

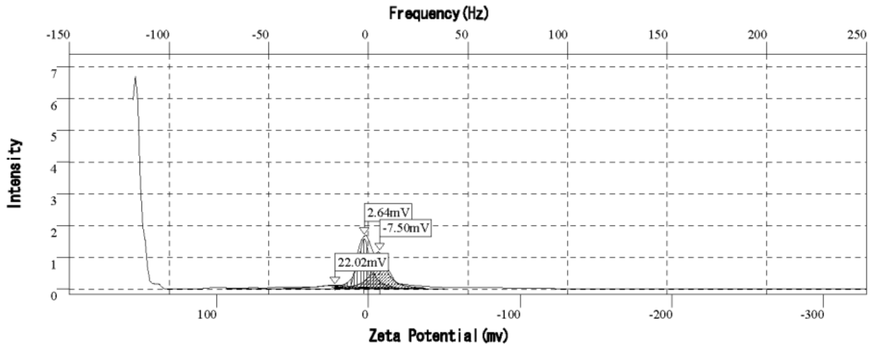

Data #2

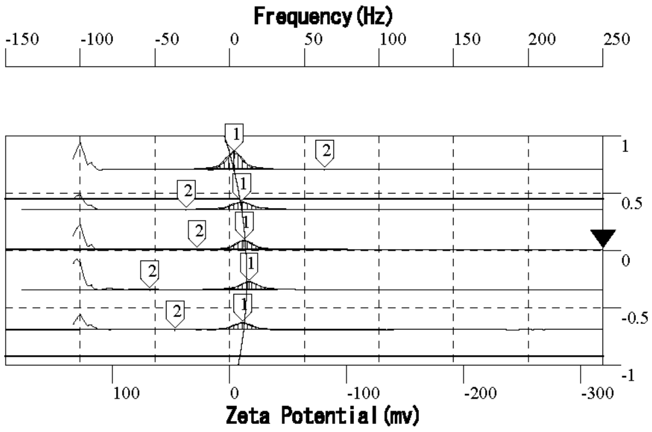

Zeta potential : −8.85 (mV)

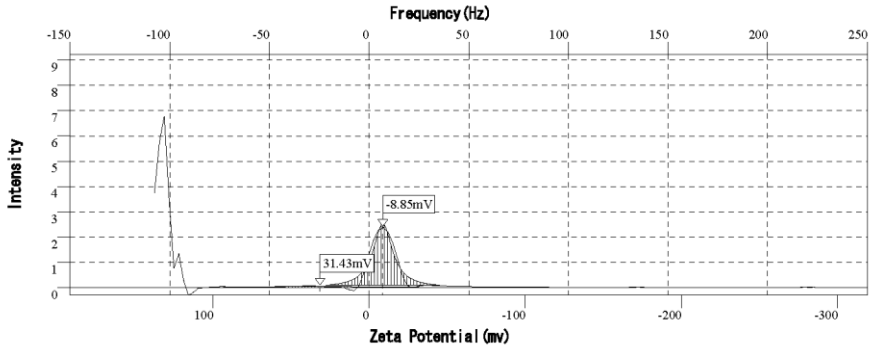

Data #3

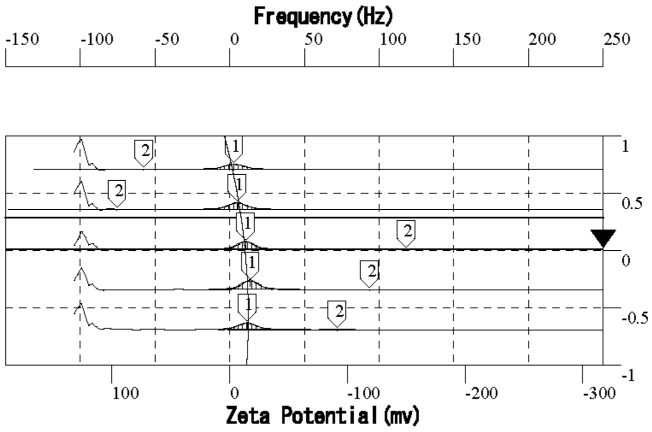

Zeta potential : −9.75 (mV)

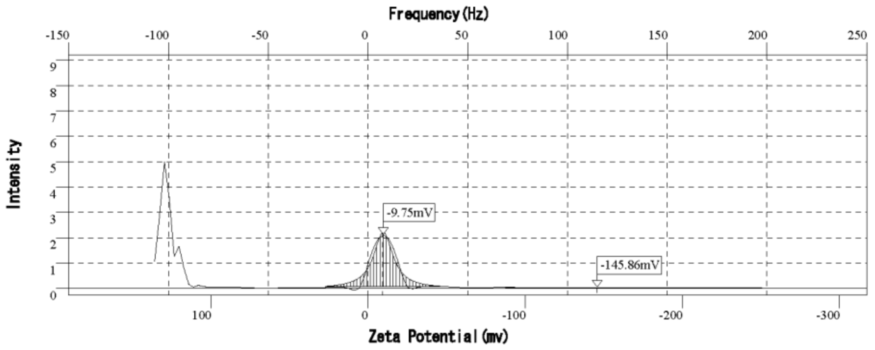

Data #4

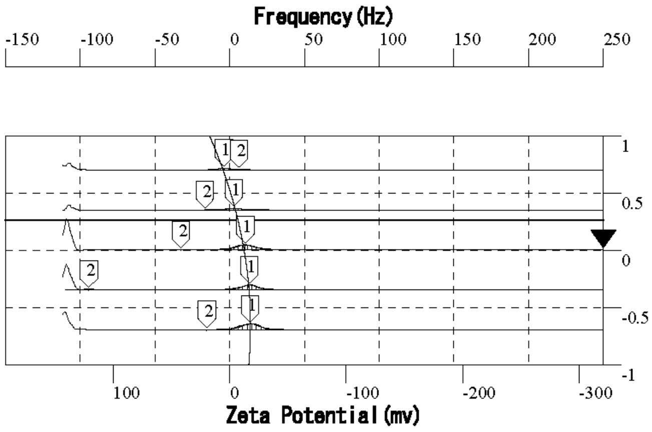

Zeta potential : −7.10 (mV)

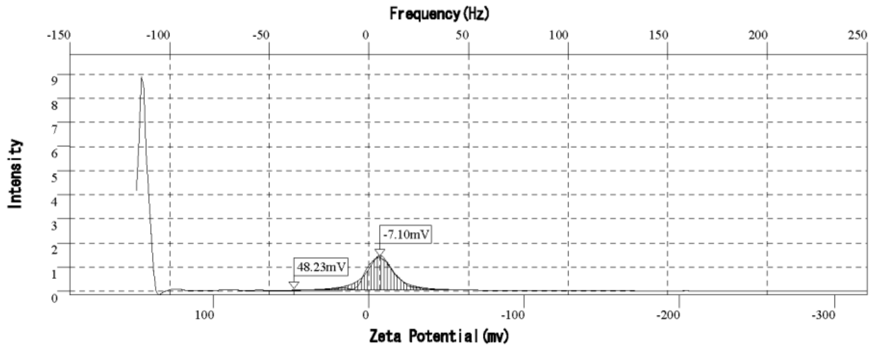

Data #5

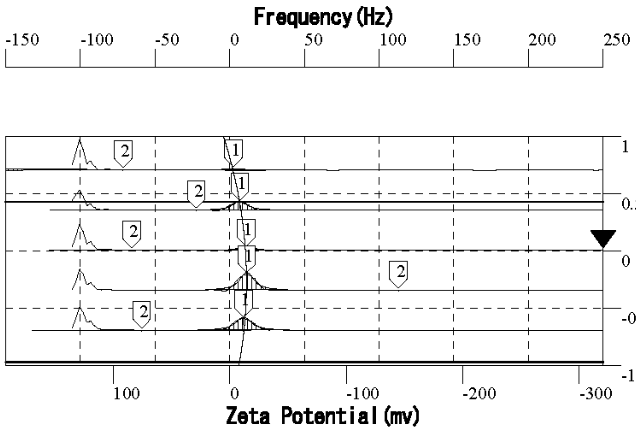

Zeta potential : −8.52 (mV)

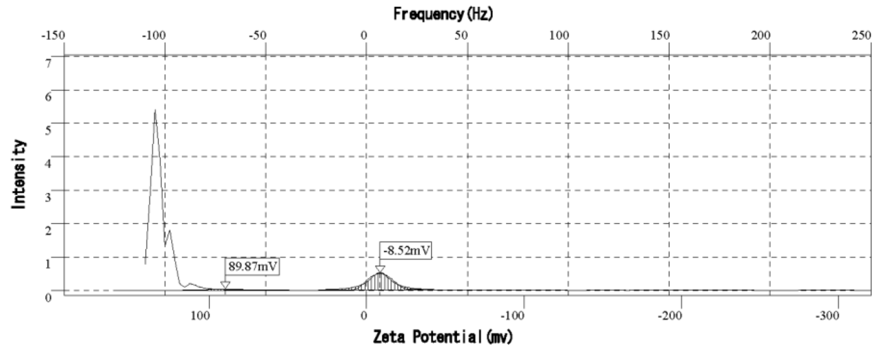

Zeta Potential of monoethylphenyl malonate Na (**4b'**) 50 mM

Electro-osmosis plot: Mobility distribution inside cell

Data #1

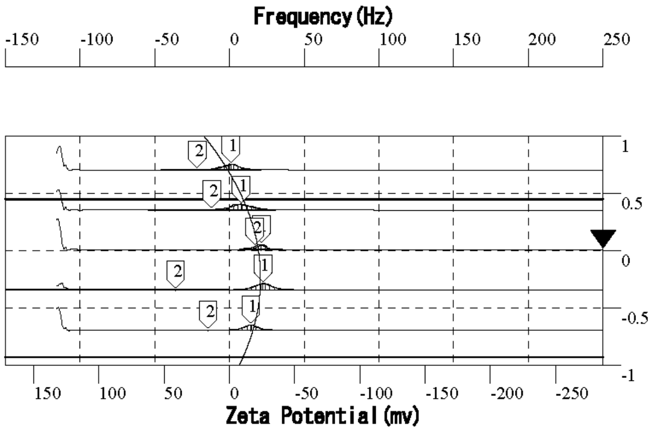

Zeta potential : −10.45 (mV)

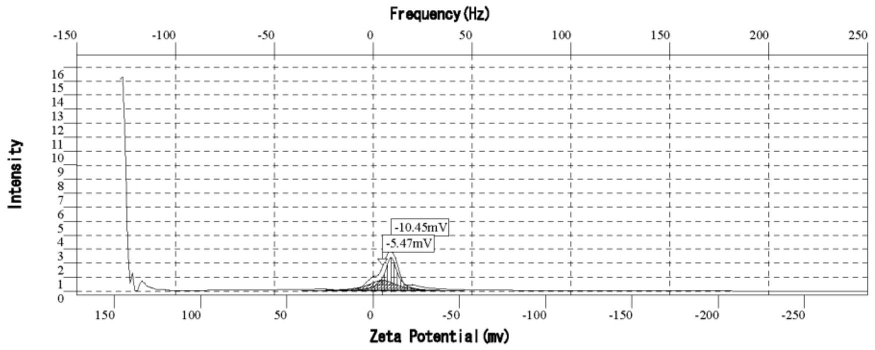

Data #2

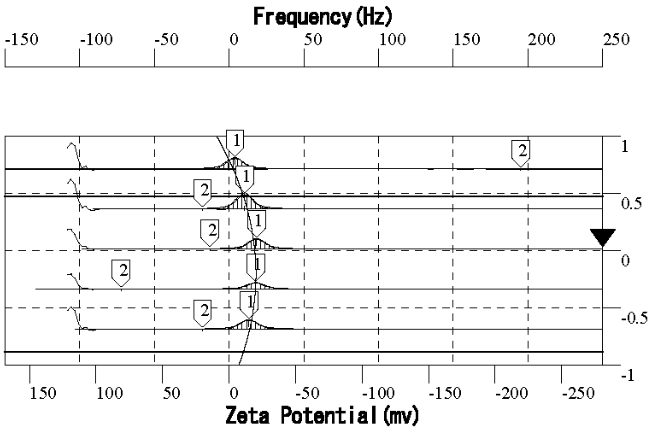

Zeta potential : −10.89 (mV)

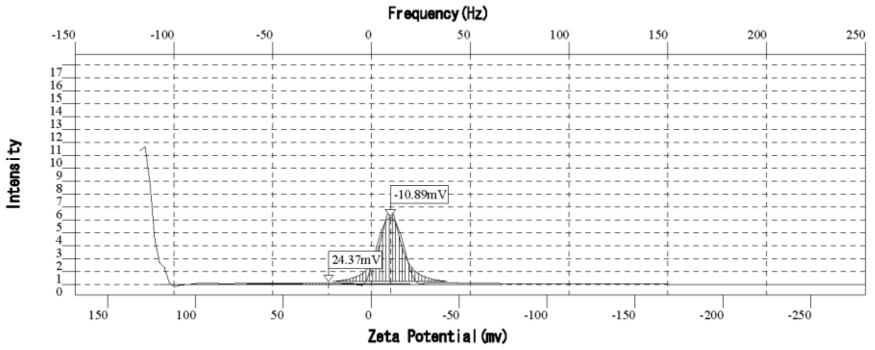

Data #3

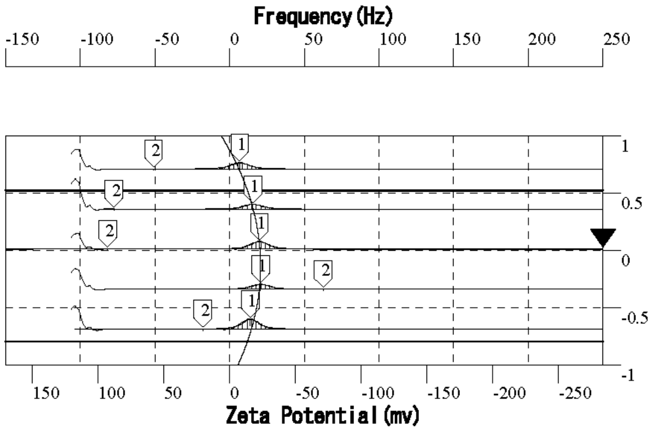

Zeta potential : −13.55 (mV)

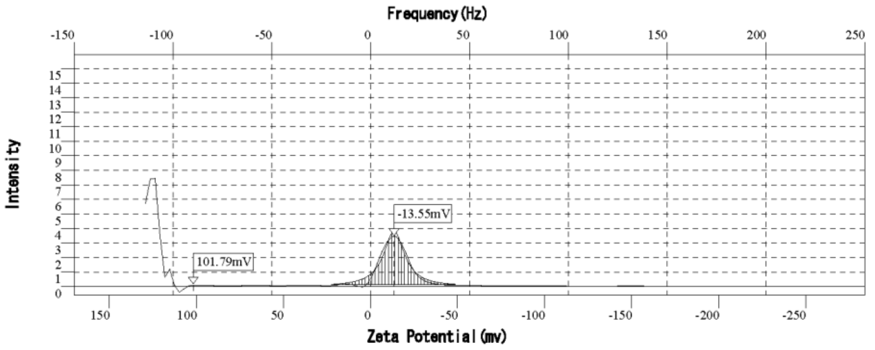

Data #4

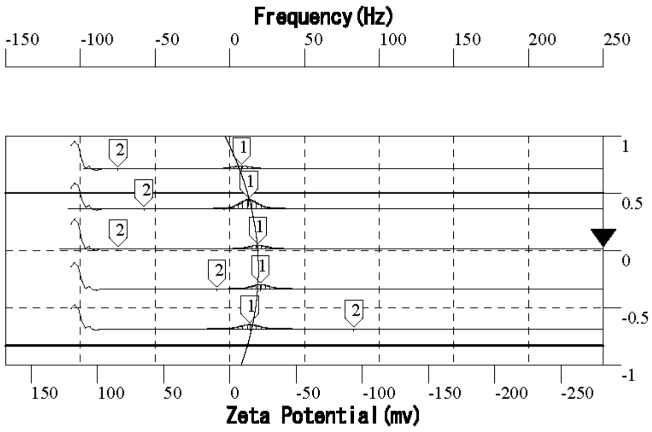

Zeta potential : −13.39 (mV)

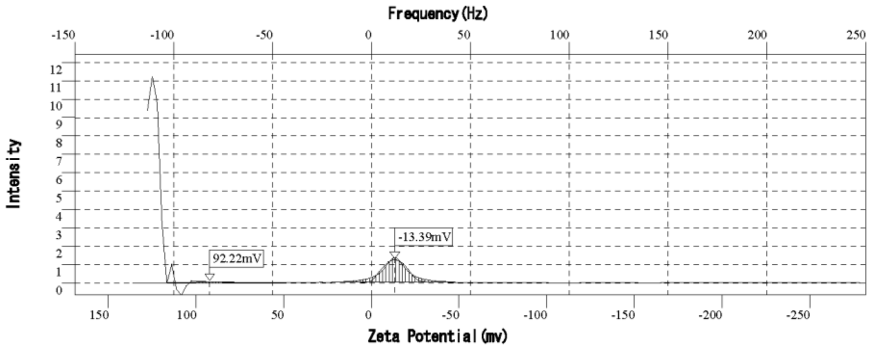

Data #5

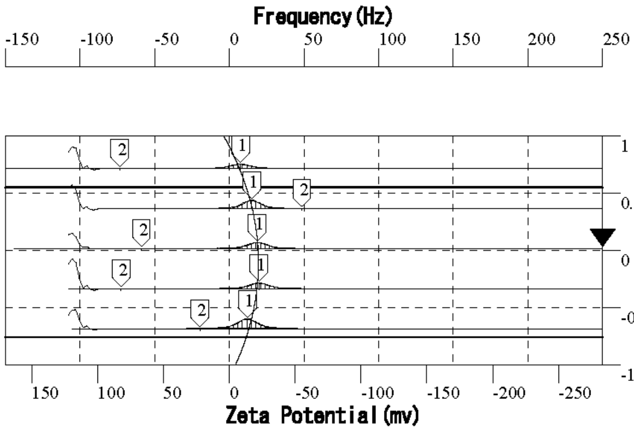

Zeta potential : −12.88 (mV)

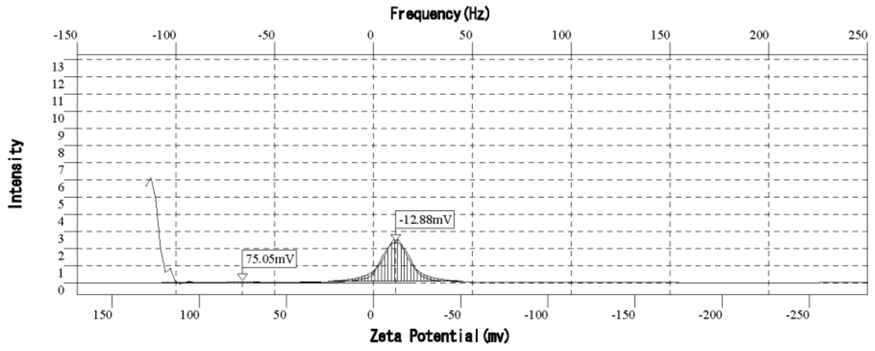

Zeta Potential of monoethylphenyl malonate Na (**4b'**) 20 mM

Electro-osmosis plot: Mobility distribution inside cell

Data #1

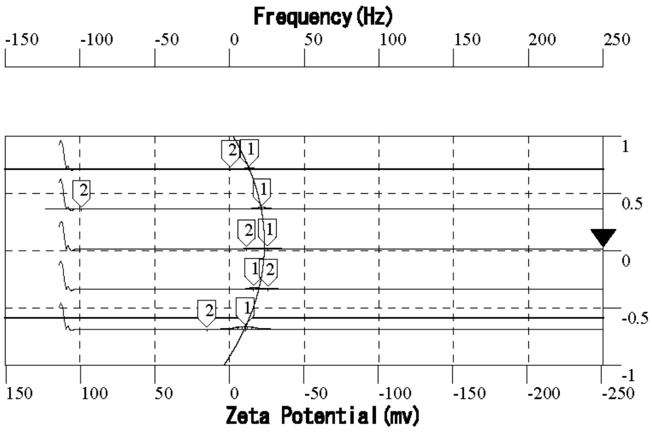

Zeta potential : −13.42 (mV)

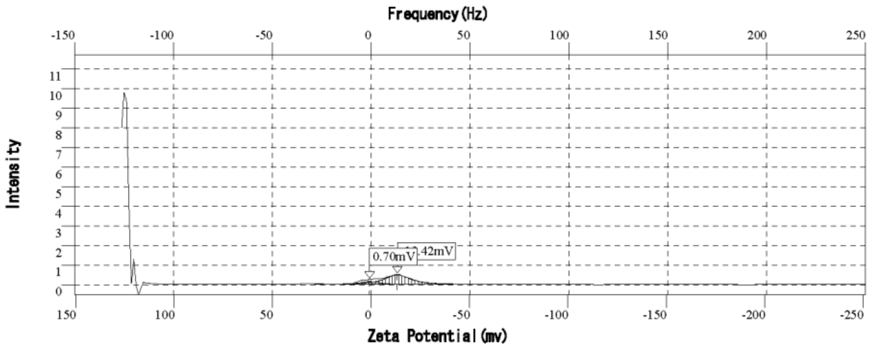

Data #2

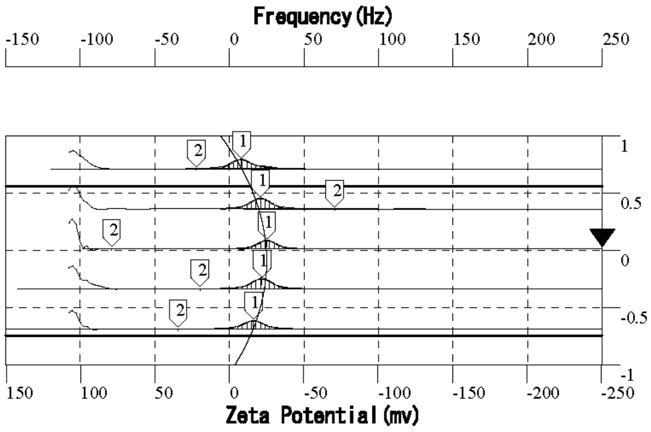

Zeta potential : −14.08 (mV)

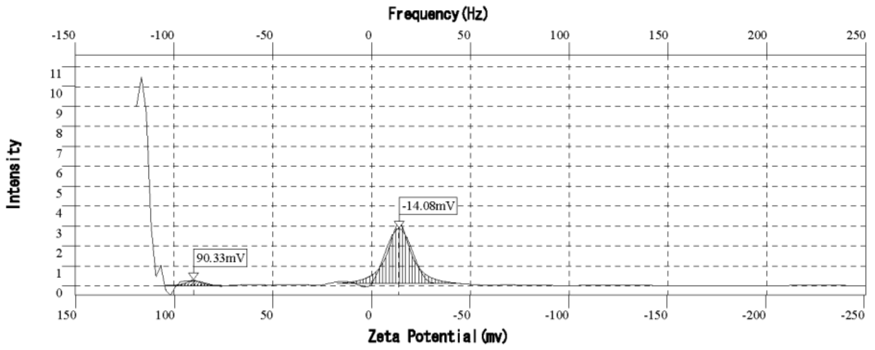

Data #3

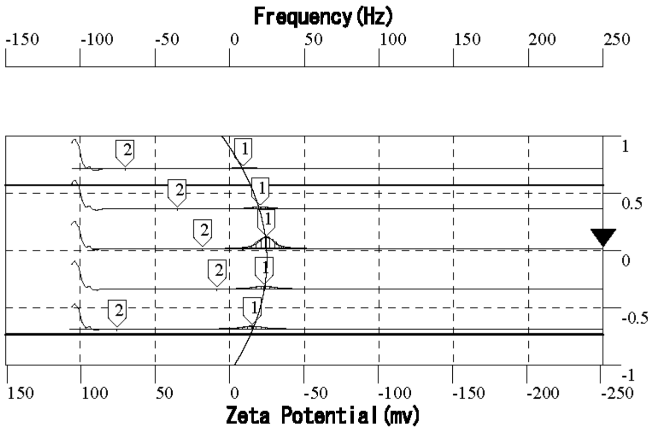

Zeta potential : −14.11 (mV)

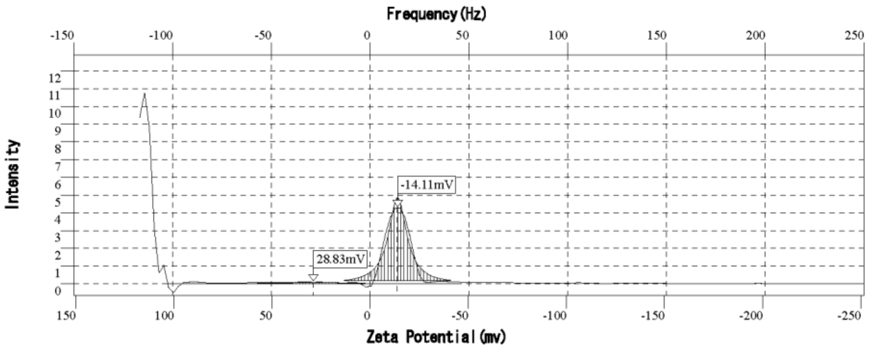

Data #4

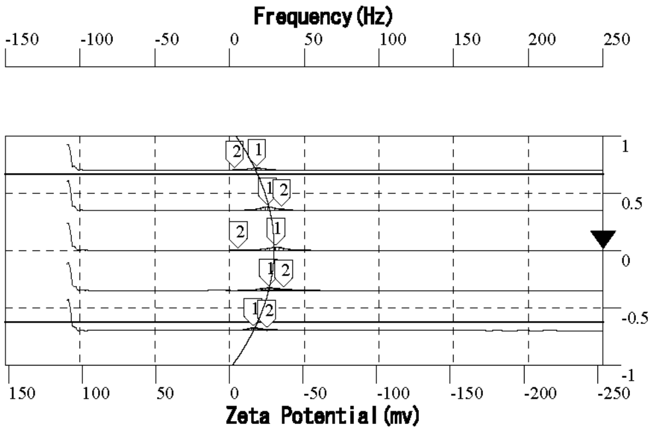

Zeta potential : −19.06 (mV)

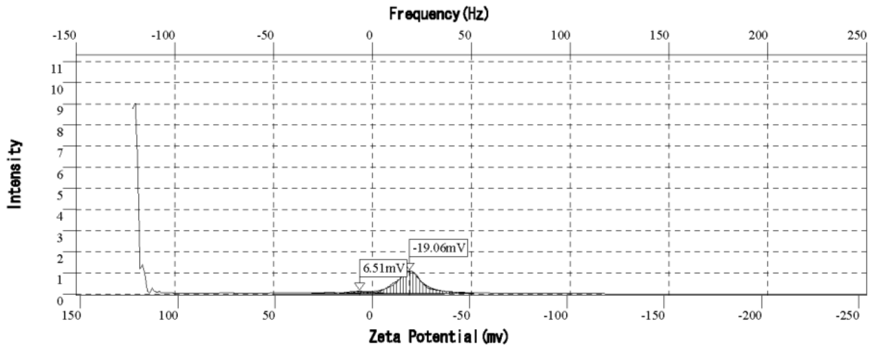

Data #5

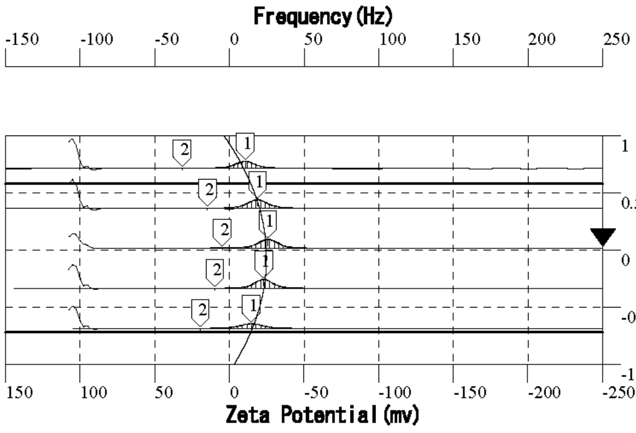

Zeta potential : −14.35 (mV)

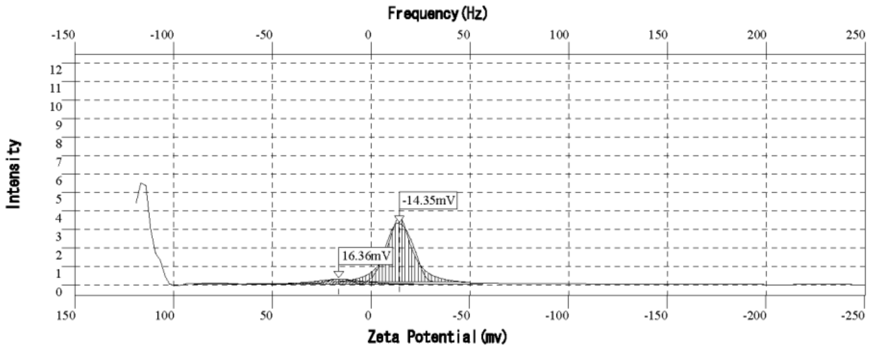

Zeta Potential of monoethylphenyl malonate Na (**4b'**) 10 mM

Electro-osmosis plot: Mobility distribution inside cell

Data #1

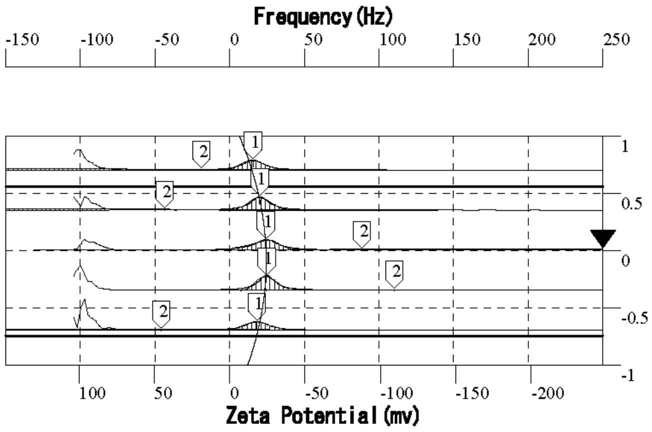

Zeta potential : −17.95 (mV)

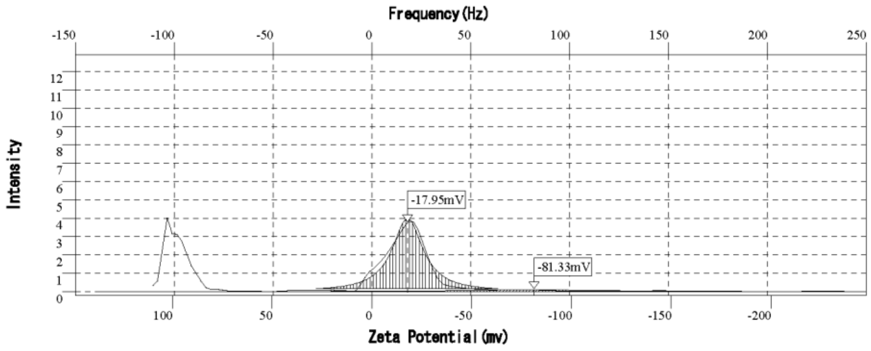

Data #2

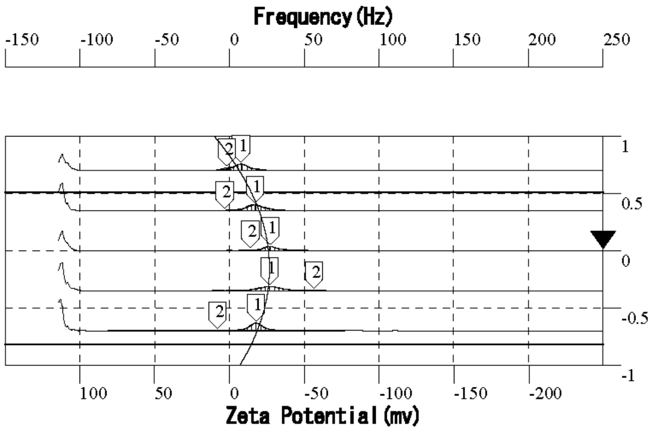

Zeta potential : −14.56 (mV)

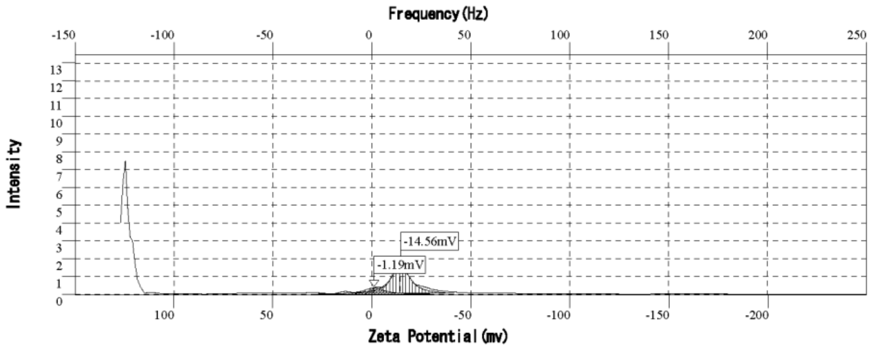

Data #3

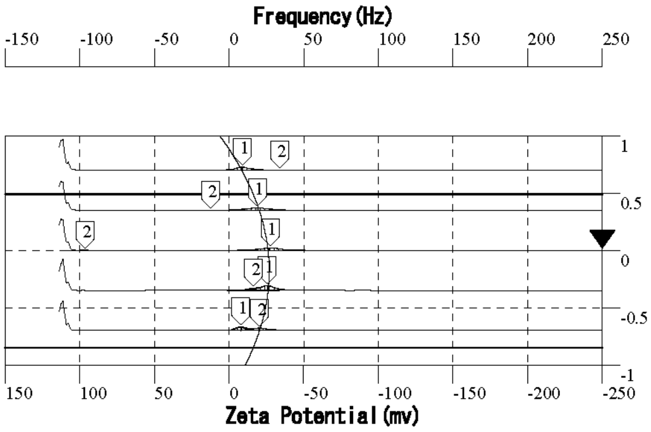

Zeta potential : −16.21 (mV)

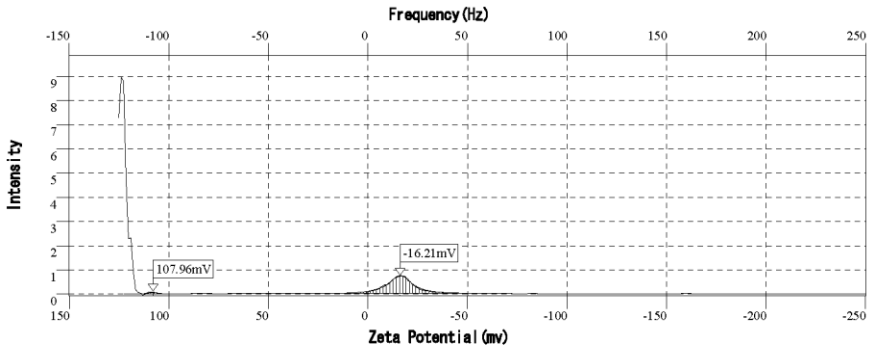

Data #4

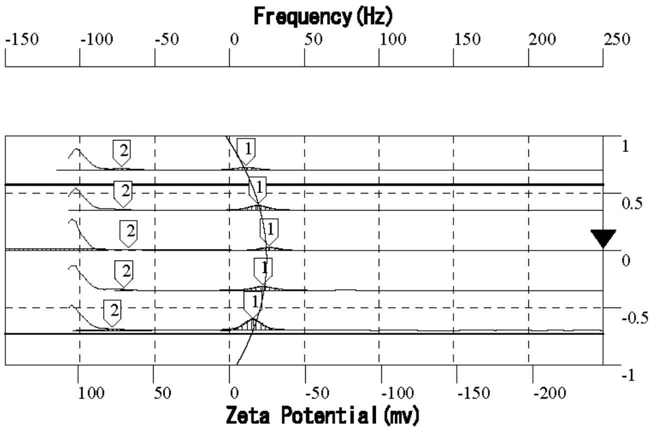

Zeta potential : −14.67 (mV)

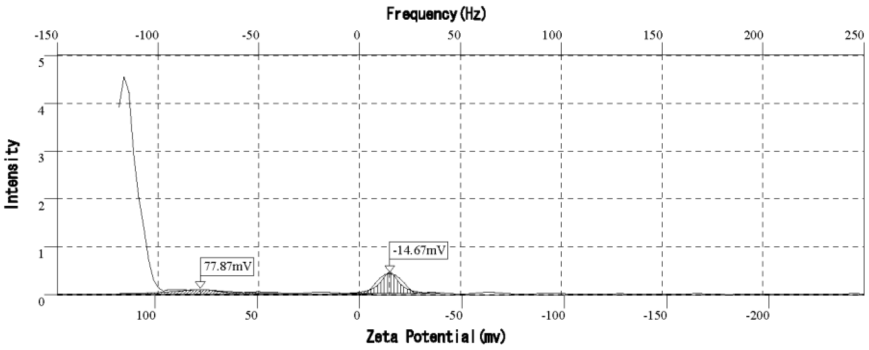

Data #5

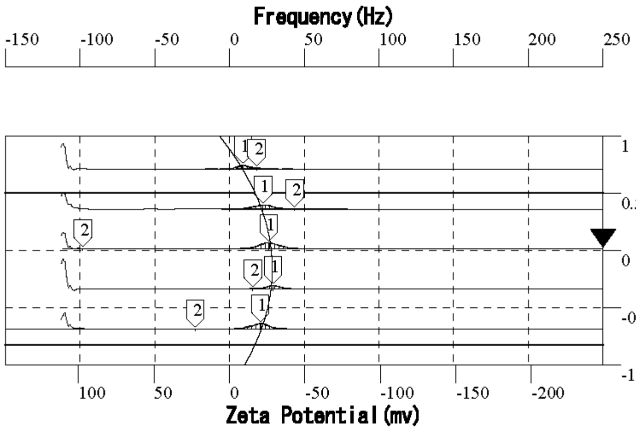

Zeta potential : −16.85 (mV)

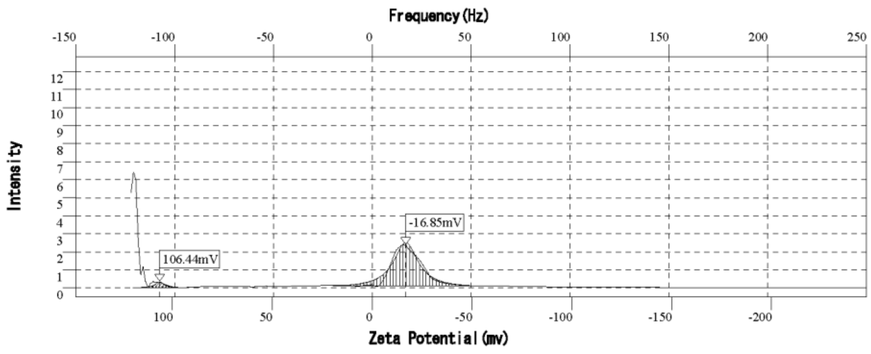

Zeta Potential of monomethyl malonate K (**5b'**) 250 mM

Electro-osmosis plot: Mobility distribution inside cell

Data #1

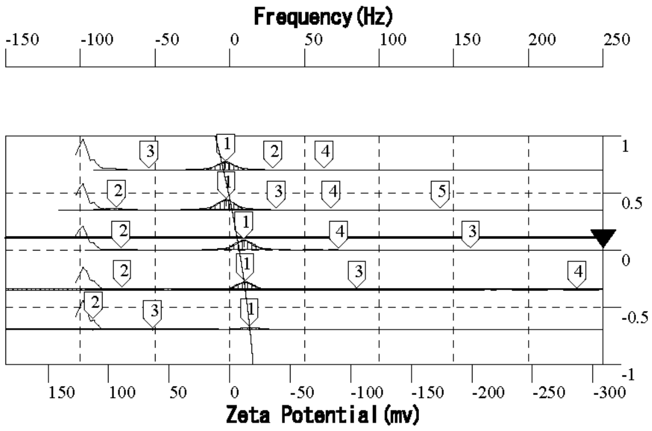

Zeta potential : −6.20 (mV)

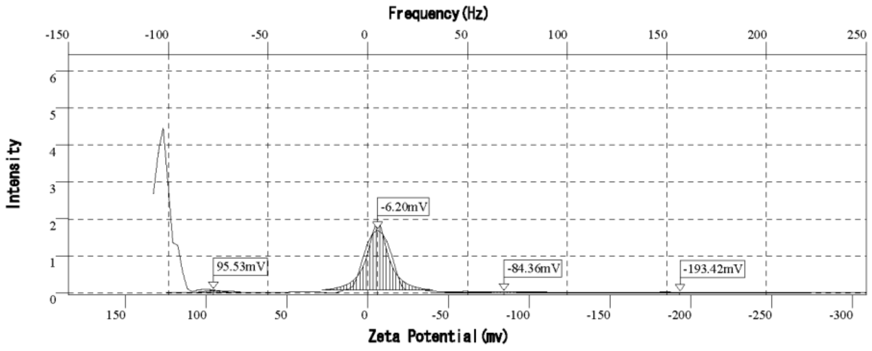

Data #2

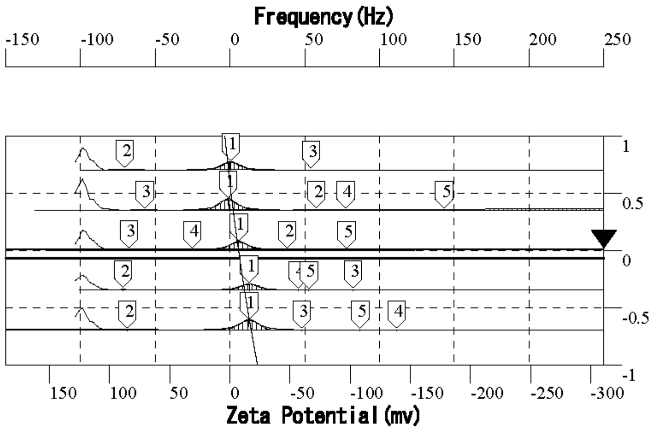

Zeta potential : −8.11 (mV)

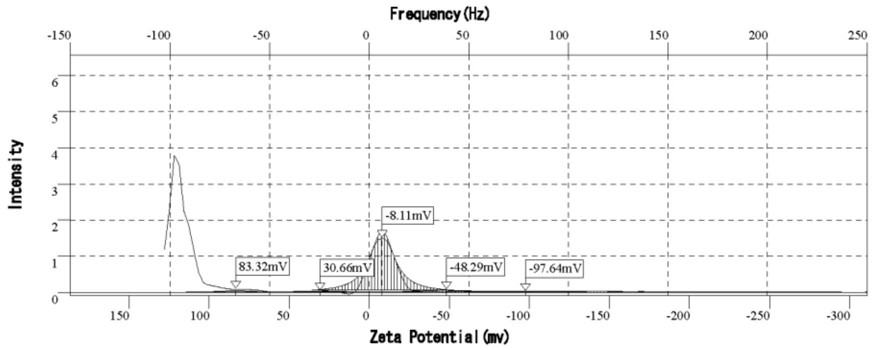

Data #3

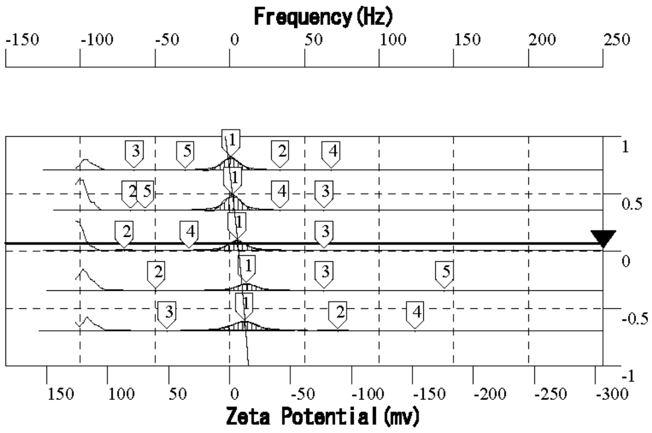

Zeta potential : −6.81 (mV)

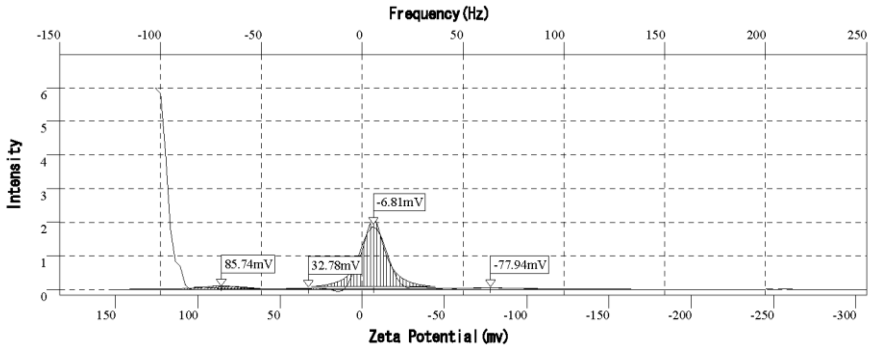

Data #4

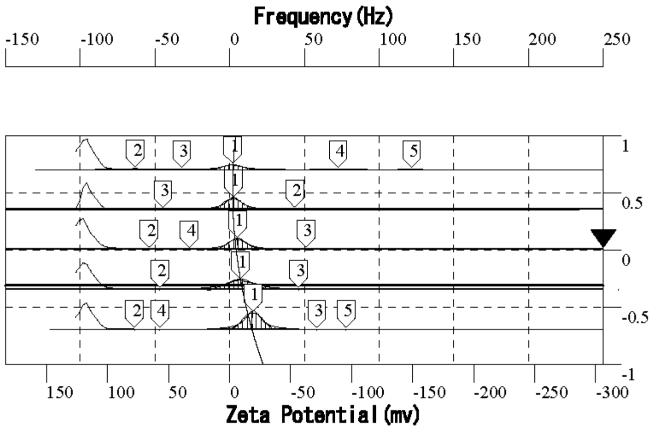

Zeta potential : −9.85 (mV)

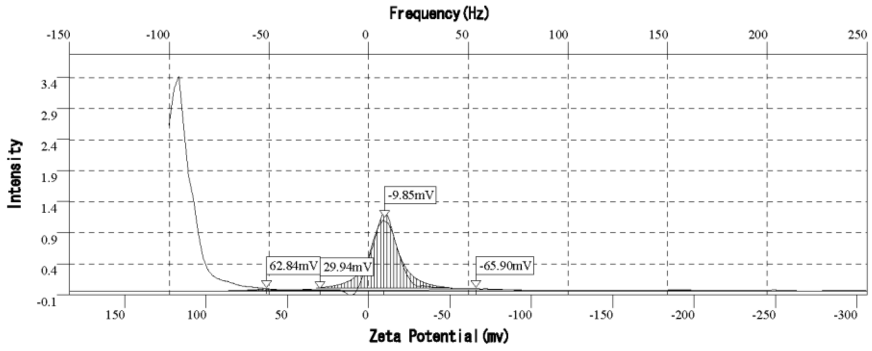

Data #5

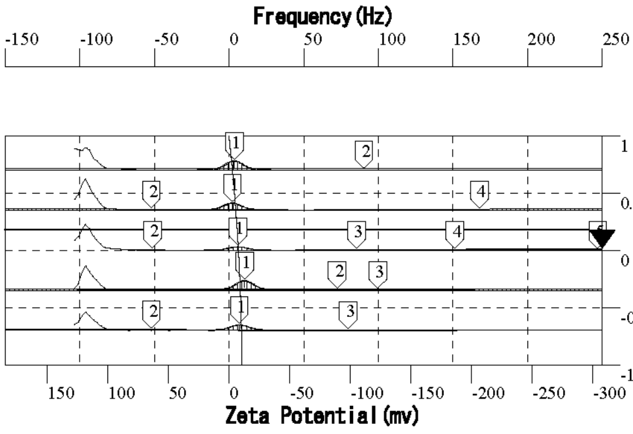

Zeta potential : −6.68 (mV)

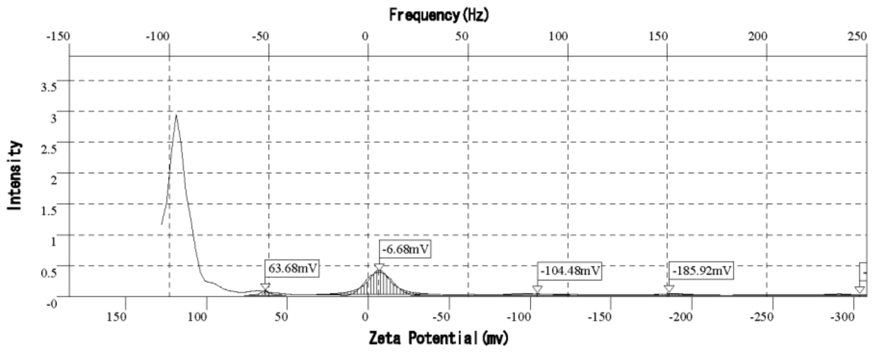

Zeta Potential of monomethyl malonate K (**5b'**) 100 mM

Electro-osmosis plot: Mobility distribution inside cell

Data #1

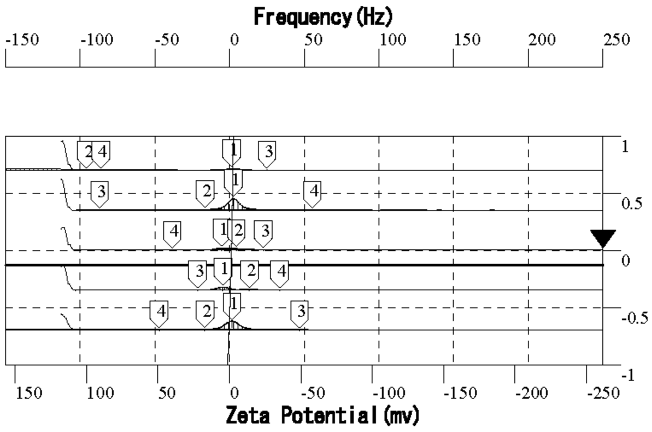

Zeta potential : −1.11 (mV)

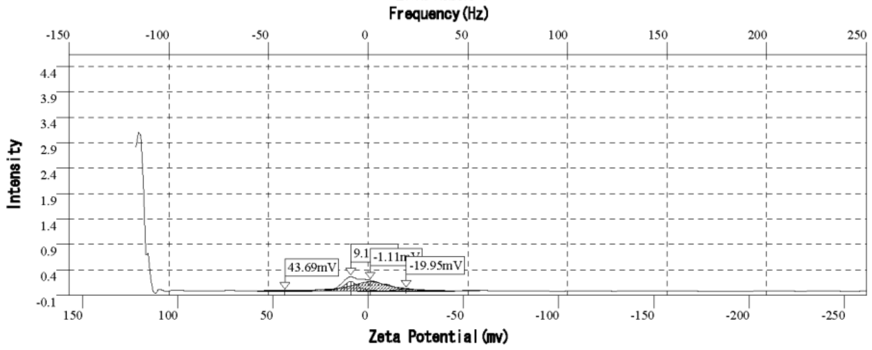

Data #2

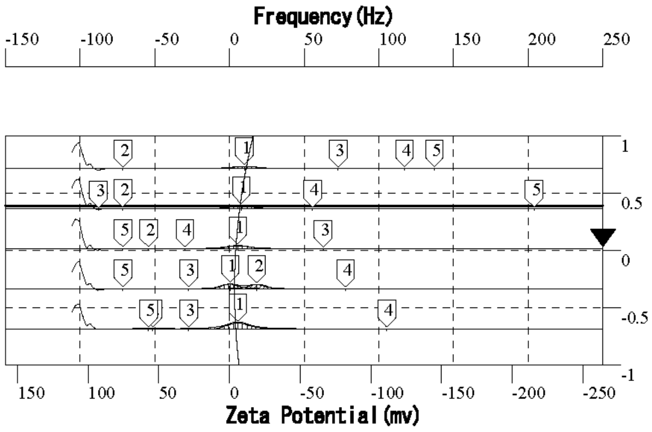

Zeta potential : −7.54 (mV)

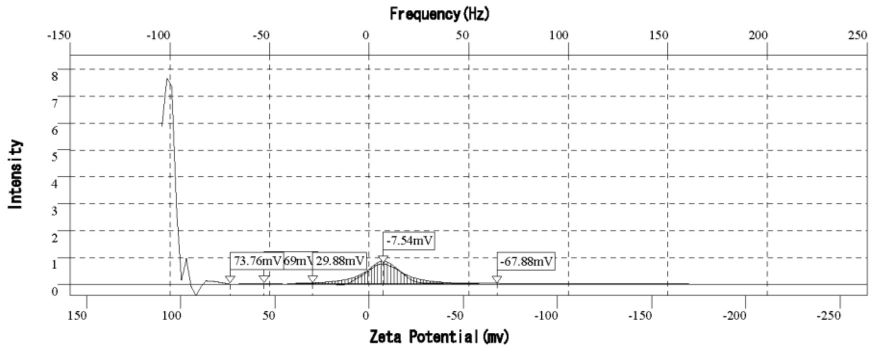

Data #3

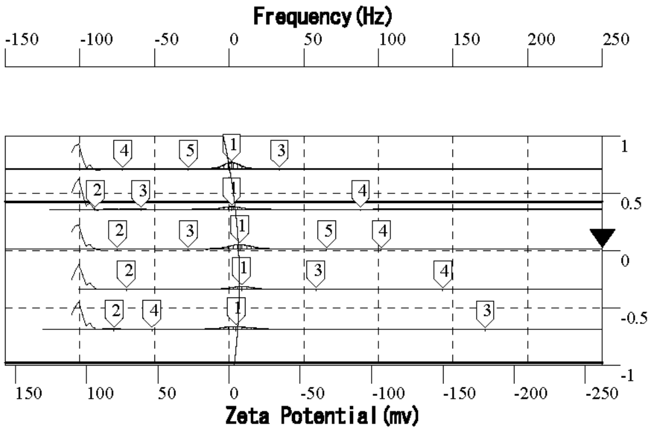

Zeta potential : −3.81 (mV)

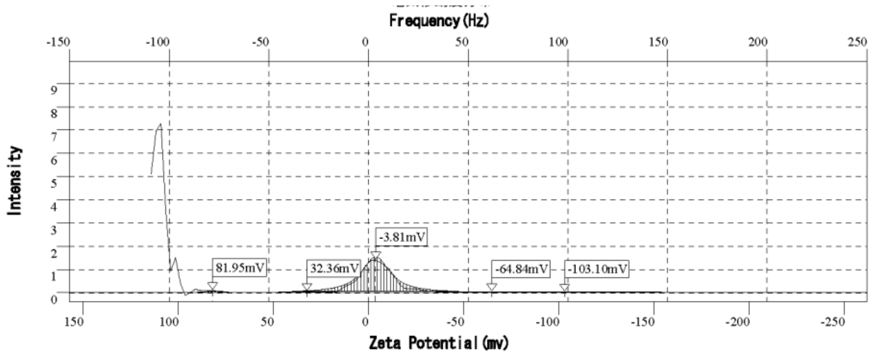

Data #4

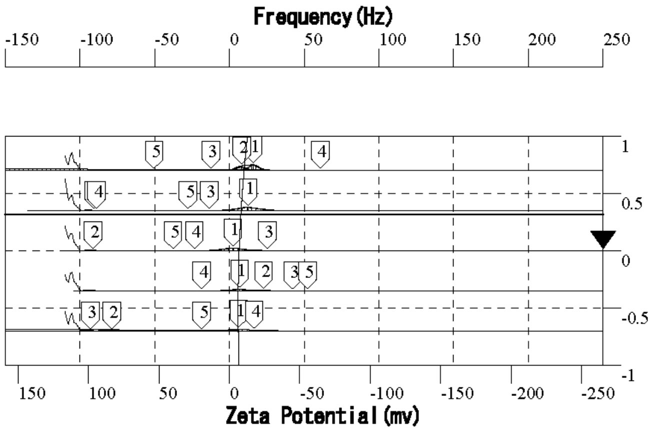

Zeta potential : −8.17 (mV)

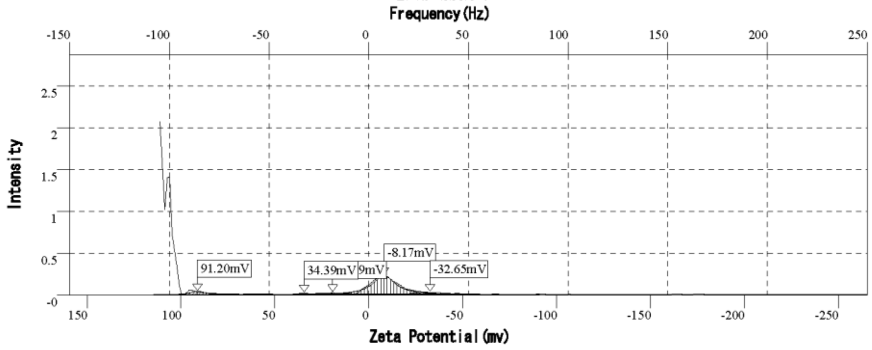

Data #5

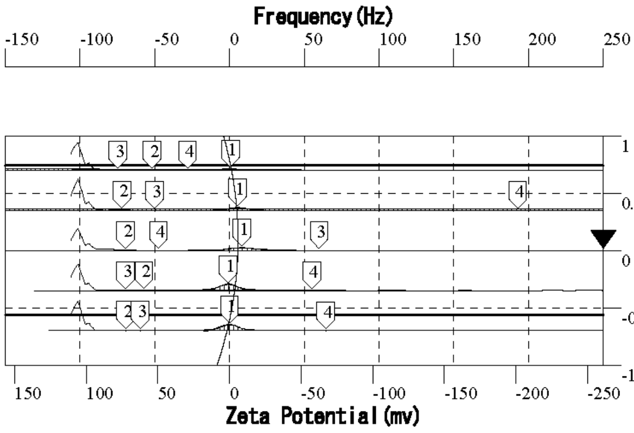

Zeta potential : −0.80 (mV)

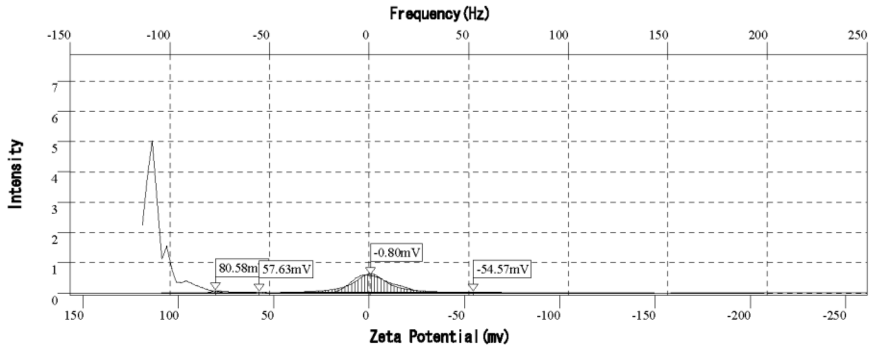

Zeta Potential of monomethyl malonate K (**5b'**) 50 mM

Electro-osmosis plot: Mobility distribution inside cell

Data #1

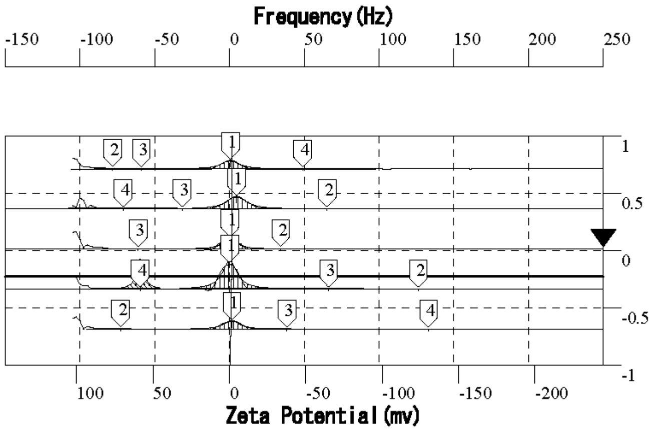

Zeta potential : −1.42 (mV)

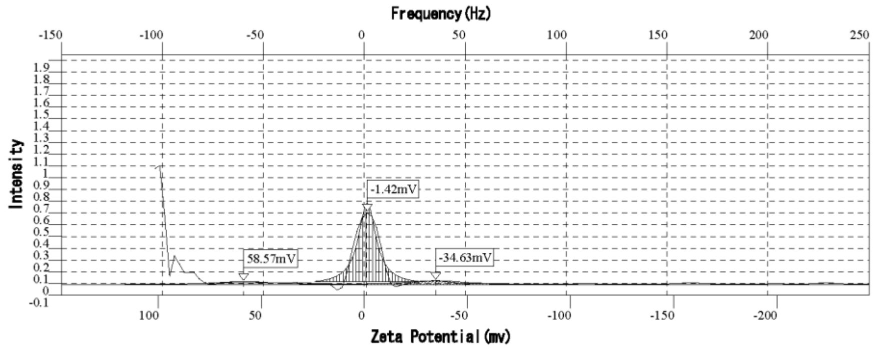

Data #2

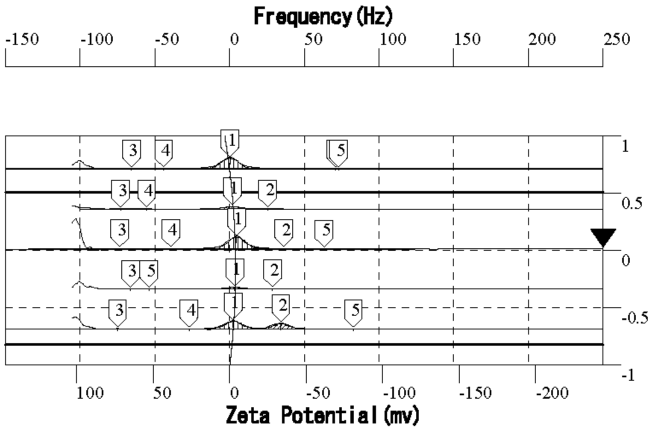

Zeta potential : −1.67 (mV)

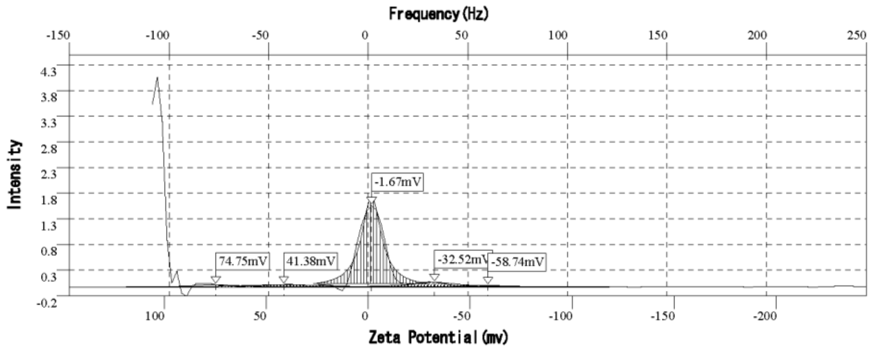

Data #3

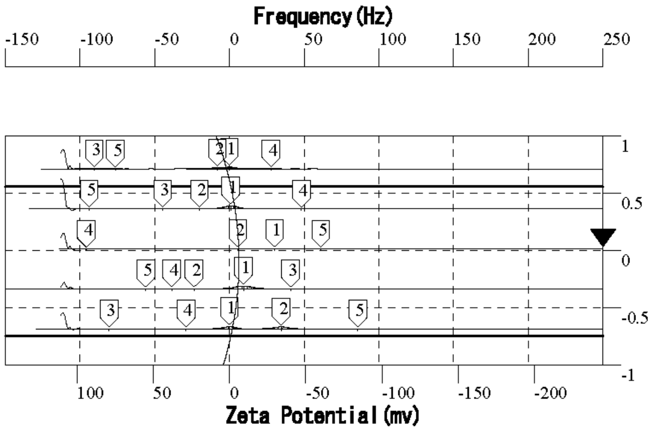

Zeta potential : −0.98 (mV)

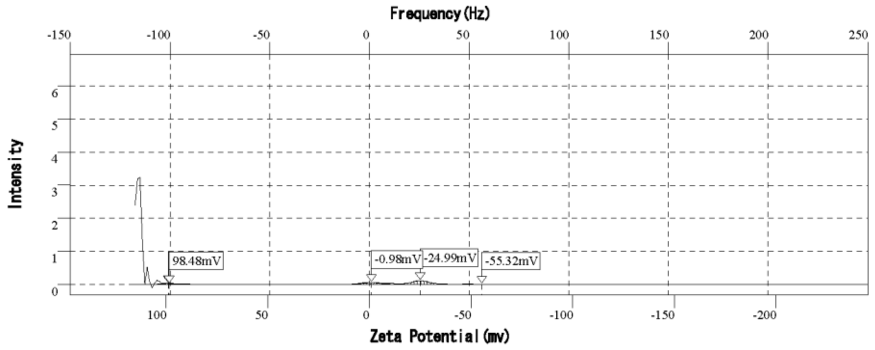

Data #4

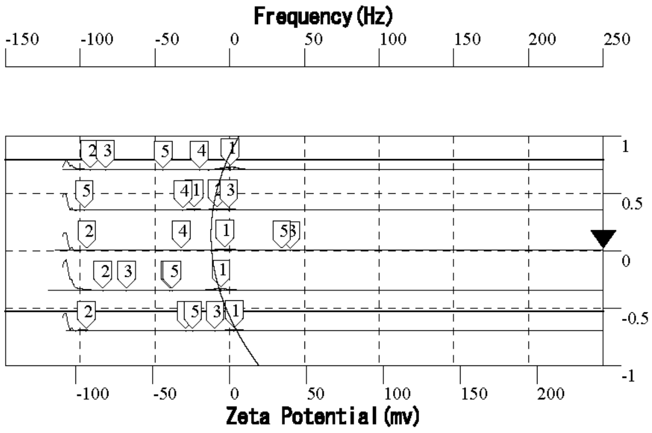

Zeta potential : −1.51 (mV)

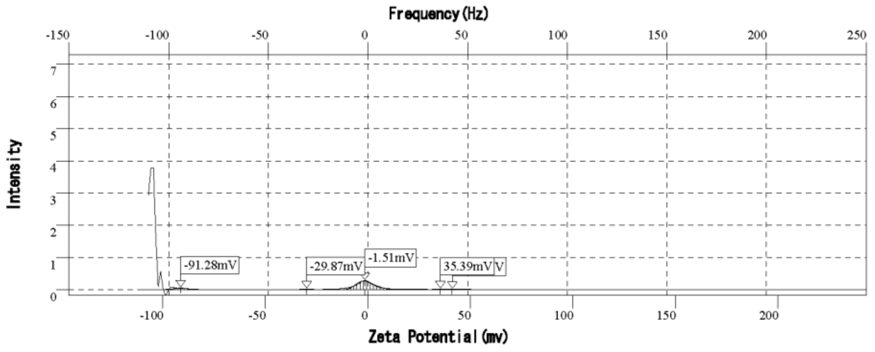

Data #5

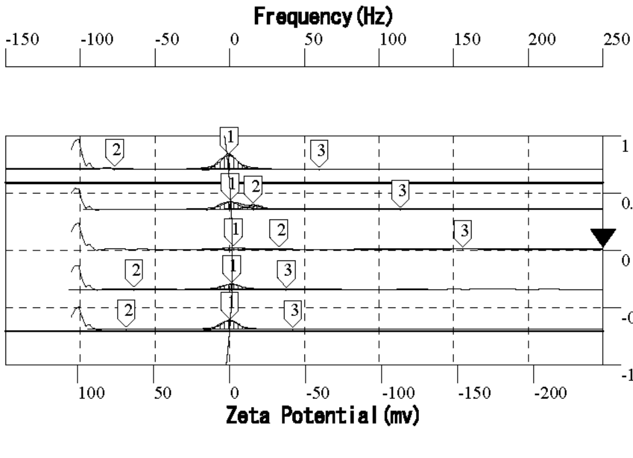

Zeta potential : 0.02 (mV)

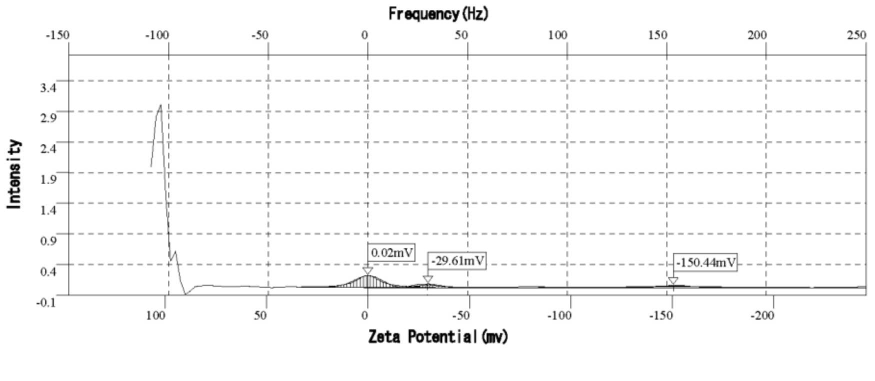

Zeta Potential of monomethyl malonate K (**5b'**) 20 mM

Electro-osmosis plot: Mobility distribution inside cell

Data #1

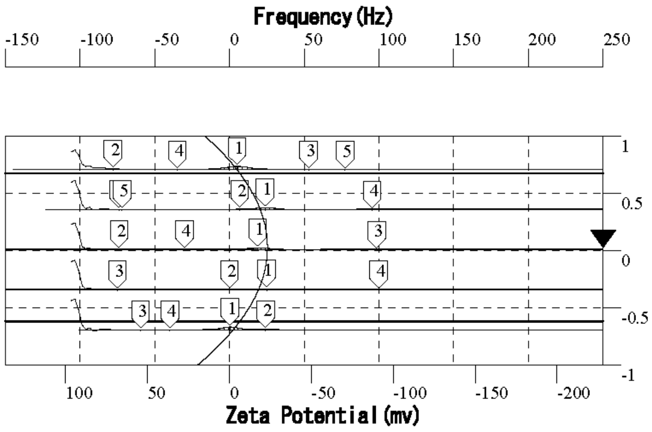

Zeta potential : −6.36 (mV)

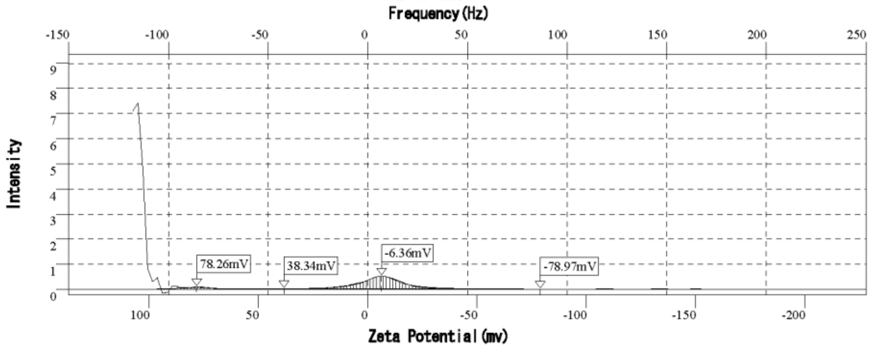

Data #2

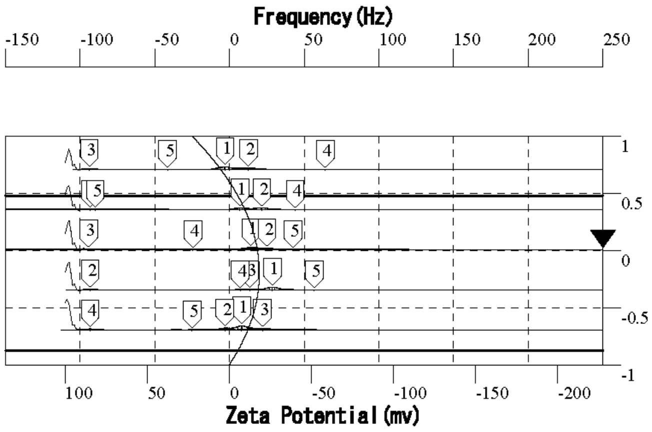

Zeta potential : −5.17 (mV)

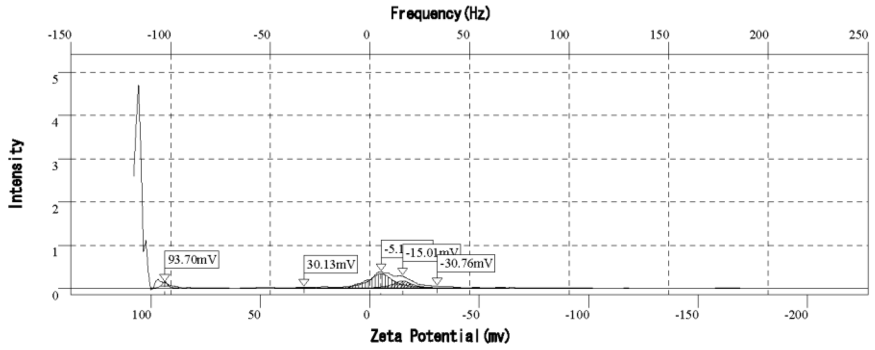

Data #3

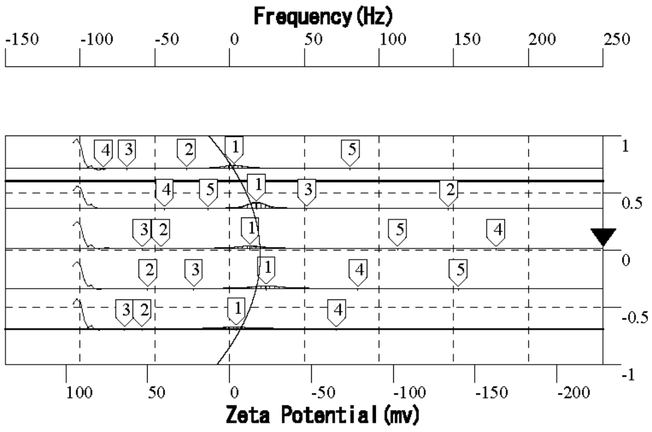

Zeta potential : −6.68 (mV)

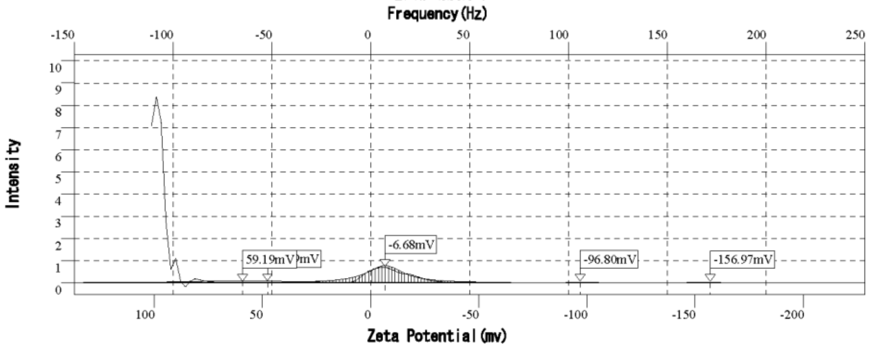

Data #4

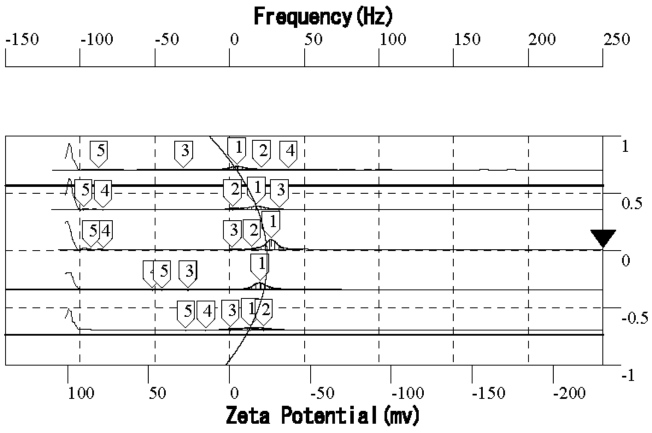

Zeta potential : −10.38 (mV)

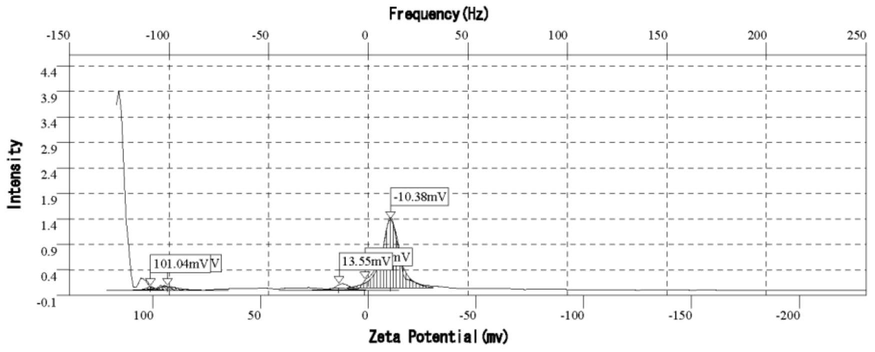

Data #5

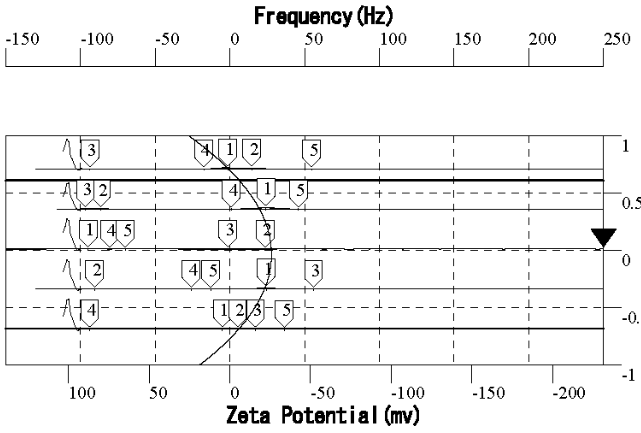

Zeta potential : −5.82 (mV)

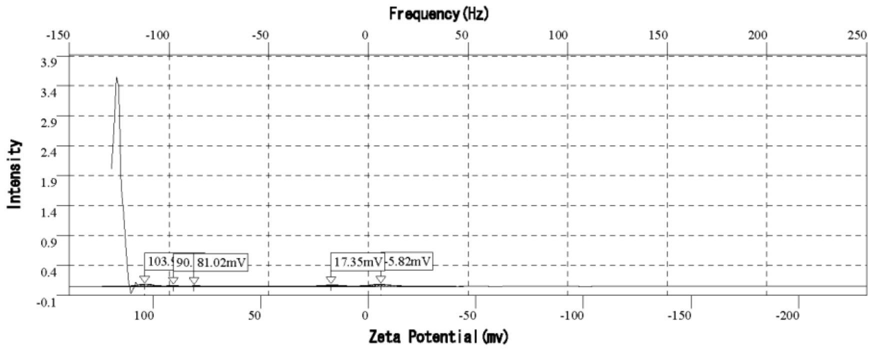

Zeta Potential of monomethyl malonate K (**5b'**) 10 mM

Electro-osmosis plot: Mobility distribution inside cell

Data #1

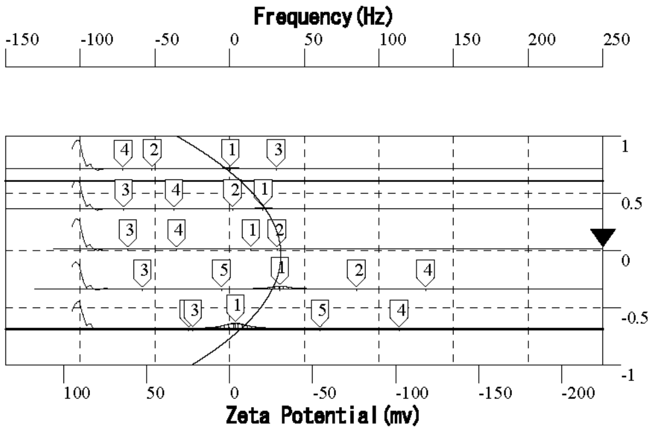

Zeta potential : −6.64 (mV)

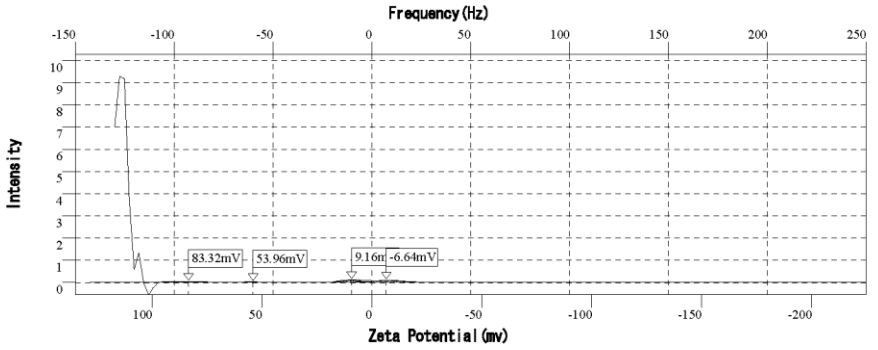

Data #2

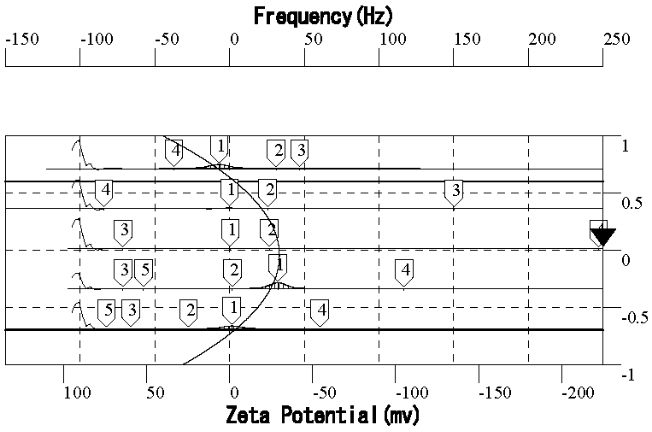

Zeta potential : −3.20 (mV)

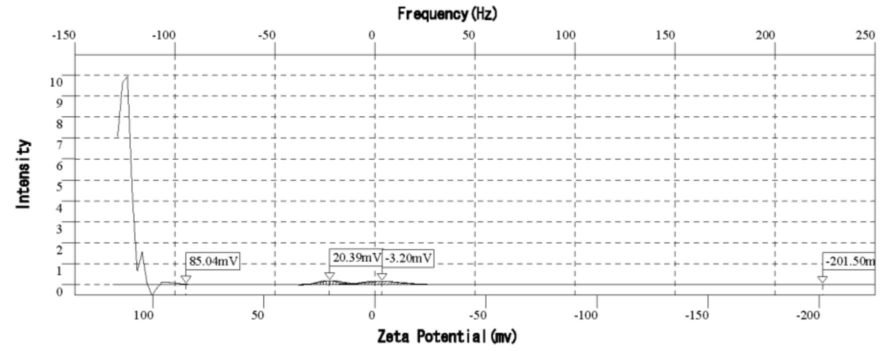

Data #3

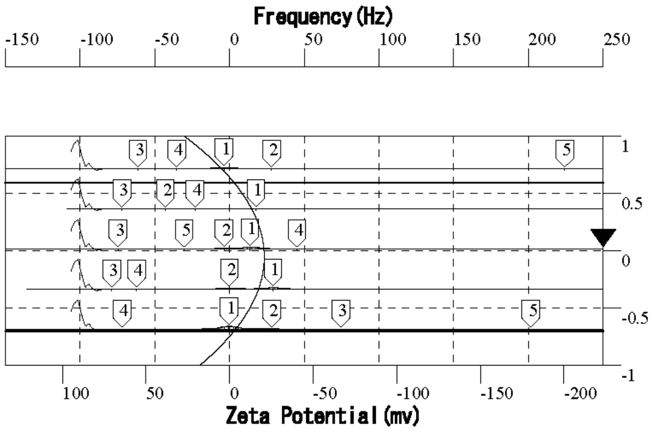

Zeta potential : −2.73 (mV)

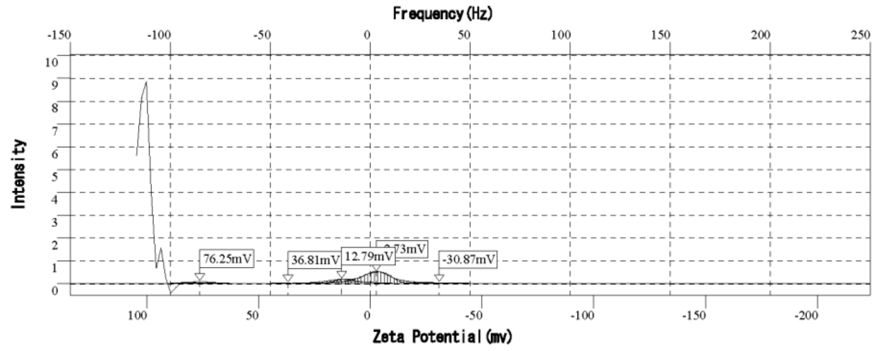

Data #4

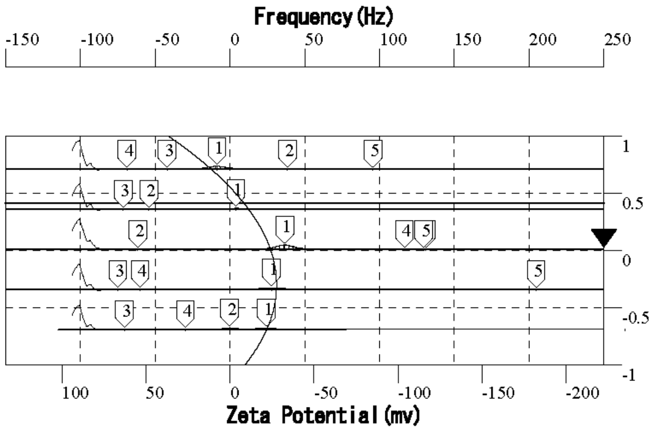

Zeta potential : −8.42 (mV)

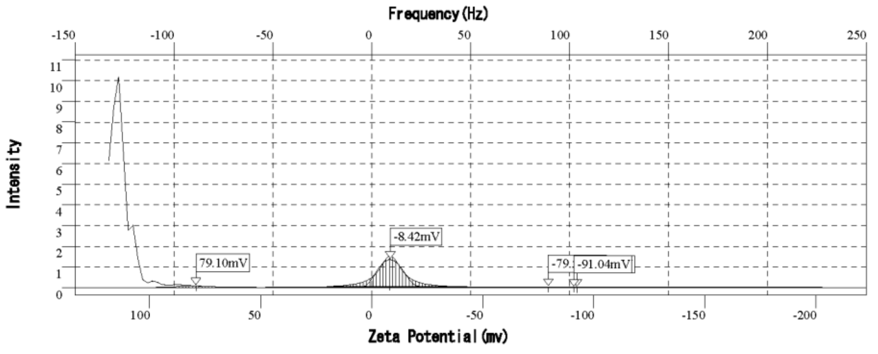

Data #5

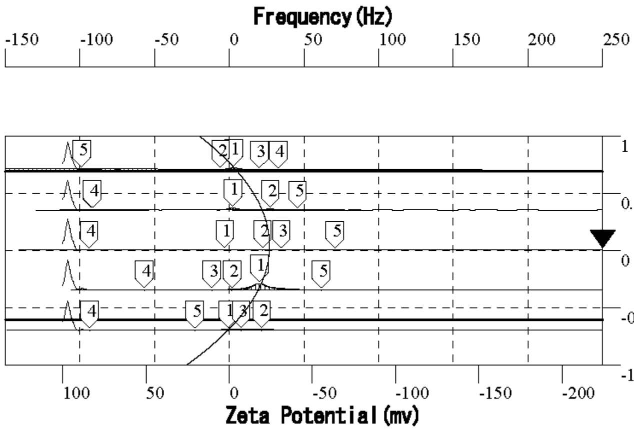

Zeta potential : −5.05 (mV)

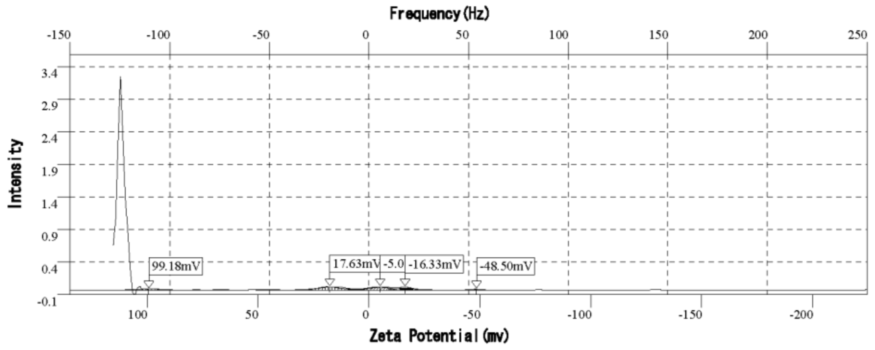

Supplement: Supplementary file 1 — ao3c04318_si_001.pdf [file ao3c04318_si_001.pdf]
